# Asymmetric synthesis of N–N axially chiral compounds *via* organocatalytic atroposelective *N*-acylation†‡

**DOI:** 10.1039/d1sc05360d

**Published:** 2021-11-24

**Authors:** Wei Lin, Qun Zhao, Yao Li, Ming Pan, Chen Yang, Guo-Hui Yang, Xin Li

**Affiliations:** State Key Laboratory of Elemento-Organic Chemistry, College of Chemistry, Nankai University Tianjin 300071 China xin_li@nankai.edu.cn

## Abstract

Compared with the well-developed C–C and C–N axial chirality, the asymmetric synthesis of N–N axial chirality remains elusive and challenging. Herein we report the first atroposelective *N*-acylation reaction of quinazolinone type benzamides with cinnamic anhydrides for the direct catalytic synthesis of optically active atropisomeric quinazolinone derivatives. This reaction features mild conditions and a broad substrate scope and produces N–N axially chiral compounds with high yields and very good enantioselectivities. Besides, the synthetic utility of the protocol was proved by a large scale reaction, transformation of the product and the utilization of the product as an acylation kinetic resolution reagent. Moreover, DFT calculations provide convincing evidence for the interpretation of stereoselection.

## Abstract 2

A highly efficient atroposelective *N*-acylation reaction of quinazolinone type benzamides with cinnamic anhydrides for the direct catalytic synthesis of optically active atropisomeric quinazolinone derivatives was developed.

## Graphical abstract

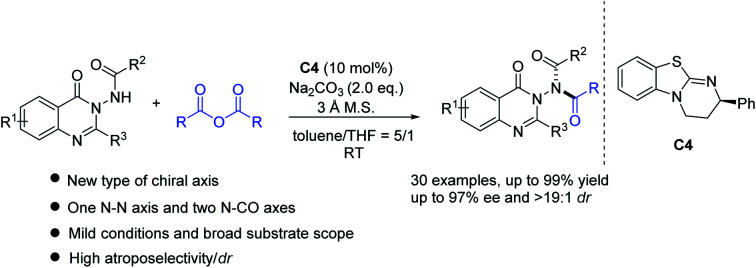

## Introduction

Axial chirality, which refers to stereoisomerism resulting from the nonplanar arrangement of four groups in pairs about a chiral axis (IUPAC), including atropisomerism, chiral allenes, spiranes, spiroindanes, and so on,^1^ has recently been investigated for its potential use in synthesis and asymmetric catalysis.^2^ The axially chiral unit was also deemed an important structural element of many bioactive molecules^3^ and natural products,^4^ enantiomers of which usually exhibit different pharmacological activities and metabolic processes. Atropisomerism, resulting from the restricted rotation around a single bond, which was firstly discovered by Christie and Kenner in 1922 for the investigation of 6,6-dinitrodiphenic acid, is the most representative subclass of axial chirality.^5^ Since the 1980s, with the development of enantiopure BINAP ligands in the field of enantioselective transition metal catalysis,^6^ the asymmetric synthesis of compounds with atropisomerism elements has attracted extensive attention.^7^ Since the introduction of chiral phosphoric acid with the binaphthyl skeleton in asymmetric catalysis by Terada and Akiyama in 2004,^8*a*,*b*^ especially the development of axially chiral imidodiphosphorimidate by List who won the Nobel Prize in 2021,^8*c*,*d*^ the past two decades have witnessed the explosive progress of atropisomeric compound-based chemistry and a series of efficient synthetic strategies have been developed for the construction of these type of compounds, which served as versatile building blocks for abundant privileged ligands and synthetically useful catalysts.^9^

**Scheme 1:**
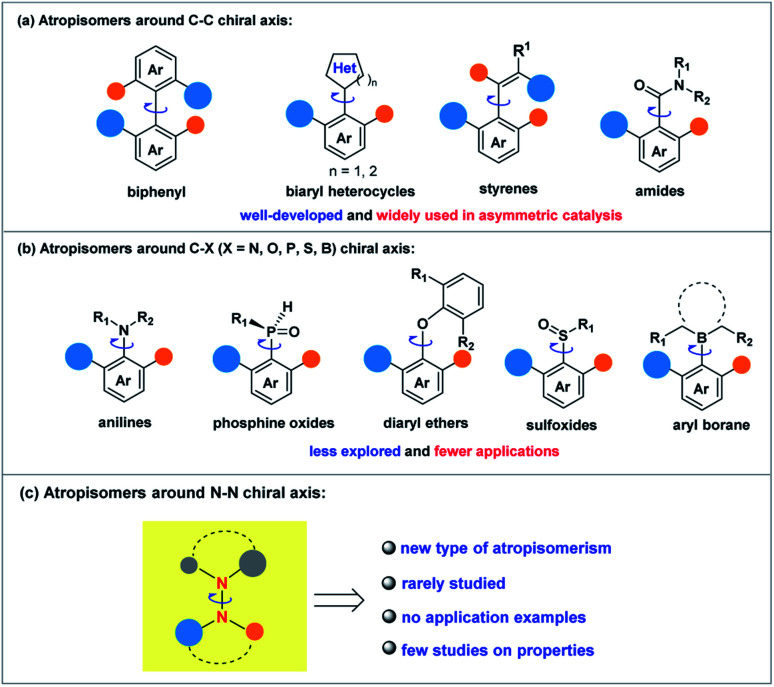
(a) Atropisomers around the C–C chiral axis. (b) Atropisomers around the C–X chiral axis. (c) Atropisomers around the N–N chiral axis.

**Scheme 2:**
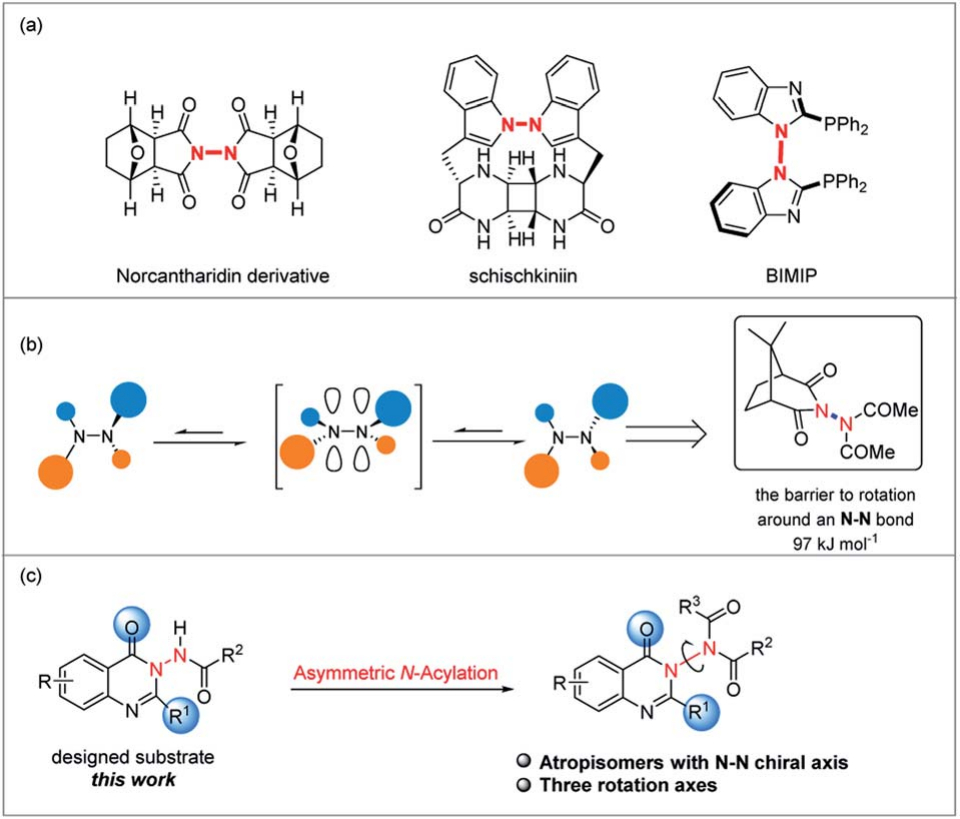
(a) Bioactive molecules, natural products and ligands containing the N–N axis. (b) The barrier to rotation around an N–N bond. (c) Our strategy for the enantioselective preparation of atropisomers with the N–N chiral axis (this work).

Although the related research fields have been greatly promoted, the mainly studied types of atropisomeric molecules are C–C axially chiral compounds represented by axially chiral biarylbenzene ring type compounds and axially chiral heteroaromatic compounds (Scheme 1a). In stark contrast, fewer studies on the synthesis and application of atropisomers with C–X (X = N, O, P, S, B) axes have been reported (Scheme 1b),^1,10^ probably due to the fact that compared to C–C axially chiral compounds, C–X atropisomers usually possess lower rotation barriers, leading to difficulties in the preservation of conformational stability. What is more, it is a pity that X–X atropisomers, such as atropisomers with the N–N chiral axis (Scheme 1c), have been extremely rarely studied to date.^11^ Just before the submission of this manuscript, the synthesis methods of N–N axially chiral compounds by allylic alkylation and Friedel–Crafts alkylation were reported.^12^ It is valuable to note that the increasing demand for enantioenriched axially chiral compounds in asymmetric catalysis and drug discovery has stimulated the development of not only efficient methods for these privileged scaffolds, but also the design and synthesis of new types of axially chiral compounds. The latter is more likely to make innovative contributions in fundamental research and related application exploration. For instance, N–N axially chiral skeletons exist widely in drug molecules (*e.g.*, norcantharidin derivative^13^) and natural products (*e.g.*, schischkiniin^14^) and are also used as ligands (*e.g.*, BIMIP^15^) (Scheme 2a). Therefore, the development of practical methods for the enantioselective preparation of atropisomers with the N–N chiral axis is very meaningful and highly desirable (Scheme 1c).

As is known, one reason for the racemization process of atropisomers is the rotation of the chiral axis that is governed by several factors such as steric hindrance around the chiral axis, electronic factors and so on.^16^ In fact, Verma and Prasad have shown that the barrier to rotation around an N–N bond is maximized when both nitrogens are acylated (*N*,*N*-diacetylaminocamphorimide is in excess of 97 kJ mol^−1^),^17^ and this increased barrier to rotation can be rationalised in terms of a destabilizing interaction in the transition state for rotation arising from the eclipsing of the filled orbitals on each sp^2^-hybridised nitrogen constituting the N–N bond (Scheme 2b). Based on the above-mentioned background, herein, we attempt to carry out the asymmetric catalytic synthesis of atropisomers with the N–N axis (Scheme 2c).

## Results and discussion

### Selection of the asymmetric catalytic reaction

**Scheme 3:**
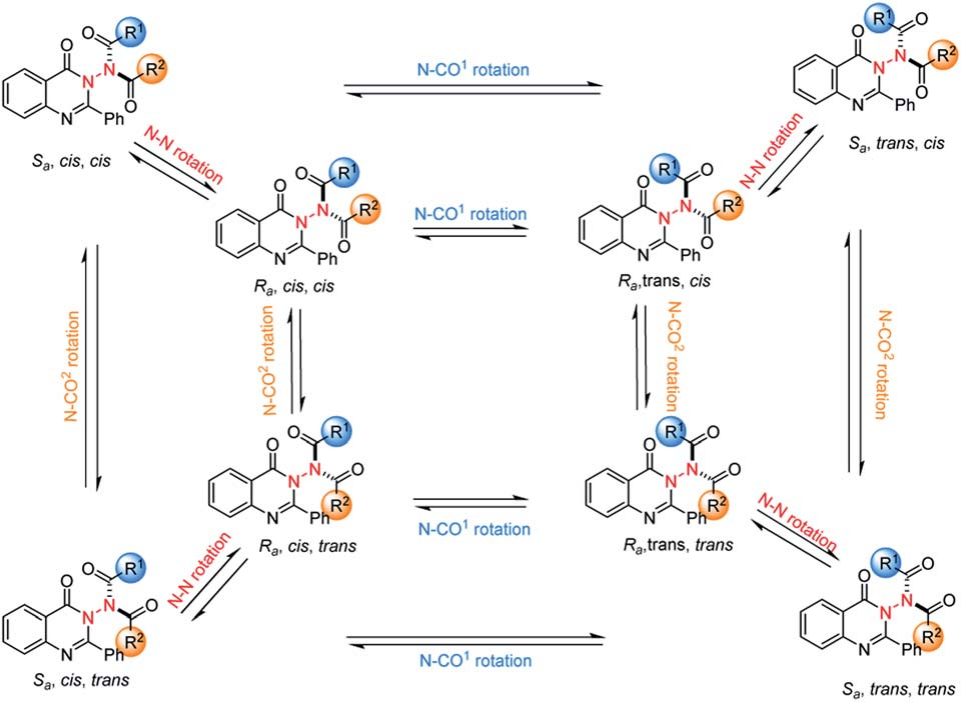
Challenges of the asymmetric synthesis of the currently studied chiral amides: two modes of rotation and three rotation axes (blue: N–CO^1^ rotation; orange: N–CO^2^ rotation; red, N–N rotation).

To probe the feasibility of this goal, we prepared compound 1a with quinazolin-4(3*H*)-one as the core structure, which was synthesized efficiently through a three-step procedure from cheap and readily available 2-aminobenzoate (see the ESI‡). With the substrate in hand, we intended to choose an organocatalytic asymmetric acylation reaction to construct N–N axially chiral compounds. The reason for choosing the acylation reaction is that there are three rotation axes in the target product, which makes the product theoretically have eight isomers (Scheme 3). It should be noted that the compounds with multiple chiral axes connected to the same atom are of great significance for enriching the types of axially chiral compounds.^18^ In 2014, a study of Miller, which researched a two-axis system containing C–N and Ar–CO axes, suggested that the enantiomeric pair around the Ar–CO axis was produced under the kinetic influence of a chiral catalyst, the *cis*/*trans* ratio was controlled by thermodynamics and the system equilibrium resulted from the interplay of kinetics and thermodynamics.^19^ Therefore, the challenges of the proposed atroposelective acylation strategy mainly stem from the following issues: (1) the reaction has to be conducted under mild conditions in order to ensure good asymmetric induction and maintain the chirality due to the relatively low atropostability of the resulting products. (2) The product in the final equilibrium state should have a high main isomer content. Moreover, to merit the goal of high enantiocontrol and economy, the chiral catalyst must be readily available and inexpensive.

### Optimization of the reaction conditions

With *N*-(4-oxo-2-phenylquinazolin-3(4*H*)-yl)benzamide 1a as the promised nucleophile, cinnamic anhydride 2a was selected as the reaction partner for the asymmetric acylation reaction (Table 1). Initially, the reaction of 1a and 2a was conducted with KO^*t*^Bu as the base in dichloromethane at room temperature for 16 h under the activation of chiral isothiourea C1.^20,21^ To our delight, the expected compound 3aa was obtained with 72% ee and >19 : 1 dr, albeit with 19% yield (Table 1, entry 1). Then isothioureas with different skeletons were examined. As a result, catalyst C4 gave the best 80% ee (Table 1, entries 1–4). Next, we investigated different bases to improve the reaction outcome. It was found that the weak inorganic bases had a positive effect on the reactivity and gave the product in a medium yield (Table 1, entries 5–8), in which Na_2_CO_3_ delivered the best result (68% yield with 82% ee and >19 : 1 dr). To further improve the enantioselectivity and yield, we turned our attention to screen reaction media. The results indicated that the solvent had a significant effect on the reaction (Table 1, entries 9–14). Comprehensively considering the yield and enantioselectivity, THF gave the best 99% yield with 90% ee of 3aa (Table 1, entry 9). When a 3 Å molecular sieve was added to the reaction, the enantiomeric excess value increased to 91% (Table 1, entry 15). Gratifyingly, when a solvent mixture of toluene and THF (v/v = 5 : 1) was used, the enantioselectivity of 3aa was further increased to 95% ee (Table 1, entry 16).

**Table 1:** Optimization of the reaction conditions^a^

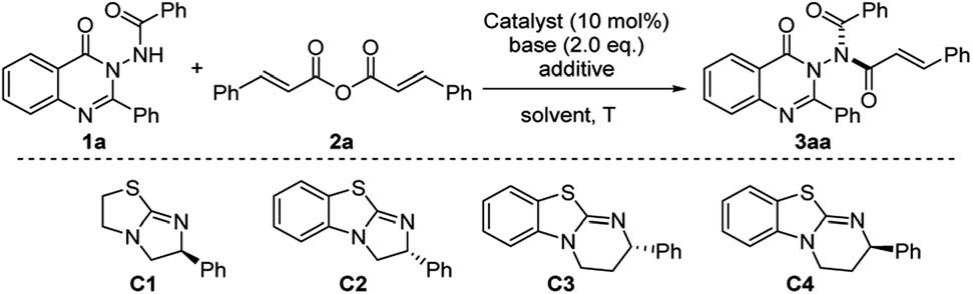						
Entry	Cat.	Solvent	Base	Yield^b^ (%)	ee^c^ (%)	dr^d^
1	C1	DCM	KO^*t*^Bu	19	72	>19 : 1
2	C2	DCM	KO^*t*^Bu	23	70	>19 : 1
3	C3	DCM	KO^*t*^Bu	34	74	>19 : 1
4	C4	DCM	KO^*t*^Bu	29	80	>19 : 1
5	C4	DCM	K_2_CO_3_	62	80	>19 : 1
6	C4	DCM	Na_2_CO_3_	68	82	>19 : 1
7	C4	DCM	Cs_2_CO_3_	45	79	>19 : 1
8	C4	DCM	NaO^*t*^Bu	>99	68	>19 : 1
9	C4	THF	Na_2_CO_3_	99	90	>19 : 1
10	C4	Toluene	Na_2_CO_3_	96	92	>19 : 1
11	C4	CH_3_CN	Na_2_CO_3_	17	43	>19 : 1
12	C4	Ether	Na_2_CO_3_	92	92	>19 : 1
13	C4	CHCl_3_	Na_2_CO_3_	63	88	>19 : 1
14	C4	DME	Na_2_CO_3_	69	90	>19 : 1
15^e^	C4	THF	Na_2_CO_3_	99	90	>19 : 1
**16** e ^,^ f	**C4**	**Toluene/THF = 5/1**	**Na**_**2**_ **CO**_**3**_	**90**	**95**	**>19 : 1**

a Reaction conditions: a mixture of 1a (0.1 mmol), 2a (0.15 mmol), base (2.0 equiv.) and catalyst (10 mol%) in solvent (1.0 mL) was stirred at room temperature for 16 h.

b Isolated yields.

c Determined by HPLC analysis.

d Only one dominant diastereomer was formed, and the ratio of dominant isomer to all other geometric isomers (dr) was >19 : 1, which was determined by ^1^H NMR analysis.

e 3 Å M.S. (20.0 mg).

f Reaction time of 24 h.

### Substrate scope

With the optimal reaction conditions in hand, we investigated the scope of quinazolinone 1 and the results are summarized in Table 2. We first examined the substituents on the phenyl group of the quinazolinone skeleton. Both electron-withdrawing groups and electron-donating groups in different positions were well tolerated with this asymmetric acylation reaction, and the corresponding N–N axially chiral products 3aa–3ga were obtained in moderate to very good yields (57–99%) with excellent stereoselectivities (>19 : 1 dr and 91–97% ee). The acyl moiety bearing different groups was also compatible, regardless of the phenyl rings with different types of substituents or alkyl substituents, and the desired products (3ha–3oa) were afforded with excellent enantioselectivities (85–95% ee). Furthermore, aryl and alkyl groups could be presented on the 2-position of quinazolinone, generating the corresponding atropisomeric quinazolinones 3pa–3sa in 62–95% yields with 89–95% ee and >19 : 1 dr under the standard conditions. To further demonstrate the generality of this strategy, drug-derived acyl groups were installed into the N–N axially chiral product. As a result, the desired atropisomeric quinazolinones bearing indomethacin and naproxen skeletons were prepared with good results (3ta, 39% yield, 90% ee and >19 : 1 dr; 3ua, 47 yield, 83% de and 5 : 1 dr).

**Table 2:** Scope of *N*-(4-oxo-quinazolin-3(4*H*)-yl)benzamides^a^

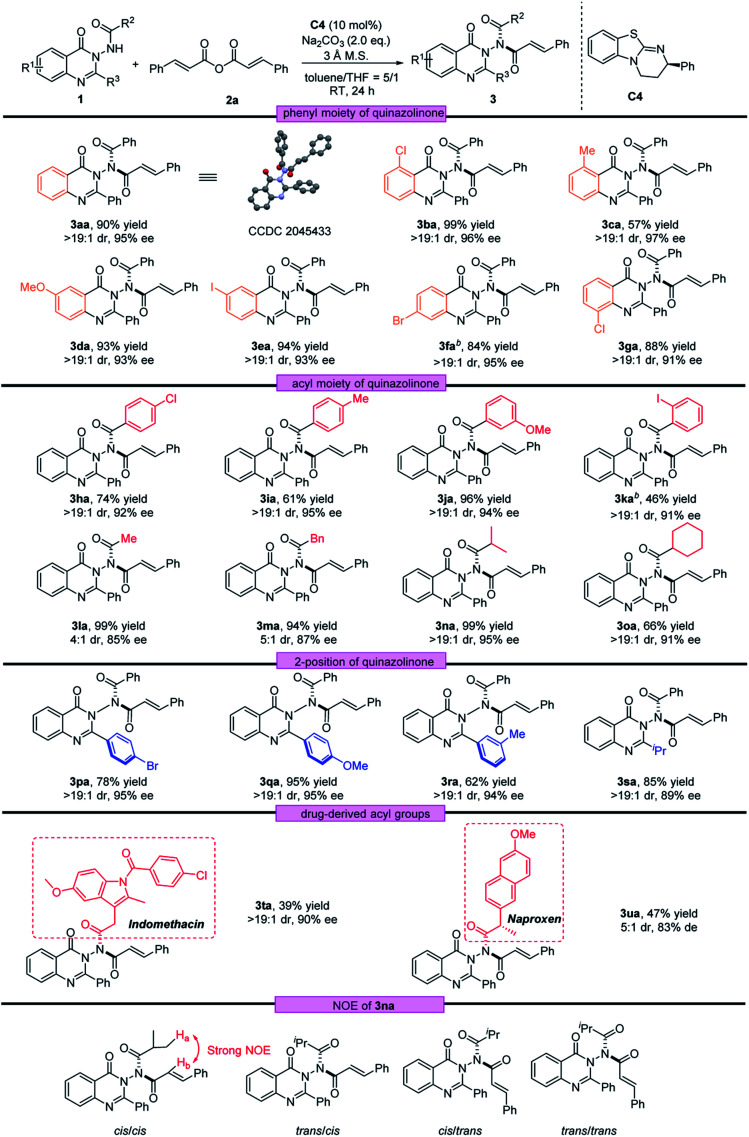

a Reaction conditions: a mixture of 1 (0.1 mmol), 2a (0.15 mmol), C4 (10 mol%), Na_2_CO_3_ (2.0 equiv.) and 3 Å M.S. (20.0 mg) in toluene/THF (5/1, 1.0 mL) was stirred at room temperature for 24 h.

b Reaction time was 48 h.

Next, using 1a as the template, a diversity of anhydrides 2 were examined. As shown in Table 3, anhydrides 2 containing either electron-withdrawing or electron-donating groups on the phenyl ring were investigated, and the asymmetric acylation strategy worked efficiently, furnishing the corresponding N–N axially chiral products 3ab–3ae in 71–96% yields with 89–94% ee and >19 : 1 dr. In addition, the naphthyl group and fused ring were also well tolerated at anhydride, providing the corresponding acylation products 3af and 3ag in 96% ee and 94% ee, respectively. Furthermore, the reaction conditions were also suitable for saturated anhydrides, giving the desired products 3ah–3aj with satisfactory outcomes (58–92% yield, 92–93% ee and >19 : 1 dr). The absolute configuration of 3aa was determined by X-ray crystallography to be (*R*_a_, *cis*, *cis*) configuration, and that of other products was assigned by analogy.^22^ To determine the *cis*/*cis* conformation, we conducted a NOESY experiment of 3na in CDCl_3_. According to NOESY spectra, strong NOE between H_a_ and H_b_ only exists in the *cis*/*cis*-isomer (see the ESI‡ for details).

**Table 3:** Scope of anhydrides^a^

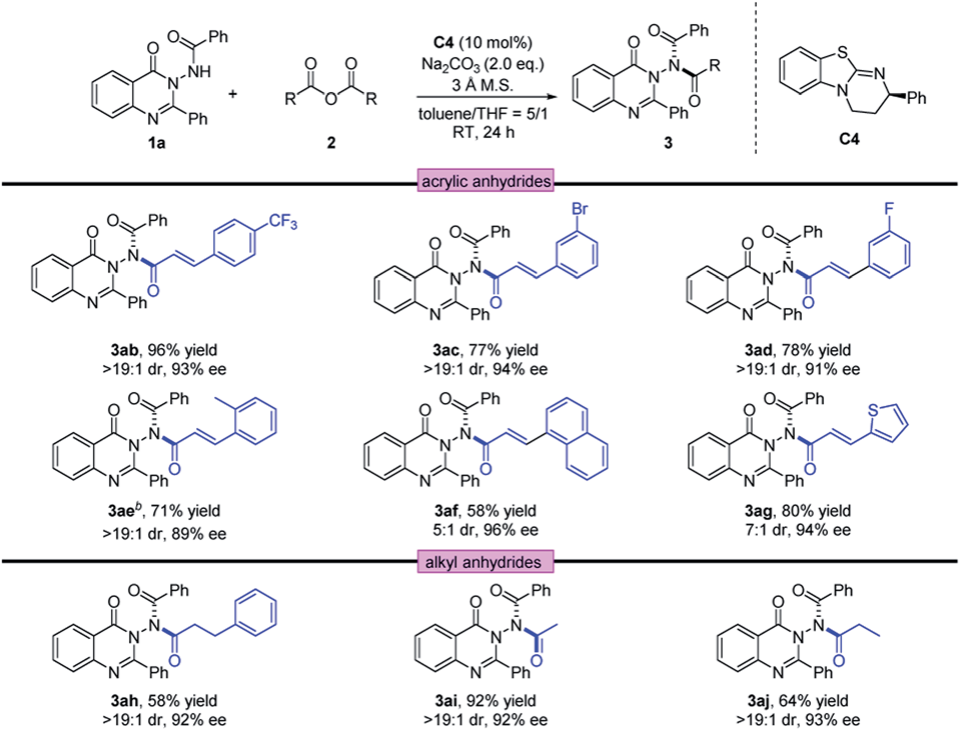

a Reaction conditions: a mixture of 1 (0.1 mmol), 2a (0.15 mmol), C4 (10 mol%), Na_2_CO_3_ (2.0 equiv.) and 3 Å M.S. (20.0 mg) in toluene/THF (5/1, 1.0 mL) was stirred at room temperature for 24 h.

b The reaction was conducted with 20 mol% of C4 for 72 h.

### Racemization experiment

**Scheme 4:**
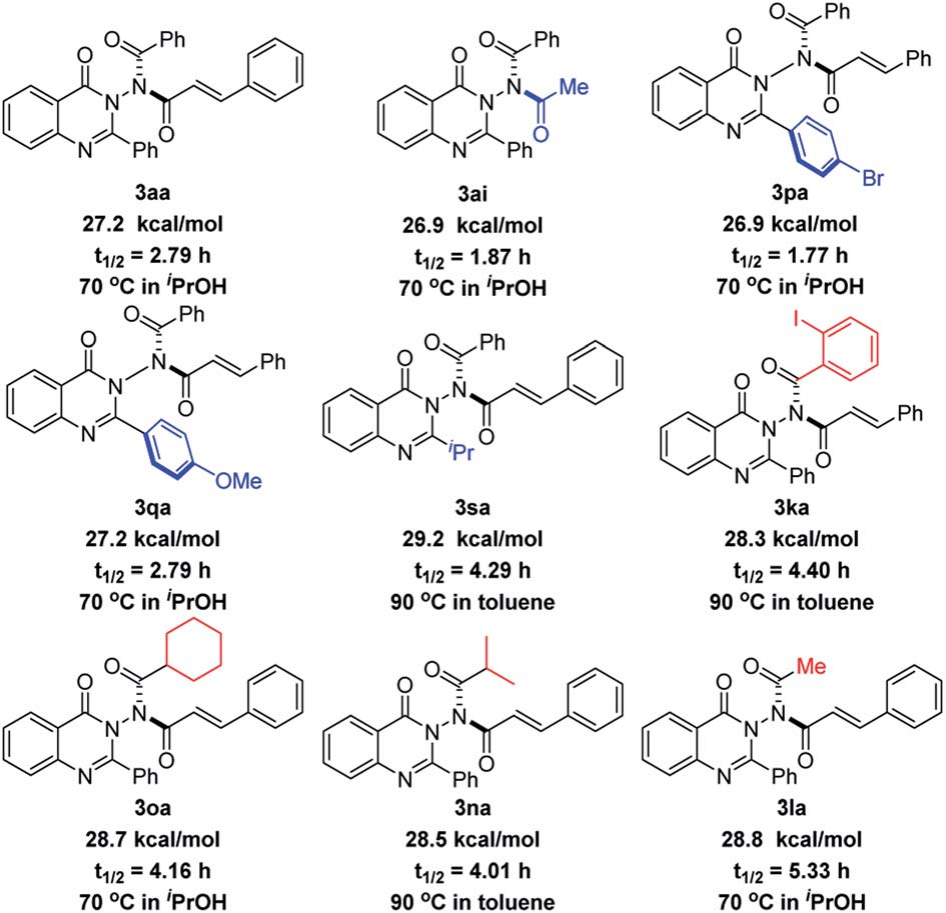
The rotation barriers and half-times of N–N axially chiral products.

In order to examine the stereochemical stability of these N–N axially chiral products, we performed the racemization experiment. As shown in Scheme 4, the comparison of the rotation barrier of 3aa (27.2 kcal mol^−1^), 3ai (26.9 kcal mol^−1^), and 3sa (29.2 kcal mol^−1^) suggested that there was no obvious effect on the stereochemical stability of the acyl group originating from anhydrides but it is closely related to the steric hindrance of the substituent at the quinazolinone 2-position. Electronic properties also had a little impact on the rotation barrier, which was demonstrated by the values of 3pa (26.9 kcal mol^−1^) and 3qa (27.2 kcal mol^−1^). The 1.1 kcal mol^−1^ energy gap between 3aa and 3ka indicated that *ortho*-substituted phenyl possessing a large steric hindrance in the acyl moiety of quinazolinone could give a better stability. It is worth noting that 3la with a small steric methyl group gives a relatively high 28.8 kcal mol^−1^ rotation barrier. The reason for this phenomenon is maybe due to the presence of diastereoisomers that resulted from the rotation of the C–N bond (4 : 1 dr, Table 3), in which the methyl group was closer to the carbonyl group of quinazolinone, increasing the steric hindrance. The rotation barriers of 3aa and 3sa were also studied by DFT calculations (see the ESI‡ for details). As a result, 27.7 kcal mol^−1^ for 3aa and 28.4 kcal mol^−1^ for 3sa were obtained, respectively, which agrees well with the experimental result.

### Large-scale reaction and transformation

**Scheme 5:**
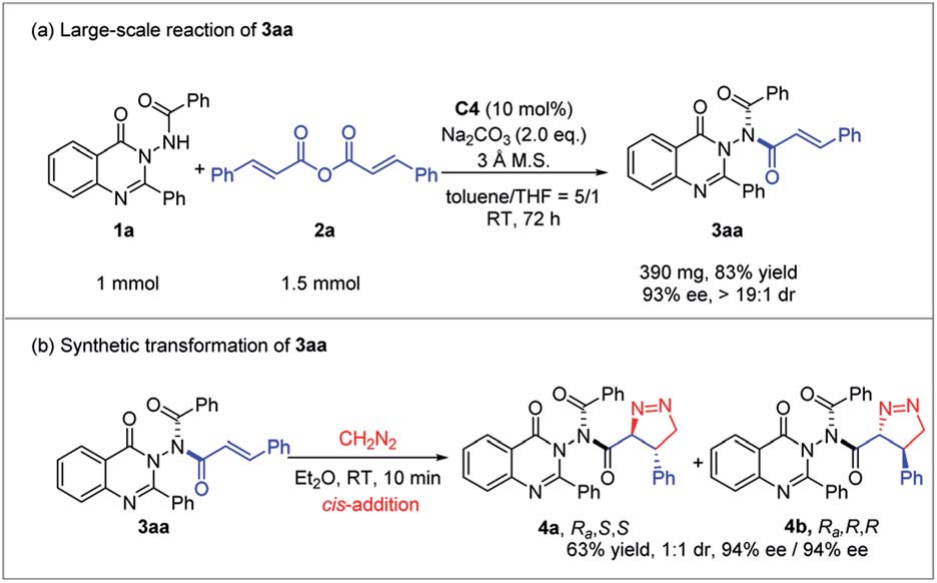
The large-scale reaction and transformation.

To evaluate the practical utility of this methodology, the reaction of 1a and 2a was conducted on a 1 mmol scale under the optimized conditions (Scheme 5). To our delight, the desired N–N axially chiral quinazolinone 3aa was obtained in 83% yield with 93% ee and >19 : 1 dr. Furthermore, 3aa could react with diazomethane through a 1,3-dipolar cycloaddition process which was a *cis*-addition way to furnish product 4 bearing C-centered chirality and the N–N chiral axis in 63% yield with 94% ee, albeit with 1 : 1 diastereoselectivity (*S*/*S*, *R*/*R*).

### Application of N–N axially chiral quinazolinone as an acylating reagent

**Scheme 6:**
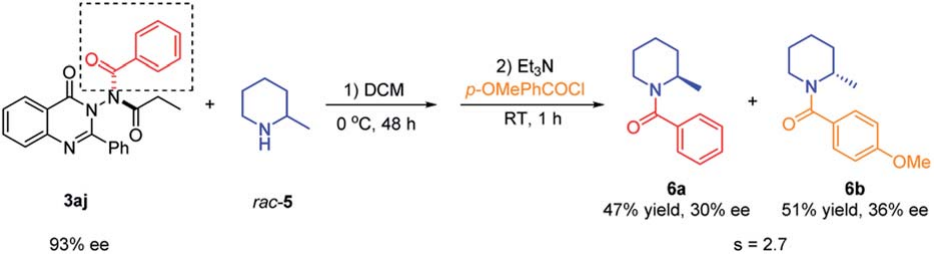
Kinetic resolution of racemic 2-methylpiperidine with N–N axially chiral quinazolinone as the chiral reagent.

To demonstrate the application of the synthesized N–N axially chiral quinazolinones, the kinetic resolution of racemic 2-methylpiperidine^23^ with 3aj as the chiral acylation source was attempted. As shown in Scheme 6, the desired product *N*-benzoylamide 6a was generated in 47% yield with 30% ee, and unreacted 2-methylpiperidine was converted to *p*-methoxy benzoylamide 6b in 51% yield with 36% ee. Although the selectivity factor was only 2.7, this represented the first example of the utilization of N–N axially chiral compounds as chiral reagents, indicated that the N–N axially chiral scaffold seemed promising for applying in the field of chiral synthesis.

### Exploration of other asymmetric *N*-nucleophilic reactions

**Scheme 7:**
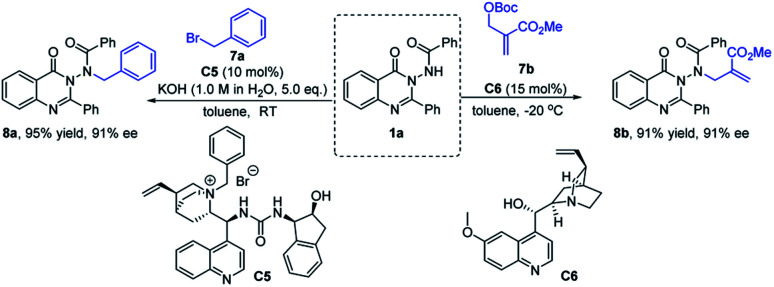
Asymmetric *N*-alkylation reaction and asymmetric *N*-allylic alkylation reaction of 1a.

In order to further explore the potential of substrate 1a in the synthesis of N–N axially chiral compounds, other asymmetric *N*-nucleophilic reactions were preliminarily attempted. As shown in Scheme 6, 1a reacted smoothly with benzyl bromide 7a by using catalyst C5 under phase transfer catalysis, delivering the corresponding N–N axially chiral product 8a in 95% yield with 91% ee. Moreover, through a quinidine-catalyzed asymmetric allylic alkylation reaction of 1a and achiral MBH adduct 7b, 8b was obtained in 91% yield with 91% ee (Scheme 7). These results not only enrich the types of N–N axially chiral compounds containing quinazolinone units, but also prove the universality of the asymmetric *N*-nucleophilic reaction in the construction of N–N axially chiral compounds.

### Theoretical calculations

**Fig. 1:**
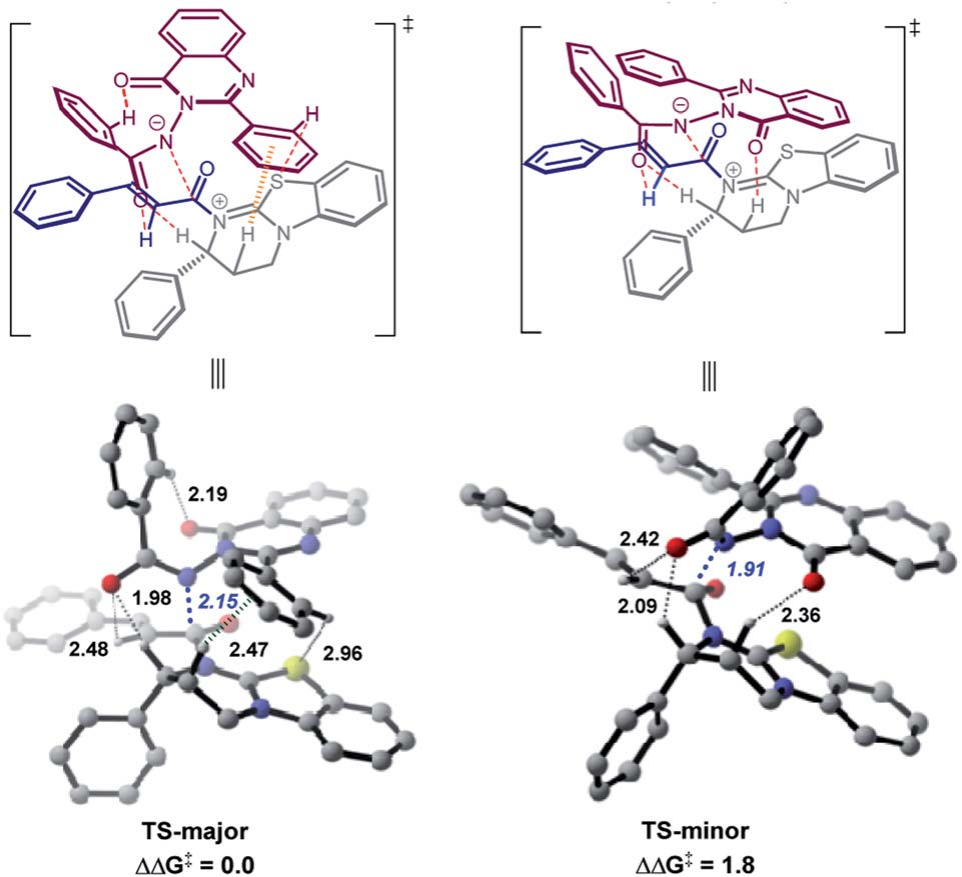
Calculated transition state structures and their relative free energies catalyzed by C4. Energies are given in kcal mol^−1^. Bond lengths are given in Å.

We performed theoretical calculations^24^ to elucidate the origins of the atroposelective *N*-acylation reaction by locating the critical C–N bond-formation transition states. Two most stable transition states for the enantiomers are obtained at the B97D3/def2TZVPP-SMD(toluene)//M06-2X/6-31g(d) level.^25–27^ As we can see from Fig. 1, the energy difference between TS-major and TS-minor is 1.8 kcal mol^−1^, predicting a 90% ee value. The calculations are in good agreement with the experiments (Table 1, entry 10, 92% ee).

**Fig. 2:**
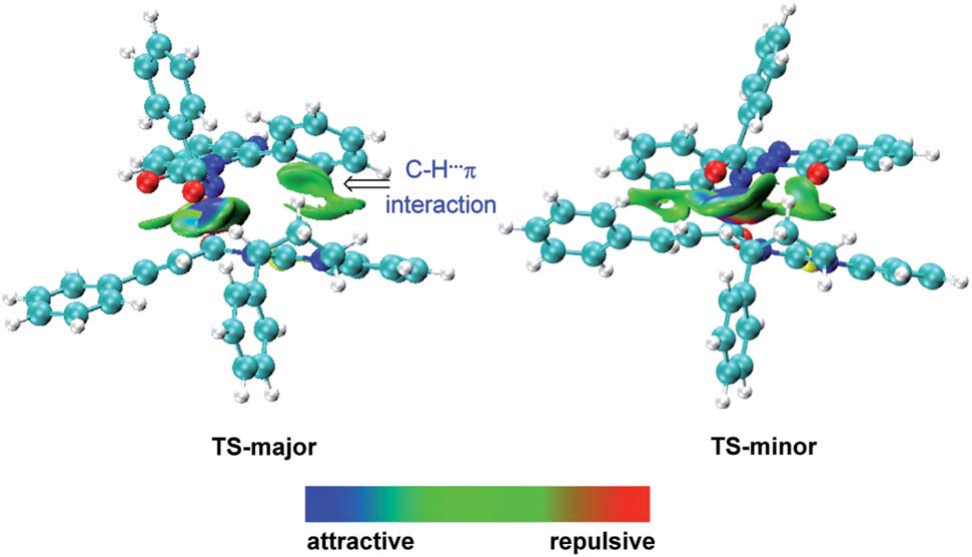
Independent Gradient Model (IGM) analysis of intermolecular interaction in transition states.

Recently, pioneered by Yang and coworkers,^28^ the visualization of noncovalent interactions has become a powerful tool to reveal both attractive and repulsive interactions by the color of reduced density gradient isosurfaces. In our study, multiple noncovalent interactions exist in both of the transition states after close examination of the two structures, which can be further proved by Independent Gradient Model (IGM) analysis (Fig. 2 and S3‡).^29,30^ In both transition states, the benzoyl involves two aliphatic C–H⋯O hydrogen bonds (1.98 and 2.48 Å *vs.* 2.09 and 2.42 Å), respectively. The carbonyl of quinazolinone also acts as a hydrogen bond acceptor to stabilize transition states. Furthermore, there is an intramolecular hydrogen bond between the aromatic C–H of benzoyl and the carbonyl of quinazolinone in TS-major (2.19 Å), which is stronger than the intermolecular hydrogen bond between the aliphatic C–H and carbonyl of quinazolinone (2.36 Å). Besides, based on the IGM analysis of TS-major (Fig. 2, left), a C–H⋯π interaction (marked in green in Fig. 1) and weak C–H⋯S interaction (2.96 Å, Fig. S3‡) make contributions to the stability. These two noncovalent interactions are not presented in TS-minor. Thus, from the above analysis, we could believe that the cooperation of multiple noncovalent interactions plays key roles in the stereocontrol.

## Conclusion

In summary, a new class of axially chiral compounds, named N–N axially chiral compounds, were synthesized by an asymmetric *N*-acylation reaction for the first time. It was worth mentioning that the product contains two rotatable N–CO axes in addition to the N–N axis, which made the stereoselectivity control of the reaction very challenging. The substrate scope was substantial, and a series of quinazolinone derivatives with novel N–N axially chiral scaffolds were synthesized in excellent yields (up to 99%) with high stereoselectivities (up to >19 : 1 dr, up to 97% ee) *via* a simple chiral isothiourea catalysis under mild conditions. The synthetic utility of the protocol was proved by a large scale reaction, transformation of the product and the utilization of the product as an acylation kinetic resolution reagent. Computational studies were conducted to elucidate the origins of the stereoselectivity of the *N*-acylation reaction.

## Data availability

All experimental and computational data is available in the ESI.‡

## Author contributions

W. L., G.-H. Yang and X. L. developed the reaction. W. L., Q. Z. and M. P. expanded the substrate scope, performed the synthetic applications and characterized all the products. Y. L. and C. Y. performed the theoretical calculations. X. L. directed the investigations. W. L., Q. Z., Y. L. and X. L. wrote the manuscript.

## Conflicts of interest

There are no conflicts to declare.

## Supplementary Material

SC-013-D1SC05360D-s001

SC-013-D1SC05360D-s002
