# Supplementary material for: Asymmetric synthesis of N–N axially chiral compounds via organocatalytic atroposelective N-acylation
Source: Chem Sci. 2021 Nov 24;13(1):141–8. doi: 10.1039/d1sc05360d (PMC8694391; doi:10.1039/d1sc05360d)
Supplement: SC-013-D1SC05360D-s001 [file SC-013-D1SC05360D-s001.pdf]

Electronic Supplementary Material (ESI) for Chemical Science.
This journal is © The Royal Society of Chemistry 2021

Supplemental Information

Asymmetric Synthesis of N-N Axially Chiral Compounds via Organocatalytic Atroposelective *N*-Acylation

Wei Lin, Qun Zhao, Yao Li, Ming Pan, Chen Yang, Guo-Hui Yang and Xin Li

Table of Contents

1. General Information and Starting Materials..S3

2. General Procedure for the Synthesis of **3aa**.. S4

3. Lage Scale Reaction and Synthetic Transformation.....................................S4

4. Procedure for Kinetic Resolution of Racemic 2-Methylpiperidines by **3aj**...S5

5. Racemization Experiment...S5

6. Calculation of the Rotation Barrier of **3aa** and **3sa**...................................... S12

7. Characterization Data.. S19

8. NOE for Compound **3na**.. S33

9. HPLC Analysis.. S34

10. NMR Analysis...S69

11. X-ray Analysis.. S104

13. Computational Methods..S106

12. References.. S113

1. General Information and Starting Materials

General Information. Commercial reagents were used as received, unless otherwise stated. ^{1}H and ^{13}C NMR were recorded on a Bruker-DPX 400 spectrometer. Chemical shifts are reported in ppm from tetramethylsilane with the solvent resonance as the internal standard. The following abbreviations were used to designate chemical shift mutiplicities: s = singlet, d = doublet, t = triplet, q = quartet, h = heptet, m = multiplet, br = broad. All first-order splitting patterns were assigned on the basis of the appearance of the multiplet. Splitting patterns that could not be easily interpreted are designated as multiplet (m) or broad (br). ^{19}F NMR were recorded on a Varian NMR 400 spectrometer. Mass spectra were obtained using electrospray ionization (ESI) mass spectrometer.

General Procedure for the Synthesis of the Substrates

Procedure for the Synthesis of 3-Monoacylaminoquinazolinone 1

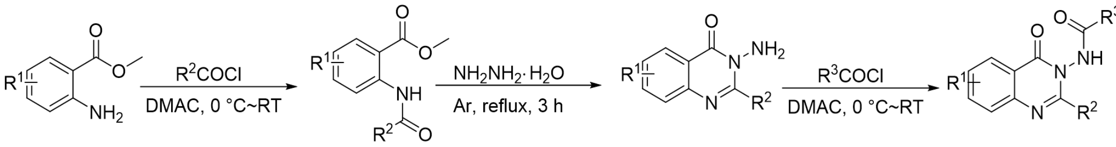

To a dry round-bottom flask equipped with a magnetic stir bar, was added 2-aminobenzoate (1.0 equiv) and DMAC (0.1 M), the mixture cooled to 0 ºC in an ice bath. Acid chloride (1.2 equiv) was added dropwise to the mixture and then stirred at room temperature for 3 hours. Upon completion of the reaction, water was poured into the solution, filtered and dried to give the **S1** as a white solid.

Under Ar, $NH_2NH_2 \cdot H_2O$ (0.1 M) and **S1** was heated to reflux for 3 h, after the reaction completed, water was poured into the solution, filtered and dried to give the **S2** as a white solid.

To a dry round-bottom flask equipped with a magnetic stir bar, was added **S2** (1.0 equiv) and DMAC (0.1 M), the mixture cooled to 0 ºC in an ice bath. Acid chloride (1.2 equiv) was added dropwise to the mixture and then stirred at room temperature for 3 hours. Upon completion of the reaction, water was poured into the solution, filtered and dried to give the **1** as a white solid.

Procedure for the Synthesis of Anhydrides 2[1]

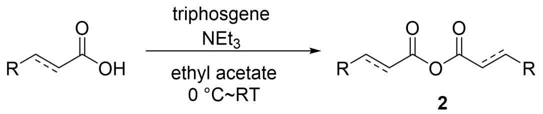

To a two-necked round-bottom flask under Ar atmosphere, the carboxylic acid (1.0 equiv) was dissolved in ethyl acetate (0.1 M). The solution was cooled to

0 °C and triethylamine (1.0 equiv) was added, followed by triphosgene (0.17 equiv). The solution was allowed to continue stirring at 0 °C for 10 minutes, before warming it up to 25 °C and allowed to stir for an additional 15 minutes. The solution was then filtered through celite to remove the salt and washed with ethyl acetate. Subsequently, the filtrate was evaporated to dryness under reduced pressure to afford the desired anhydride substrate **2**.

2. General Procedure for the Synthesis of 3aa.

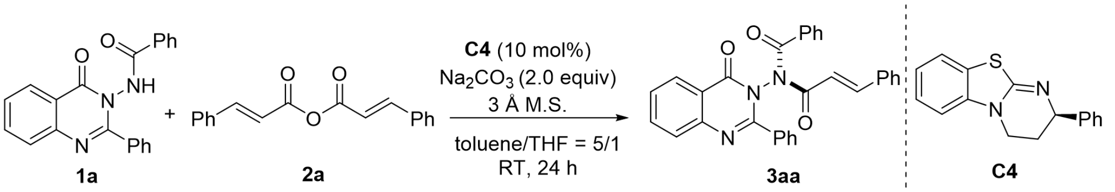

A mixture of *N*-(4-oxo-2-phenylquinazolin-3(4*H*)-yl)benzamide **1** (0.1 mmol), cinnamic anhydride **2** (0.15 mmol), Na_2CO_3 (2.0 equiv), 3 Å M.S. (20 mg) and **C4** (10 mol%) in toluene/THF = 5/1 (1.0 mL) was stirred at room temperature for 24 h, and then the solvent was removed under vacuum. The residue was purified by silica gel chromatography to yield the desired product **3aa** in 90% yield with >19:1 dr and 95% ee.

3. Lage Scale Reaction and Synthetic Transformation

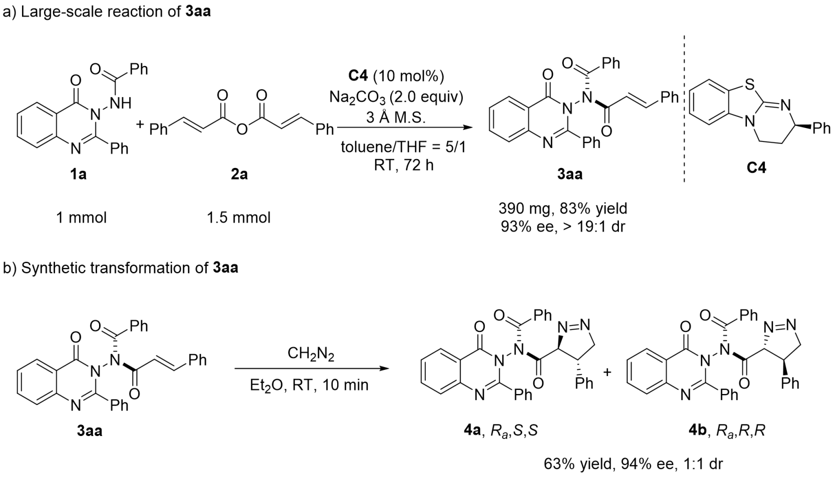

To a 25.0 mL round-bottom flask was added the *N*-(4-oxo-quinazolin-3(4*H*)-yl)benzamides **1a** (341.0 mg, 1.0 mmol), cinnamic anhydride **2a** (209.0 mg, 1.5 mmol), isothiourea catalyst **C4** (26.6 mg, 0.1 mmol), Na_2CO_3 (212.0 mg, 2.0 mmol), toluene/THF = 5/1 (10.0 mL) and 3 Å M.S. (200.0 mg). Then the reaction mixture was stirred at room temperature for 72 h. Upon completion of the reaction (monitored by TLC), the mixture was filtered to remove the 3 Å MS. The solvent was subsequently removed under reduced pressure and the crude mixture was purified by column chromatography on silica gel (Hexane/Ethyl Acetate = 8/1) to give the pure N-N axially chiral product **3aa** (390 mg, 83% yield, 93% ee).

To the flask containing the above **3aa (**0.1 mmol) was added the freshly prepared CH_2N_2 ether solution[2] (1.0 mL). Then the reaction mixture was stirred

at room temperature for 10 min on a magnetic stirrer. Upon the reaction completed, the mixture was concentrated under reduced pressure. The resulting crude residue was purified *via* column chromatography on silica gel to afford **4** (63% yield, 94% ee, 1:1 dr).

4. Procedure for Kinetic Resolution of Racemic 2-Methylpiperidines by 3aj

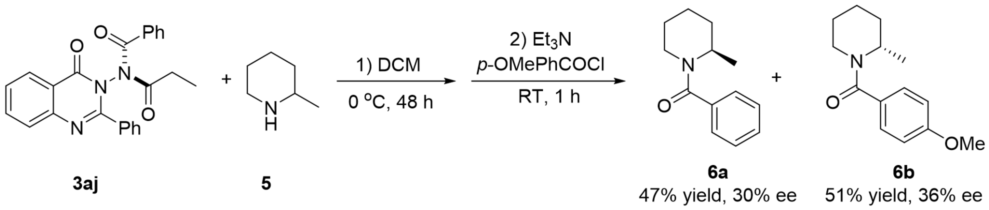

To a solution of **3aj** (39.7 mg, 0.1 mmol) and DCM (1.0 mL) was added 2-methylpiperidine **5** (19.8 mg, 0.2 mmol) at 0 °C. The mixture was stirred at the same temperature for 48 h. After accomplishment of kinetic resolution process, the 4-methoxybenzoyl chloride (42.5 mg, 0.25 mmol), Et_3N (28.28 mg, 0.28 mmol) were added, and then the mixture was stirred for extra 1 h at room temperature. The reaction was quenched with water and diluted with DCM (5 mL). The organic layer was washed by brine (2 × 5 mL), and the combined organic phase was dried over anhydrous Na_2SO_4. Evaporation of organic solvent and purification by column chromatography gave the corresponding product **6a** (19.1 mg, 47% yield, 30% ee) and **6b** (23.6 mg, 51% yield, 36% ee).

5. Racemization Experiment

Table S1a. Measured data of rotation barrier and half-life of **3aa**

P/M	(1+(P/M))/(1-(P/M))	t/(s)	ln
0.138	1.322	0	0.279
0.172	1.416	1920	0.348
0.236	1.618	3840	0.481
0.335	2.008	5760	0.697
0.356	2.109	7680	0.746
0.412	2.403	9600	0.877

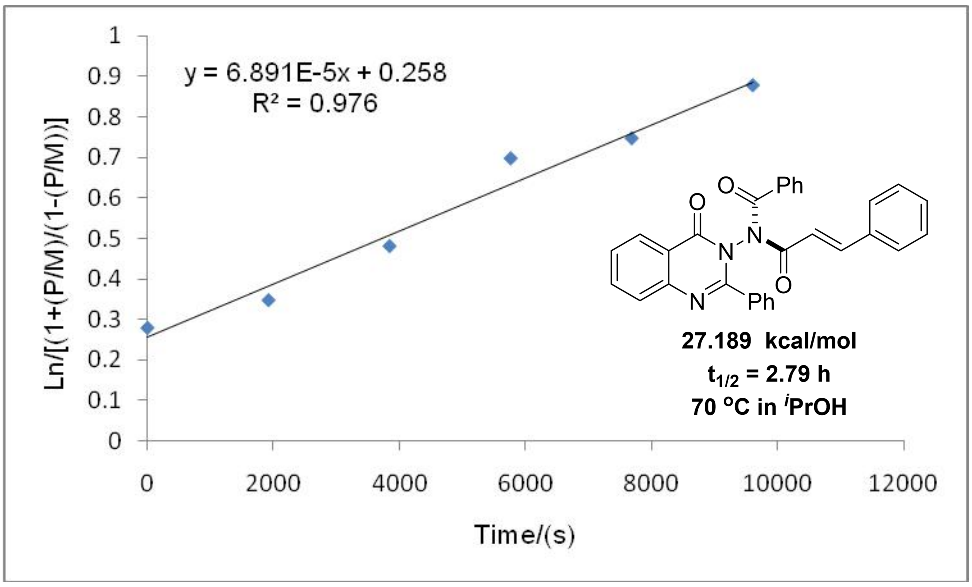

Figure S1a. rotating barrier and half-life of **3aa**

Table S1b. Measured data of rotation barrier and half-life of **3ai.**

P/M	(1+(P/M))/(1-(P/M))	t/(s)	ln
0.151	1.355	0	0.304
0.276	1.761	1200	0.566
0.316	1.923	2400	0.654
0.383	2.242	3600	0.807
0.410	2.392	4800	0.872
0.490	2.924	6000	1.073

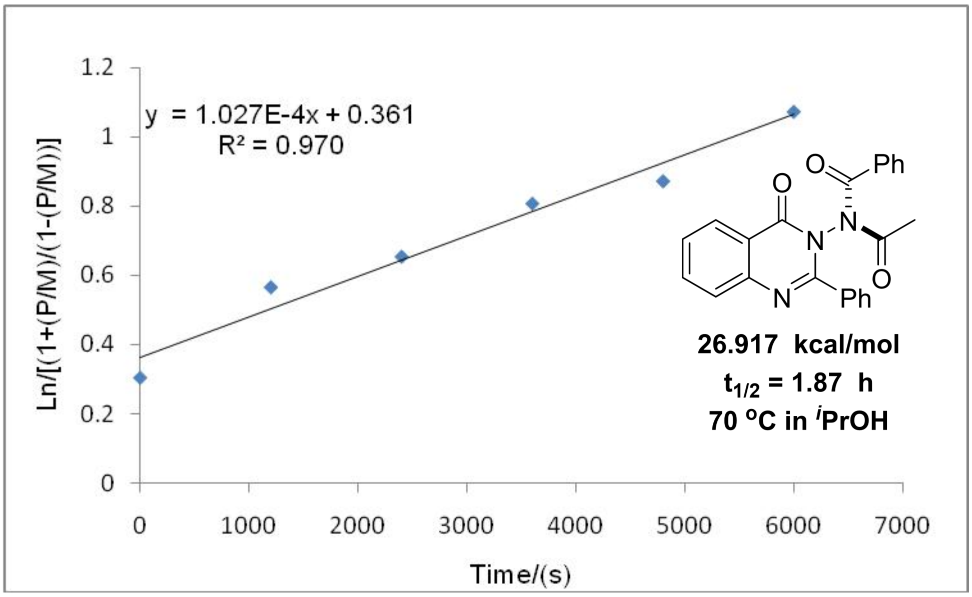

Figure S1b. rotating barrier and half-life of **3ai**

Table S1c. Measured data of rotation barrier and half-life of **3pa.**

P/M	(1+(P/M))/(1-(P/M))	t/(s)	ln
0.047	1.099	0	0.094
0.124	1.282	1440	0.248
0.192	1.475	2880	0.389
0.271	1.742	4320	0.555
0.344	2.049	5760	0.717
0.412	2.404	7200	0.877

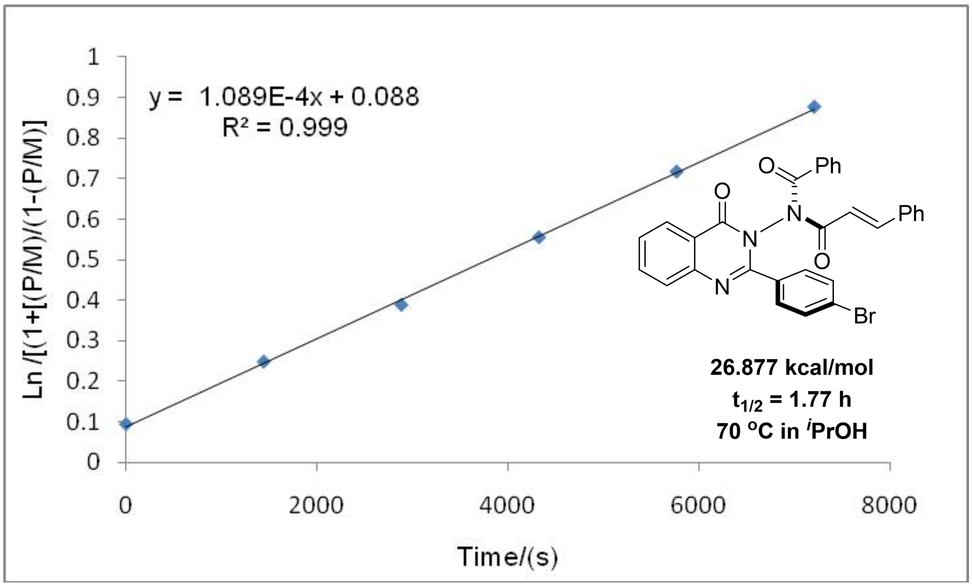

Figure S1c. rotating barrier and half-life of **3pa**

Table S1d. Measured data of rotation barrier and half-life of **3qa.**

P/M	(1+(P/M))/(1-(P/M))	t/(s)	ln
0.043	1.089	0	0.086
0.093	1.205	2100	0.186
0.160	1.381	4140	0.322
0.217	1.553	6120	0.440
0.277	1.767	8100	0.569
0.362	2.137	10200	0.759

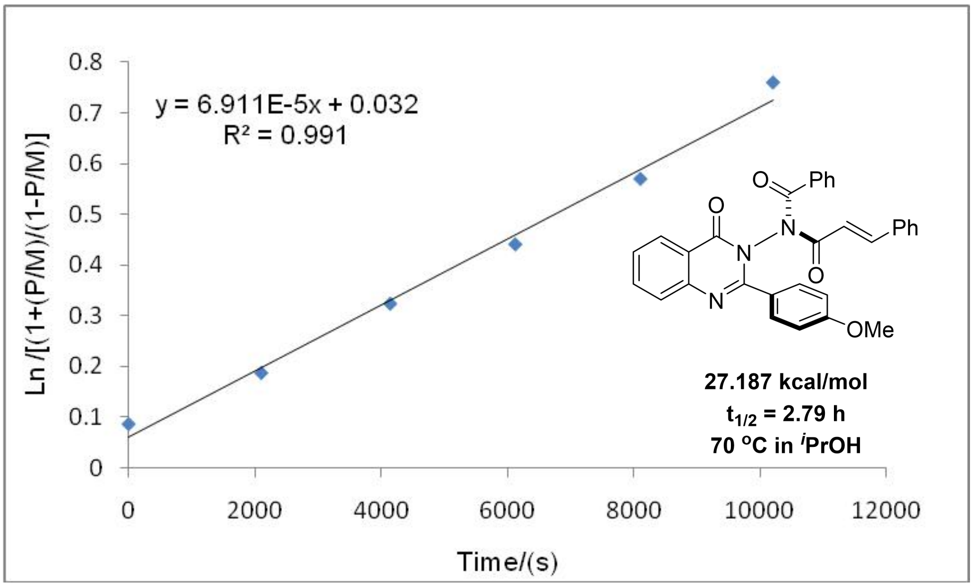

Figure S1d. rotating barrier and half-life of **3qa.**

Table S1e. Measured data of rotation barrier and half-life of **3sa.**

P/M	(1+(P/M))/(1-(P/M))	t/(s)	ln
0.055	1.116	0	0.110
0.099	1.219	1980	0.198
0.129	1.299	4080	0.261
0.174	1.420	6180	0.351
0.210	1.534	8280	0.428
0.255	1.684	10320	0.521

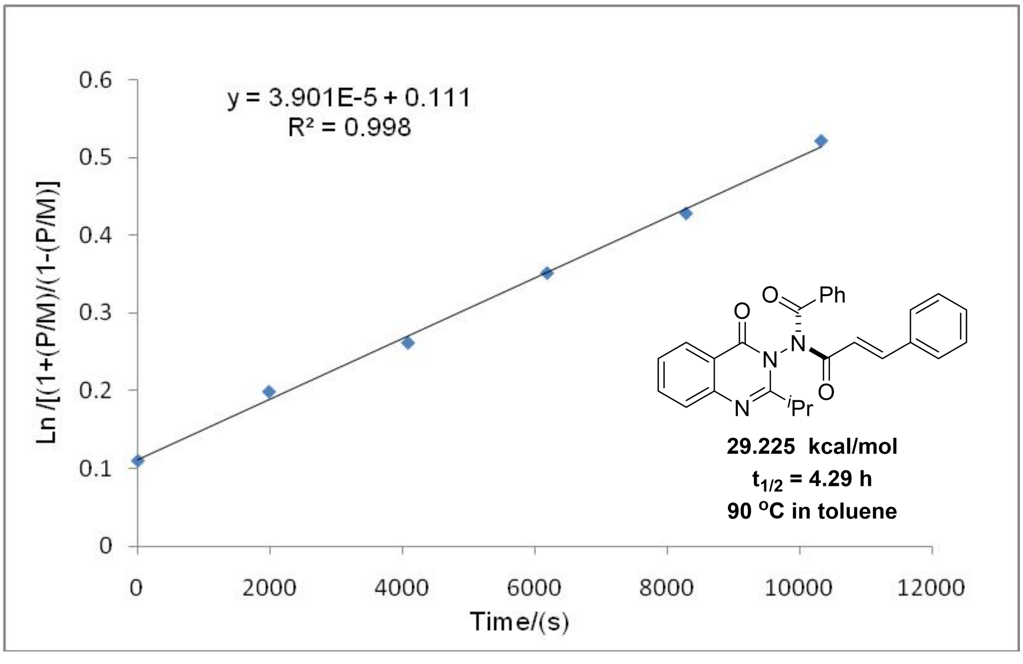

Figure S1e. rotating barrier and half-life of **3sa.**

Table S1f. Measured data of rotation barrier and half-life of **3oa.**

P/M	(1+(P/M))/(1-(P/M))	t/(s)	ln
0.044	1.092	0	0.088
0.053	1.111	1680	0.105
0.057	1.121	3360	0.114
0.062	1.131	5040	0.123
0.073	1.157	6720	0.146
0.076	1.166	8400	0.153

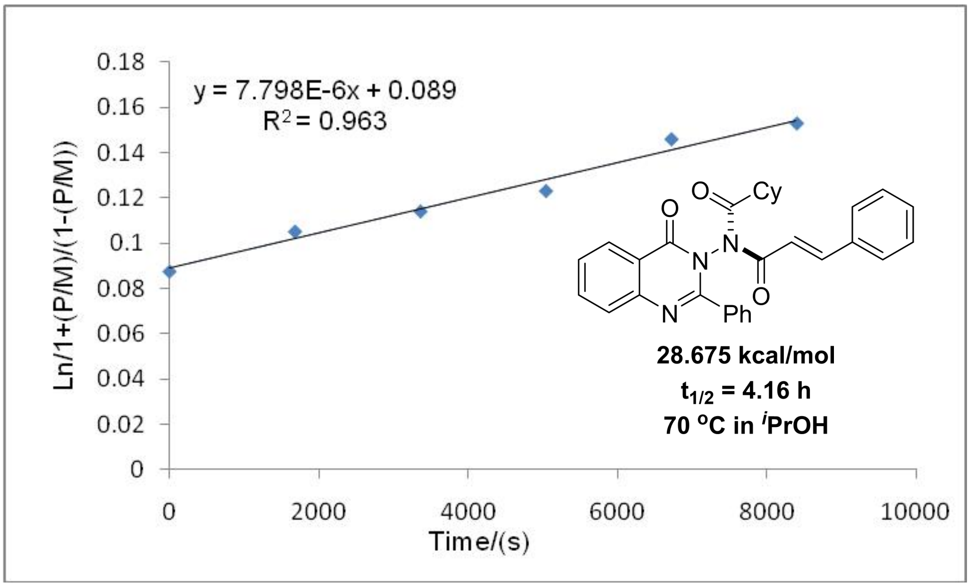

Figure S1f. rotating barrier and half-life of **3oa.**

Table S1g. Measured data of rotation barrier and half-life of **3na.**

P/M	(1+(P/M))/(1-(P/M))	t/(s)	ln
0.030	1.062	0	0.060
0.071	1.152	780	0.142
0.117	1.266	1560	0.236
0.161	1.385	2340	0.326
0.203	1.511	3120	0.412

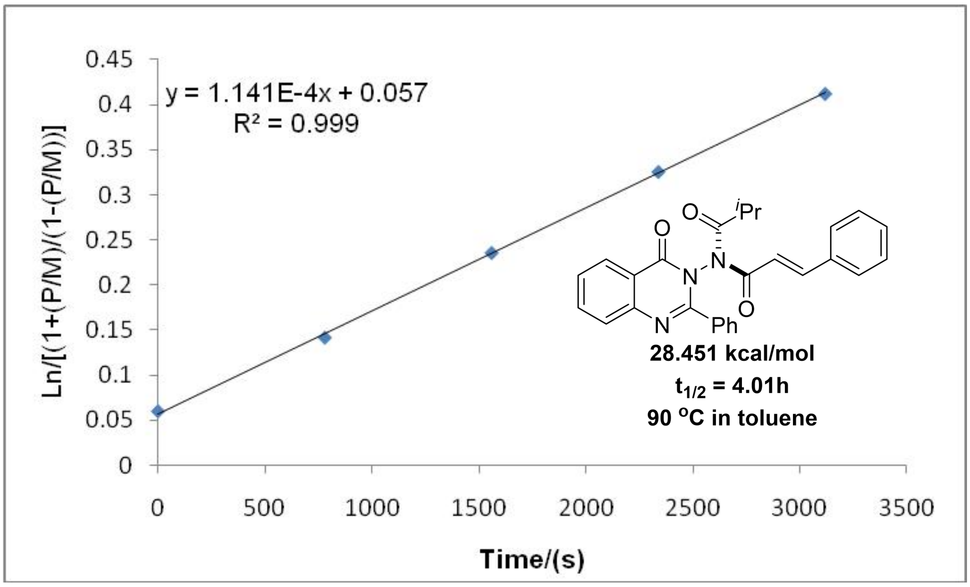

Figure S1g. rotating barrier and half-life of **3na.**

Table S1h. Measured data of rotation barrier and half-life of **3ka.**

P/M	(1+(P/M))/(1-(P/M))	t/(s)	ln
0.065	1.139	0	0.130
0.229	1.592	2160	0.465
0.335	2.008	3660	0.697
0.451	2.646	5220	0.973
0.541	3.356	6840	1.211
0.550	3.448	8520	1.238

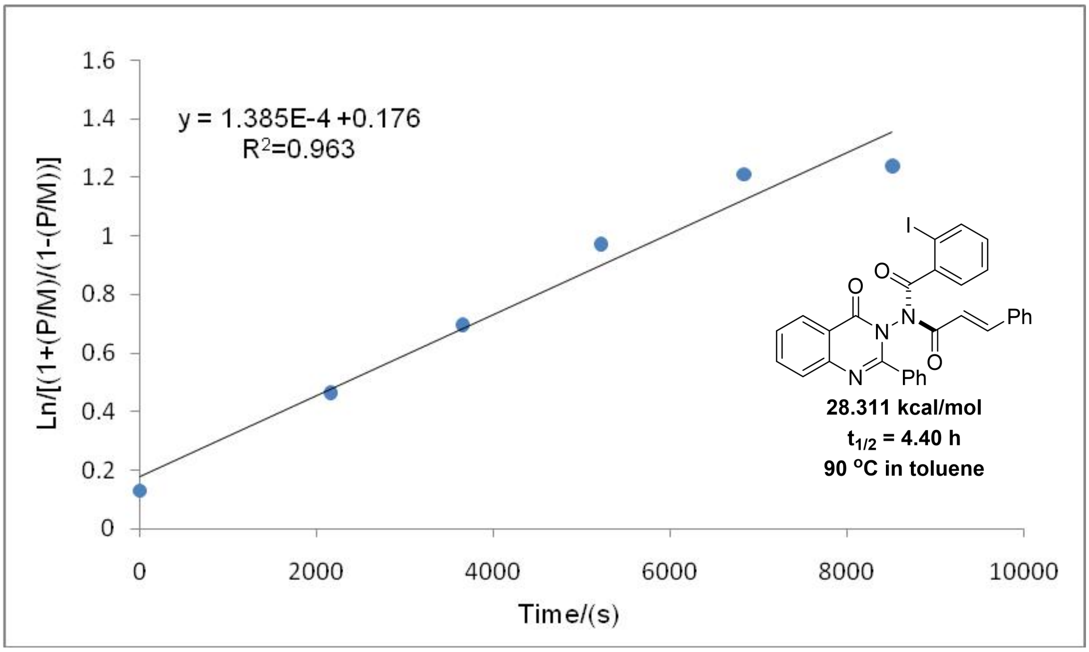

Figure S1h. rotating barrier and half-life of **3ka**

Table S1i. Measured data of rotation barrier and half-life of **3la.**

P/M	(1+(P/M))/(1-(P/M))	t/(s)	ln
0.143	1.333	0	0.288
0.1471	1.344	1260	0.296
0.154	1.363	2580	0.310
0.159	1.377	3900	0.320
0.161	1.385	5280	0.326
0.166	1.397	6540	0.334

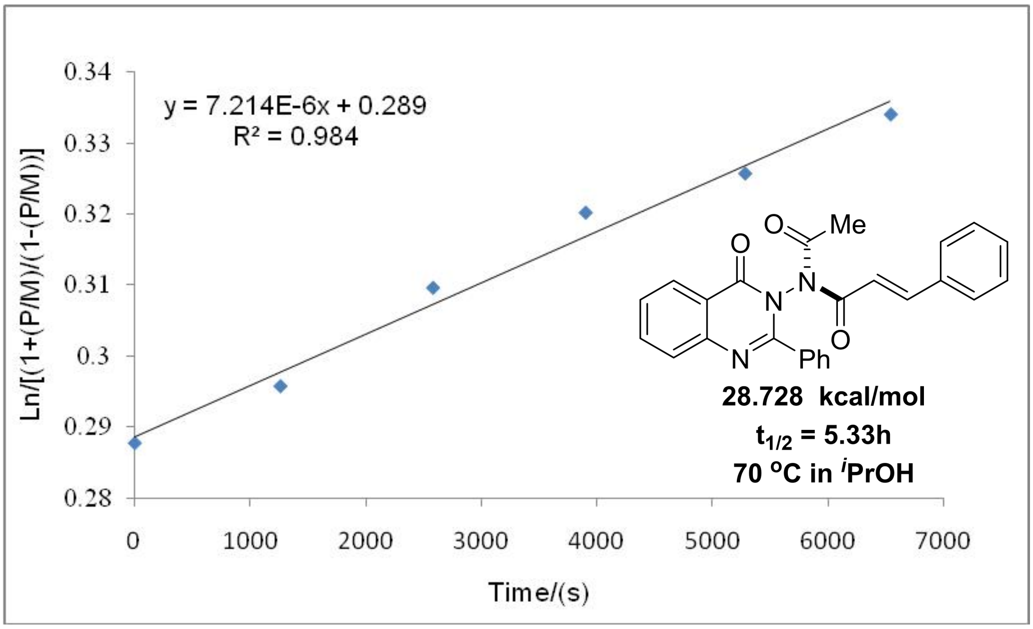

Figure S1i. rotating barrier and half-life of **3la**

6. Calculation of the Rotation Barrier of 3aa and 3sa

All density functional theory (DFT) calculations were performed using Gaussian 16.[3,4] The geometries and frequency calculations were performed using the M06-2X[5] density functional in conjunction with the 6-31G(d) basis set. The SMD[6] solvation model was used to account for the solvation effects of 2-propanol, which was used for measuring the rotation barriers. Frequency calculations confirmed that optimized structures are minima (no imaginary frequency) or transition structures (one imaginary frequency). Thermal free energy corrections were obtained at 343.15 K to match measuring conditions. Low frequencies (< 100 cm^{-1}) were corrected in the vibrational component of the entropy using a free rotor approximation according to the method of Grimme *et al.*, since entropy associated with these loose vibrational modes was the most prone to computational error.[7,8,9] The quasi-harmonic oscillator corrections were obtained using the GoodVibes.[10] To obtain more accurate electronic energies, single-point energy calculations were performed at the M06-2X/def2-TZVPP-SMD(2-propanol) level of theory with the optimized structures.[11,12]

Calculated Cartesian coordinates
3aa

Standard orientation:

Center Number	Atomic Number	Atomic Type	Coordinates (Angstroms) X	Y	Z
1	6	0	2.304221	5.250398	-0.449470
2	6	0	2.230523	4.263487	0.516515
3	6	0	1.291266	3.226745	0.390120
4	6	0	0.438122	3.215907	-0.726758
5	6	0	0.517695	4.220048	-1.701774
6	6	0	1.446675	5.234026	-1.562451
7	1	0	3.032291	6.049221	-0.345415
8	1	0	2.883909	4.266923	1.383213
9	6	0	-0.514791	2.123308	-0.881241
10	1	0	-0.155192	4.182448	-2.552681

11	1	0	1.514980	6.017254	-2.310370
12	6	0	0.375421	1.307165	1.278497
13	7	0	1.232913	2.260903	1.385173
14	7	0	-0.468299	1.188005	0.171090
15	8	0	-1.290423	1.964389	-1.807698
16	6	0	0.312955	0.262038	2.333955
17	6	0	1.511914	-0.331513	2.739541
18	6	0	-0.890817	-0.100872	2.946005
19	6	0	1.504605	-1.295676	3.742270
20	1	0	2.440562	-0.041994	2.256372
21	6	0	-0.889454	-1.057971	3.955803
22	1	0	-1.818502	0.381982	2.653169
23	6	0	0.304383	-1.660244	4.350023
24	1	0	2.435840	-1.762847	4.047344
25	1	0	-1.823391	-1.330985	4.437127
26	1	0	0.299251	-2.412041	5.133633
27	7	0	-1.281304	0.083508	0.011333
28	6	0	-2.669998	0.325445	0.143993
29	8	0	-3.046965	1.289693	0.776177
30	6	0	-3.595965	-0.578062	-0.584015
31	6	0	-4.846954	-0.826254	-0.016004
32	6	0	-3.278141	-1.079794	-1.849467
33	6	0	-5.770815	-1.608800	-0.700529
34	1	0	-5.081884	-0.413615	0.960582
35	6	0	-4.213263	-1.845126	-2.536540
36	1	0	-2.317380	-0.850324	-2.302664
37	6	0	-5.454012	-2.117175	-1.959347
38	1	0	-6.738268	-1.817722	-0.254786
39	1	0	-3.975795	-2.227530	-3.524258
40	1	0	-6.178559	-2.722122	-2.496333
41	6	0	-0.676476	-1.189809	-0.152850
42	8	0	-1.301106	-2.187882	0.143760
43	6	0	0.702971	-1.171230	-0.666461
44	6	0	1.448961	-2.280772	-0.538327
45	1	0	1.073351	-0.264799	-1.133905
46	1	0	0.994398	-3.137864	-0.041939
47	6	0	2.829112	-2.464734	-0.988775
48	6	0	3.435878	-3.710968	-0.779316
49	6	0	3.566053	-1.447614	-1.616822
50	6	0	4.745431	-3.941336	-1.189186
51	1	0	2.869868	-4.500660	-0.291993
52	6	0	4.872861	-1.678717	-2.024548
53	1	0	3.118684	-0.472186	-1.784655
54	6	0	5.465894	-2.925472	-1.812821

55	1	0	5.202426	-4.911800	-1.021452
56	1	0	5.434112	-0.885465	-2.508900
57	1	0	6.488537	-3.100942	-2.133287

3sa

Standard orientation:

Center Number	Atomic Number	Atomic Type	Coordinates (Angstroms) X	Y	Z
1	6	0	-1.197929	4.817075	0.091572
2	6	0	-1.372374	3.801064	1.013414
3	6	0	-1.100693	2.471148	0.651453
4	6	0	-0.651309	2.202193	-0.651585
5	6	0	-0.476439	3.236741	-1.582230
6	6	0	-0.749150	4.539474	-1.211019
7	1	0	-1.409999	5.842825	0.377775
8	1	0	-1.717402	4.002558	2.022586
9	6	0	-0.383185	0.823779	-1.035016
10	1	0	-0.129648	2.993878	-2.581662
11	1	0	-0.617667	5.348127	-1.922472
12	6	0	-1.044919	0.249959	1.284034
13	7	0	-1.279883	1.473944	1.598857
14	7	0	-0.623504	-0.106476	-0.004914
15	8	0	0.010668	0.441594	-2.124954
16	7	0	-0.458767	-1.439429	-0.340273
17	6	0	-1.617263	-2.254339	-0.455812
18	8	0	-1.534384	-3.456393	-0.355766
19	6	0	-2.927473	-1.552788	-0.613479
20	6	0	-3.137447	-0.576968	-1.591672
21	6	0	-3.977762	-1.966910	0.209415
22	6	0	-4.399225	-0.008498	-1.732730
23	1	0	-2.330596	-0.288168	-2.259173
24	6	0	-5.231954	-1.378533	0.076971
25	1	0	-3.803461	-2.740185	0.952684
26	6	0	-5.441796	-0.399933	-0.893123
27	1	0	-4.568297	0.740343	-2.500127
28	1	0	-6.045107	-1.688155	0.725957
29	1	0	-6.421961	0.054908	-1.000225
30	6	0	0.847517	-1.901221	-0.675708
31	8	0	0.978353	-2.944314	-1.279281
32	6	0	1.970344	-1.043867	-0.246152

33	6	0	3.213979	-1.393971	-0.610998
34	1	0	1.771741	-0.165645	0.356896
35	1	0	3.327589	-2.285017	-1.227337
36	6	0	4.454390	-0.694842	-0.269172
37	6	0	4.487435	0.434672	0.564227
38	6	0	5.658148	-1.180718	-0.798164
39	6	0	5.693687	1.058888	0.852470
40	1	0	3.568588	0.825241	0.991634
41	6	0	6.866366	-0.553607	-0.509666
42	1	0	5.638221	-2.056486	-1.441878
43	6	0	6.886157	0.567627	0.316386
44	1	0	5.707484	1.931321	1.498775
45	1	0	7.790341	-0.940685	-0.928229
46	1	0	7.827027	1.059415	0.544993
47	6	0	-1.159322	-0.830449	2.341013
48	1	0	-1.482596	-1.768862	1.884668
49	6	0	0.221242	-1.063904	2.973293
50	1	0	0.949520	-1.445702	2.251187
51	1	0	0.607088	-0.131047	3.398098
52	1	0	0.130481	-1.799174	3.778427
53	6	0	-2.189732	-0.443639	3.398754
54	1	0	-2.295743	-1.266410	4.111877
55	1	0	-1.878111	0.450929	3.943887
56	1	0	-3.167261	-0.247985	2.947138

Ts-3aa

Standard orientation:

Center Number	Atomic Number	Atomic Type	Coordinates (Angstroms) X	Y	Z
1	6	0	4.274957	4.348328	0.611947
2	6	0	4.360556	2.973197	0.488605
3	6	0	3.253220	2.247106	0.020464
4	6	0	2.078757	2.933294	-0.318818
5	6	0	2.006301	4.328672	-0.215469
6	6	0	3.099911	5.032069	0.255887
7	1	0	5.130258	4.908072	0.978307
8	1	0	5.270388	2.435510	0.736551
9	6	0	0.911739	2.167540	-0.749231
10	1	0	1.084912	4.832190	-0.490925
11	1	0	3.052295	6.111892	0.352103

12	6	0	2.315415	0.201818	-0.494033
13	7	0	3.368171	0.885922	-0.196416
14	7	0	1.038759	0.778066	-0.508113
15	8	0	-0.114853	2.643399	-1.199410
16	7	0	-0.190139	0.075755	-0.618140
17	6	0	-0.265126	-1.371822	-0.341903
18	8	0	-0.972759	-2.023737	-1.067613
19	6	0	0.394596	-1.925424	0.865052
20	6	0	0.847724	-1.118237	1.914571
21	6	0	0.553563	-3.314875	0.918050
22	6	0	1.473246	-1.708563	3.007889
23	1	0	0.709995	-0.041128	1.888687
24	6	0	1.187777	-3.894708	2.008640
25	1	0	0.199590	-3.922623	0.090840
26	6	0	1.652575	-3.090747	3.050527
27	1	0	1.822941	-1.087711	3.826545
28	1	0	1.326220	-4.970659	2.046138
29	1	0	2.152803	-3.544119	3.901078
30	6	0	-1.252180	0.710688	0.165306
31	8	0	-0.996012	1.481971	1.062418
32	6	0	-2.598733	0.324208	-0.272146
33	6	0	-3.655556	0.741981	0.445390
34	1	0	-2.686242	-0.267661	-1.174963
35	1	0	-3.457010	1.328391	1.341975
36	6	0	-5.067201	0.497978	0.147678
37	6	0	-6.028950	0.996195	1.037929
38	6	0	-5.495478	-0.207008	-0.988900
39	6	0	-7.385712	0.794596	0.804977
40	1	0	-5.702323	1.545128	1.917518
41	6	0	-6.849711	-0.407289	-1.220079
42	1	0	-4.769782	-0.597571	-1.696041
43	6	0	-7.798287	0.091789	-0.324572
44	1	0	-8.118887	1.186252	1.503407
45	1	0	-7.170060	-0.953362	-2.102184
46	1	0	-8.856307	-0.067290	-0.510695
47	6	0	2.528106	-1.192697	-0.956021
48	6	0	3.437844	-2.001447	-0.266145
49	6	0	1.909460	-1.662595	-2.116885
50	6	0	3.686926	-3.292770	-0.709774
51	1	0	3.920594	-1.616232	0.627013
52	6	0	2.166704	-2.958902	-2.559060
53	1	0	1.227625	-1.022552	-2.671564
54	6	0	3.044225	-3.776607	-1.852090
55	1	0	4.376669	-3.927998	-0.162405

56	1	0	1.682250	-3.324979	-3.458850
57	1	0	3.235510	-4.789437	-2.193727

Ts-3sa

Standard orientation:

Center Number	Atomic Number	Atomic Type	Coordinates (Angstroms) X	Y	Z
1	6	0	-5.173481	-2.912305	1.247367
2	6	0	-4.955233	-1.582532	0.933441
3	6	0	-3.780096	-1.210781	0.260257
4	6	0	-2.848617	-2.199212	-0.084197
5	6	0	-3.086318	-3.547521	0.213115
6	6	0	-4.243361	-3.900856	0.883792
7	1	0	-6.080633	-3.197402	1.771845
8	1	0	-5.678130	-0.812917	1.185436
9	6	0	-1.597513	-1.794673	-0.721520
10	1	0	-2.347677	-4.290105	-0.072124
11	1	0	-4.433782	-4.940222	1.130938
12	6	0	-2.469429	0.479214	-0.625605
13	7	0	-3.603305	0.099543	-0.145588
14	7	0	-1.371089	-0.396989	-0.658117
15	8	0	-0.772486	-2.553019	-1.196134
16	7	0	-0.018714	-0.044427	-0.888278
17	6	0	0.379850	1.377944	-0.918663
18	8	0	1.005994	1.762391	-1.872209
19	6	0	0.081735	2.213028	0.270437
20	6	0	-0.406225	1.672440	1.466021
21	6	0	0.296020	3.592572	0.154220
22	6	0	-0.668615	2.515845	2.542081
23	1	0	-0.575520	0.605146	1.572491
24	6	0	0.023989	4.427159	1.229010
25	1	0	0.666413	3.995177	-0.783455
26	6	0	-0.458718	3.888193	2.423452
27	1	0	-1.040643	2.098465	3.472082
28	1	0	0.185282	5.496620	1.138701
29	1	0	-0.672402	4.541853	3.263821
30	6	0	0.900863	-0.767972	-0.039244
31	8	0	0.514003	-1.454678	0.882846
32	6	0	2.309364	-0.595561	-0.427744
33	6	0	3.266082	-1.048037	0.400071

34	1	0	2.526174	-0.120403	-1.377317
35	1	0	2.948236	-1.508808	1.334781
36	6	0	4.711970	-0.989822	0.181362
37	6	0	5.555443	-1.493486	1.181561
38	6	0	5.285367	-0.453689	-0.983031
39	6	0	6.937945	-1.462676	1.028103
40	1	0	5.116031	-1.911076	2.083843
41	6	0	6.665083	-0.423658	-1.134875
42	1	0	4.652244	-0.060967	-1.773140
43	6	0	7.495113	-0.927246	-0.130718
44	1	0	7.578403	-1.856369	1.811445
45	1	0	7.098238	-0.007165	-2.039198
46	1	0	8.573626	-0.901667	-0.255049
47	6	0	-2.480915	1.802615	-1.378971
48	1	0	-1.485160	2.098265	-1.697135
49	6	0	-3.285920	1.572389	-2.668744
50	1	0	-3.271304	2.485233	-3.272310
51	1	0	-2.857661	0.760190	-3.265079
52	1	0	-4.324185	1.323187	-2.432117
53	6	0	-3.094791	2.924169	-0.542662
54	1	0	-3.061024	3.860657	-1.107602
55	1	0	-4.139545	2.694625	-0.315518
56	1	0	-2.562850	3.069984	0.402616

----

7. Characterization Data

($R_{a,cis,cis}$)-*N*-cinnamoyl-*N*-(4-oxo-2-phenylquinazolin-3(4*H*)-yl)benzamide (3aa)

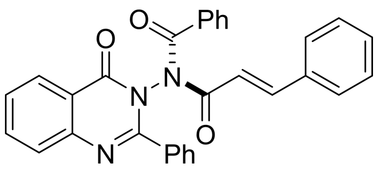

Eluent for flash column chromatography: petroleum ether/ethyl acetate = 8/1. White solid, 42.4 mg, 90% yield. ^{1}H NMR (400 MHz, Chloroform-*d*) δ 8.36 (d, *J* = 7.9 Hz, 1H), 7.88-7.83 (m, 2H), 7.67 (d, *J* = 6.6 Hz, 2H), 7.60-7.55 (m, 2H), 7.47-7.41 (m, 4H), 7.31-7.28 (m, 5H), 7.26 (d, *J* = 3.5 Hz, 1H), 7.24 (d, *J* = 7.3 Hz, 1H), 7.09 (d, *J* = 7.5 Hz, 2H), 6.19 (d, *J* = 15.5 Hz, 1H). ^{13}C NMR (101 MHz, Chloroform-d) δ 169.0, 166.1, 159.5, 155.5, 147.0, 146.3, 135.4, 133.8, 133.7, 133.3, 133.2, 131.0, 130.5, 128.8, 128.7, 128.7, 128.6, 128.3, 128.2, 128.0, 127.7, 127.5, 121.3, 118.5. HRMS (ESI): exact mass calculated for $[M+H]^+$ ($C_{30}H_{22}N_3O_3$) requires m/z 472.1656, found m/z 472.1656. The enantiomeric excess was determined to be 95% by HPLC. [OD-H column, 210 nm, *n*-hexane:IPA = 9:1, 1.0 mL/min]: 23.2 min (minor), 26.8 min (major). $[\alpha]^{22}_D$ = 107.8 (c = 1.00, $CHCl_3$).

($R_{a,cis,cis}$)-*N*-(5-chloro-4-oxo-2-phenylquinazolin-3(4*H*)-yl)-*N*-cinnamoylbenzamide (3ba)

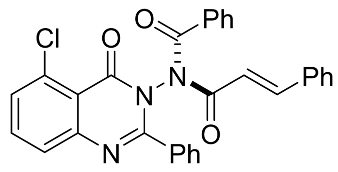

Eluent for flash column chromatography: petroleum ether/ethyl acetate = 8/1. White solid, 50.6 mg, 99% yield. ^{1}H NMR (400 MHz, Chloroform-*d*) δ 7.67-7.59 (m, 4H), 7.48-7.44 (m, 2H), 7.41-7.33 (m, 4H), 7.24-7.21 (m, 5H), 7.16 (t, *J* = 7.5 Hz, 2H), 6.96 (d, *J* = 7.5 Hz, 2H), 6.04 (d, *J* = 15.5 Hz, 1H). ^{13}C NMR (101 MHz, Chloroform-*d*) δ 168.8, 166.1, 157.3, 156.4, 149.4, 148.1, 146.1, 135.0, 134.7, 133.8, 133.7, 133.2, 133.0, 131.0, 130.7, 130.2, 128.8, 128.7, 128.6, 128.2, 127.9, 127.5, 118.8, 118.7. HRMS (ESI): exact mass calculated for $[M+H]^+$ ($C_{30}H_{21}ClN_3O_3$) requires m/z 506.1266, found m/z 506.1262. The enantiomeric excess was determined to be 96% by HPLC. [OD-H column, 210 nm, *n*-hexane:IPA = 5:1, 1.0 mL/min]: 15.4 min (minor), 18.8 min (major). $[\alpha]^{22}_D$ = 131.2 (c = 1.00, $CHCl_3$).

($R_{a,cis,cis}$)-*N*-cinnamoyl-*N*-(5-methyl-4-oxo-2-phenylquinazolin-3(4*H*)-yl)benzamide (3ca)

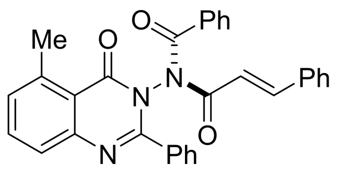

Eluent for flash column chromatography: petroleum ether/ethyl acetate = 8/1. White solid, 27.6 mg, 57% yield. ^{1}H NMR (400 MHz, Chloroform-*d*) δ 7.68-7.66 (m, 4H), 7.58 (d, *J* = 15.5 Hz, 1H), 7.46-7.40 (m, 4H), 7.32-7.29 (m, 5H), 7.24 (d, *J* = 8.0 Hz, 2H), 7.08 (d,

J = 7.5 Hz, 2H), 6.19 (d, *J* = 15.5 Hz, 1H), 2.91 (s, 3H). ^{13}C NMR (101 MHz, Chloroform-*d*) δ 169.0, 166.3, 159.6, 155.2, 148.5, 146.1, 142.1, 134.4, 133.9, 133.8, 133.5, 133.1, 130.9, 130.4, 130.2, 128.8, 128.8, 128.7, 128.6, 128.2, 127.9, 126.4, 119.8, 118.7, 22.9. HRMS (ESI): exact mass calculated for $[M+H]^+$ ($C_{31}H_{24}N_3O_3$) requires m/z 486.1812, found m/z 486.1807. The enantiomeric excess was determined to be 97% by HPLC. [OD-H column, 210 nm, *n*-hexane:IPA = 15:1, 1.0 mL/min]: 24.1 min (minor), 25.6 min (major). $[\alpha]^{22}_D$ = 103.8 (c = 1.00, CHCl$_3$).

($R_{a,cis,cis}$)-*N*-cinnamoyl-*N*-(6-methoxy-4-oxo-2-phenylquinazolin-3(4*H*)-yl)benzamide (3da)

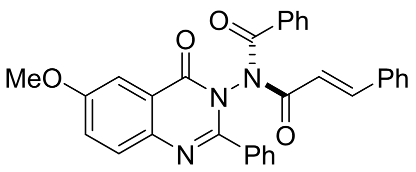

Eluent for flash column chromatography: petroleum ether/ethyl acetate = 5/1. White solid, 46.6 mg, 93% yield. ^{1}H NMR (400 MHz, Chloroform-*d*) δ 7.79 (d, *J* = 8.9 Hz, 1H), 7.75 (d, *J* = 3.0 Hz, 1H), 7.69-7.66 (m, 2H), 7.61 (d, *J* = 15.5 Hz, 1H), 7.48-7.44 (m, 5H), 7.34 (d, *J* = 7.2 Hz, 4H), 7.31-7.28 (m, 2H), 7.13 (d, *J* = 7.2 Hz, 2H), 6.24 (d, *J* = 15.5 Hz, 2H), 3.95 (s, 3H). ^{13}C NMR (101 MHz, Chloroform-*d*) δ 169.1, 166.1, 159.5, 158.9, 153.1, 146.3, 141.6, 133.8, 133.3, 133.1, 131.0, 130.4, 129.8, 128.8, 128.7, 128.7, 128.6, 128.3, 128.1, 125.4, 122.2, 118.4, 107.0, 55.9. HRMS (ESI): exact mass calculated for $[M+H]^+$ ($C_{31}H_{24}N_3O_4$) requires m/z 502.1761, found m/z 502.1759. The enantiomeric excess was determined to be 93% by HPLC. [AD-H column, 210 nm, *n*-hexane:IPA = 2:3, 1.0 mL/min]: 31.2 min (minor), 47.0 min (major). $[\alpha]^{22}_D$ = 75.6 (c =1.00, CHCl$_3$).

($R_{a,cis,cis}$)-*N*-cinnamoyl-*N*-(6-iodo-4-oxo-2-phenylquinazolin-3(4*H*)-yl)benzamide (3ea)

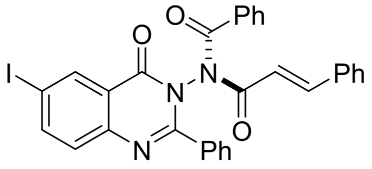

Eluent for flash column chromatography: petroleum ether/ethyl acetate = 7/1. White solid, 56.4 mg, 94% yield. ^{1}H NMR (400 MHz, Chloroform-*d*) δ 8.61 (d, *J* = 2.1 Hz, 1H), 8.04 (dd, *J* = 8.6, 2.1 Hz, 1H), 7.60-7.57 (m, 2H), 7.53-7.48 (m, 2H), 7.41-7.34 (m, 4H), 7.27-7.24 (m, 5H), 7.20-7.16 (m, 2H), 7.01 (d, *J* = 7.1 Hz, 2H)., 6.09 (d, *J* = 15.5 Hz, 1H). ^{13}C NMR (101 MHz, Chloroform-*d*) δ 168.8, 165.9, 158.1, 156.1, 146.3, 144.1, 136.3, 133.7, 133.6, 133.2, 133.1, 131.0, 130.7, 129.9, 128.8, 128.8, 128.7, 128.6, 128.3, 127.9, 122.9, 118.4, 92.1. HRMS (ESI): exact mass calculated for $[M+H]^+$ ($C_{30}H_{21}IN_3O_3$) requires m/z 598.0622, found m/z 598.0621. The enantiomeric excess was determined to be 93% by HPLC. [OD-H column, 210 nm, *n*-hexane:IPA = 5:1, 1.0 mL/min]: 17.6 min (minor), 13.9 min (major). $[\alpha]^{22}_D$ = 64.7 (c = 1.00, CHCl$_3$).

($R_{a,cis,cis}$)-*N*-(7-bromo-4-oxo-2-phenylquinazolin-3(4*H*)-yl)-*N*-cinnamoylben

zamide (3fa)

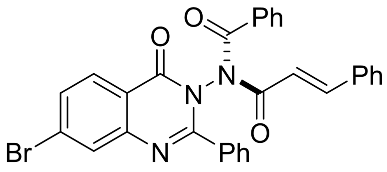

Eluent for flash column chromatography: petroleum ether/ethyl acetate = 5/1. White solid, 46.0 mg, 84% yield. ^{1}H NMR (400 MHz, Chloroform-*d*) δ 8.20 (d, *J* = 8.5 Hz, 1H), 8.02 (d, *J* = 1.9 Hz, 1H), 7.69-7.64 (m, 3H), 7.58 (d, *J* = 15.5 Hz, 1H), 7.48-7.41 (m, 4H), 7.32 (t, *J* = 4.1 Hz, 5H), 7.27 (d, *J* = 3.9 Hz, 1H), 7.25 (d, *J* = 7.5 Hz, 1H), 7.08 (d, *J* = 7.1 Hz, 2H), 6.15 (d, *J* = 15.5 Hz, 1H). ^{13}C NMR (101 MHz, Chloroform-*d*) δ 168.8, 166.0, 159.0, 158.8, 156.7, 147.9, 146.3, 133.7, 133.6, 133.3, 133.0, 131.0, 130.7, 130.2, 128.9, 128.9, 128.8, 128.7, 128.6, 128.4, 128.2, 127.9, 120.2, 118.4. HRMS (ESI): exact mass calculated for $[M+H]^+$ ($C_{30}H_{21}BrN_3O_3$) requires m/z 550.0761, found m/z 550.0759. The enantiomeric excess was determined to be 95% by HPLC. [OD-H column, 210 nm, *n*-hexane:IPA = 15:1, 1.0 mL/min]: 35.5 min (minor), 39.7 min (major). $[\alpha]^{22}_D$ = 40.8 (c = 1.00, $CHCl_3$).

($R_{a,cis,cis}$)-*N*-(8-chloro-4-oxo-2-phenylquinazolin-3(4*H*)-yl)-*N*-cinnamoylben zamide (3ga)

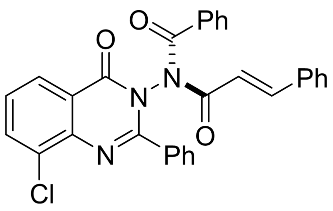

Eluent for flash column chromatography: petroleum ether/ethyl acetate = 7/1. White solid, 44.4 mg, 88% yield. ^{1}H NMR (400 MHz, Chloroform-*d*) δ 8.18 (d, *J* = 8.0 Hz, 1H), 7.83 (d, *J* = 7.9 Hz, 1H). 7.63 (d, *J* = 6.5 Hz, 1H), 7.50 (d, *J* = 15.5 Hz, 2H), 7.40-7.32 (m, 5H), 7.26-7.14 (m, 7H), 6.99 (d, *J* = 7.6 Hz, 2H), 6.08 (d, *J* = 15.5 Hz, 1H). ^{13}C NMR (101 MHz, Chloroform-*d*) δ 168.8, 166.0, 159.0, 156.0, 146.4, 143.8, 135.7, 133.7, 133.6, 133.3, 133.0, 132.8, 131.0, 130.7, 128.8, 128.8, 128.7, 128.6, 128.3, 128.3, 127.7, 126.3, 123.0, 118.4. HRMS (ESI): exact mass calculated for $[M+H]^+$ ($C_{30}H_{21}ClN_3O_3$) requires m/z 506.1266, found m/z 506.1263. The enantiomeric excess was determined to be 93% by HPLC. [OD-H column, 210 nm, *n*-hexane:IPA = 15:1, 1.0 mL/min]: 34.2 min (minor), 38.0 min (major). $[\alpha]^{22}_D$ = 133.5 (c = 1.00, $CHCl_3$).

($R_{a,cis,cis}$)-4-chloro-*N*-cinnamoyl-*N*-(4-oxo-2-phenylquinazolin-3(4*H*)-yl)ben zamide (3ha)

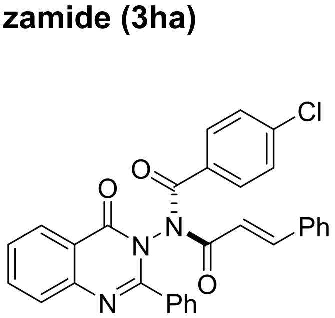

Eluent for flash column chromatography: petroleum ether/ethyl acetate = 8/1. White solid, 37.6 mg, 74% yield. ^{1}H NMR (400 MHz, Chloroform-*d*) δ 8.34 (d, *J* = 8.5 Hz, 1H), 7.85-7.81 (m, 2H), 7.64-7.60 (m, 3H), 7.58-7.54 (m, 1H), 7.46-7.38 (m, 3H), 7.33 (d, *J* = 7.2 Hz, 1H), 7.29 (d, *J* = 7.7 Hz, 2H), 7.25 (d, *J* = 4.8 Hz, 4H), 7.17 (d, *J* = 7.3 Hz, 2H), 6.28 (d, *J* = 15.5 Hz, 1H). ^{13}C NMR (101 MHz, Chloroform-*d*) δ 168.2, 165.9, 159.6, 155.2, 147.2, 146.9, 139.5, 135.5, 133.7,

133.2, 132.1, 131.2, 130.6, 130.0, 129.0, 128.9, 128.7, 128.4, 128.3, 128.0, 127.8, 127.6, 121.2, 117.6. HRMS (ESI): exact mass calculated for $[M+H]^+$ ($C_{30}H_{21}ClN_3O_3$) requires m/z 506.1266, found m/z 506.1267. The enantiomeric excess was determined to be 92% by HPLC. [AD-H column, 210 nm, *n*-hexane:IPA = 3:2, 1.0 mL/min]: 23.6 min (minor), 44.1 min (major). $[\alpha]^{22}_D$ = 68.6 (c = 1.00, $CHCl_3$).

(*R*$_{a,cis,cis}$)-*N*-cinnamoyl-4-methyl-*N*-(4-oxo-2-phenylquinazolin-3(4*H*)-yl)benzamide (3ia)

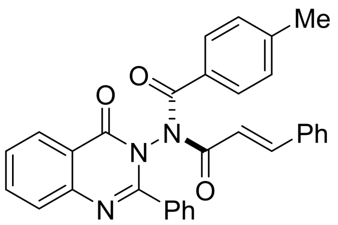

Eluent for flash column chromatography: petroleum ether/ethyl acetate = 7/1.White solid, 29.6 mg, 61% yield. ^{1}H NMR (400 MHz, Chloroform-*d*) δ 8.36 (d, *J* = 7.5 Hz, 1H), 7.85-7.82 (m, 2H), 7.68-7.65 (m, 2H), 7.60-7.54 (m, 2H), 7.48-7.39 (m, 3H), 7.33-7.30 (m, 1H), 7.28 (s, 1H), 7.26-7.20 (m, 3H), 7.10 (dd, *J* = 7.7, 1.8 Hz, 4H), 6.20 (d, *J* = 15.5 Hz, 1H), 2.30 (s, 3H). ^{13}C NMR (101 MHz, Chloroform-*d*) δ 168.9, 166.2, 159.5, 155.5, 147.0, 145.9, 144.2, 135.3, 134.0, 133.4, 130.9, 130.8, 130.4, 129.3, 128.9, 128.8, 128.6, 128.2, 128.2, 128.0, 127.6, 127.5, 121.4, 118.7, 21.6. HRMS (ESI): exact mass calculated for $[M+H]^+$ ($C_{31}H_{24}N_3O_3$) requires m/z 486.1812, found m/z 486.1810. The enantiomeric excess was determined to be 95% by HPLC. [OD-H column, 210 nm, *n*-hexane:IPA = 15:1, 1.0 mL/min]: 38.6 min (minor), 41.8 min (major). $[\alpha]^{22}_D$ = 59.7 (c = 1.00, $CHCl_3$).

(*R*$_{a,cis,cis}$)-*N*-cinnamoyl-3-methoxy-*N*-(4-oxo-2-phenylquinazolin-3(4*H*)-yl)benzamide (3ja)

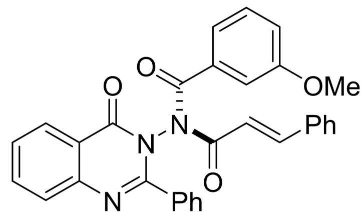

Eluent for flash column chromatography: petroleum ether/ethyl acetate = 5/1. White solid, 48.0 mg, 96% yield. ^{1}H NMR (400 MHz, Chloroform-*d*) δ 8.37 (d, *J* = 7.9 Hz, 1H), 7.87-7.85 (m, 2H), 7.70-7.68 (m, 2H), 7.63 -7.56 (m, 2H), 7.49-7.43 (m, 3H), 7.36-7.32 (m, 1H), 7.28 (t, *J* = 7.4 Hz, 2H), 7.21 (t, *J* = 8.0 Hz, 1H), 7.14 (d, *J* = 7.1 Hz, 2H), 6.99 (dd, *J* = 8.4, 2.6 Hz, 1H), 6.87 (d, *J* = 9.9 Hz, 2H), 6.27 (d, *J* = 15.5 Hz, 1H), 3.70 (s, 3H). ^{13}C NMR (101 MHz, Chloroform-*d*) δ 168.8, 166.2, 159.7, 159.4, 155.4, 147.0, 146.1, 135.4, 134.9, 133.9, 133.3, 130.9, 130.5, 129.9, 128.8, 128.6, 128.3, 128.2, 128.0, 127.6, 127.5, 121.3, 121.1, 119.8, 118.5, 112.9, 55.5. HRMS (ESI): exact mass calculated for $[M+H]^+$ ($C_{31}H_{24}N_3O_4$) requires m/z 502.1761, found m/z 502.1756. The enantiomeric excess was determined to be 94% by HPLC. [IC column, 210 nm, *n*-hexane:IPA = 4:1, 1.0 mL/min]: 69.3 min (minor), 45.9 min (major). $[\alpha]^{22}_D$ = 138.1 (c = 1.00, $CHCl_3$).

(*R*$_{a,cis,cis}$)-*N*-cinnamoyl-2-iodo-*N*-(4-oxo-2-phenylquinazolin-3(4*H*)-yl)benza

mide (3ka)

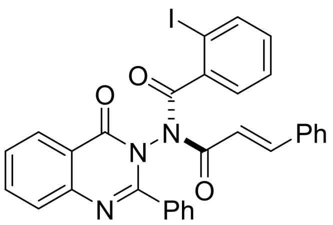

Eluent for flash column chromatography: petroleum ether/ethyl acetate = 8/1. White solid, 27.2 mg, 46% yield. ^1H NMR (400 MHz, Chloroform-*d*) δ 8.38 (d, *J* = 8.0 Hz, 1H), 7.87-7.80 (m, 4H), 7.72 (d, *J* = 7.1 Hz, 2H), 7.58 (dd, *J* = 8.1, 4.0 Hz, 1H), 7.49-7.45 (m, 3H), 7.41-7.32 (m, 3H), 7.29-7.26 (m, 2H), 7.14 (d, *J* = 7.5 Hz, 2H), 7.05-7.00 (m, 1H), 6.10 (s, 1H). ^{13}C NMR (101 MHz, Chloroform-*d*) δ 167.9, 165.5, 159.5, 155.4, 147.8, 147.1, 140.0, 135.4, 133.6, 132.3, 131.1, 130.6, 129.5, 129.0, 128.9, 128.7, 128.6, 128.5, 128.4, 128.3, 127.7, 127.5, 121.4, 117.8, 100.0, 93.0. HRMS (ESI): exact mass calculated for $[M+H]^+$ ($C_{30}H_{21}IN_3O_3$) requires m/z 598.0622, found m/z 598.0621. The enantiomeric excess was determined to be 91% by HPLC. [OD-H column, 210 nm, *n*-hexane:IPA = 5:1, 1.0 mL/min]: 23.1 min (minor), 32.8 min (major). $[\alpha]^{22}_D$ = -60 (c = 1.00, $CHCl_3$).

($R_{a,cis,cis}$)-*N*-acetyl-*N*-(4-oxo-2-phenylquinazolin-3(4*H*)-yl)cinnamamide (3la)

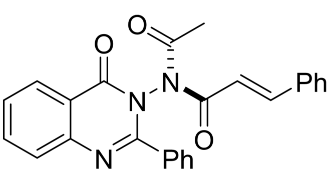

Eluent for flash column chromatography: petroleum ether/ethyl acetate = 8/1. Colorless oil, 40.8 mg, 99% yield. ^1H NMR (400 MHz, Chloroform-*d*) δ 8.36 (d, *J* = 7.2 Hz, 1H), 7.88-7.84 (m, 3H), 7.61-7.57 (m, 1H), 7.52-7.46 (m, 5H), 7.44-7.42 (m, 2H), 7.37 (dd, *J* = 6.2, 4.2 Hz, 3H), 6.86 (d, *J* = 15.5 Hz, 1H), 2.38 (s, 3H). ^{13}C NMR (101 MHz, Chloroform-*d*) δ 170.2, 165.8, 159.8, 155.5, 148.6, 146.9, 135.7, 133.9, 132.8, 131.2, 130.7, 129.0, 128.7, 128.6, 128.3, 127.9, 127.8, 127.7, 121.0, 116.4, 25.1. HRMS (ESI): exact mass calculated for $[M+H]^+$ ($C_{25}H_{20}N_3O_3$) requires m/z 410.1499, found m/z 410.1498. The enantiomeric excess was determined to be 85% by HPLC. [OD-H column, 210 nm, *n*-hexane:IPA = 5:1, 1.0 mL/min]: 11.5 min (minor), 17.1 min (major). $[\alpha]^{22}_D$ = -36.4 (c = 1.00, $CHCl_3$).

($R_{a,cis,cis}$)-*N*-(4-oxo-2-phenylquinazolin-3(4*H*)-yl)-*N*-(2-phenylacetyl)cinnamamide (3ma)

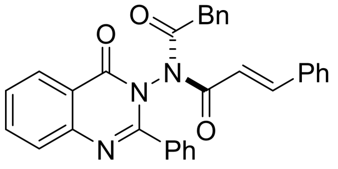

Eluent for flash column chromatography: petroleum ether/ethyl acetate = 6/1. Colorless oil, 45.6 mg, 94% yield. ^1H NMR (400 MHz, Chloroform-*d*) δ 8.36 (d, *J* = 7.8 Hz, 1H), 7.93-7.86 (m, 3H), 7.60-7.56 (m, 2.2 Hz, 1H), 7.49-7.46 (m, 5H), 7.41-7.33 (m, 6H), 7.27-7.23 (m, 2H), 7.02-6.99 (m, 2H), 6.96-6.88 (m, 1H), 4.04 (dd, *J* = 8.4 Hz, 2H). ^{13}C NMR (101 MHz, Chloroform-*d*) δ 171.3, 166.1, 160.0, 155.5, 148.9, 146.9, 135.8, 133.9, 132.8, 132.7, 131.3, 131.1, 130.7, 129.8, 129.6, 129.0, 128.7, 128.7, 128.6, 128.5, 128.3, 128.0, 127.9, 127.7, 127.2, 121.0, 116.3, 43.5.

HRMS (ESI): exact mass calculated for $[M+H]^+$ ($C_{31}H_{24}N_3O_3$) requires m/z 486.1812, found m/z 486.1812. The enantiomeric excess was determined to be 87% by HPLC. [AD-H column, 210 nm, *n*-hexane:IPA = 3:2, 1.0 mL/min]: 19.3 min (minor), 26.1 min (major). $[\alpha]^{22}_D$ = -66.1 (c = 1.00, $CHCl_3$).

(*R*$_{a,cis,cis}$)-*N*-isobutyryl-*N*-(4-oxo-2-phenylquinazolin-3(4*H*)-yl)cinnamamide (3na)

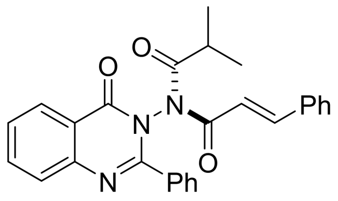

Eluent for flash column chromatography: petroleum ether/ethyl acetate = 8/1. Colorless oil, 43.6 mg, 99% yield. ^{1}H NMR (400 MHz, Chloroform-*d*) δ 8.35 (d, *J* = 7.3 Hz, 1H), 8.94-7.84 (m, 3H), 7.60-7.56 (m, 1H), 7.50-7.46 (m, 5H), 7.43-7.35 (m, 5H), 6.77 (d, *J* = 15.4 Hz, 1H), 3.45-3.41 (m, 1H), 1.13 (d, *J* = 6.6 Hz, 3H), 0.77 (d, *J* = 6.8 Hz, 3H). ^{13}C NMR (101 MHz, Chloroform-*d*) δ 177.2, 166.1, 160.1, 155.8, 148.7, 147.0, 135.7, 133.9, 132.8, 131.2, 130.7, 129.0, 128.7, 128.5, 128.3, 128.1, 127.8, 127.7, 121.0, 116.2, 35.3, 18.9, 18.6. HRMS (ESI): exact mass calculated for $[M+H]^+$ ($C_{27}H_{24}N_3O_3$) requires m/z 438.1812, found m/z 438.1810. The enantiomeric excess was determined to be 95% by HPLC. [OD-H column, 210 nm, *n*-hexane:IPA = 9:1, 1.0 mL/min]: 11.1 min (minor), 8.7 min (major). $[\alpha]^{22}_D$ = -66.1 (c = 1.00, $CHCl_3$).

(*R*$_{a,cis,cis}$)-*N*-cinnamoyl-*N*-(4-oxo-2-phenylquinazolin-3(4*H*)-yl)cyclohexane carboxamide (3oa)

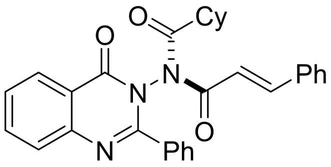

Eluent for flash column chromatography: petroleum ether/ethyl acetate = 10/1. Colorless oil, 31.6 mg, 66% yield. ^{1}H NMR (400 MHz, Chloroform-*d*) δ 8.35 (d, *J* = 8.0 Hz, 1H), 7.93-7.84 (m, 3H), 7.60-7.55 (m, 1H), 7.49-7.45 (m, 5H), 7.43-7.35 (m, 5H), 6.72 (d, *J* = 15.3 Hz, 1H), 3.18 (d, *J* = 11.7 Hz, 1H), 1.89 (d, *J* = 13.0 Hz, 1H), 1.80-1.69 (m, 1H), 1.61 (d, *J* = 11.3 Hz, 2H), 1.47-1.36 (m, 2H), 1.23-1.11 (m, 3H), 0.94-0.86 (m, 1H). ^{13}C NMR (101 MHz, Chloroform-*d*) δ 175.9, 166.2, 160.1, 155.8, 148.7, 147.0, 141.9, 135.6, 133.9, 132.8, 131.2, 130.6, 129.0, 128.7, 128.5, 128.1, 127.7, 121.0, 116.2, 108.9, 44.9, 29.1, 28.6, 25.7, 25.6, 25.3. HRMS (ESI): exact mass calculated for $[M+H]^+$ ($C_{30}H_{28}N_3O_3$) requires m/z 478.2125, found m/z 478.2124. The enantiomeric excess was determined to be 91% by HPLC. [AD-H column, 210 nm, *n*-hexane:IPA = 4:1, 1.0 mL/min]: 14.4 min (minor), 21.7 min (major). $[\alpha]^{22}_D$ = -44.7 (c = 1.00, $CHCl_3$).

(*R*$_{a,cis,cis}$)-*N*-(2-(4-bromophenyl)-4-oxoquinazolin-3(4*H*)-yl)-*N*-cinnamoylbe nzamide (3pa)

Eluent for flash column chromatography: petroleum ether/ethyl acetate = 4/1.

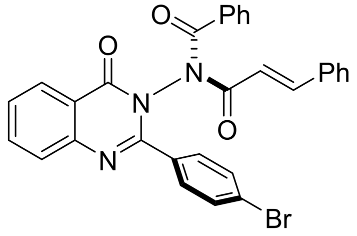

White solid, 42.8 mg, 78% yield. ^{1}H NMR (400 MHz, Chloroform-*d*) δ 8.35 (d, *J* = 7.3 Hz, 1H), 7.81-7.81 (m, 2H), 7.60-7.54 (m, 6H), 7.50-7.46 (m, 1H), 7.36 (m, 5H), 7.26 (d, *J* = 3.2 Hz, 1H), 7.08 (d, *J* = 7.2 Hz, 2H), 6.18 (d, *J* = 15.5 Hz, 1H). ^{13}C NMR (101 MHz, Chloroform-*d*) δ 169.0, 166.1, 159.4, 154.5, 146.9, 146.5, 135.5, 133.7, 133.6, 133.4, 132.2, 131.8, 131.1, 129.7, 128.9, 128.8, 128.3, 128.2, 127.9, 127.6, 125.2, 121.3, 118.4. HRMS (ESI): exact mass calculated for [M+Na]$^+$ ($C_{30}H_{20}BrN_3NaO_3$) requires m/z 572.0580, found m/z 572.0579. The enantiomeric excess was determined to be 95% by HPLC. [OD-H column, 210 nm, *n*-hexane:IPA = 4:1, 1.0 mL/min]: 14.9 min (minor), 17.9 min (major). $[\alpha]^{22}_D$ = 142.6 (c = 1.00, $CHCl_3$).

(*R*$_{a,cis,cis}$)-*N*-cinnamoyl-*N*-(2-(4-methoxyphenyl)-4-oxoquinazolin-3(4*H*)-yl) benzamide (3qa)

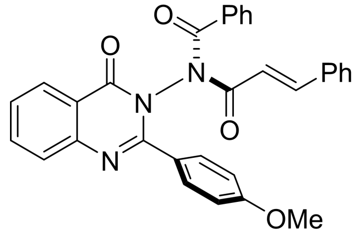

Eluent for flash column chromatography: petroleum ether/ethyl acetate = 5/1. White solid, 47.8 mg, 95% yield. ^{1}H NMR (400 MHz, Chloroform-*d*) δ 8.34 (d, *J* = 7.4 Hz, 1H), 7.84-7.81 (m, 2H), 7.65-7.61 (m, 2H), 7.57-7.53 (m, 2H), 7.49-7.41 (m, 4H), 7.35-7.31 (m, 3H), 7.29-7.25 (m, 1H), 7.14-7.12 (m, 2H), 6.94 (d, *J* = 8.8 Hz, 2H), 6.28 (d, *J* = 15.5 Hz, 1H), 3.80 (s, 3H). ^{13}C NMR (101 MHz, Chloroform-*d*) δ 169.1, 166.2, 161.4, 159.7, 147.1, 146.3, 135.3, 133.9, 133.8, 133.1, 130.9, 129.9, 128.8, 128.8, 128.7, 128.3, 128.1, 127.5, 127.4, 125.5, 121.1, 118.5, 114.0, 55.4. HRMS (ESI): exact mass calculated for [M+H]$^+$ ($C_{31}H_{24}N_3O_4$) requires m/z 502.1761, found m/z 502.1759. The enantiomeric excess was determined to be 95% by HPLC. [OD-H column, 210 nm, *n*-hexane:IPA = 5:1, 1.0 mL/min]: 26.2 min (minor), 33.0 min (major). $[\alpha]^{22}_D$ = 38.7 (c = 1.00, $CHCl_3$).

(*R*$_{a,cis,cis}$)-*N*-cinnamoyl-*N*-(4-oxo-2-(m-tolyl)quinazolin-3(4*H*)-yl)benzamide (3ra)

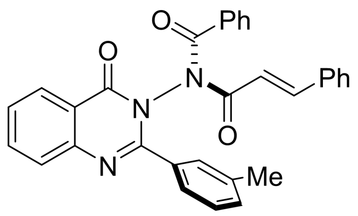

Eluent for flash column chromatography: petroleum ether/ethyl acetate = 8/1. White solid, 30.0 mg, 62% yield. ^{1}H NMR (400 MHz, Chloroform-*d*) δ 8.37 (d, *J* = 8.0 Hz, 1H), 7.86-7.84 (m, 2H), 7.61-7.55 (m, 2H), 7.50-7.45 (m, 2H), 7.43 (d, *J* = 1.8 Hz, 1H), 7.35-7.31 (m, 6H), 7.28-7.26 (m, 2H), 7.25 (d, *J* = 1.4 Hz, 1H), 7.12-7.10 (m, 2H), 6.23 (d, *J* = 15.5 Hz, 1H), 2.31 (s, 3H). ^{13}C NMR (101 MHz, Chloroform-*d*) δ 169.0, 166.1, 159.5, 155.7, 147.0, 146.2, 138.6, 135.4, 133.9, 133.8, 133.1, 131.2, 131.0, 128.8, 128.7, 128.7, 128.6, 128.5, 128.2, 128.2, 127.6, 127.5, 124.9, 121.3, 118.5, 21.3. HRMS (ESI): exact mass calculated for [M+H]$^+$ ($C_{31}H_{24}N_3O_3$) requires m/z 486.1812,

found m/z 486.1810. The enantiomeric excess was determined to be 94% by HPLC. [OD-H column, 210 nm, *n*-hexane:IPA = 9:1, 1.0 mL/min]: 22.7 min (minor), 25.9 min (major). $[\alpha]^{22}_D$ = 42.7 (c = 1.00, $CHCl_3$).

($R_{a,cis,cis}$)-*N*-cinnamoyl-*N*-(2-isopropyl-4-oxoquinazolin-3(4*H*)-yl)benzamide (3sa)

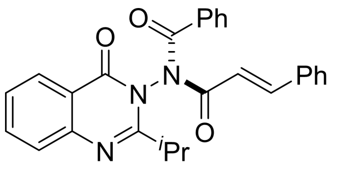

Eluent for flash column chromatography: petroleum ether/ethyl acetate = 10/1. White solid, 37.2 mg, 85% yield. ^1H NMR (400 MHz, Chloroform-*d*) δ 8.25 (d, *J* = 8.0, 1.4 Hz, 1H), 7.89 (d, *J* = 7.2 Hz, 2H), 7.79-7.77 (m, 1H), 7.75-7.70 (m, 2H), 7.56-7.52 (m, 1H), 7.48-7.43 (m, 3H), 7.36-7.32 (m, 1H), 7.30-7.22 (m, 4H), 6.49 (d, *J* = 15.5 Hz, 1H), 3.09 (q, *J* = 6.7 Hz, 1H), 1.34 (dd, *J* = 6.7, 2.6 Hz, 6H). ^{13}C NMR (101 MHz, Chloroform-*d*) δ 169.8, 166.4, 161.7, 159.9, 147.3, 147.2, 135.1, 133.9, 133.8, 133.2, 131.1, 129.0, 128.9, 128.8, 128.4, 127.7, 127.3, 126.8, 120.9, 117.7, 31.2, 21.6, 21.3. HRMS (ESI): exact mass calculated for $[M+H]^+$ ($C_{27}H_{24}N_3O_3$) requires m/z 438.1812, found m/z 438.1813. The enantiomeric excess was determined to be 89% by HPLC. [AD-H column, 210 nm, *n*-hexane:IPA = 4:1, 1.0 mL/min]: 14.0 min (minor), 28.6 min (major). $[\alpha]^{22}_D$ = -117.7 (c = 1.00, $CHCl_3$).

($R_{a,cis,cis}$)-(*E*)-*N*-(2-(1-(4-chlorobenzoyl)-5-methoxy-2-methyl-1H-indol-3-yl)acetyl)-3-(4-methoxyphenyl)-*N*-(4-oxo-2-phenylquinazolin-3(4*H*)-yl)acrylamide (3ta)

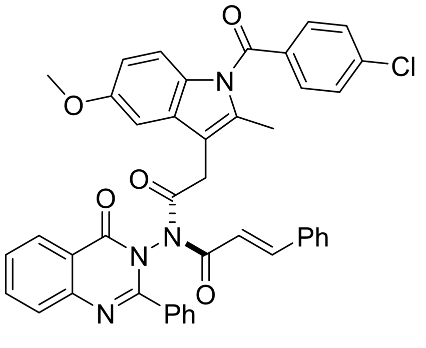

Eluent for flash column chromatography: petroleum ether/ethyl acetate = 8/1. White solid, 27.2 mg, 39% yield. ^1H NMR (400 MHz, Chloroform-*d*) δ 8.33 (d, *J* = 7.9 Hz, 1H), 7.98 (d, *J* = 15.4 Hz, 1H), 7.88-7.84 (m, 2H), 7.64 (d, *J* = 8.2 Hz, 2H), 7.60-7.56 (m, 1H), 7.54-7.50 (m, 4H), 7.48-7.44 (m, 4H), 7.42-7.35 (m, 5H), 6.83 (d, *J* = 8.9 Hz, 1H), 6.73 (d, *J* = 2.5 Hz, 1H), 6.62 (d, *J* = 8.9, 1H), 4.11 (s, 2H), 3.83 (s, 3H), 2.16 (s, 3H). ^{13}C NMR (101 MHz, Chloroform-*d*) δ 169.3, 167.2, 165.4, 158.9, 154.9, 154.3, 148.1, 145.8, 138.1, 135.4, 134.7, 132.8, 132.8, 131.6, 130.3, 130.1, 129.8, 129.6, 129.5, 128.0, 127.9, 127.7, 127.6, 127.3, 127.0, 126.9, 126.6, 119.8, 113.8, 110.7, 100.0, 54.6, 28.7, 12.3. HRMS (ESI): exact mass calculated for $[M+Na]^+$ ($C_{42}H_{31}ClN_4O_5Na$) requires m/z 729.1875, found m/z 729.1880.The enantiomeric excess was determined to be 90% by HPLC. [IC column, 210 nm, *n*-hexane:IPA = 4:1, 1.0 mL/min]: 12.8 min (minor), 11.0 min (major). $[\alpha]^{22}_D$ = 1.1 (c = 1.00, $CHCl_3$).

($R_{a,cis,cis}$)-(*S*)-*N*-(2-(6-methoxynaphthalen-2-yl)propanoyl)-*N*-(4-oxo-2-phen

ylquinazolin-3(4*H*)-yl)cinnamamide (3ua)

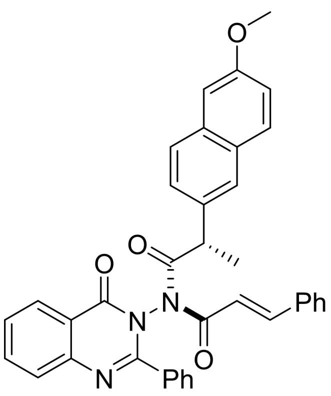

Eluent for flash column chromatography: petroleum ether/ethyl acetate = 8/1. White solid, 27.2 mg, 47% yield. ^{1}H NMR (400 MHz, Chloroform-*d*) δ 8.21 (d, *J* = 7.7 Hz, 1H), 7.92 (d, *J* = 15.4 Hz, 1H), 7.86-7.82 (m, 2H), 7.53 (d, *J* = 8.5 Hz, 2H), 7.49 (d, *J* = 9.0 Hz, 1H), 7.45-7.42 (m, 2H), 7.38-7.33 (m, 5H), 7.28 (s, 1H), 7.15-7.05 (m, 5H), 7.01 (d, *J* = 7.4 Hz, 1H), 6.77 (d, *J* = 15.7 Hz, 1H), 3.93 (s, 3H), 2.97 (d, *J* = 23.8 Hz, 1H), 1.54 (d, *J* = 6.9 Hz, 3H). ^{13}C NMR (101 MHz, Chloroform-*d*) δ 174.1, 166.2, 157.6, 155.6, 148.9, 146.9, 135.6, 134.2, 133.9, 133.6, 132.2, 131.3, 130.5, 129.5, 129.3, 129.0, 128.8, 128.7, 128.3, 128.2, 128.1, 127.9, 127.7, 127.6, 127.2, 126.5, 126.3, 120.9, 118.7, 105.4, 55.4, 46.3, 20.1. HRMS (ESI): exact mass calculated for $[M+Na]^+$ ($C_{37}H_{29}N_3O_4Na$) requires m/z 602.2050, found m/z 602.2055. The diastereoisomeric excess was determined to be 83% by HPLC. [IA column, 210 nm, *n*-hexane:IPA = 4:1, 1.0 mL/min]: 16.4 min (minor), 25.0 min (major). $[\alpha]^{22}_D$ = -23.6 (c = 1.00, $CHCl_3$).

($R_{a,cis,cis}$)-(*E*)-*N*-(4-oxo-2-phenylquinazolin-3(4*H*)-yl)-*N*-(3-(4-(trifluorometh yl)phenyl)acryloyl)benzamide (3ab)

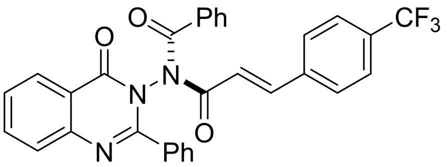

Eluent for flash column chromatography: petroleum ether/ethyl acetate = 6/1. White solid, 52.0 mg, 96% yield. ^{1}H NMR (400 MHz, Chloroform-*d*) δ 8.37 (d, *J* = 7.5 Hz, 1H), 7.86 (dd, *J* = 6.3, 1.6 Hz, 2H), 7.67-7.65 (m, 2H), 7.60-7.58 (m, 1H), 7.57-7.51 (m, 3H), 7.49-7.42 (m, 4H), 7.32 (d, *J* = 4.7 Hz, 4H), 7.19 (d, *J* = 8.1 Hz, 2H), 6.28 (d, *J* = 15.6 Hz, 1H). ^{13}C NMR (101 MHz, Chloroform-*d*) δ 168.8, 165.7, 157.3 (d, *J* = 417.4 Hz), 147.0, 143.8, 137.1, 135.4, 133.6, 133.3, 133.2, 132.4, 132.0, 130.6, 128.8, 128.8, 128.6, 128.2, 128.0, 127.7, 127.5, 125.8 (d, *J* = 4.3 Hz), 125.0, 122.3, 121.2 (d, *J* = 22.8 Hz). ^{19}F NMR (376 MHz, Chloroform-*d*) δ -63.0. HRMS (ESI): exact mass calculated for $[M+H]^+$ ($C_{31}H_{21}F_3N_3O_3$) requires m/z 540.1530, found m/z 540.1526. The enantiomeric excess was determined to be 93% by HPLC. [OD-H column, 210 nm, *n*-hexane:IPA = 5:1, 1.0 mL/min]: 17.4 min (minor), 23.1 min (major). $[\alpha]^{22}_D$ = 55.2 (c = 1.00, $CHCl_3$).

($R_{a,cis,cis}$)-(*E*)-*N*-(3-(3-bromophenyl)acryloyl)-*N*-(4-oxo-2-phenylquinazolin-3(4*H*)-yl)benzamide (3ac)

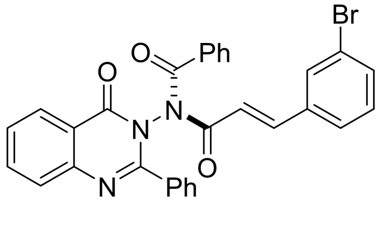

Eluent for flash column chromatography: petroleum ether/ethyl acetate = 5/1. White solid, 42.2 mg, 77% yield. ^{1}H NMR (400 MHz,

Chloroform-*d*) δ 8.36 (d, *J* = 7.9 Hz, 1H), 7.86-7.83 (m, 2H), 7.66-7.64 (m, 2H), 7.59-7.55 (m, 1H), 7.50-7.42 (m, 6H), 7.35-7.29 (m, 4H), 7.15-7.11 (m, 2H), 7.02 (d, *J* = 7.7 Hz, 1H), 6.16 (d, *J* = 15.5 Hz, 1H). ^{13}C NMR (101 MHz, Chloroform-*d*) δ 168.9, 165.7, 159.4, 155.3, 147.0, 144.1, 135.9, 135.4, 133.7, 133.6, 133.4, 133.2, 130.7, 130.6, 130.3, 128.8, 128.7, 128.6, 128.2, 128.0, 127.7, 127.5, 126.9, 122.9, 121.3, 120.1. HRMS (ESI): exact mass calculated for $[M+H]^+$ ($C_{30}H_{21}BrN_3O_3$) requires m/z 550.0761, found m/z 550.0759. The enantiomeric excess was determined to be 94% by HPLC. [OD-H column, 210 nm, *n*-hexane:IPA = 9:1, 1.0 mL/min]: 28.5 min (minor), 30.6 min (major). $[\alpha]^{22}_D$ = 68.2 (c = 1.00, $CHCl_3$).

($R_{a,cis,cis}$)-(*E*)-*N*-(3-(3-fluorophenyl)acryloyl)-*N*-(4-oxo-2-phenylquinazolin-3 (4*H*)-yl)benzamide (3ad)

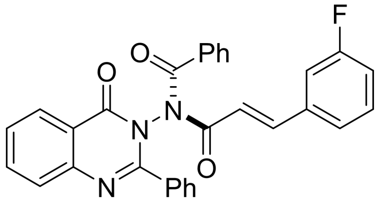

Eluent for flash column chromatography: petroleum ether/ethyl acetate = 7/1. White solid, 38.2 mg, 78% yield. ^{1}H NMR (400 MHz, Chloroform-*d*) δ 8.35 (d, *J* = 7.9 Hz, 1H), 7.85-7.84 (m, 2H), 7.66 (d, *J* = 7.0 Hz, 2H), 7.58-7.53 (m, 2H), 7.49-7.41 (m, 4H), 7.31 (d, *J* = 4.7 Hz, 4H), 7.25-7.19 (m, 1H), 7.03-6.98 (m, 1H), 6.89 (d, *J* = 7.7 Hz, 1H), 6.71 (d, *J* = 9.6 Hz, 1H), 6.18 (d, *J* = 15.5 Hz, 1H). ^{13}C NMR (101 MHz, Chloroform-*d*) δ 168.9, 165.8, 164.0, 161.5, 159.4, 155.3, 145.7 (d, *J* = 250.0 Hz), 136.0 (d, *J* = 7.6 Hz), 135.4, 133.7, 133.3, 133.2, 130.6, 130.4 (d, *J* = 8.2 Hz), 128.8, 128.7, 128.6, 128.2, 128.0, 127.7, 127.5, 124.3 (d, *J* = 3.1 Hz), 121.3, 119.9, 117.8 (d, *J* = 21.3 Hz), 114.3 (d, *J* = 21.8 Hz). ^{19}F NMR (376 MHz, Chloroform-*d*) δ -112.2. HRMS (ESI): exact mass calculated for $[M+H]^+$ ($C_{30}H_{21}FN_3O_3$) requires m/z 490.1561, found m/z 490.1558. The enantiomeric excess was determined to be 91% by HPLC. [OD-H column, 210 nm, *n*-hexane:IPA = 5:1, 1.0 mL/min]: 16.6 min (minor), 18.6 min (major). $[\alpha]^{22}_D$ = 164.0 (c = 1.00, $CHCl_3$).

($R_{a,cis,cis}$)-(*E*)-*N*-(4-oxo-2-phenylquinazolin-3(4*H*)-yl)-*N*-(3-(o-tolyl)acryloyl) benzamide (3ae)

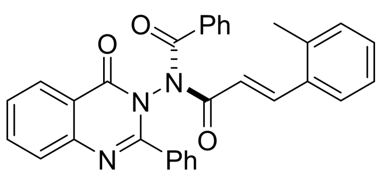

Eluent for flash column chromatography: petroleum ether/ethyl acetate = 6/1. White solid, 34.4 mg, 71% yield. ^{1}H NMR (400 MHz, Chloroform-*d*) δ 8.37 (d, *J* = 8.0 Hz, 1H), 7.90-7.85 (m, 3H), 7.68 (d, *J* = 9.3 Hz, 2H), 7.59-7.55 (m, 1H), 7.50-7.41 (m, 4H), 7.32 (d, *J* = 4.9 Hz, 4H), 7.20 (t, *J* = 7.4 Hz, 1H), 7.11 (d, *J* = 7.6 Hz, 1H), 7.01 (t, *J* = 7.6 Hz, 1H), 6.75 (d, *J* = 7.7 Hz, 1H), 6.12 (d, *J* = 15.4 Hz, 1H), 2.30 (s, 3H). ^{13}C NMR (101 MHz, Chloroform-*d*) δ 169.0, 166.2, 159.5, 155.5, 147.0, 143.9, 138.3, 135.4, 133.9, 133.3, 133.1, 132.8, 130.8, 130.7, 130.5, 128.8, 128.7, 128.6, 128.2, 128.0, 127.6, 127.5,

126.5, 126.2, 121.3, 119.5, 19.7. HRMS (ESI): exact mass calculated for $[M+H]^+$ ($C_{31}H_{24}N_3O_3$) requires m/z 486.1812, found m/z 486.1811. The enantiomeric excess was determined to be 89% by HPLC. [OD-H column, 210 nm, *n*-hexane:IPA = 5:1, 1.0 mL/min]: 15.3 min (minor), 17.0 min (major). $[\alpha]^{22}_D$ =71.5 (c = 1.00, $CHCl_3$).

($R_{a,cis,cis}$)-(*E*)-*N*-(3-(naphthalen-1-yl)acryloyl)-*N*-(4-oxo-2-phenylquinazolin-3(4*H*)-yl)benzamide (3af)

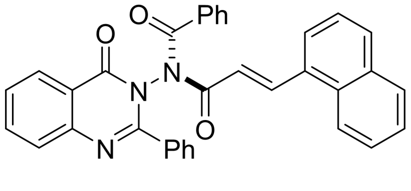

Eluent for flash column chromatography: petroleum ether/ethyl acetate = 5/1. Yellow solid, 30.2 mg, 58% yield. ^{1}H NMR (400 MHz, Chloroform-*d*) δ 8.41-8.37 (m, 2H), 7.99-7.94 (m, 2H), 7.89 (d, *J* = 3.4 Hz, 2H), 7.88-7.85 (m, 2H), 7.84-7.82 (m, 2H), 7.52-7.49 (m, 3H), 7.47-7.45 (m, 3H), 7.40-7.35 (m, 4H), 7.29 (d, *J* = 7.6 Hz, 1H), 6.94 (d, *J* = 7.3 Hz, 1H), 6.29 (d, *J* = 15.3 Hz, 1H). ^{13}C NMR (101 MHz, Chloroform-*d*) δ 169.0, 166.1, 159.5, 155.5, 147.0, 143.1, 135.4, 133.9, 133.5, 133.3, 133.2, 131.3, 130.5, 128.9, 128.8, 128.7, 128.6, 128.6, 128.2, 128.0, 127.7, 127.6, 127.1, 126.4, 125.4, 125.1, 124.0, 123.2, 121.4, 121.1. HRMS (ESI): exact mass calculated for $[M+H]^+$ ($C_{34}H_{24}N_3O_3$) requires m/z 522.1812, found m/z 522.1812. The enantiomeric excess was determined to be 96% by HPLC. [IC column, 210 nm, *n*-hexane:IPA = 3:2, 1.0 mL/min]: 32.9 min (minor), 25.5 min (major). $[\alpha]^{22}_D$ = 35.7 (c = 1.00, $CHCl_3$).

($R_{a,cis,cis}$)-(*E*)-*N*-(4-oxo-2-phenylquinazolin-3(4*H*)-yl)-*N*-(3-(thiophen-2-yl)acryloyl)benzamide (3ag)

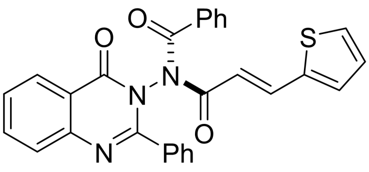

Eluent for flash column chromatography: petroleum ether/ethyl acetate = 5/1. White solid, 38.0 mg, 80% yield. ^{1}H NMR (400 MHz, Chloroform-*d*) δ 8.35 (d, *J* = 7.4 Hz, 1H), 7.85-7.82 (m, 2H), 7.70-7.67 (m, 2H), 7.66-7.65 (m, 1H), 7.58-7.55 (m, 1H), 7.47-7.42 (m, 4H), 7.32-7.23 (m, 5H), 7.08 (d, *J* = 3.7 Hz, 1H), 6.95 (dd, *J* = 5.1, 3.6 Hz, 1H), 5.96 (d, *J* = 15.1 Hz, 1H). ^{13}C NMR (101 MHz, Chloroform-*d*) δ 168.9, 165.9, 159.5, 155.5, 147.0, 139.1, 138.5, 135.4, 133.7, 133.3, 133.2, 132.3, 130.5, 129.8, 128.7, 128.7, 128.6, 128.3, 128.2, 128.0, 127.6, 127.5, 121.3, 117.0. HRMS (ESI): exact mass calculated for $[M+H]^+$ ($C_{28}H_{20}N_3O_3S$) requires m/z 478.1220, found m/z 478.1219. The enantiomeric excess was determined to be 94% by HPLC. [IA column, 210 nm, *n*-hexane:IPA = 15:1, 1.0 mL/min]: 24.7 min (minor), 13.3 min (major). $[\alpha]^{22}_D$ = 67.00 (c = 1.00, $CHCl_3$).

($R_{a,cis,cis}$)-*N*-(4-oxo-2-phenylquinazolin-3(4*H*)-yl)-*N*-(3-phenylpropanoyl)benzamide (3ah)

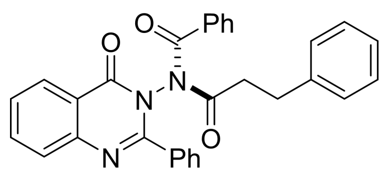

Eluent for flash column chromatography: petroleum ether/ethyl acetate = 8/1. White solid, 27.2 mg, 58% yield. ^{1}H NMR (400 MHz, Chloroform-*d*) δ 8.37 (d, *J* = 7.9 Hz, 1H), 7.88-7.80 (m, 2H), 7.59 (t, *J* = 7.5 Hz, 1H), 7.51-7.46 (m, 4H), 7.41-7.38 (m, 2H), 7.31-7.37 (m, 3H), 7.24-7.19 (m, 4H), 7.03 (d, *J* = 7.2 Hz, 2H), 2.94-2.68 (m, 3H), 2.65-2.51 (m, 1H). ^{13}C NMR (101 MHz, Chloroform-*d*) δ 172.7, 169.4, 159.8, 155.0, 146.8, 139.9, 135.5, 133.5, 133.0, 132.9, 130.6, 128.7, 128.5, 128.5, 128.4, 128.3, 128.2, 128.0, 127.8, 127.5, 126.4, 121.2, 38.8, 30.5. HRMS (ESI): exact mass calculated for $[M+H]^+$ ($C_{30}H_{24}N_3O_3$) requires m/z 474.1812, found m/z 474.1812. The enantiomeric excess was determined to be 92% by HPLC. [IC column, 210 nm, *n*-hexane:IPA = 5:1, 1.0 mL/min]: 34.2 min (minor), 36.3 min (major). $[\alpha]^{22}_{D}$ = 44.0 (c = 1.00, $CHCl_3$).

(*R*$_{a,cis,cis}$)-*N*-acetyl-*N*-(4-oxo-2-phenylquinazolin-3(4*H*)-yl)benzamide (3ai)

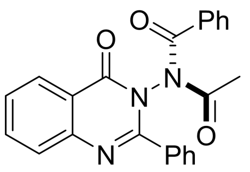

Eluent for flash column chromatography: petroleum ether/ethyl acetate = 8/1. Colorless oil, 35.4 mg, 92% yield. ^{1}H NMR (400 MHz, Chloroform-*d*) δ 8.28 (d, *J* = 7.9 Hz, 1H), 7.78-7.71 (m, 2H), 7.49-7.44 (m, 3H), 7.42-7.35 (m, 4H), 7.24-7.19 (m, 2H), 7.15 (d, *J* = 7.5 Hz, 2H), 2.00 (s, 3H). ^{13}C NMR (101 MHz, Chloroform-*d*) δ 170.3, 169.3, 159.5, 155.1, 146.9, 135.5, 133.5, 133.1, 133.0, 130.6, 128.7, 128.6, 128.3, 128.2, 127.9, 127.8, 127.5, 121.2, 25.2. HRMS (ESI): exact mass calculated for $[M+H]^+$ ($C_{23}H_{18}N_3O_3$) requires m/z 384.1343, found m/z 384.1340. The enantiomeric excess was determined to be 92% by HPLC. [OD-H column, 210 nm, *n*-hexane:IPA = 5:1, 1.0 mL/min]: 13.1 min (minor), 14.9 min (major). $[\alpha]^{22}_{D}$ = 105.6 (c = 1.00, $CHCl_3$).

(*R*$_{a,cis,cis}$)- *N*-(4-oxo-2-phenylquinazolin-3(4*H*)-yl)-*N*-propionylbenzamide (3aj)

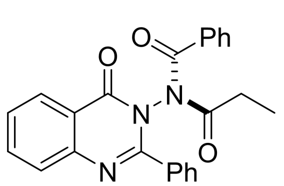

Eluent for flash column chromatography: petroleum ether/ethyl acetate = 8/1. Colorless oil, 25.6 mg, 64% yield. ^{1}H NMR (400 MHz, Chloroform-*d*) δ 8.12 (d, *J* = 8.0, 1.3 Hz, 1H), 7.64-7.56 (m, 2H), 7.36-7.32 (m, 3H), 7.31-7.26 (m, 2H), 7.24-7.20 (m, 2H), 7.11-7.07 (m, 2H), 7.05-7.03 (m, 2H), 2.14 (q, *J* = 17.2, 7.3 Hz, 1H), 1.95 (q, *J* = 17.1, 7.3 Hz, 1H), 0.76 (t, *J* = 7.3 Hz, 3H). ^{13}C NMR (101 MHz, Chloroform-*d*) δ 174.3, 169.5, 159.6, 155.2, 146.9, 135.4, 133.8, 133.2, 132.9, 130.6, 128.7, 128.6, 128.3, 128.2, 127.9, 127.7, 127.5, 121.3, 30.9, 9.1. HRMS (ESI): exact mass calculated for $[M+H]^+$ ($C_{24}H_{20}N_3O_3$) requires m/z 398.1499, found m/z 398.1495. The enantiomeric excess was determined to be 93% by HPLC. [OD-H column, 210 nm, *n*-hexane:IPA = 4:1, 1.0 mL/min]: 9.1 min (minor), 12.8

min (major). [α]$^{22}_{D}$ = 97.3 (c = 1.00, $CHCl_3$).

(*R*$_a$)-*N*-benzoyl-*N*-(4-oxo-2-phenylquinazolin-3(4*H*)-yl)-4-phenyl-4,5-dihydro-3*H*-3λ^3,4λ^3-pyrazole-3-carboxamide (4)

Eluent for flash column chromatography: petroleum ether/ethyl acetate = 4/1.

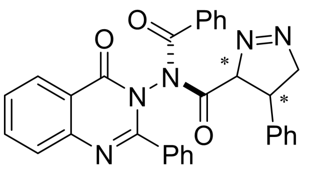

White solid, 32.4 mg, 63% yield. ^{1}H NMR (400 MHz, Chloroform-*d*) δ 8.30 (d, *J* = 2.3 Hz, 1H), 7.85-7.80 (m, 2H), 7.68 (t, *J* = 6.7 Hz, 2H), 7.57-7.51 (m, 2H), 7.38 (d, *J* = 7.7 Hz, 2H), 7.34 (d, *J* = 7.7 Hz, 4H), 7.25-7.21 (m, 2H), 7.14 (d, *J* = 8.0 Hz, 2H), 6.80 (d, *J* = 6.9 Hz, 1H), 6.52 (s, 1H), 4.08 (d, *J* = 12.3, 5.4 Hz, 2H), 3.59 (t, *J* = 11.5 Hz, 1H), 3.46 (d, *J* = 5.6 Hz, 1H). ^{13}C NMR (101 MHz, Chloroform-*d*) δ 170.1, 162.7, 160.2, 155.9, 147.0, 135.2, 133.3, 131.8, 130.0, 128.7, 128.7, 128.6, 128.4, 128.3, 128.2, 128.0, 127.5, 127.4, 127.3, 127.2, 127.1, 121.5, 57.4, 47.8, 29.7. HRMS (ESI): exact mass calculated for [M+H]$^+$ ($C_{31}H_{24}N_5O_3$) requires m/z 514.1874, found m/z 514.1865. The enantiomeric excess was determined to be 94% by HPLC. [AD-H column, 210 nm, *n*-hexane:IPA = 3:1, 1.0 mL/min]: 30.3 min (minor), 16.1 min (major), 57.6 min (minor), 25.9 min (major). [α]$^{22}_{D}$ = 7.5 (c = 1.00, $CHCl_3$).

(*R*)-(2-methylpiperidin-1-yl)(phenyl)methanone (6a)

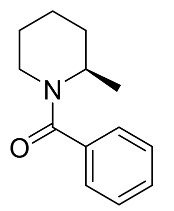

Eluent for flash column chromatography: dichloromethane/ethyl acetate = 20/1. Colorless oil, 12.7 mg, 45% yield. ^{1}H NMR (400 MHz, Chloroform-*d*) δ 7.39-7.33 (m, 5H), 4.90-3.61 (br, 2H), 2.98 (s, 1H), 1.73-1.61 (m, 4H), 1.48 (d, *J* = 20.8 Hz, 2H), 1.22 (d, *J* = 7.0 Hz, 3H). ^{13}C NMR (101 MHz, Chloroform-*d*) δ 170.5, 137.0, 129.1, 128.4, 126.3, 30.3, 26.0, 18.9, 16.2. The enantiomeric excess was determined to be 30% by HPLC. [AS-H column, 210 nm, *n*-hexane:IPA = 5:1, 1.0 mL/min]: 20.1 min (minor), 16.8 min (major). [α]$^{22}_{D}$ = -11.1 (c = 1.00, $CHCl_3$).

(*S*)-(4-methoxyphenyl)(2-methylpiperidin-1-yl)methanone (6b)

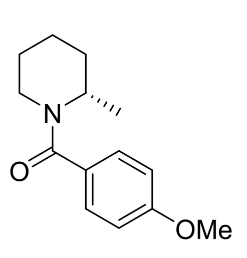

Eluent for flash column chromatography: dichloromethane/ethyl acetate = 12/1. Colorless oil, 23.6 mg, 51% yield. ^{1}H NMR (400 MHz, Chloroform-*d*) δ 7.25 (d, *J* = 8.7 Hz, 2H), 6.81 (d, *J* = 8.7 Hz, 2H), 4.21 (d, *J* = 197.1 Hz, 2H), 3.74 (s, 3H), 2.91 (t, *J* = 13.3 Hz, 1H), 1.64-1.54 (m, 4H), 1.46-1.33 (m, 2H), 1.15 (d, *J* = 7.0 Hz, 3H). ^{13}C NMR (101 MHz, Chloroform-*d*) δ 170.5, 160.3, 129.2, 128.4, 113.7, 55.3, 30.4, 26.1, 18.9, 16.1. The enantiomeric excess was determined to be 36% by HPLC. [AS-H column, 210 nm, *n*-hexane:IPA = 5:1, 1.0 mL/min]: 21.4 min (minor), 23.7 min (major). [α]$^{22}_{D}$ = 5.7 (c = 1.00, $CHCl_3$).

N-benzyl-*N*-(4-oxo-2-phenylquinazolin-3(4*H*)-yl)benzamide (8a)

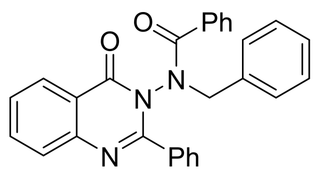

Eluent for flash column chromatography: petroleum ether/ethyl acetate = 3/1. White solid, 40.9 mg, 95% yield. ^{1}H NMR (400 MHz, $CDCl_3$) δ 8.41 (d, *J* = 7.8 Hz, 1H), 7.87-7.70 (m, 2H), 7.60-7.38 (m, 7H), 7.27-7.18 (m, 3H), 7.08 (t, *J* = 8.3 Hz, 4H), 6.69 (d, *J* = 7.3 Hz, 2H), 4.63 (q, *J* = 14.3 Hz, 2H). ^{13}C NMR (101 MHz, $CDCl_3$) δ 172.0, 159.0, 156.5, 146.9, 135.1, 134.3, 133.7, 132.7, 130.9, 129.9, 129.8, 128.9, 128.8, 128.5, 128.1, 127.9, 127.7, 127.3, 127.1, 121.6, 55.3. HRMS (ESI): exact mass calculated for $[M+H]^+$ ($C_{28}H_{22}N_3O_2$) requires m/z 432.1707, found m/z 432.1709. The enantiomeric excess was determined to be 91% by HPLC. [OD-H column, 210 nm, *n*-hexane:IPA = 9:1, 1.0 mL/min]: 13.4 min (minor), 19.7 min (major). $[\alpha]^{22}_D$ = 9.33 (c = 1.00, CH_2Cl_2).

Methyl 2-((*N*-(4-oxo-2-phenylquinazolin-3(4*H*)-yl)benzamido)methyl)acrylate (8b)

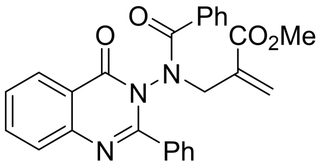

Eluent for flash column chromatography: petroleum ether/ethyl acetate = 3/1. White solid, 40.0 mg, 91% yield. ^{1}H NMR (400 MHz, Methanol-*d*$_4$) δ 8.30 (d, *J* = 8.1 Hz, 1H), 7.91 (t, *J* = 7.7 Hz, 1H), 7.77 (d, *J* = 8.2 Hz, 1H), 7.65-7.61 (m, 4H), 7.54 (t, *J* = 7.6 Hz, 3H), 7.48 (t, *J* = 7.6 Hz, 2H), 7.41 (d, *J* = 8.2 Hz, 2H), 6.09 (s, 1H), 5.42 (s, 1H), 4.36 (d, *J* = 14.3 Hz, 1H), 4.23 (d, *J* = 14.3 Hz, 1H), 3.47 (s, 3H). ^{13}C NMR (101 MHz, Methanol-*d*$_4$) δ 173.3, 166.6, 160.5, 158.1, 147.8, 136.7, 135.1, 135.0, 134.6, 133.4, 132.2, 131.9, 129.7, 129.6, 129.4, 129.0, 128.6, 128.3, 128.2, 122.5, 53.7, 52.4. HRMS (ESI): exact mass calculated for $[M+H]^+$ ($C_{26}H_{22}N_3O_4$) requires m/z 440.1605, found m/z 440.1610. The enantiomeric excess was determined to be 91% by HPLC. [OD-H column, 210 nm, *n*-hexane:IPA = 4:1, 1.0 mL/min]: 12.8 min (minor), 21.7 min (major). $[\alpha]^{22}_D$ = 16.3 (c = 1.00, $CHCl_3$).

8. NOE for Compound 3na

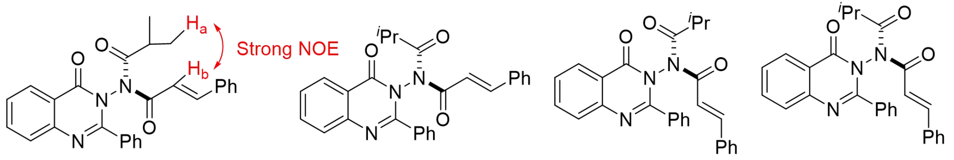

cis/cis *trans/cis* *cis/trans* *trans/trans*

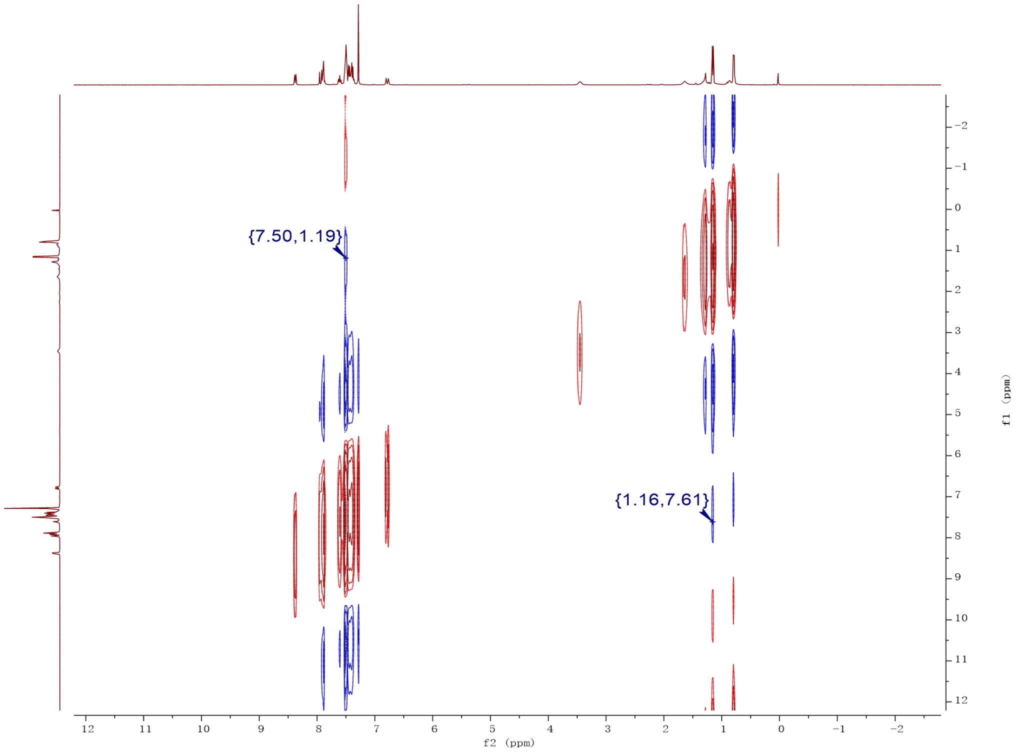

9. HPLC Analysis

($R_{a,cis,cis}$)-*N*-cinnamoyl-*N*-(4-oxo-2-phenylquinazolin-3(4*H*)-yl)benzamide (3aa)

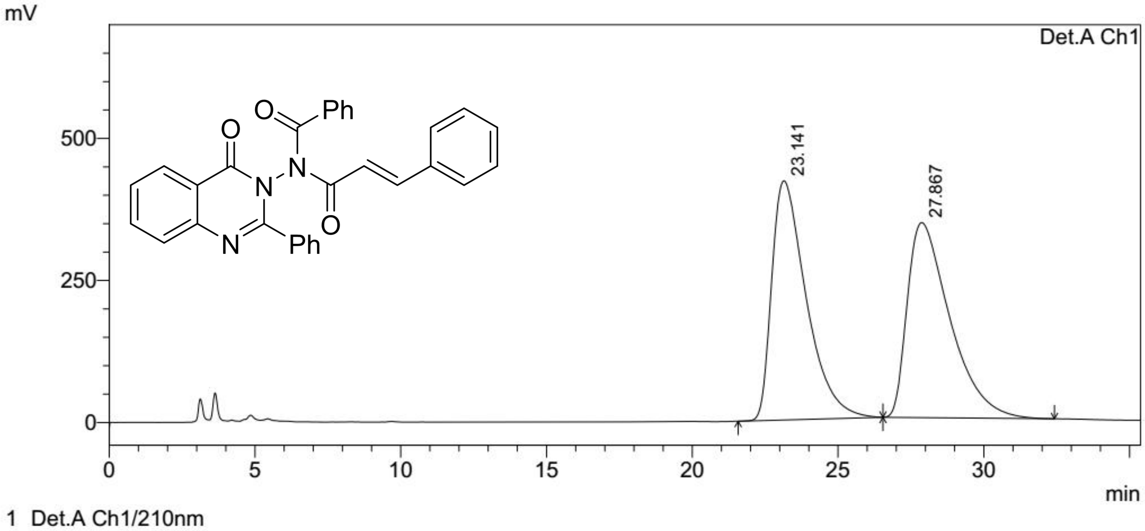

PeakTable

Detector A Ch1 210nm

Peak#	Ret. Time	Area	Height	Area %	Height %
1	23.141	34640354	420879	49.794	55.083
2	27.867	34927662	343206	50.206	44.917
Total		69568015	764085	100.000	100.000

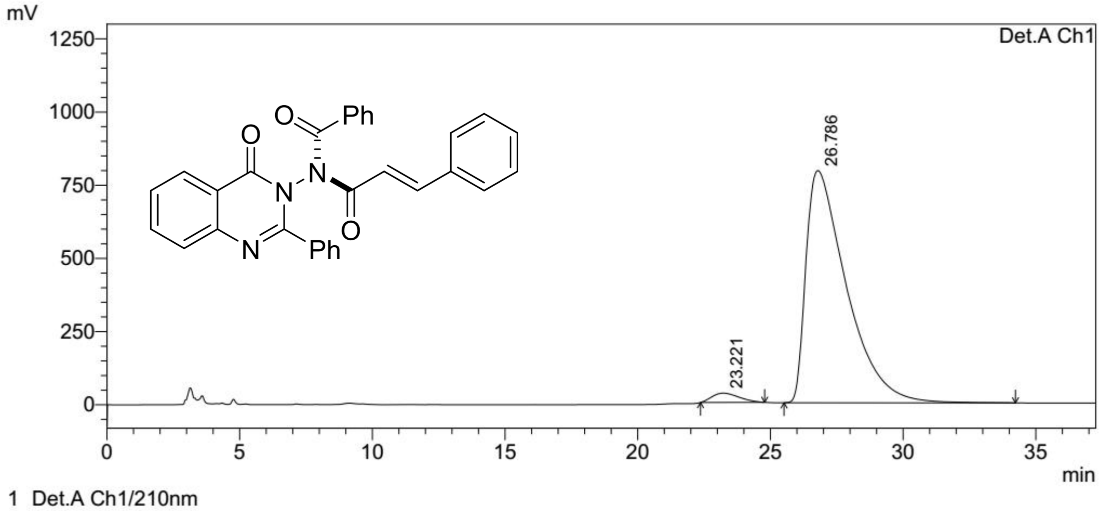

PeakTable

Detector A Ch1 210nm

Peak#	Ret. Time	Area	Height	Area %	Height %
1	23.221	2260681	31776	2.613	3.852
2	26.786	84241649	793066	97.387	96.148
Total		86502330	824842	100.000	100.000

(*R*$_{a,cis,cis}$)-*N*-(5-chloro-4-oxo-2-phenylquinazolin-3(4*H*)-yl)-*N*-cinnamoylbenzamide (3ba)

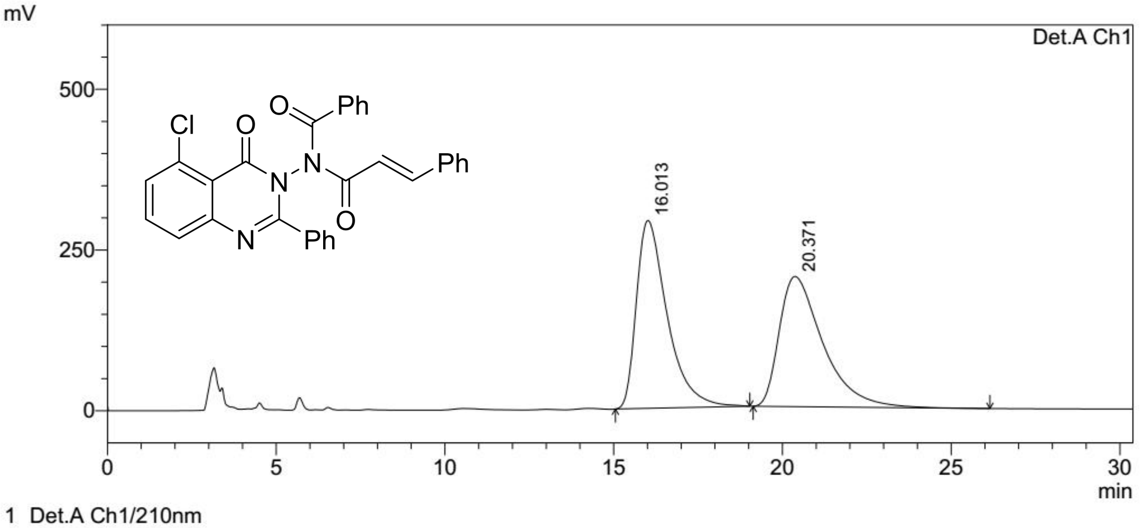

PeakTable

Detector A Ch1 210nm

Peak#	Ret. Time	Area	Height	Area %	Height %
1	16.013	18567404	292216	50.472	59.075
2	20.371	18219889	202439	49.528	40.925
Total		36787293	494654	100.000	100.000

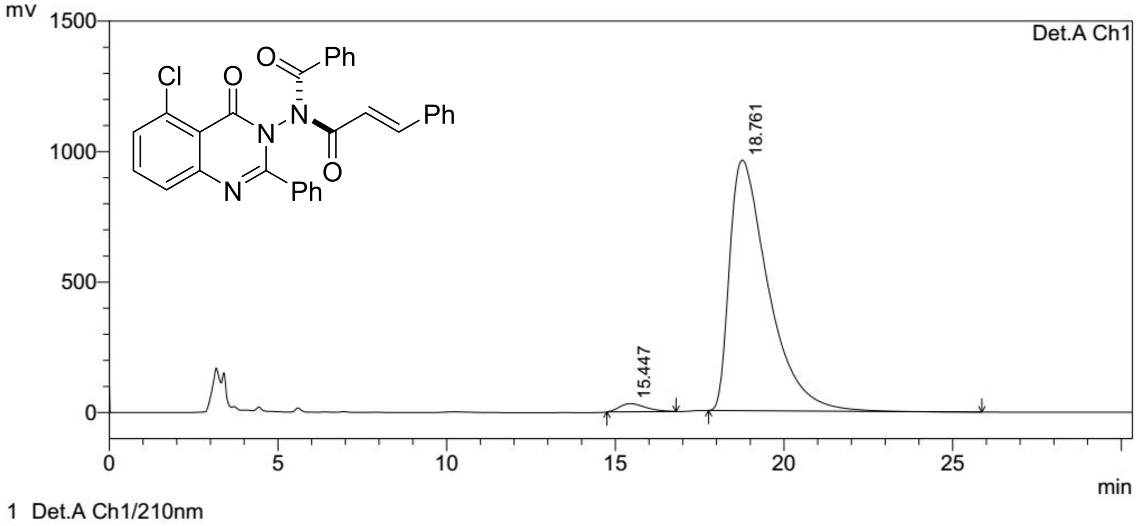

PeakTable

Detector A Ch1 210nm

Peak#	Ret. Time	Area	Height	Area %	Height %
1	15.447	1686862	31353	2.149	3.162
2	18.761	76799815	960294	97.851	96.838
Total		78486676	991647	100.000	100.000

($R_{a,cis,cis}$)-*N*-cinnamoyl-*N*-(5-methyl-4-oxo-2-phenylquinazolin-3(4*H*)-yl)benzamide (3ca)

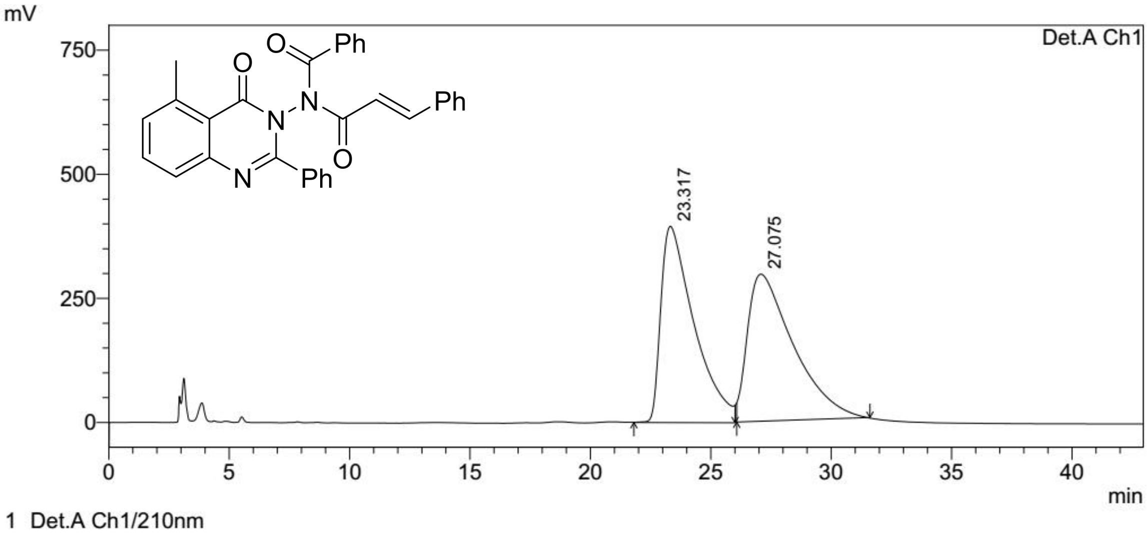

PeakTable

Detector A Ch1 210nm

Peak#	Ret. Time	Area	Height	Area %	Height %
1	23.317	37863452	395411	49.057	57.166
2	27.075	39319270	296279	50.943	42.834
Total		77182722	691690	100.000	100.000

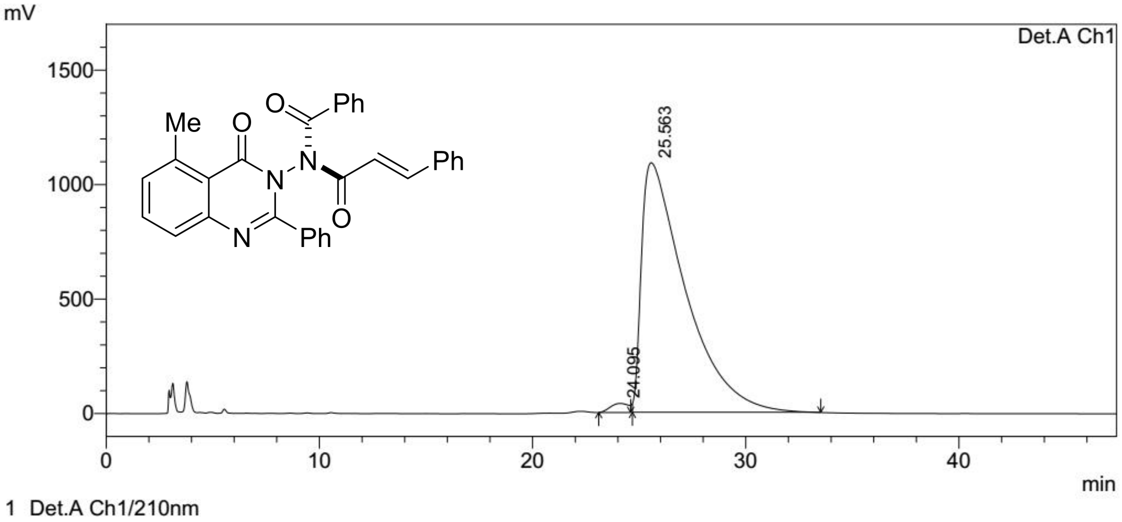

PeakTable

Detector A Ch1 210nm

Peak#	Ret. Time	Area	Height	Area %	Height %
1	24.095	2305419	39490	1.457	3.497
2	25.563	155921813	1089684	98.543	96.503
Total		158227232	1129174	100.000	100.000

($R_{a,cis,cis}$)-*N*-cinnamoyl-*N*-(6-methoxy-4-oxo-2-phenylquinazolin-3(4*H*)-yl)benzamide (3da)

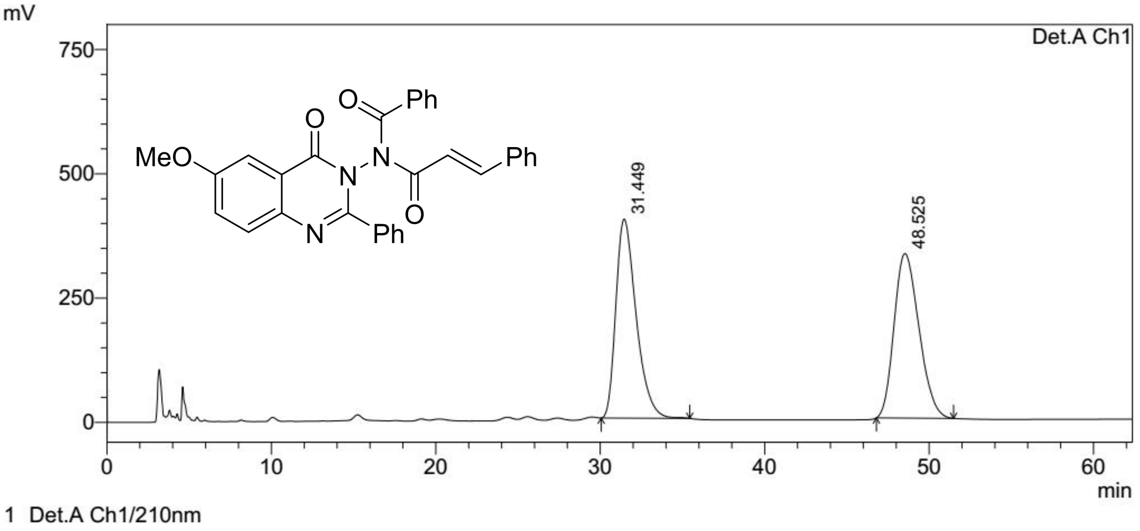

PeakTable

Detector A Ch1 210nm

Peak#	Ret. Time	Area	Height	Area %	Height %
1	31.449	34345065	400299	49.690	54.744
2	48.525	34772938	330927	50.310	45.256
Total		69118002	731225	100.000	100.000

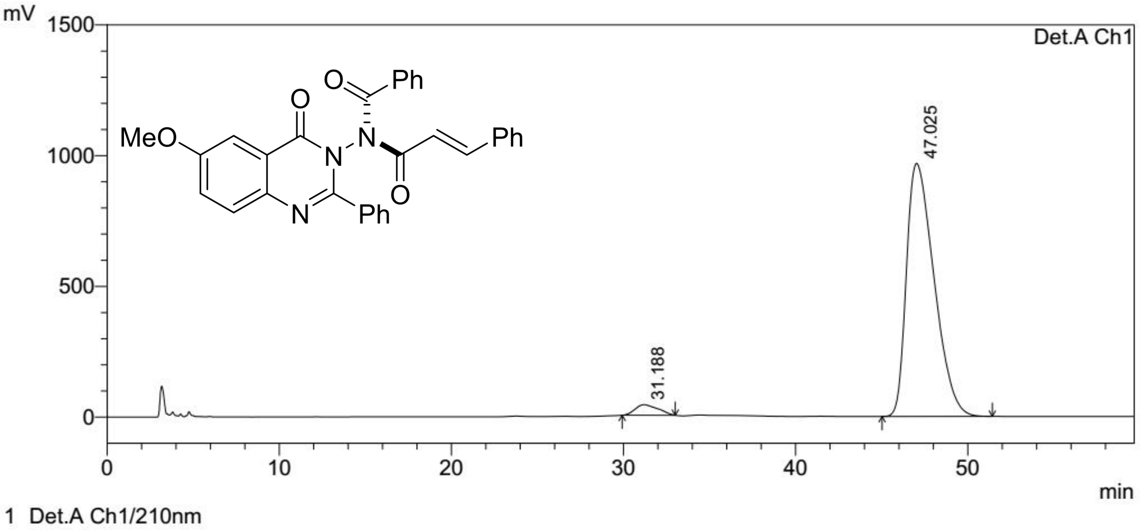

PeakTable

Detector A Ch1 210nm

Peak#	Ret. Time	Area	Height	Area %	Height %
1	31.188	3751911	40346	3.364	4.001
2	47.025	107780640	968142	96.636	95.999
Total		111532551	1008488	100.000	100.000

($R_{a,cis,cis}$)-*N*-cinnamoyl-*N*-(6-iodo-4-oxo-2-phenylquinazolin-3(4*H*)-yl)benzamide (3ea)

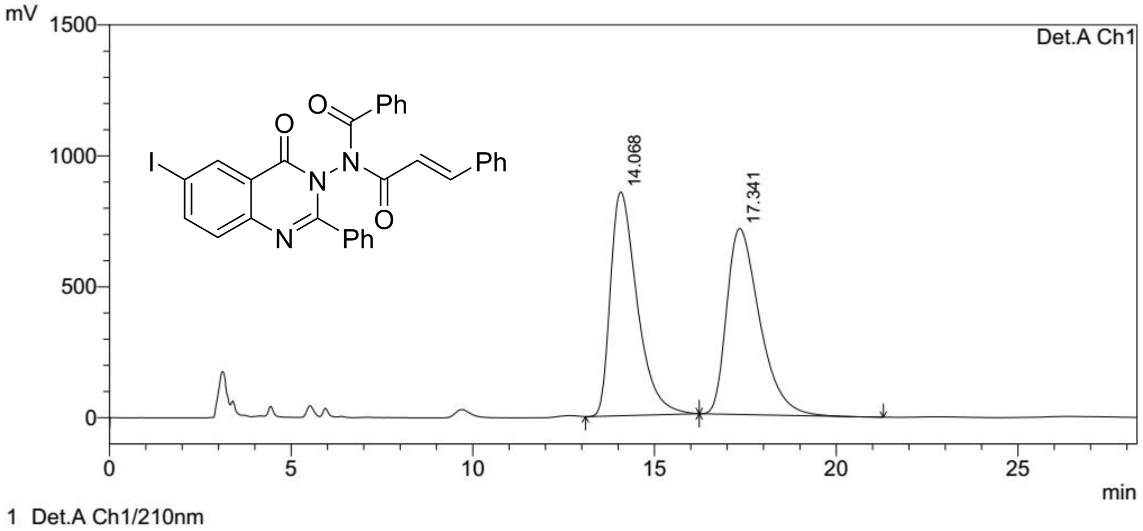

PeakTable

Detector A Ch1 210nm

Peak#	Ret. Time	Area	Height	Area %	Height %
1	14.068	43750739	854003	49.405	54.589
2	17.341	44804427	710422	50.595	45.411
Total		88555166	1564425	100.000	100.000

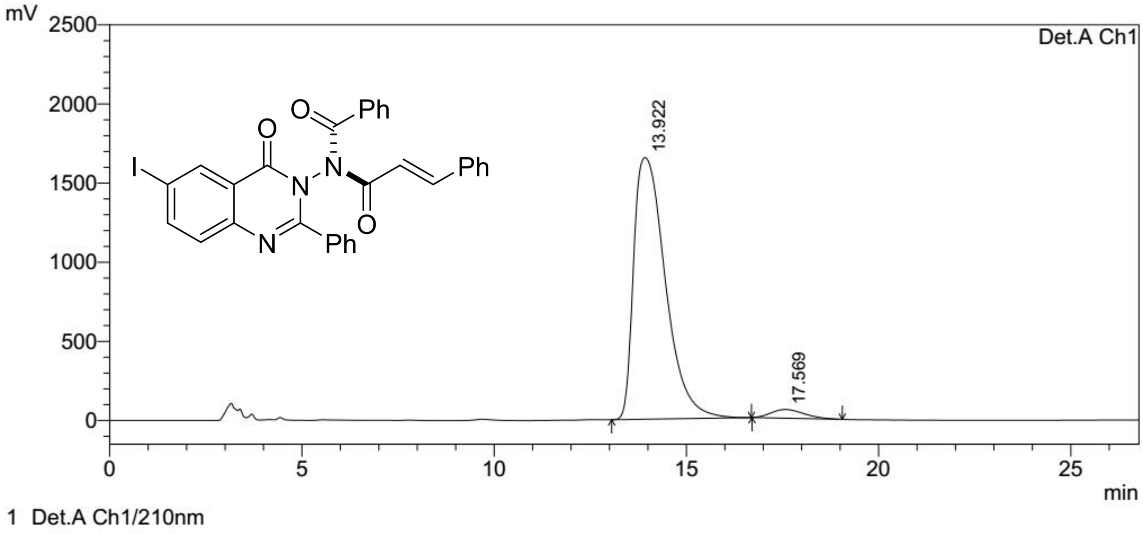

PeakTable

Detector A Ch1 210nm

Peak#	Ret. Time	Area	Height	Area %	Height %
1	13.922	94827796	1653376	96.653	96.786
2	17.569	3283753	54911	3.347	3.214
Total		98111549	1708286	100.000	100.000

($R_{a,cis,cis}$)-*N*-(7-bromo-4-oxo-2-phenylquinazolin-3(4*H*)-yl)-*N*-cinnamoylbenzamide (3fa)

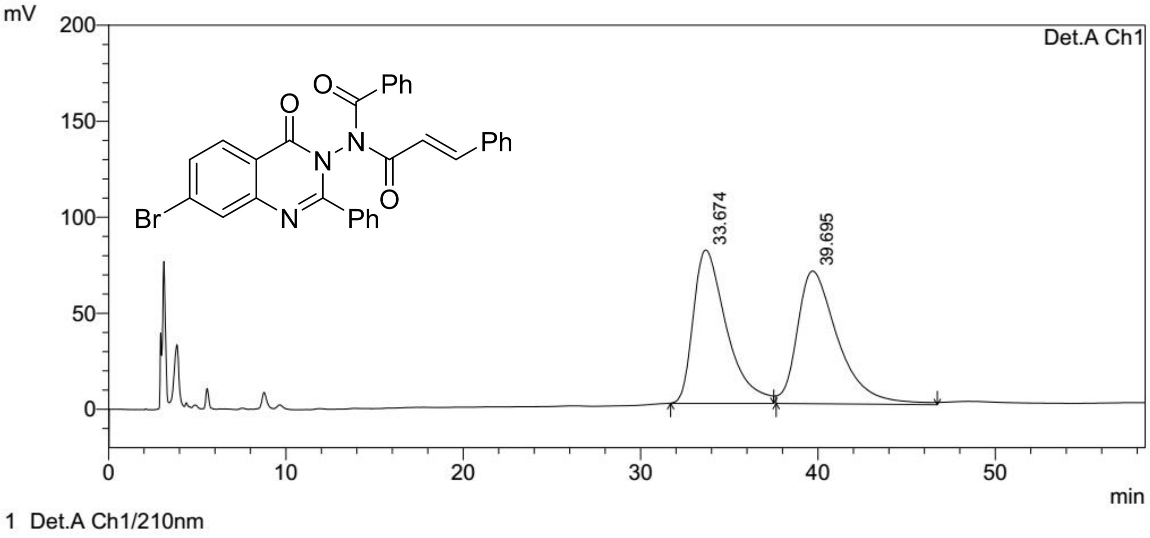

PeakTable

Detector A Ch1 210nm

Peak#	Ret. Time	Area	Height	Area %	Height %
1	33.674	10417988	79899	48.477	53.629
2	39.695	11072471	69085	51.523	46.371
Total		21490459	148984	100.000	100.000

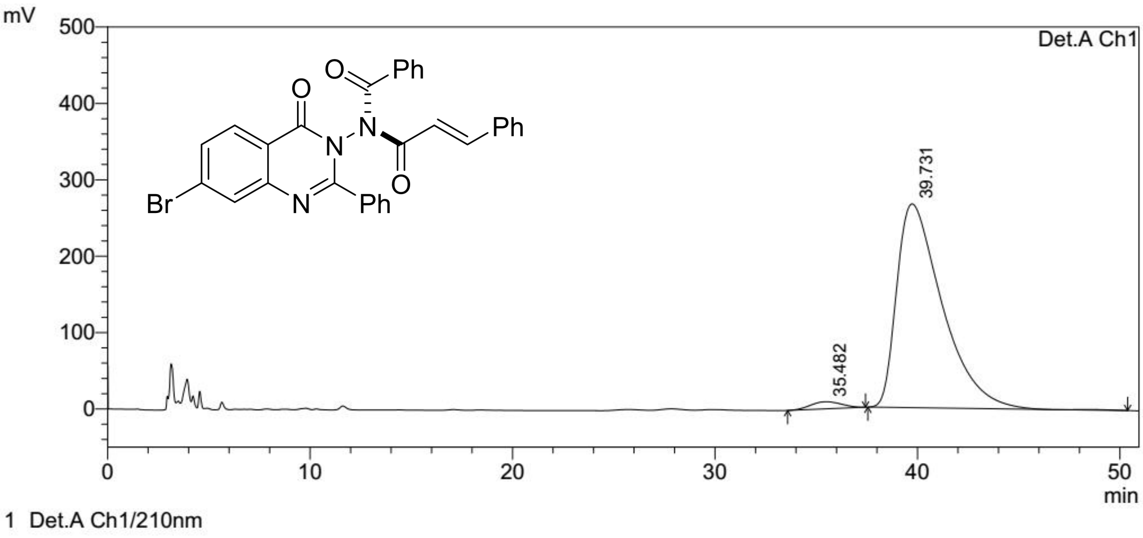

PeakTable

Detector A Ch1 210nm

Peak#	Ret. Time	Area	Height	Area %	Height %
1	35.482	985123	9309	2.300	3.371
2	39.731	41841413	266795	97.700	96.629
Total		42826536	276103	100.000	100.000

($R_{a,cis,cis}$)-*N*-(8-chloro-4-oxo-2-phenylquinazolin-3(4*H*)-yl)-*N*-cinnamoylbenzamide (3ga)

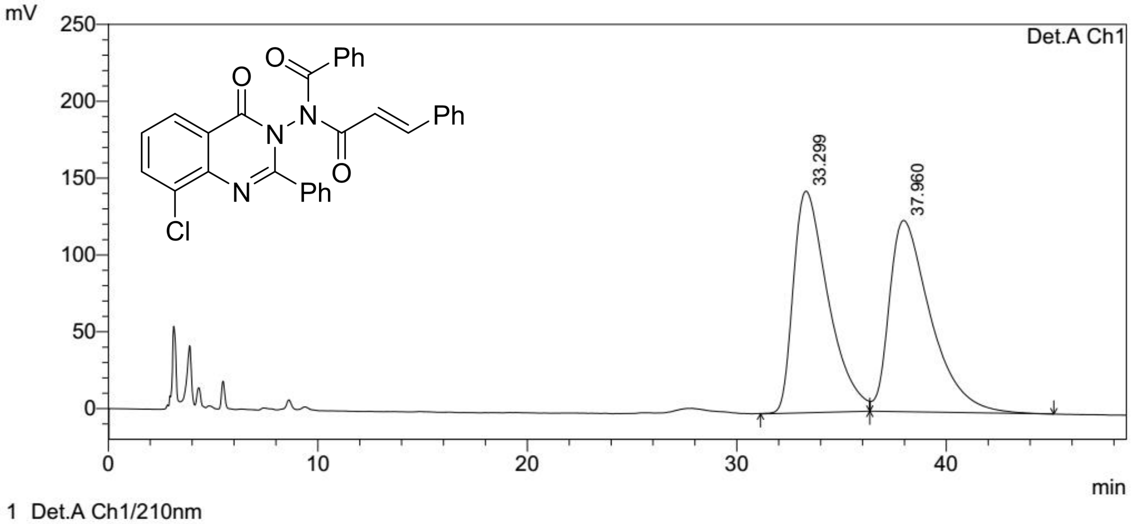

PeakTable

Detector A Ch1 210nm

Peak#	Ret. Time	Area	Height	Area %	Height %
1	33.299	16653643	144250	49.318	53.685
2	37.960	17114013	124446	50.682	46.315
Total		33767656	268696	100.000	100.000

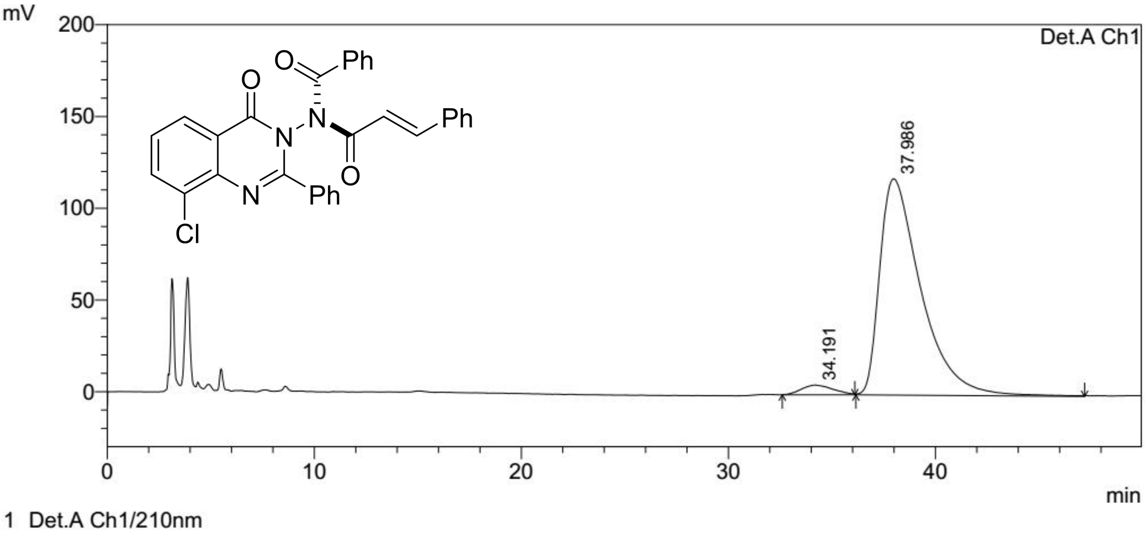

PeakTable

Detector A Ch1 210nm

Peak#	Ret. Time	Area	Height	Area %	Height %
1	34.191	560483	5247	3.316	4.262
2	37.986	16344462	117849	96.684	95.738
Total		16904946	123095	100.000	100.000

($R_{a,cis,cis}$)-4-chloro-*N*-cinnamoyl-*N*-(4-oxo-2-phenylquinazolin-3(4*H*)-yl)benzamide (3ha)

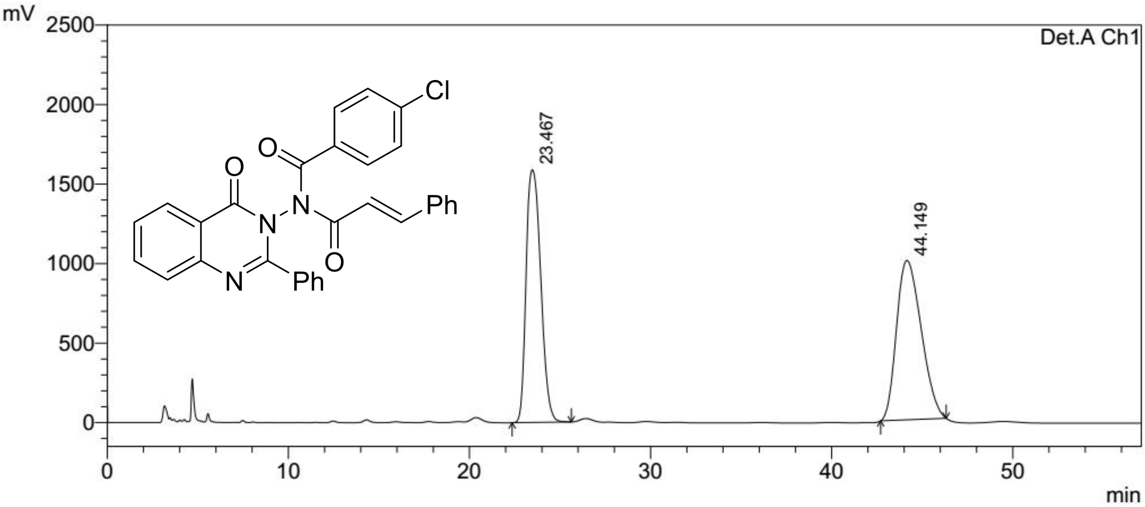

1 Det.A Ch1/210nm

PeakTable

Detector A Ch1 210nm

Peak#	Ret. Time	Area	Height	Area %	Height %
1	23.467	89302817	1589466	48.411	61.324
2	44.149	95163728	1002435	51.589	38.676
Total		184466545	2591901	100.000	100.000

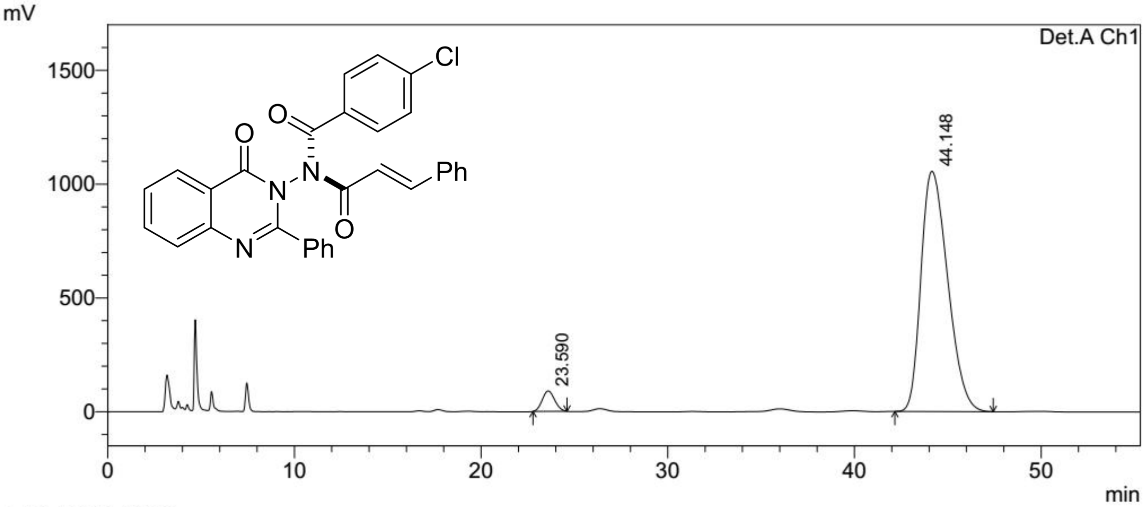

1 Det.A Ch1/210nm

PeakTable

Detector A Ch1 210nm

Peak#	Ret. Time	Area	Height	Area %	Height %
1	23.590	4140268	89503	3.794	7.816
2	44.148	104997794	1055570	96.206	92.184
Total		109138062	1145073	100.000	100.000

($R_{a,cis,cis}$)-*N*-cinnamoyl-4-methyl-*N*-(4-oxo-2-phenylquinazolin-3(4*H*)-yl)ben zamide (3ia)

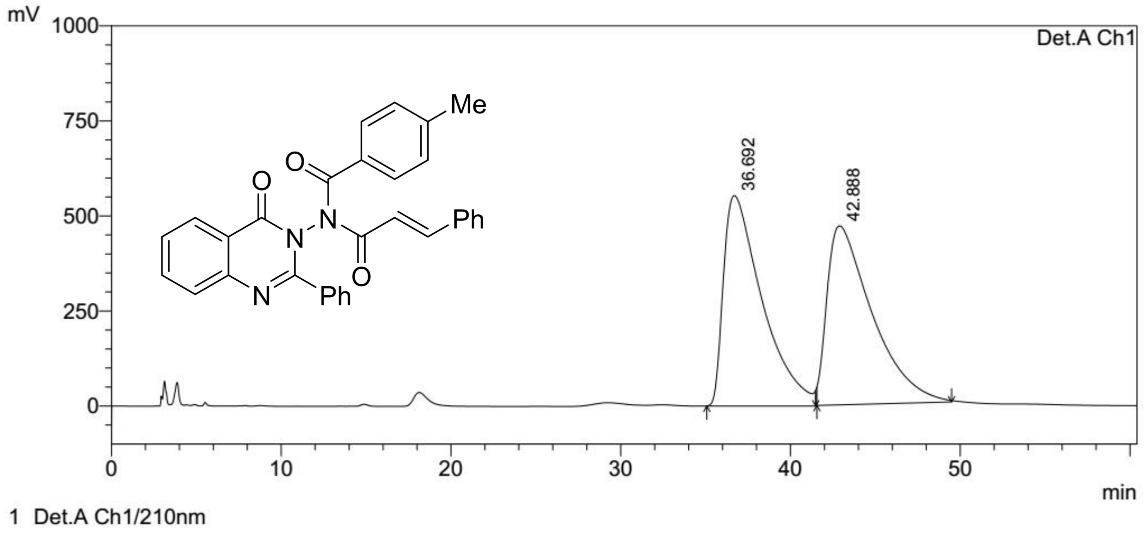

PeakTable

Detector A Ch1 210nm

Peak#	Ret. Time	Area	Height	Area %	Height %
1	36.692	87751023	553565	50.178	54.029
2	42.888	87128192	470997	49.822	45.971
Total		174879214	1024562	100.000	100.000

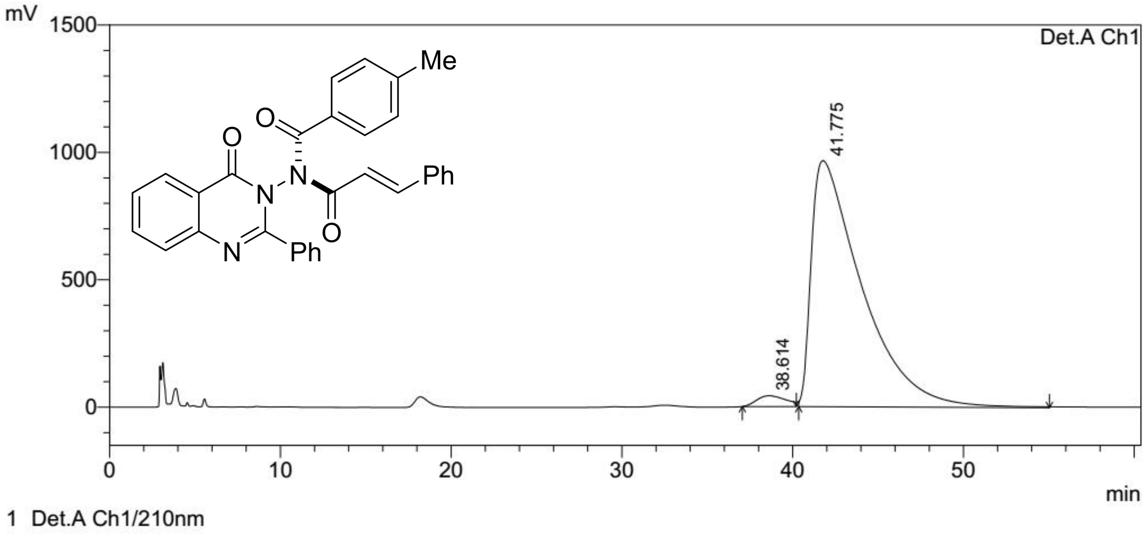

PeakTable

Detector A Ch1 210nm

Peak#	Ret. Time	Area	Height	Area %	Height %
1	38.614	5027290	42399	2.464	4.205
2	41.775	199026773	965819	97.536	95.795
Total		204054063	1008219	100.000	100.000

($R_{a,cis,cis}$)-*N*-cinnamoyl-3-methoxy-*N*-(4-oxo-2-phenylquinazolin-3(4*H*)-yl)benzamide (3ja)

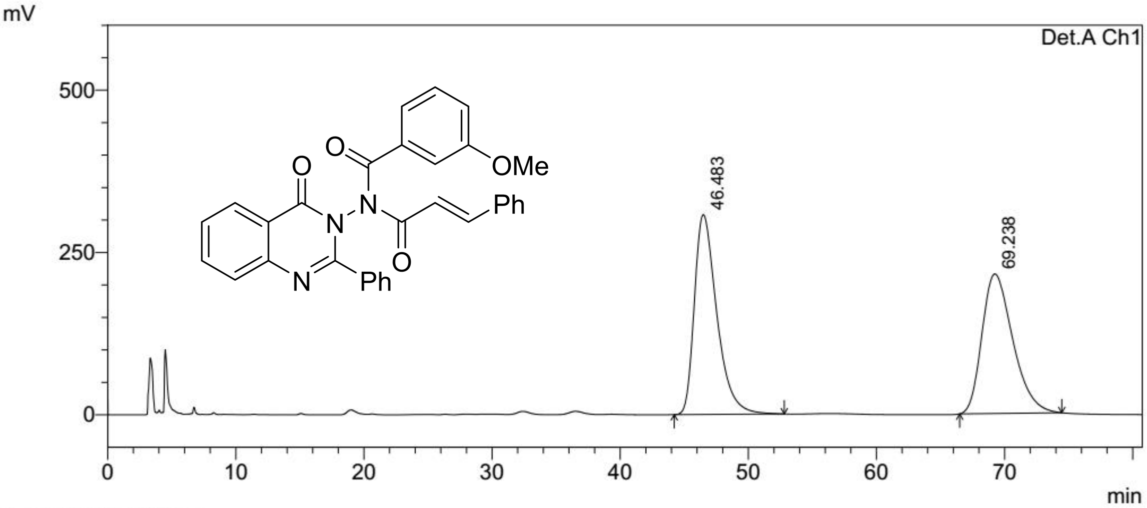

1 Det.A Ch1/210nm

PeakTable

Detector A Ch1 210nm

Peak#	Ret. Time	Area	Height	Area %	Height %
1	46.483	37480639	307855	51.040	58.888
2	69.238	35953089	214925	48.960	41.112
Total		73433727	522780	100.000	100.000

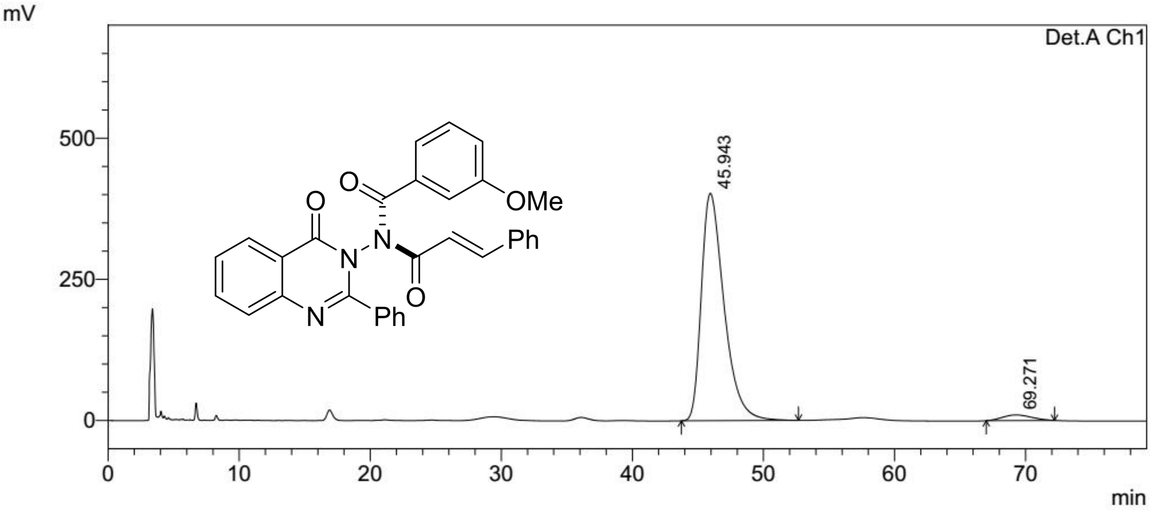

1 Det.A Ch1/210nm

PeakTable

Detector A Ch1 210nm

Peak#	Ret. Time	Area	Height	Area %	Height %
1	45.943	48449543	402640	96.937	97.585
2	69.271	1530909	9966	3.063	2.415
Total		49980453	412606	100.000	100.000

(*R*$_{a,cis,cis}$)-*N*-cinnamoyl-2-iodo-*N*-(4-oxo-2-phenylquinazolin-3(4*H*)-yl)benzamide (3ka)

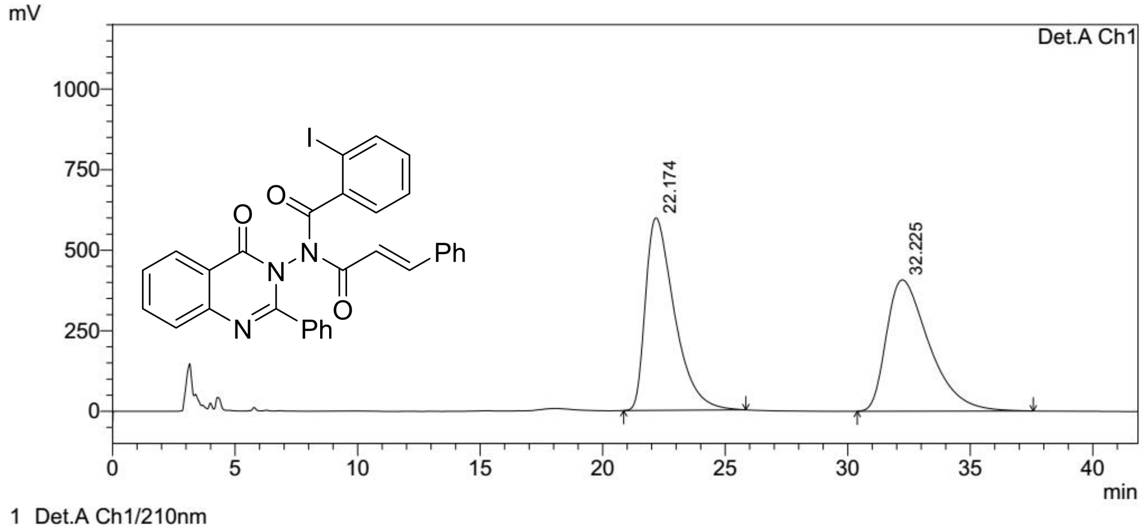

PeakTable

Detector A Ch1 210nm

Peak#	Ret. Time	Area	Height	Area %	Height %
1	22.174	49214481	597251	49.914	59.433
2	32.225	49384864	407660	50.086	40.567
Total		98599345	1004910	100.000	100.000

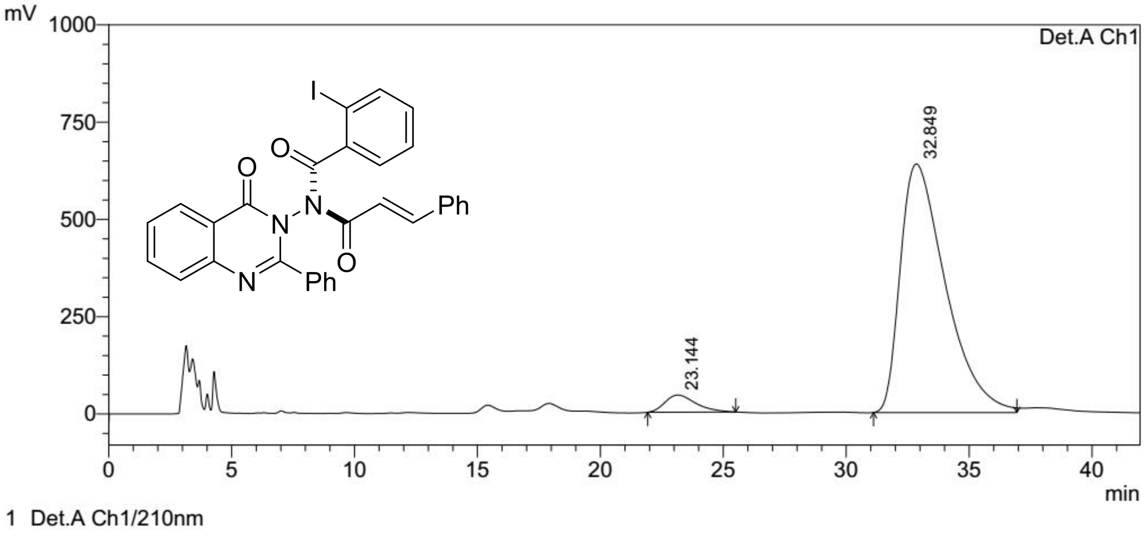

PeakTable

Detector A Ch1 210nm

Peak#	Ret. Time	Area	Height	Area %	Height %
1	23.144	3844458	44057	4.484	6.447
2	32.849	81899133	639270	95.516	93.553
Total		85743591	683327	100.000	100.000

($R_{a,cis,cis}$)-*N*-acetyl-*N*-(4-oxo-2-phenylquinazolin-3(4*H*)-yl)cinnamamide (3la)

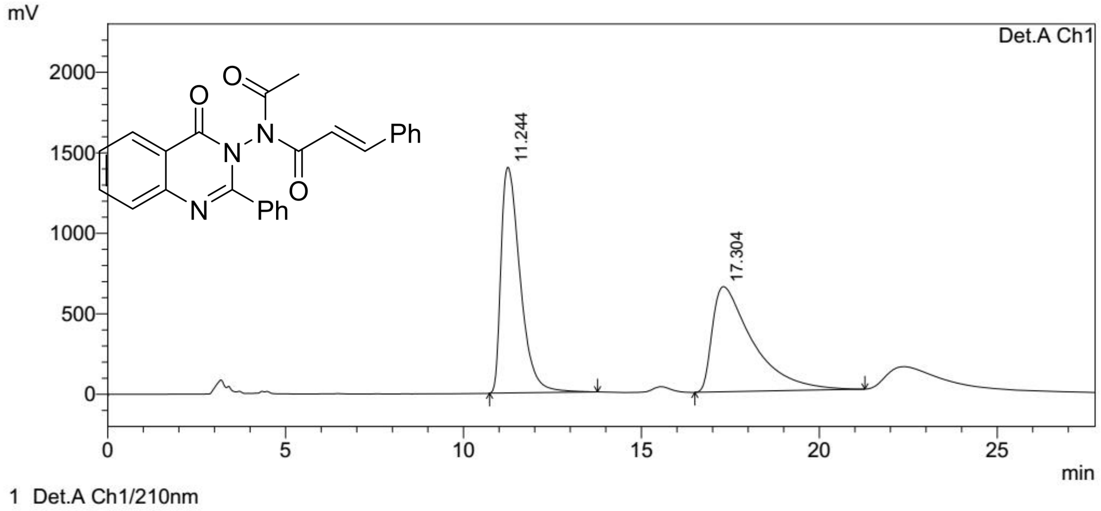

PeakTable

Detector A Ch1 210nm

Peak#	Ret. Time	Area	Height	Area %	Height %
1	11.244	52470292	1401699	50.348	68.204
2	17.304	51744321	653454	49.652	31.796
Total		104214613	2055152	100.000	100.000

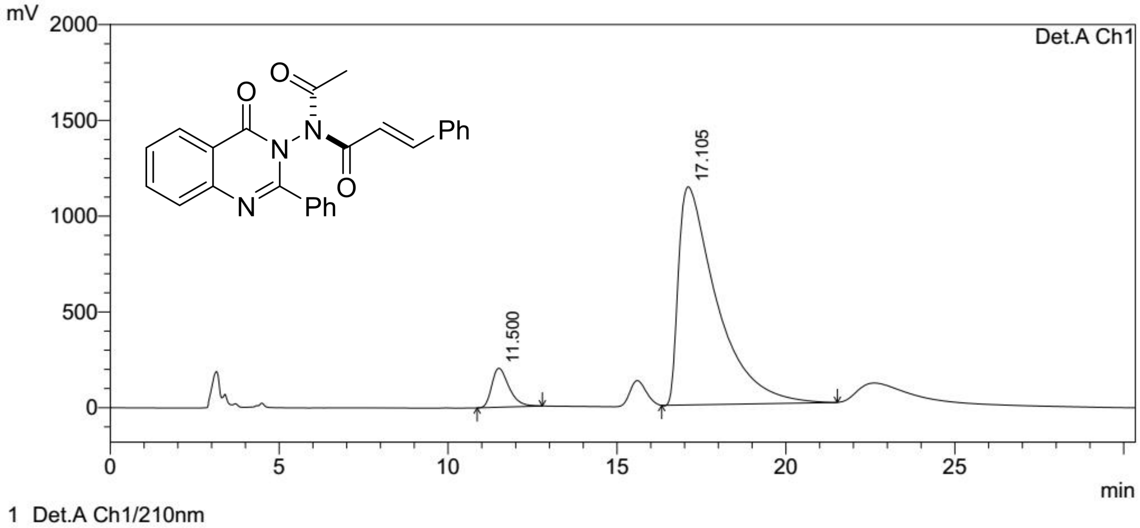

PeakTable

Detector A Ch1 210nm

Peak#	Ret. Time	Area	Height	Area %	Height %
1	11.500	7212986	203451	7.421	15.157
2	17.105	89985026	1138837	92.579	84.843
Total		97198012	1342288	100.000	100.000

($R_{a,cis,cis}$)-*N*-(4-oxo-2-phenylquinazolin-3(4*H*)-yl)-*N*-(2-phenylacetyl)cinnamamide (3ma)

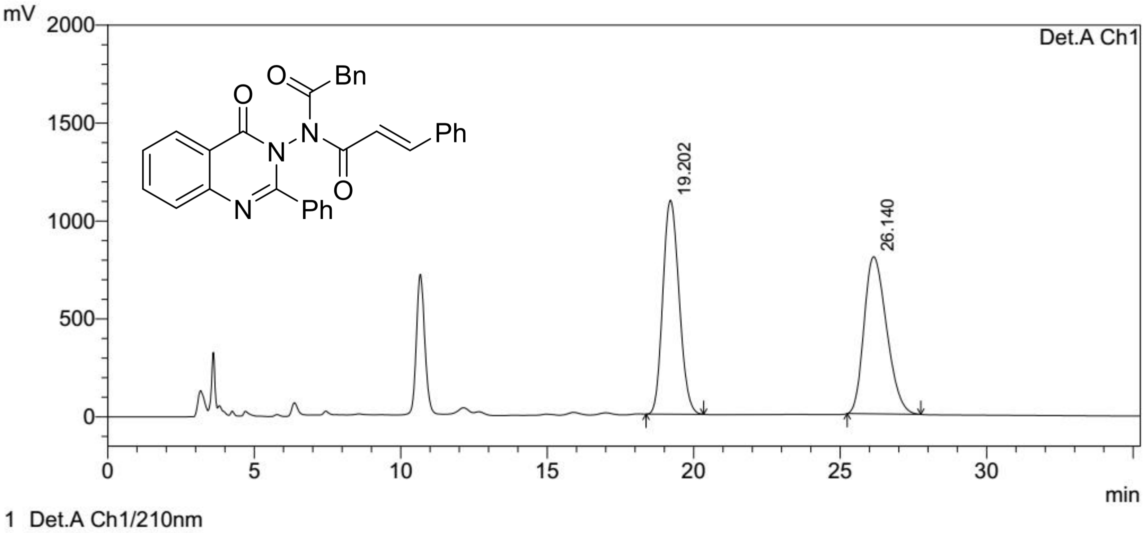

PeakTable

Detector A Ch1 210nm

Peak#	Ret. Time	Area	Height	Area %	Height %
1	19.202	42193620	1092847	49.496	57.656
2	26.140	43052471	802624	50.504	42.344
Total		85246091	1895470	100.000	100.000

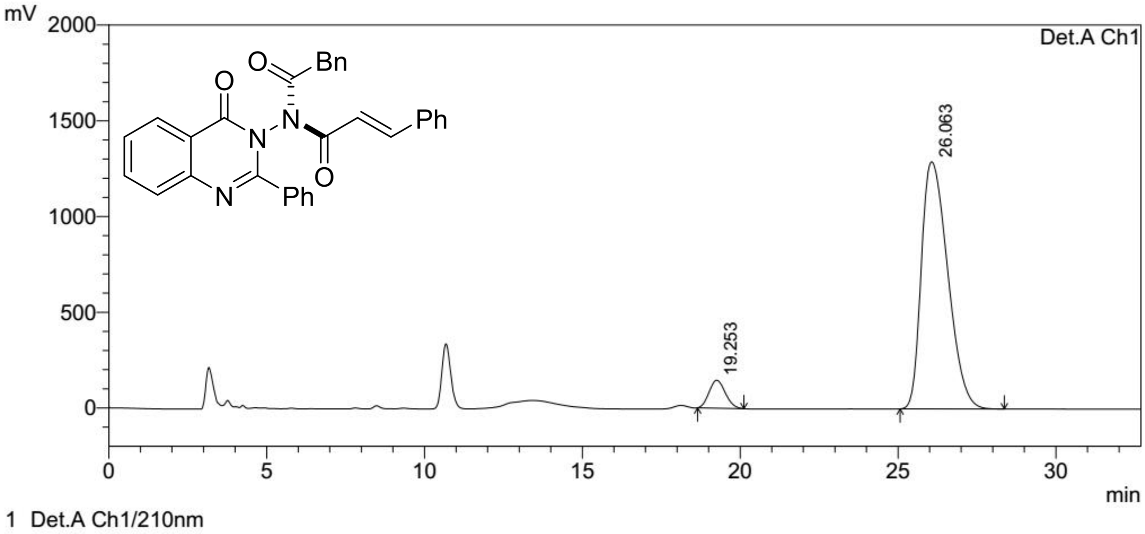

PeakTable

Detector A Ch1 210nm

Peak#	Ret. Time	Area	Height	Area %	Height %
1	19.253	5217122	145683	6.485	10.142
2	26.063	75234782	1290751	93.515	89.858
Total		80451904	1436433	100.000	100.000

($R_{a,cis,cis}$)-*N*-isobutyryl-*N*-(4-oxo-2-phenylquinazolin-3(4*H*)-yl)cinnamamide (3na)

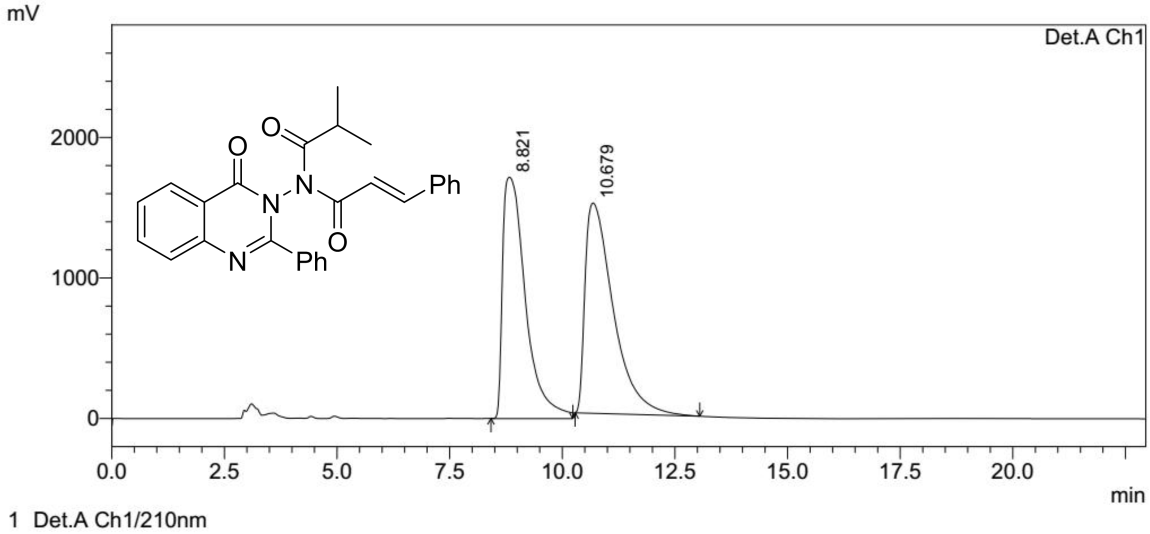

PeakTable

Detector A Ch1 210nm

Peak#	Ret. Time	Area	Height	Area %	Height %
1	8.821	60583949	1716566	48.167	53.430
2	10.679	65194278	1496156	51.833	46.570
Total		125778227	3212722	100.000	100.000

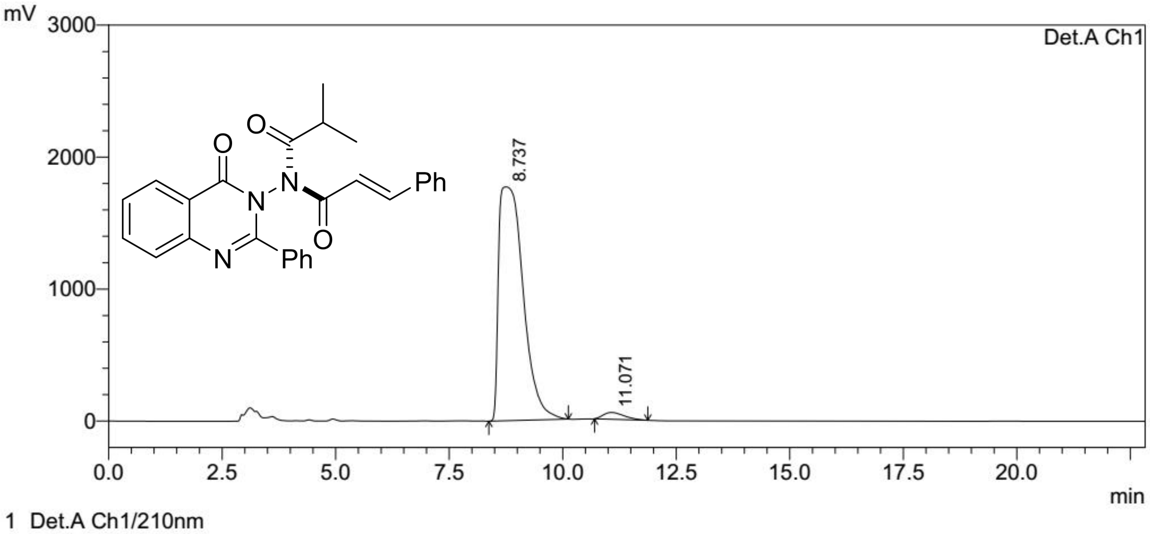

PeakTable

Detector A Ch1 210nm

Peak#	Ret. Time	Area	Height	Area %	Height %
1	8.737	66447698	1772257	97.571	97.169
2	11.071	1654489	51634	2.429	2.831
Total		68102188	1823891	100.000	100.000

($R_{a,cis,cis}$)-*N*-cinnamoyl-*N*-(4-oxo-2-phenylquinazolin-3(4*H*)-yl)cyclohexane carboxamide (3oa)

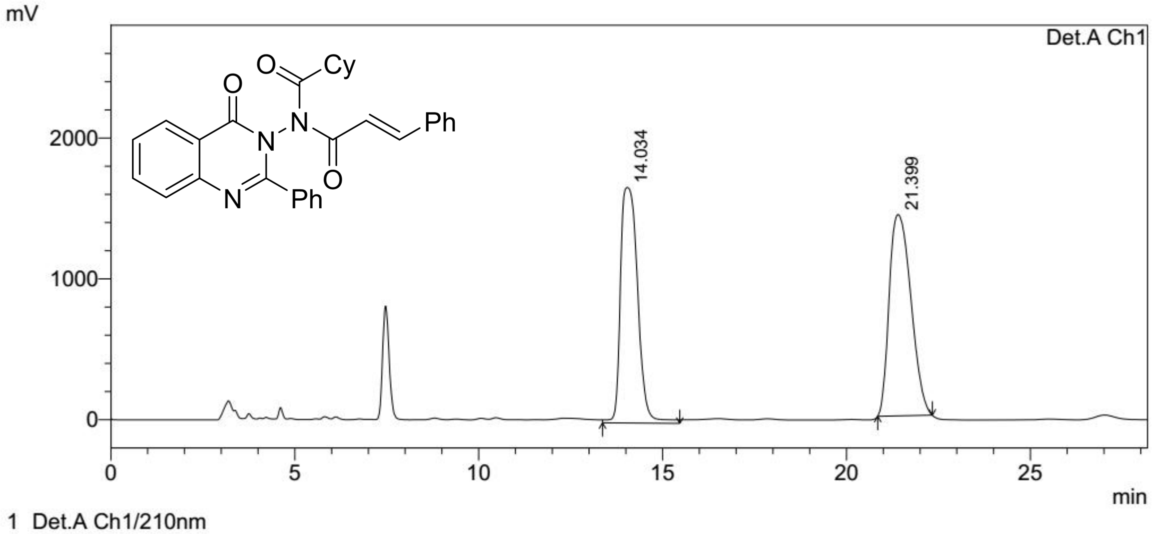

PeakTable

Detector A Ch1 210nm

Peak#	Ret. Time	Area	Height	Area %	Height %
1	14.034	54526456	1671541	48.202	53.915
2	21.399	58593742	1428792	51.798	46.085
Total		113120198	3100333	100.000	100.000

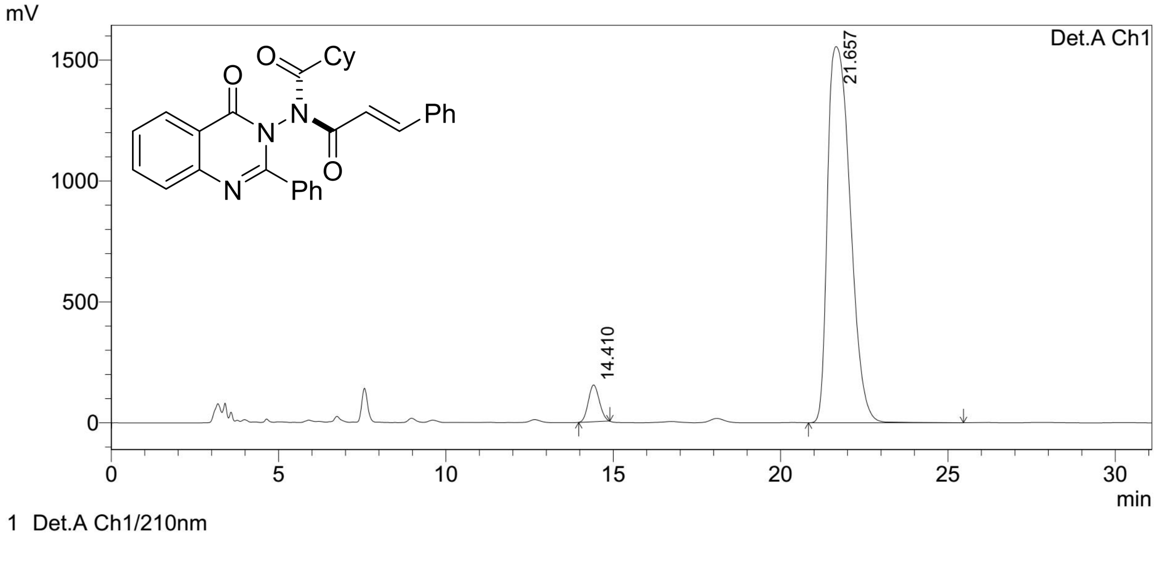

PeakTable

Detector A Ch1 210nm

Peak#	Ret. Time	Area	Height	Area %	Height %
1	14.410	3493539	151761	4.544	8.891
2	21.657	73394086	1555189	95.456	91.109
Total		76887625	1706950	100.000	100.000

($R_{a,cis,cis}$)-*N*-(2-(4-bromophenyl)-4-oxoquinazolin-3(4*H*)-yl)-*N*-cinnamoylbenzamide (3pa)

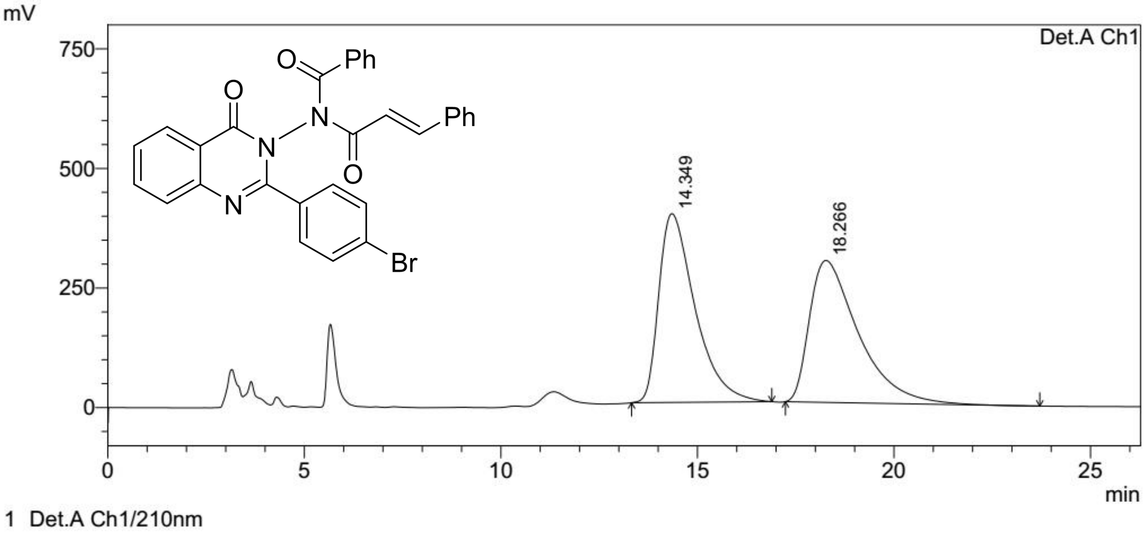

PeakTable

Detector A Ch1 210nm

Peak#	Ret. Time	Area	Height	Area %	Height %
1	14.349	25295840	394767	49.569	57.096
2	18.266	25736049	296645	50.431	42.904
Total		51031889	691413	100.000	100.000

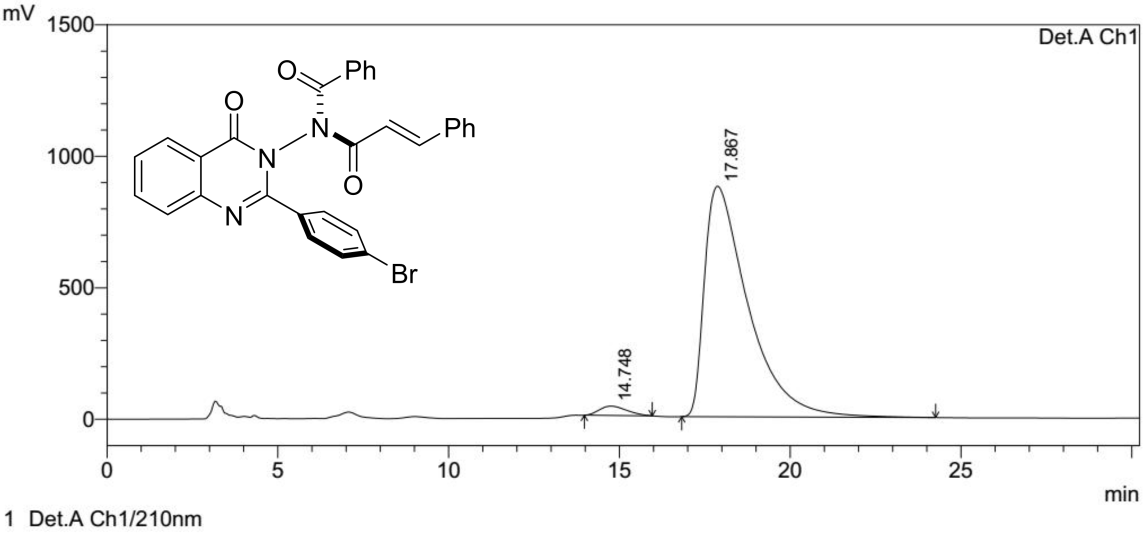

PeakTable

Detector A Ch1 210nm

Peak#	Ret. Time	Area	Height	Area %	Height %
1	14.748	1986938	35466	2.485	3.889
2	17.867	77984411	876459	97.515	96.111
Total		79971349	911925	100.000	100.000

($R_{a,cis,cis}$)-*N*-cinnamoyl-*N*-(2-(4-methoxyphenyl)-4-oxoquinazolin-3(4*H*)-yl) benzamide (3qa)

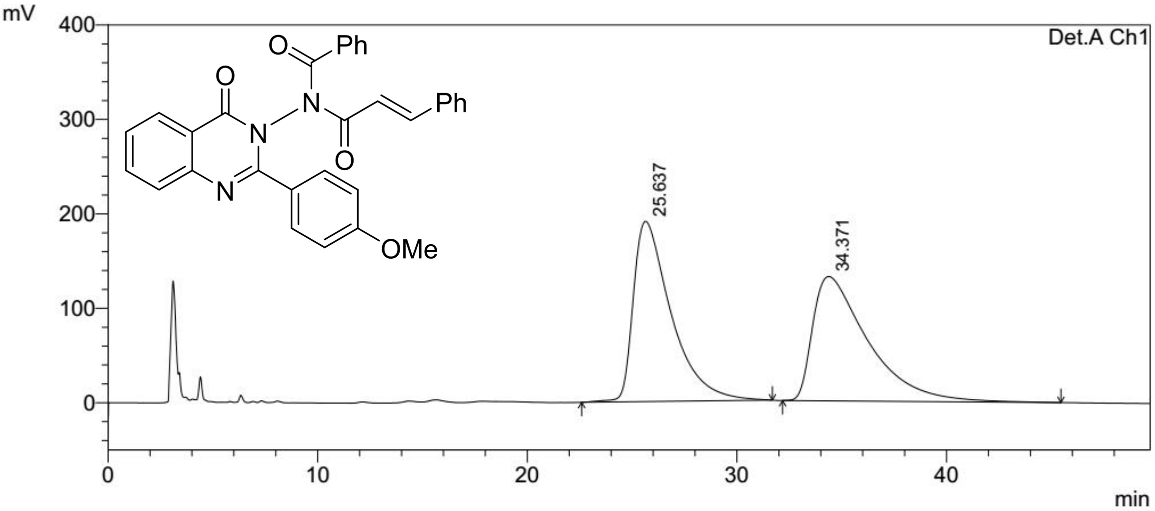

1 Det.A Ch1/210nm

PeakTable

Detector A Ch1 210nm

Peak#	Ret. Time	Area	Height	Area %	Height %
1	25.637	24004787	190815	49.863	59.181
2	34.371	24136922	131613	50.137	40.819
Total		48141709	322428	100.000	100.000

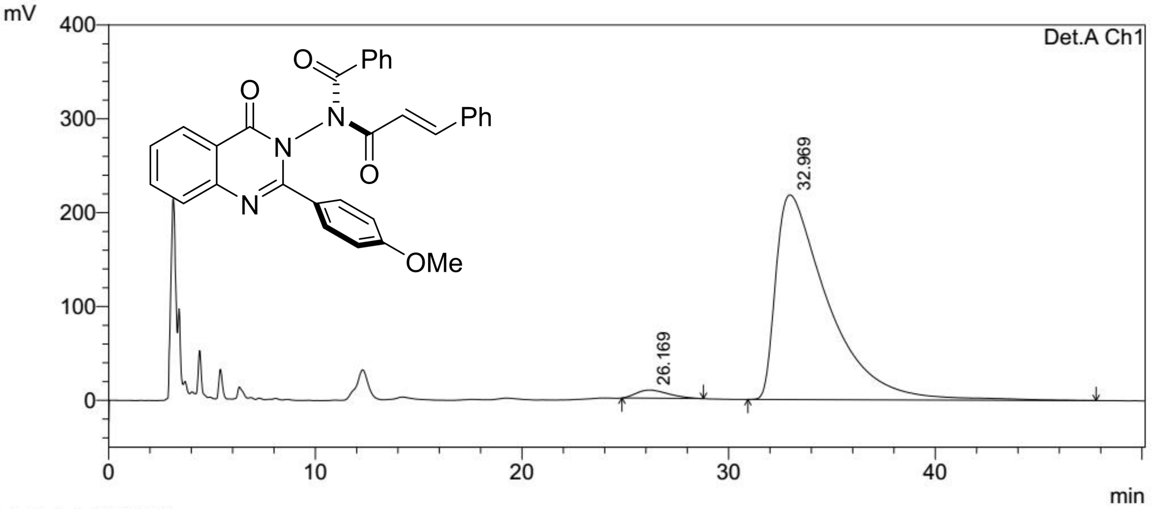

1 Det.A Ch1/210nm

PeakTable

Detector A Ch1 210nm

Peak#	Ret. Time	Area	Height	Area %	Height %
1	26.169	941540	8653	2.387	3.814
2	32.969	38502047	218217	97.613	96.186
Total		39443587	226870	100.000	100.000

($R_{a,cis,cis}$)-*N*-cinnamoyl-*N*-(4-oxo-2-(m-tolyl)quinazolin-3(4*H*)-yl)benzamide (3ra)

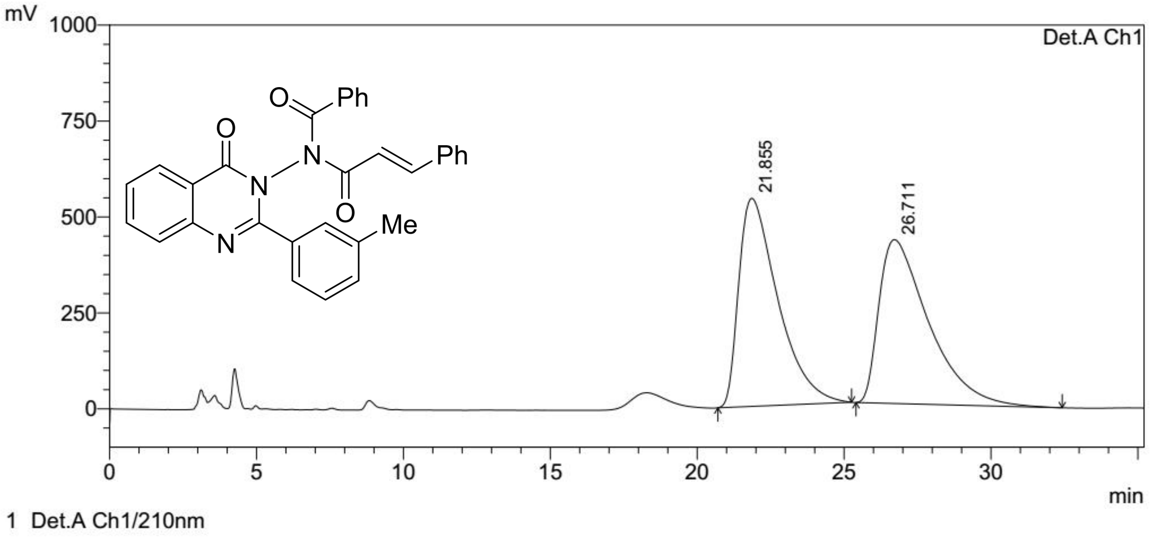

PeakTable

Detector A Ch1 210nm

Peak#	Ret. Time	Area	Height	Area %	Height %
1	21.855	50230494	542287	49.949	55.955
2	26.711	50332133	426853	50.051	44.045
Total		100562627	969139	100.000	100.000

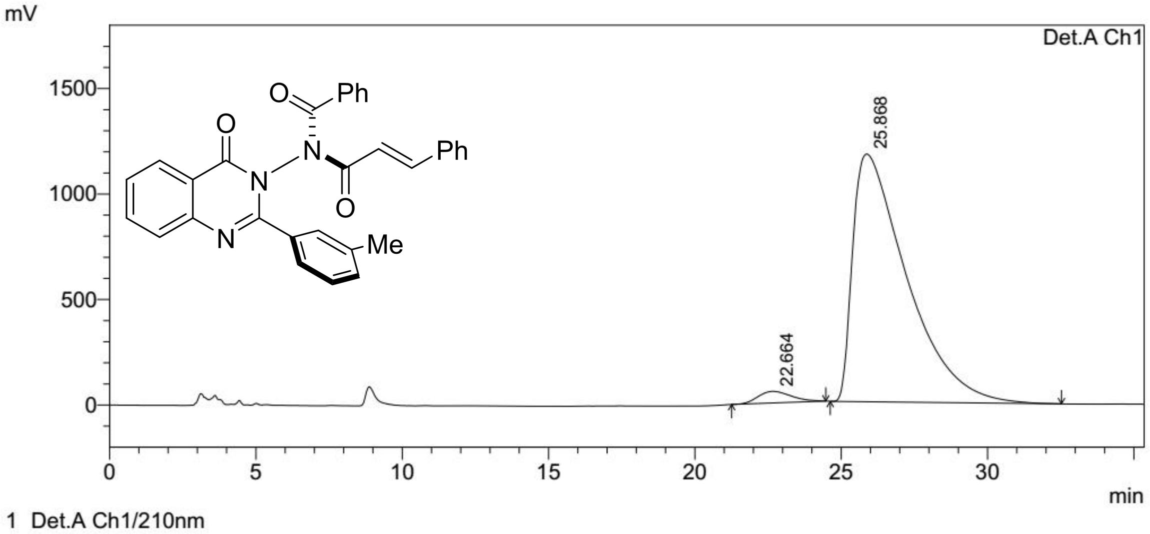

PeakTable

Detector A Ch1 210nm

Peak#	Ret. Time	Area	Height	Area %	Height %
1	22.664	4403254	55267	2.835	4.496
2	25.868	150894074	1174013	97.165	95.504
Total		155297328	1229280	100.000	100.000

($R_{a,cis,cis}$)-*N*-cinnamoyl-*N*-(2-isopropyl-4-oxoquinazolin-3(4*H*)-yl)benzamide (3sa)

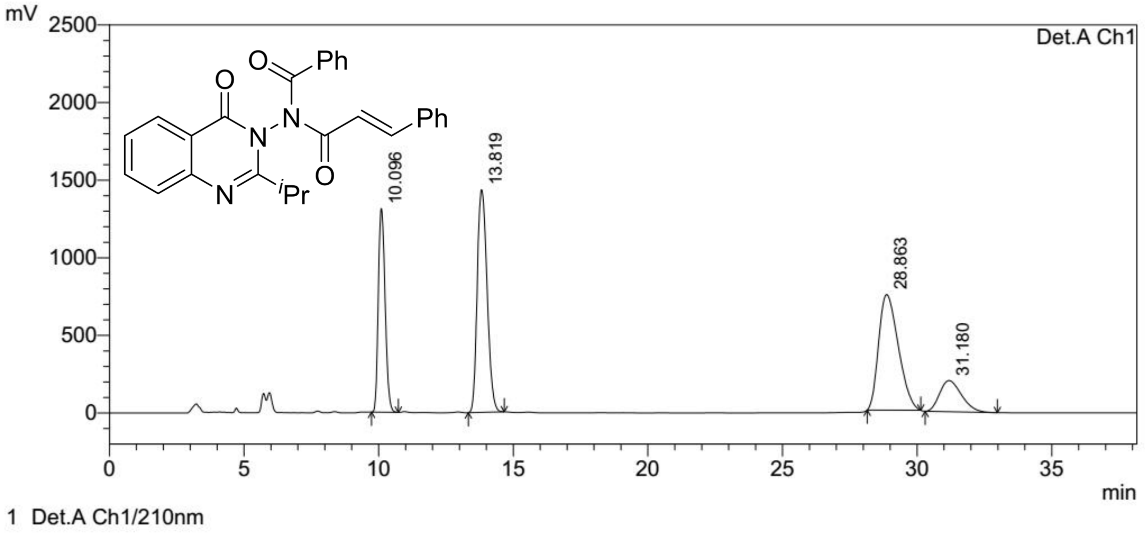

PeakTable

Detector A Ch1 210nm

Peak#	Ret. Time	Area	Height	Area %	Height %
1	10.096	23096378	1311528	21.207	35.541
2	13.819	36615615	1433448	33.621	38.844
3	28.863	37842855	744776	34.747	20.182
4	31.180	11353657	200473	10.425	5.433
Total		108908505	3690225	100.000	100.000

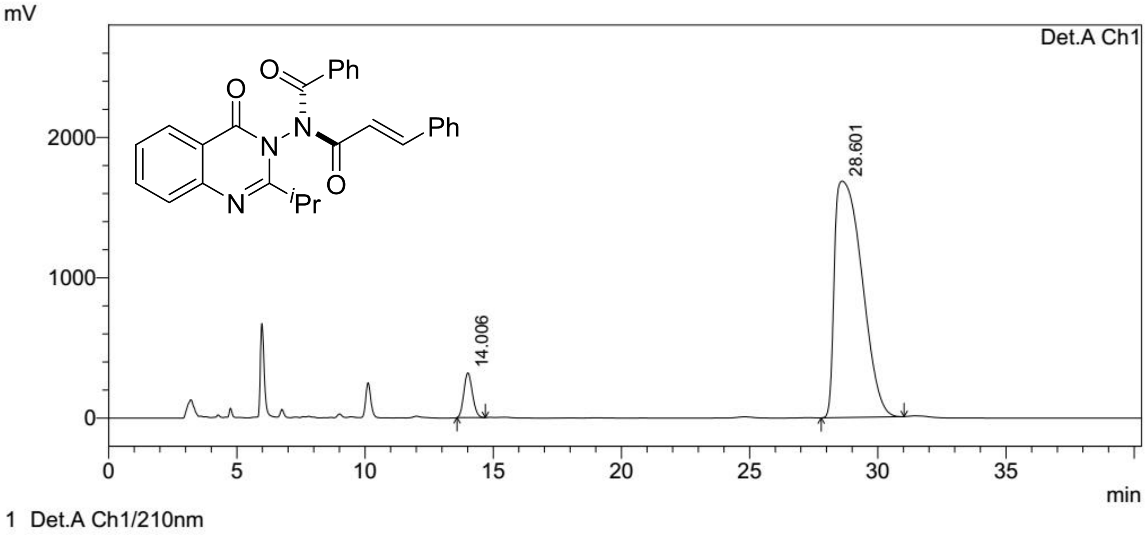

PeakTable

Detector A Ch1 210nm

Peak#	Ret. Time	Area	Height	Area %	Height %
1	14.006	7424260	317992	5.577	15.885
2	28.601	125704028	1683906	94.423	84.115
Total		133128288	2001898	100.000	100.000

($R_{a,cis,cis}$)-(*E*)-*N*-(2-(1-(4-chlorobenzoyl)-5-methoxy-2-methyl-1H-indol-3-yl) acetyl)-3-(4-methoxyphenyl)-*N*-(4-oxo-2-phenylquinazolin-3(4*H*)-yl)acrylamide (3tb)

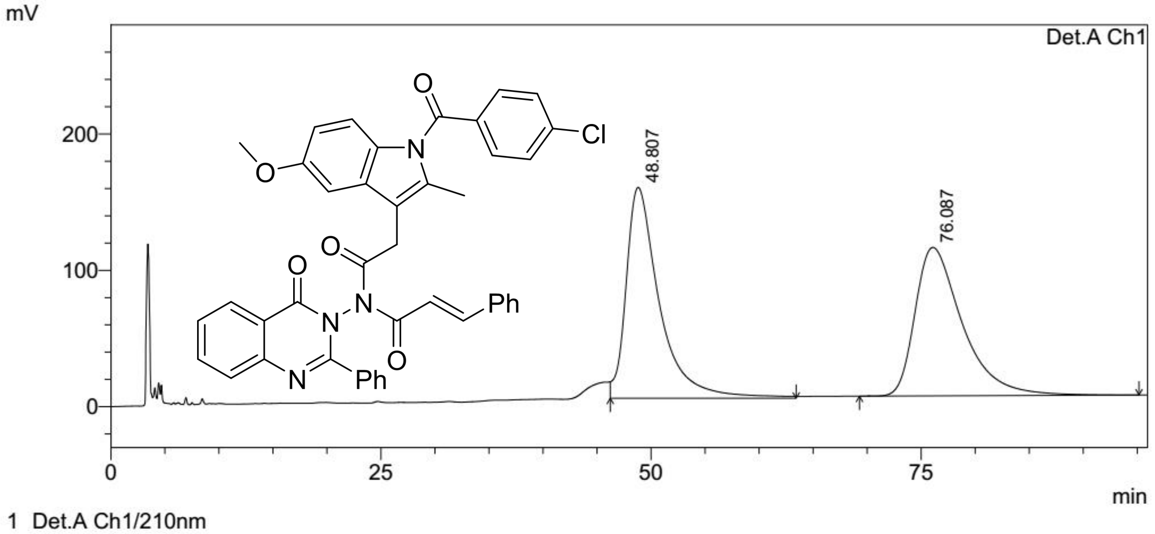

1 Det.A Ch1/210nm

PeakTable

Detector A Ch1 210nm

Peak#	Ret. Time	Area	Height	Area %	Height %
1	48.807	33294887	153893	50.107	58.584
2	76.087	33153231	108793	49.893	41.416
Total		66448118	262686	100.000	100.000

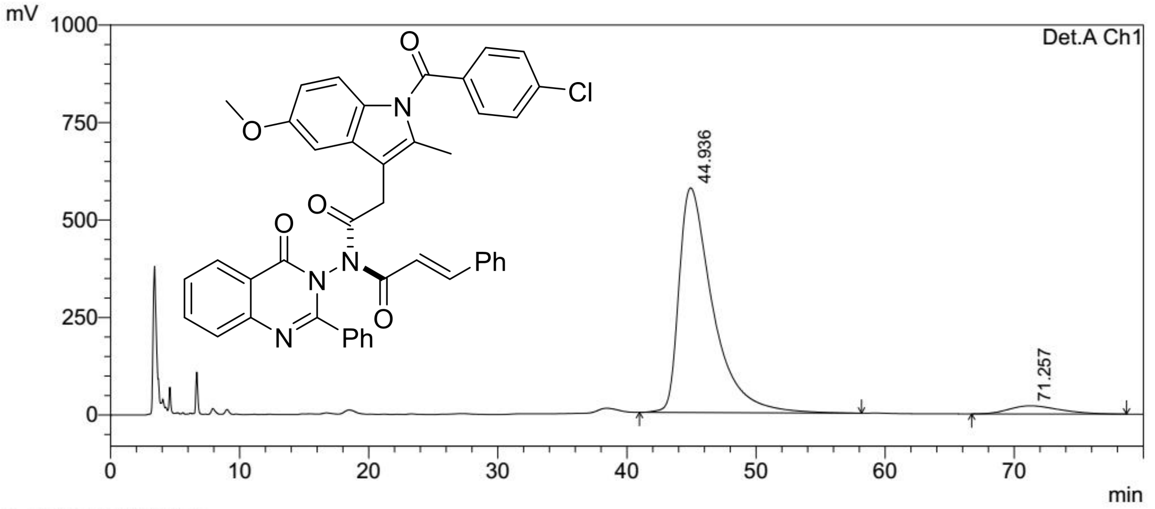

1 Det.A Ch1/210nm

PeakTable

Detector A Ch1 210nm

Peak#	Ret. Time	Area	Height	Area %	Height %
1	44.936	104830775	575682	94.788	96.499
2	71.257	5764165	20887	5.212	3.501
Total		110594940	596569	100.000	100.000

(*R*$_{a,cis,cis}$)-(*S*)-*N*-(2-(6-methoxynaphthalen-2-yl)propanoyl)-*N*-(4-oxo-2-phenylquinazolin-3(4*H*)-yl)cinnamamide (3ua)

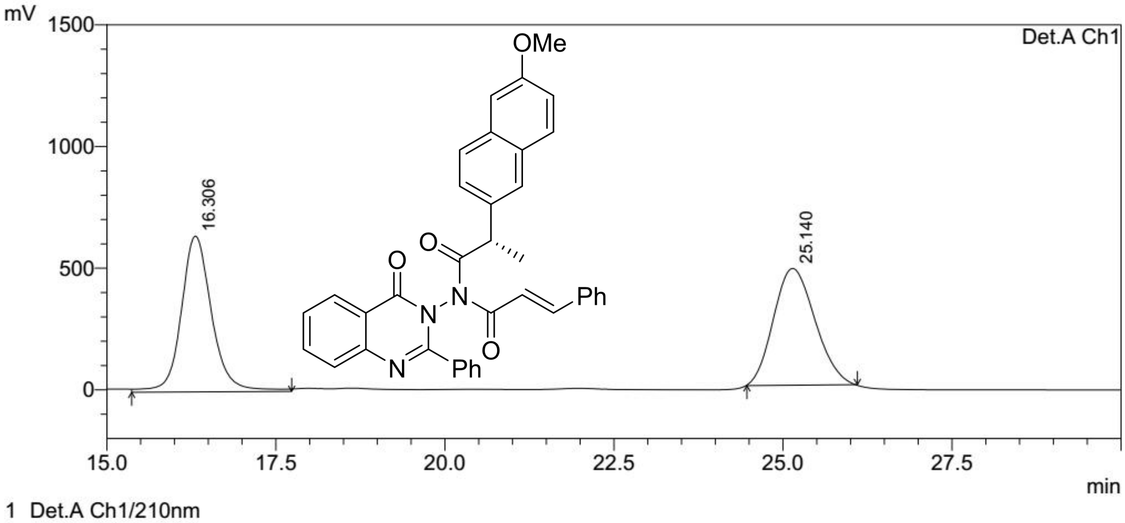

PeakTable

Detector A Ch1 210nm

Peak#	Ret. Time	Area	Height	Area %	Height %
1	16.306	20251807	639382	49.751	57.172
2	25.140	20454785	478957	50.249	42.828
Total		40706592	1118340	100.000	100.000

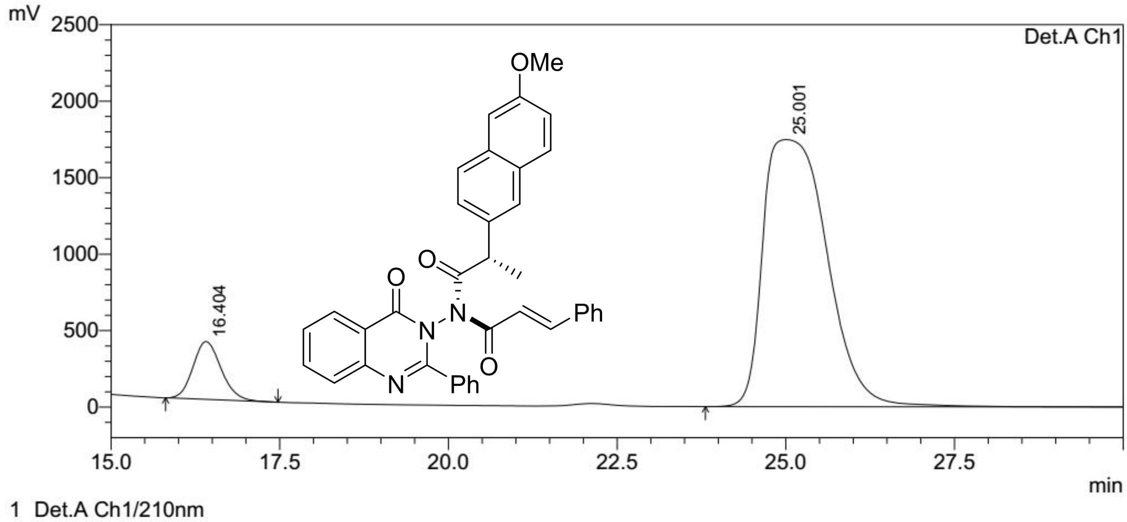

PeakTable

Detector A Ch1 210nm

Peak#	Ret. Time	Area	Height	Area %	Height %
1	16.404	10961648	371893	8.684	17.565
2	25.001	115262804	1745355	91.316	82.435
Total		126224452	2117247	100.000	100.000

($R_{a,cis,cis}$)-(*E*)-*N*-(4-oxo-2-phenylquinazolin-3(4*H*)-yl)-*N*-(3-(4-(trifluoromethy l)phenyl)acryloyl)benzamide (3ab)

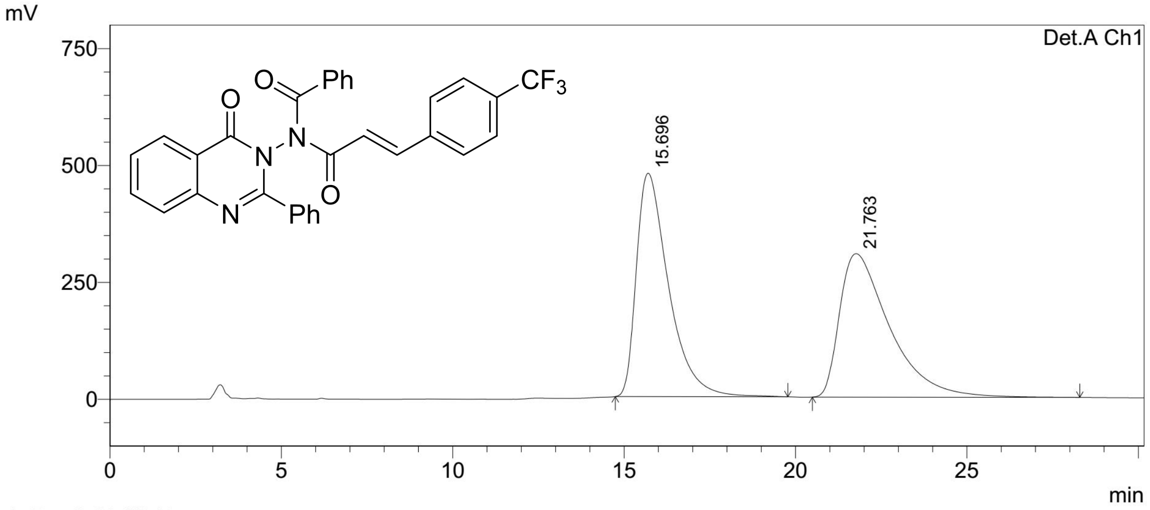

1 Det.A Ch1/210nm

PeakTable

Detector A Ch1 210nm

Peak#	Ret. Time	Area	Height	Area %	Height %
1	15.696	31251457	477958	50.038	60.886
2	21.763	31204464	307045	49.962	39.114
Total		62455921	785003	100.000	100.000

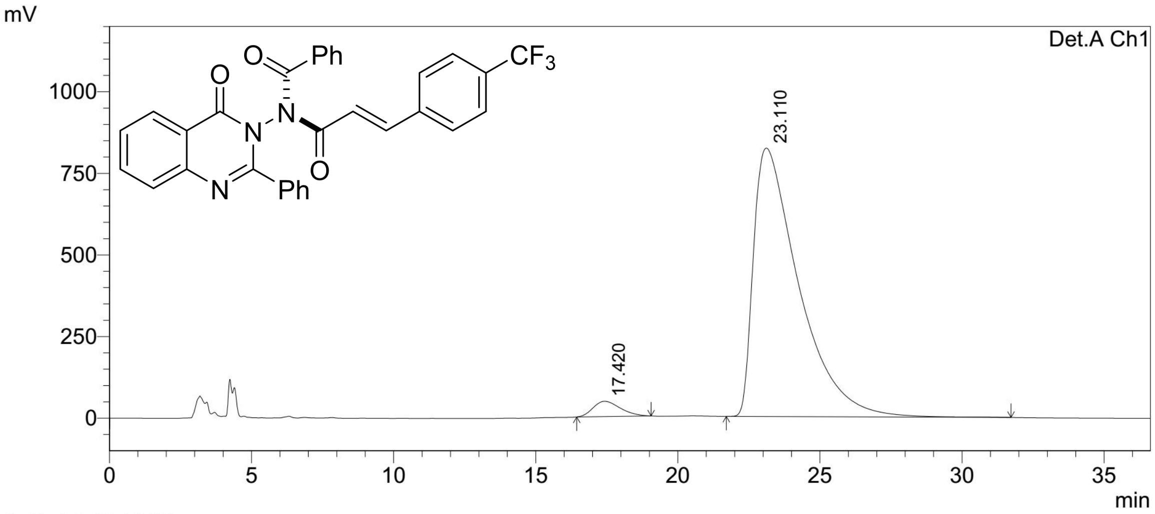

1 Det.A Ch1/210nm

PeakTable

Detector A Ch1 210nm

Peak#	Ret. Time	Area	Height	Area %	Height %
1	17.420	3129944	46925	3.316	5.398
2	23.110	91248491	822366	96.684	94.602
Total		94378434	869291	100.000	100.000

($R_{a,cis,cis}$)-(*E*)-*N*-(3-(3-bromophenyl)acryloyl)-*N*-(4-oxo-2-phenylquinazolin-3(4*H*)-yl)benzamide (3ac)

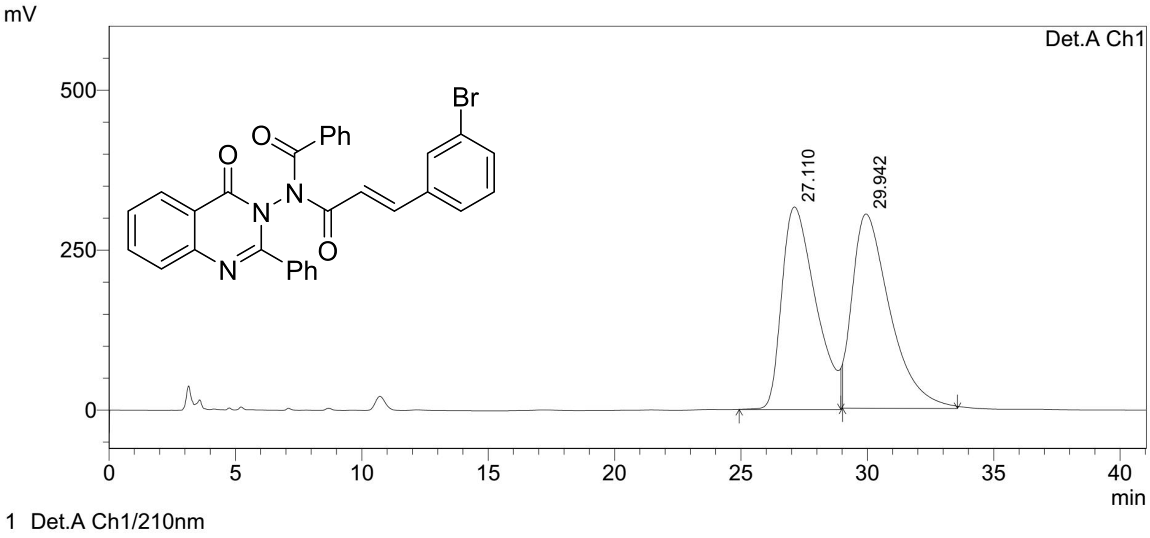

PeakTable

Detector A Ch1 210nm

Peak#	Ret. Time	Area	Height	Area %	Height %
1	27.110	29632889	316813	48.428	51.065
2	29.942	31556306	303603	51.572	48.935
Total		61189195	620415	100.000	100.000

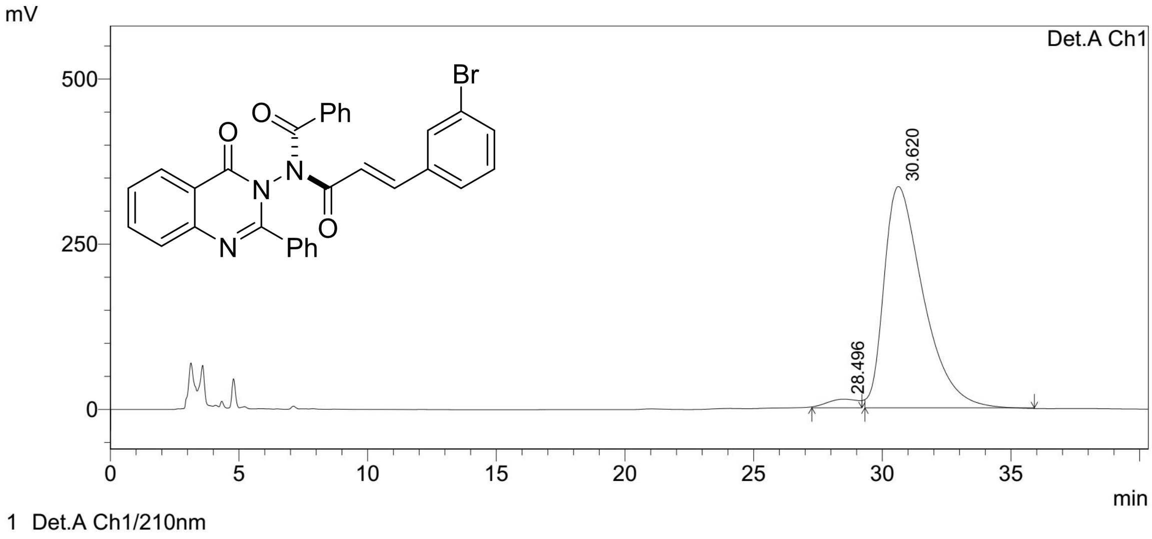

PeakTable

Detector A Ch1 210nm

Peak#	Ret. Time	Area	Height	Area %	Height %
1	28.496	1096302	13063	2.993	3.754
2	30.620	35533008	334949	97.007	96.246
Total		36629310	348012	100.000	100.000

($R_{a,cis,cis}$)-(*E*)-*N*-(3-(3-fluorophenyl)acryloyl)-*N*-(4-oxo-2-phenylquinazolin-3(4*H*)-yl)benzamide (3ad)

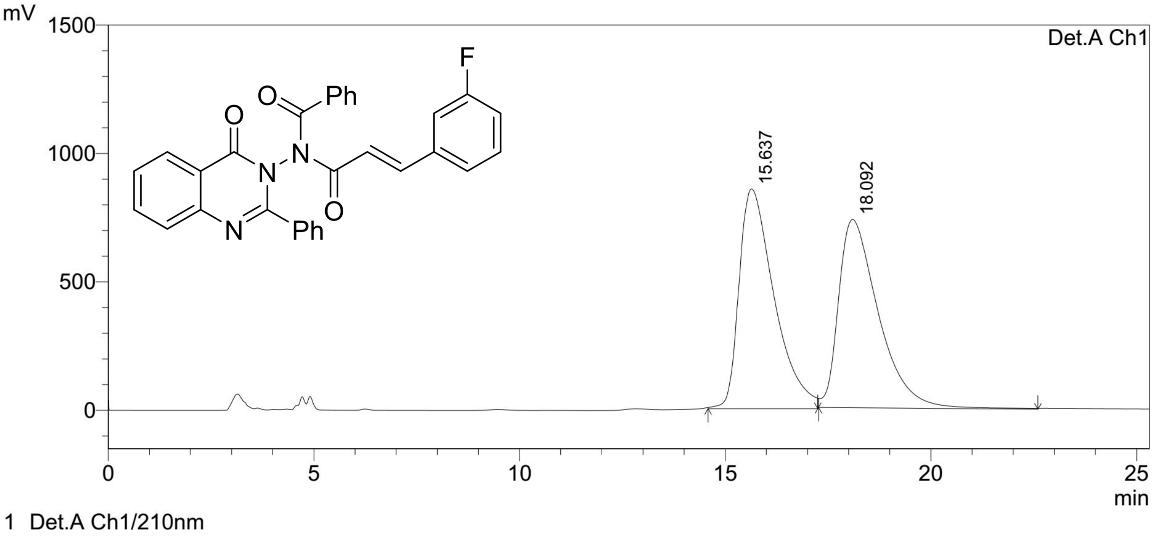

PeakTable

Detector A Ch1 210nm

Peak#	Ret. Time	Area	Height	Area %	Height %
1	15.637	50654396	855361	50.474	53.843
2	18.092	49703960	733261	49.526	46.157
Total		100358356	1588622	100.000	100.000

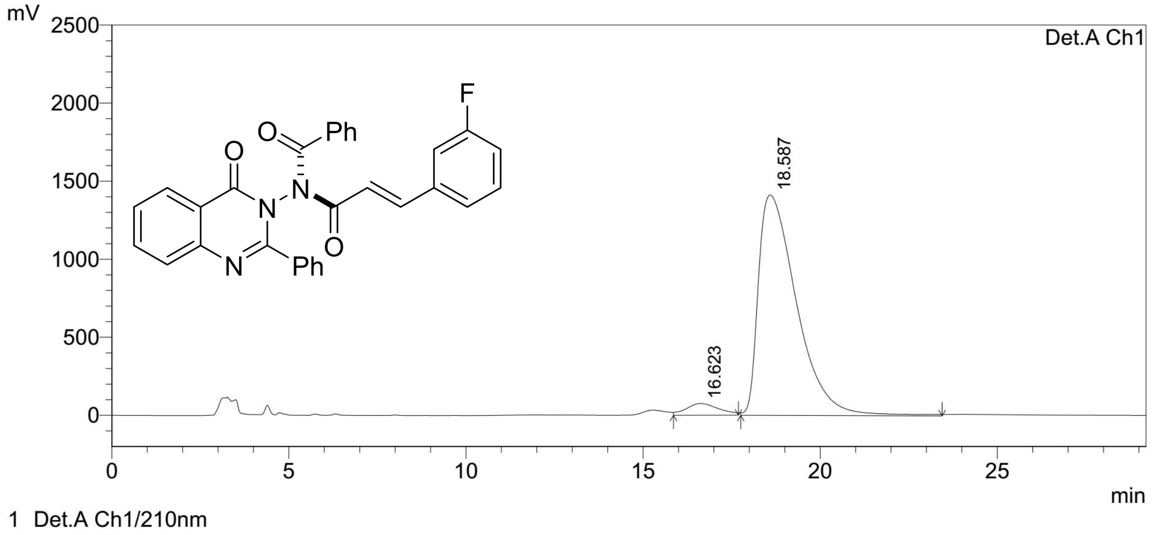

PeakTable

Detector A Ch1 210nm

Peak#	Ret. Time	Area	Height	Area %	Height %
1	16.623	4896899	75900	4.410	5.097
2	18.587	106135961	1413082	95.590	94.903
Total		111032860	1488982	100.000	100.000

(*R*$_{a,cis,cis}$)-(*E*)-*N*-(4-oxo-2-phenylquinazolin-3(4*H*)-yl)-*N*-(3-(o-tolyl)acryloyl) benzamide (3ae)

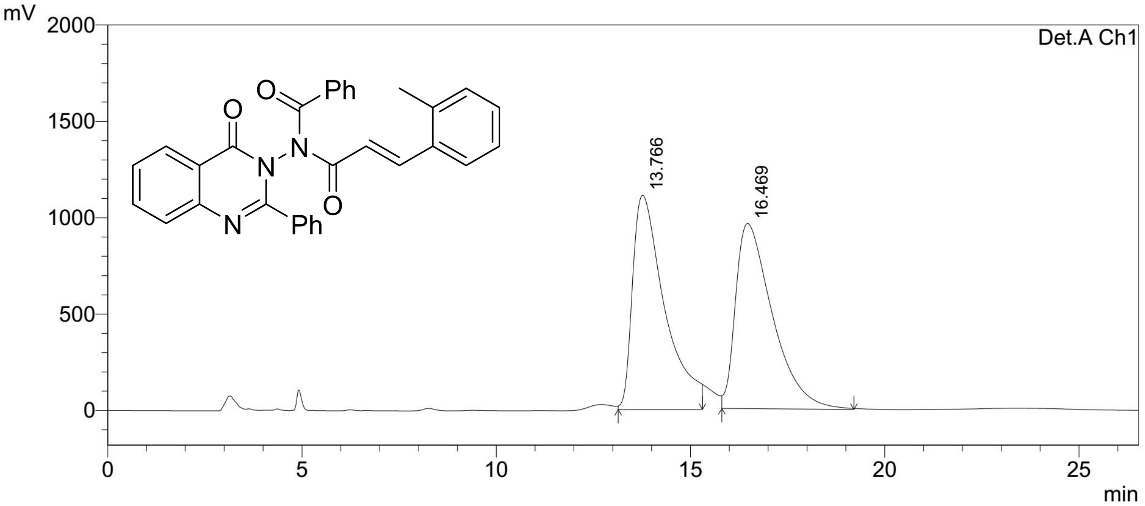

1 Det.A Ch1/210nm

PeakTable

Detector A Ch1 210nm

Peak#	Ret. Time	Area	Height	Area %	Height %
1	13.766	63695441	1111748	50.037	53.650
2	16.469	63602152	960469	49.963	46.350
Total		127297593	2072217	100.000	100.000

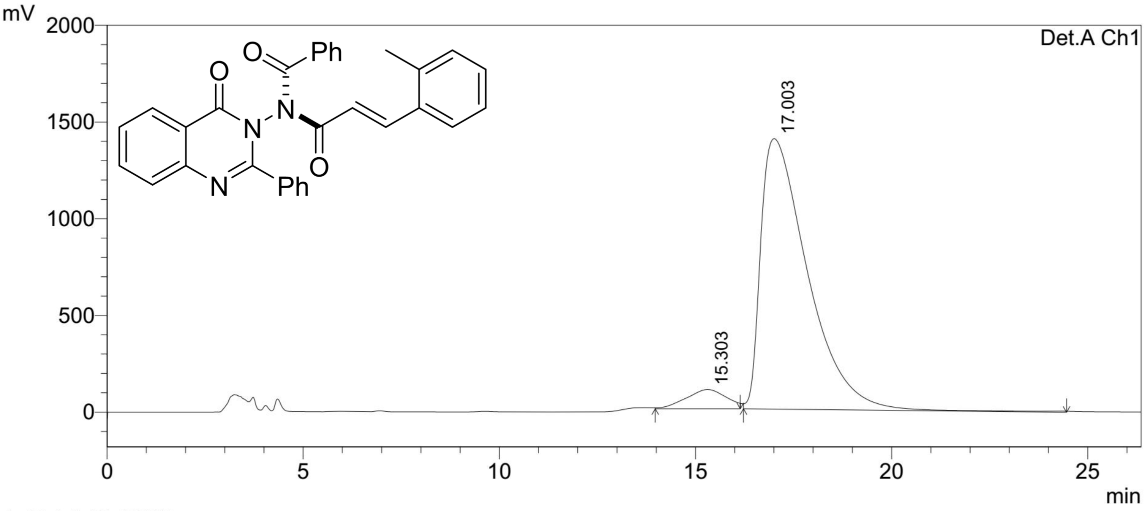

1 Det.A Ch1/210nm

PeakTable

Detector A Ch1 210nm

Peak#	Ret. Time	Area	Height	Area %	Height %
1	15.303	7072572	99500	5.703	6.643
2	17.003	116936335	1398233	94.297	93.357
Total		124008906	1497733	100.000	100.000

($R_{a,cis,cis}$)-(*E*)-*N*-(3-(naphthalen-1-yl)acryloyl)-*N*-(4-oxo-2-phenylquinazolin-3(4*H*)-yl)benzamide (3af)

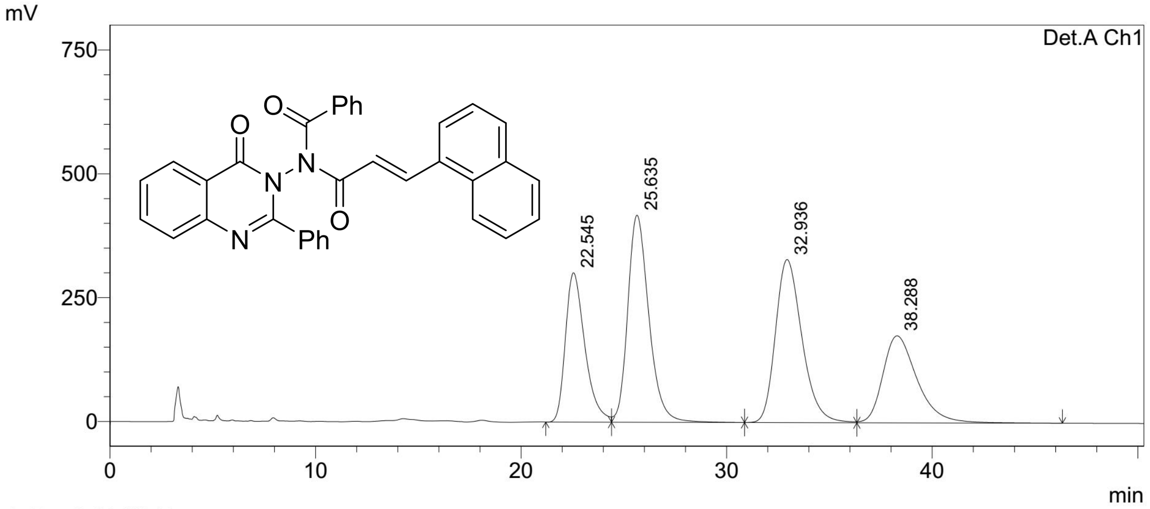

1 Det.A Ch1/210nm

PeakTable

Detector A Ch1 210nm

Peak#	Ret. Time	Area	Height	Area %	Height %
1	22.545	19917598	300876	19.990	24.611
2	25.635	29880802	417753	29.989	34.171
3	32.936	29487219	328503	29.594	26.871
4	38.288	20353981	175390	20.428	14.347
Total		99639599	1222522	100.000	100.000

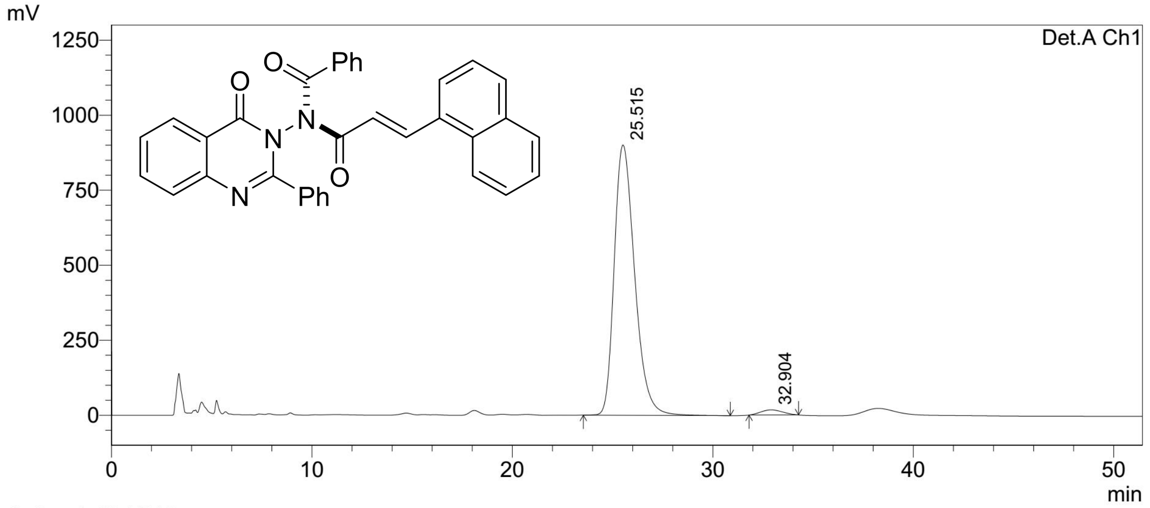

1 Det.A Ch1/210nm

PeakTable

Detector A Ch1 210nm

Peak#	Ret. Time	Area	Height	Area %	Height %
1	25.515	63884186	900148	98.009	98.133
2	32.904	1297953	17127	1.991	1.867
Total		65182139	917275	100.000	100.000

($R_{a,cis,cis}$)-(*E*)-*N*-(4-oxo-2-phenylquinazolin-3(4*H*)-yl)-*N*-(3-(thiophen-2-yl)acryloyl)benzamide (3ag)

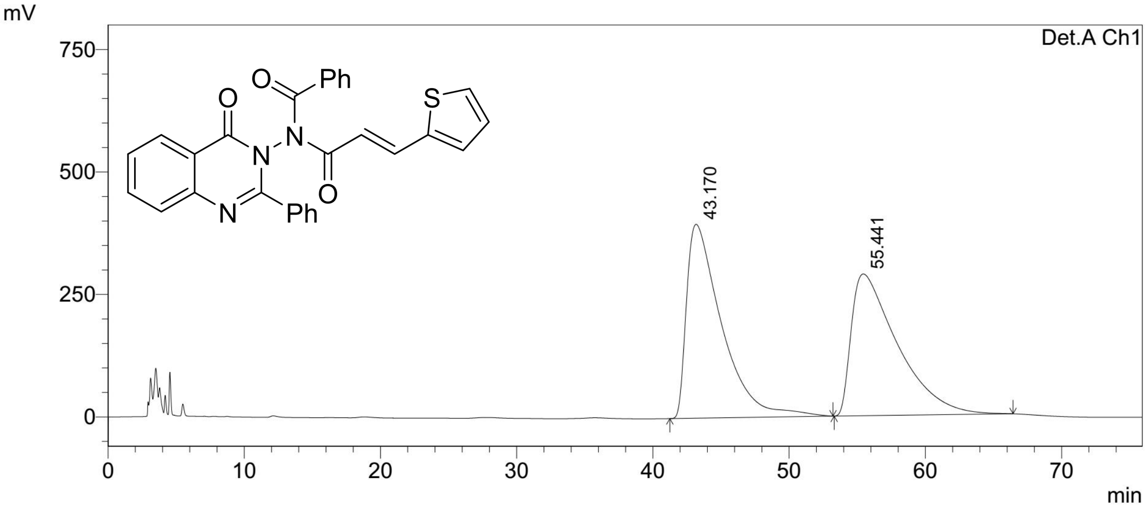

1 Det.A Ch1/210nm

PeakTable

Detector A Ch1 210nm

Peak#	Ret. Time	Area	Height	Area %	Height %
1	43.170	70980859	395830	50.973	57.771
2	55.441	68272262	289336	49.027	42.229
Total		139253121	685166	100.000	100.000

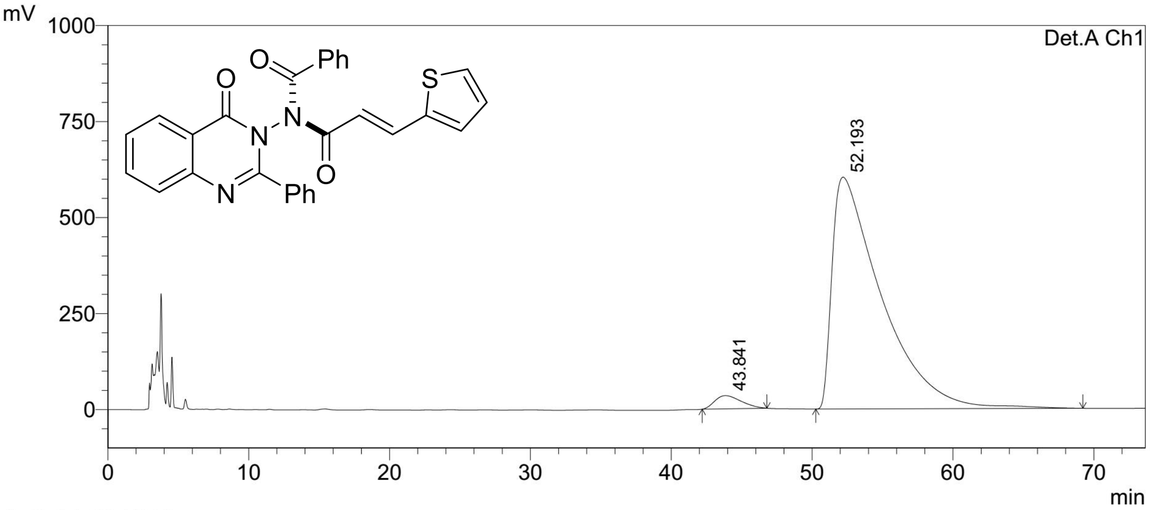

1 Det.A Ch1/210nm

PeakTable

Detector A Ch1 210nm

Peak#	Ret. Time	Area	Height	Area %	Height %
1	43.841	4487912	34422	2.961	5.395
2	52.193	147086396	603648	97.039	94.605
Total		151574309	638069	100.000	100.000

($R_{a,cis,cis}$)-*N*-(4-oxo-2-phenylquinazolin-3(4*H*)-yl)-*N*-(3-phenylpropanoyl)benzamide (3ah)

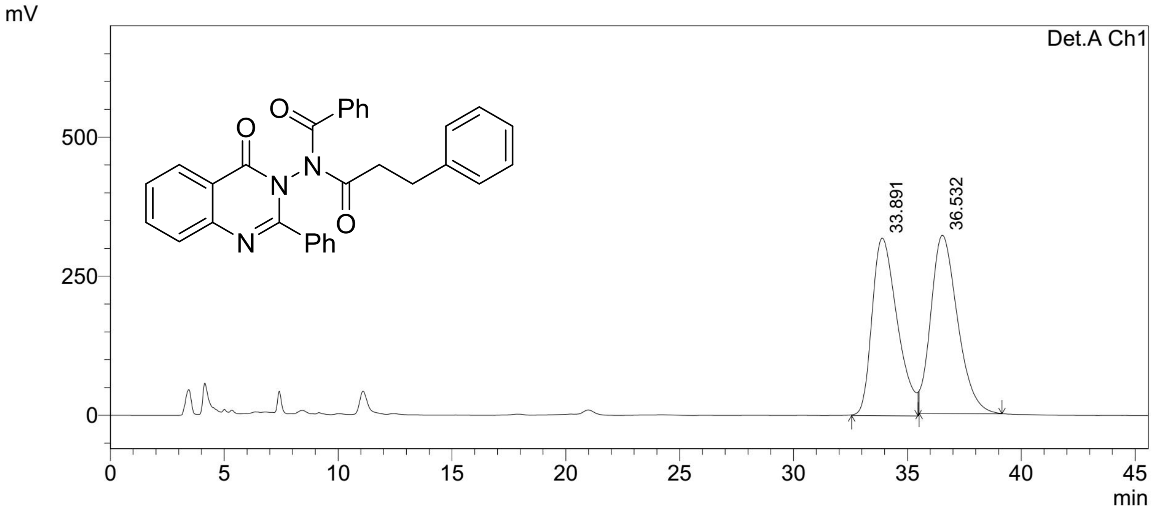

1 Det.A Ch1/210nm

PeakTable

Detector A Ch1 210nm

Peak#	Ret. Time	Area	Height	Area %	Height %
1	33.891	24658262	319478	48.582	49.943
2	36.532	26097982	320206	51.418	50.057
Total		50756244	639684	100.000	100.000

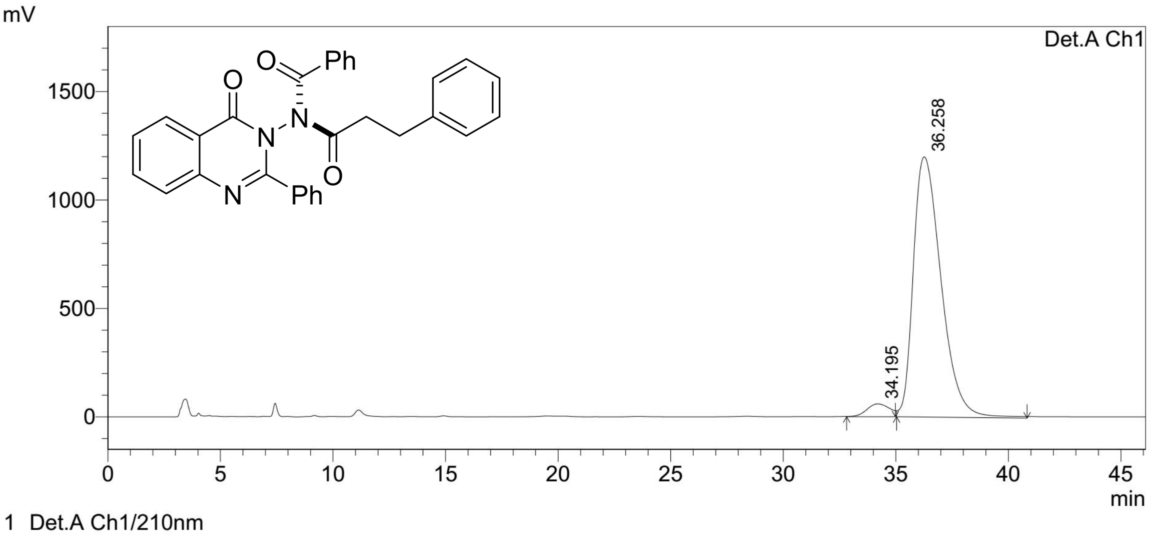

1 Det.A Ch1/210nm

PeakTable

Detector A Ch1 210nm

Peak#	Ret. Time	Area	Height	Area %	Height %
1	34.195	4140704	59430	3.864	4.719
2	36.258	103009123	1199849	96.136	95.281
Total		107149828	1259279	100.000	100.000

($R_{a,cis,cis}$)-*N*-acetyl-*N*-(4-oxo-2-phenylquinazolin-3(4*H*)-yl)benzamide (3ai)

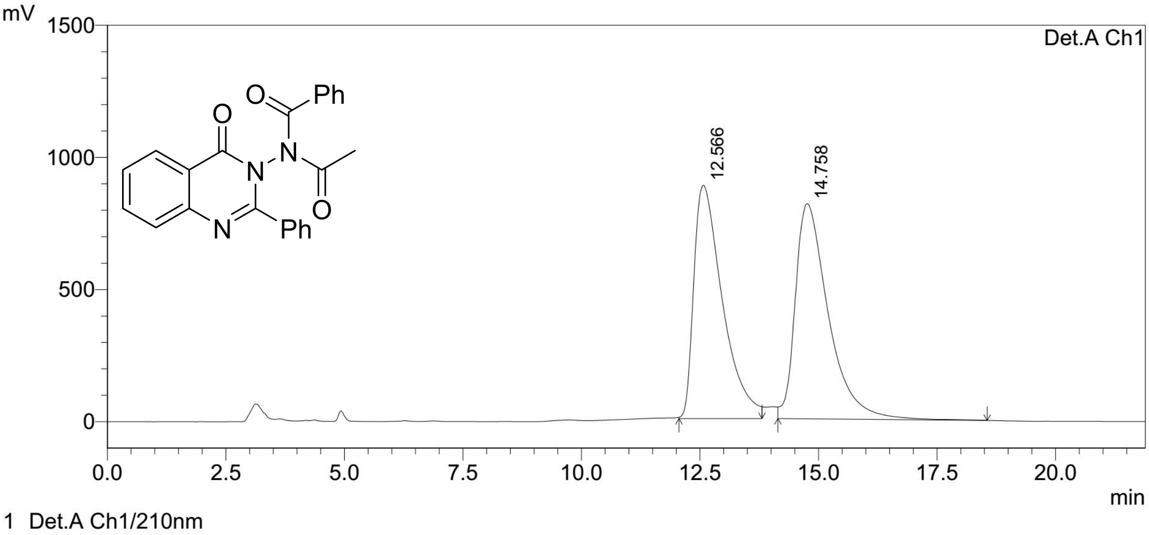

PeakTable

Detector A Ch1 210nm

Peak#	Ret. Time	Area	Height	Area %	Height %
1	12.566	37099706	882977	48.360	52.032
2	14.758	39616209	814018	51.640	47.968
Total		76715915	1696996	100.000	100.000

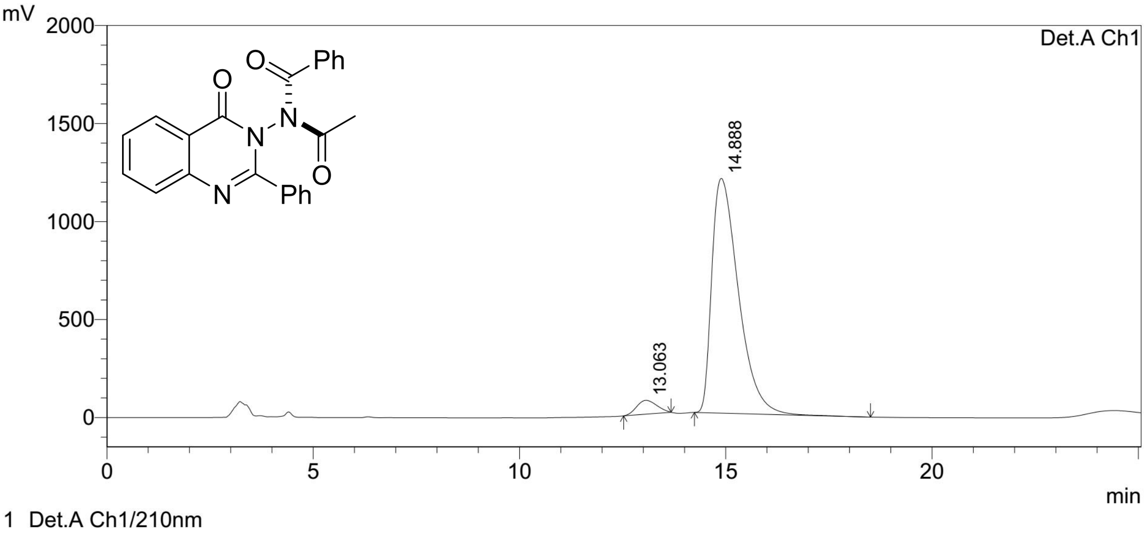

PeakTable

Detector A Ch1 210nm

Peak#	Ret. Time	Area	Height	Area %	Height %
1	13.063	2383426	70449	4.242	5.556
2	14.888	53807461	1197514	95.758	94.444
Total		56190887	1267963	100.000	100.000

($R_{a,cis,cis}$)- *N*-(4-oxo-2-phenylquinazolin-3(4*H*)-yl)-*N*-propionylbenzamide (3aj)

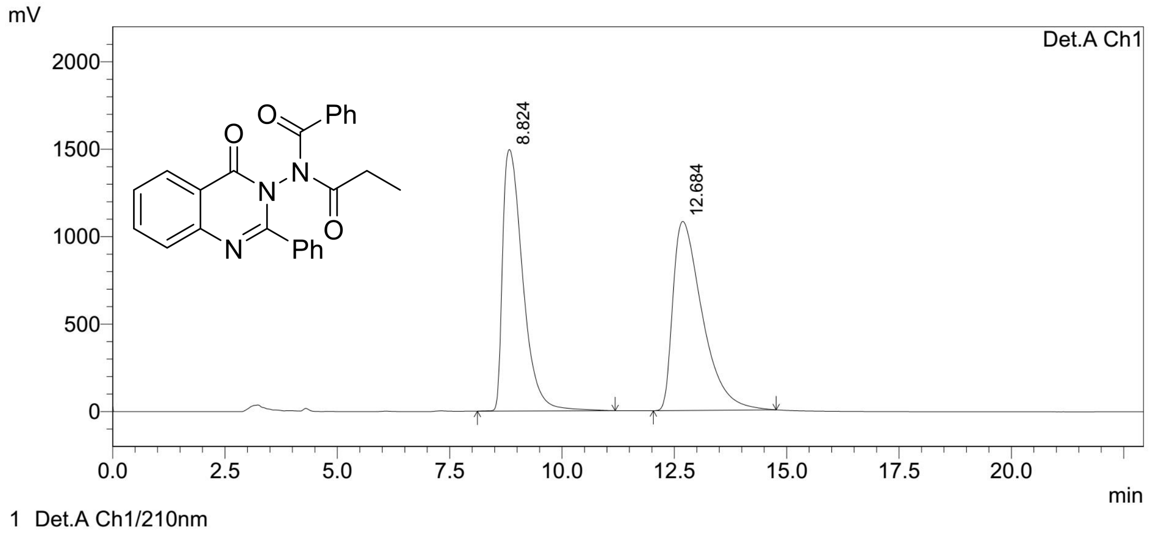

PeakTable

Detector A Ch1 210nm

Peak#	Ret. Time	Area	Height	Area %	Height %
1	8.824	46651700	1495714	48.604	58.048
2	12.684	49332519	1080966	51.396	41.952
Total		95984219	2576680	100.000	100.000

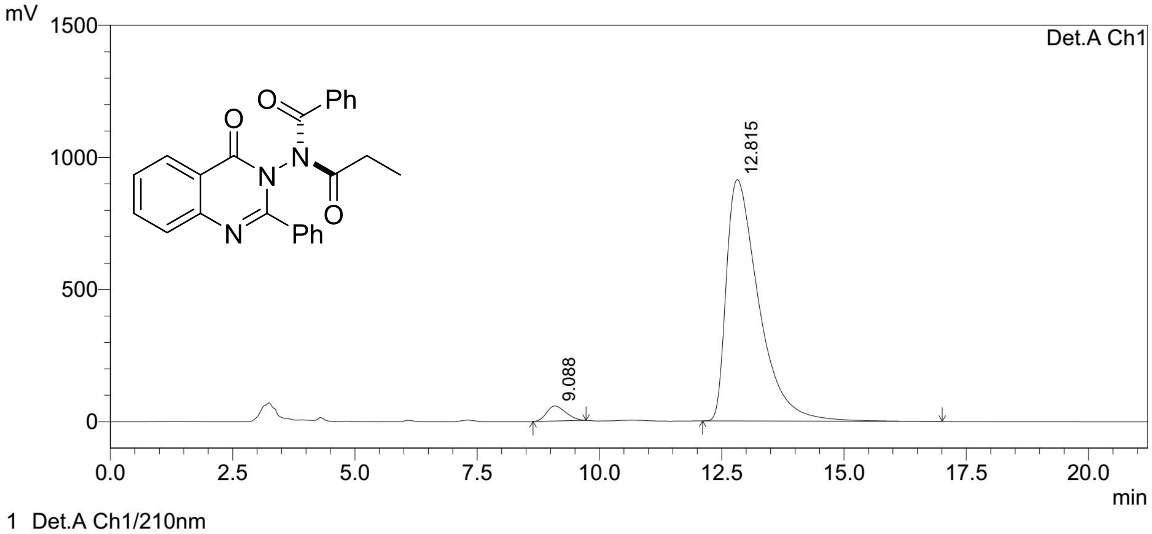

PeakTable

Detector A Ch1 210nm

Peak#	Ret. Time	Area	Height	Area %	Height %
1	9.088	1553576	57160	3.557	5.890
2	12.815	42117559	913318	96.443	94.110
Total		43671135	970478	100.000	100.000

($R_{a,cis,cis}$)*N*-benzoyl-*N*-(4-oxo-2-phenylquinazolin-3(4*H*)-yl)-4-phenyl-4,5-dihydro-3*H*-3λ^3,4λ^3-pyrazole-3-carboxamide (4)

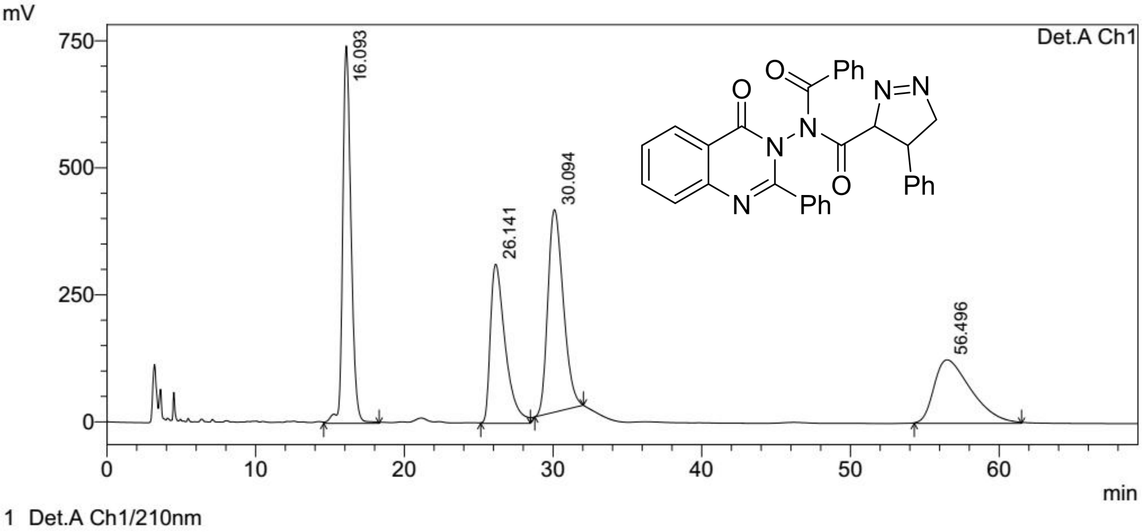

PeakTable

Detector A Ch1 210nm

Peak#	Ret. Time	Area	Height	Area %	Height %
1	16.093	28848836	742672	28.517	47.020
2	26.141	21855164	313218	21.604	19.831
3	30.094	28807258	398698	28.476	25.242
4	56.496	21652959	124889	21.404	7.907
Total		101164218	1579476	100.000	100.000

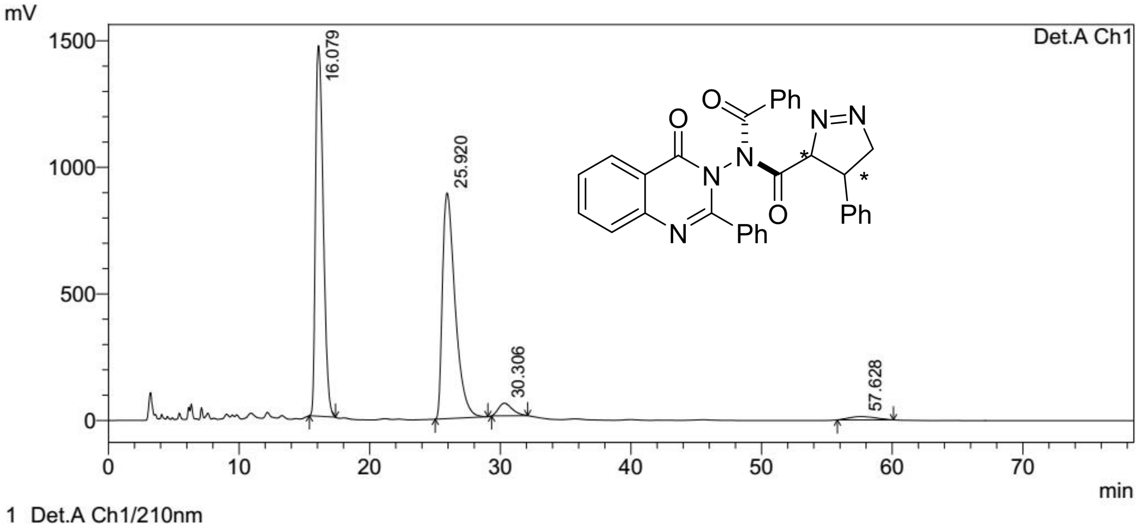

PeakTable

Detector A Ch1 210nm

Peak#	Ret. Time	Area	Height	Area %	Height %
1	16.079	60086412	1464372	47.843	60.545
2	25.920	60030568	891591	47.798	36.863
3	30.306	3660863	49675	2.915	2.054
4	57.628	1813144	12996	1.444	0.537
Total		125590988	2418633	100.000	100.000

(*R*)-(2-methylpiperidin-1-yl)(phenyl)methanone (6a)

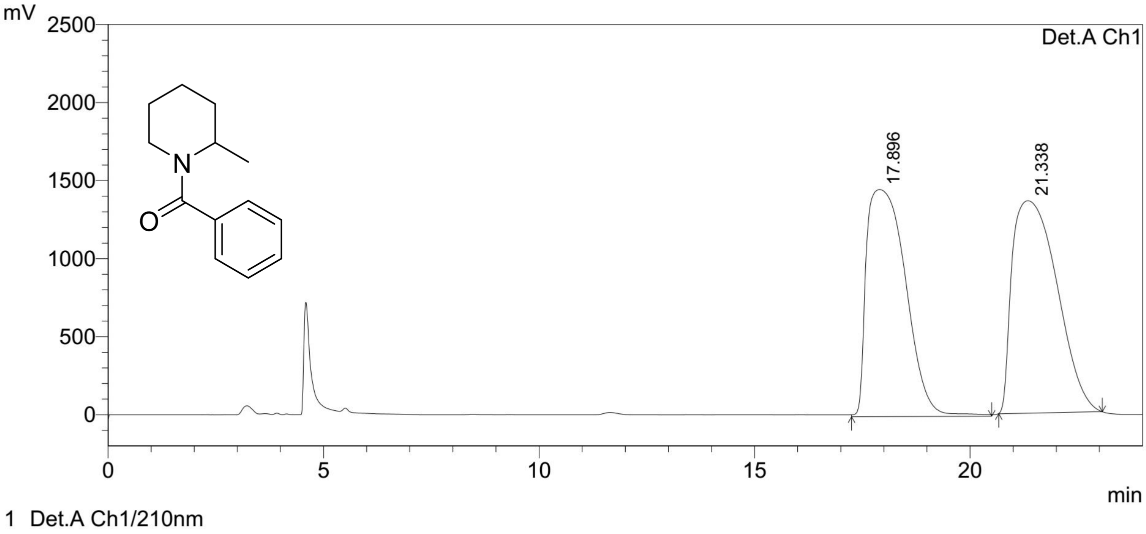

PeakTable

Detector A Ch1 210nm

Peak#	Ret. Time	Area	Height	Area %	Height %
1	17.896	92663582	1455179	48.408	51.675
2	21.338	98756738	1360821	51.592	48.325
Total		191420320	2816001	100.000	100.000

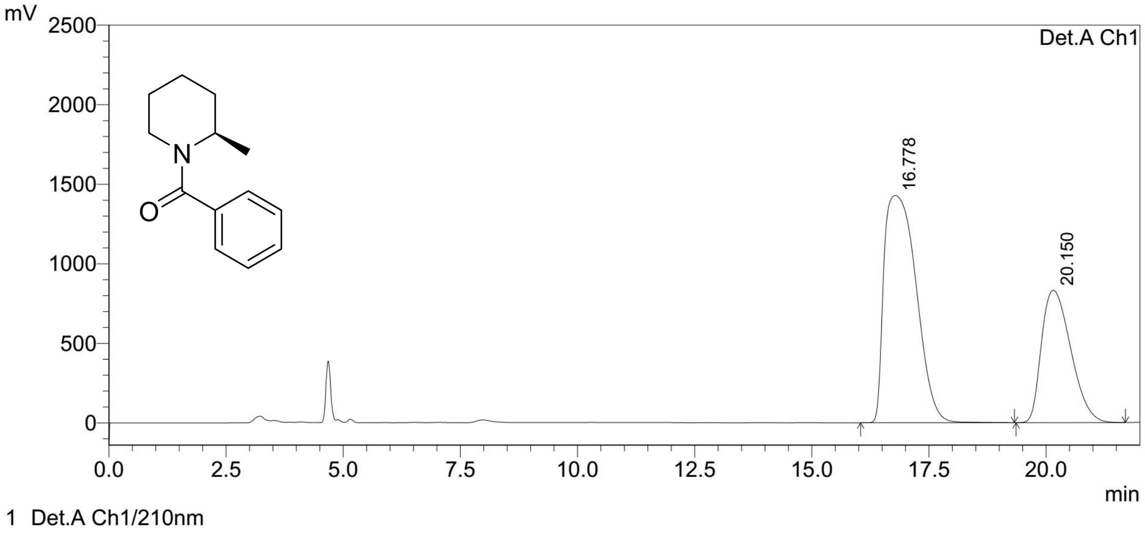

PeakTable

Detector A Ch1 210nm

Peak#	Ret. Time	Area	Height	Area %	Height %
1	16.778	68510197	1427969	65.413	63.200
2	20.150	36224337	831458	34.587	36.800
Total		104734535	2259427	100.000	100.000

(*S*)-(4-methoxyphenyl)(2-methylpiperidin-1-yl)methanone (6b)

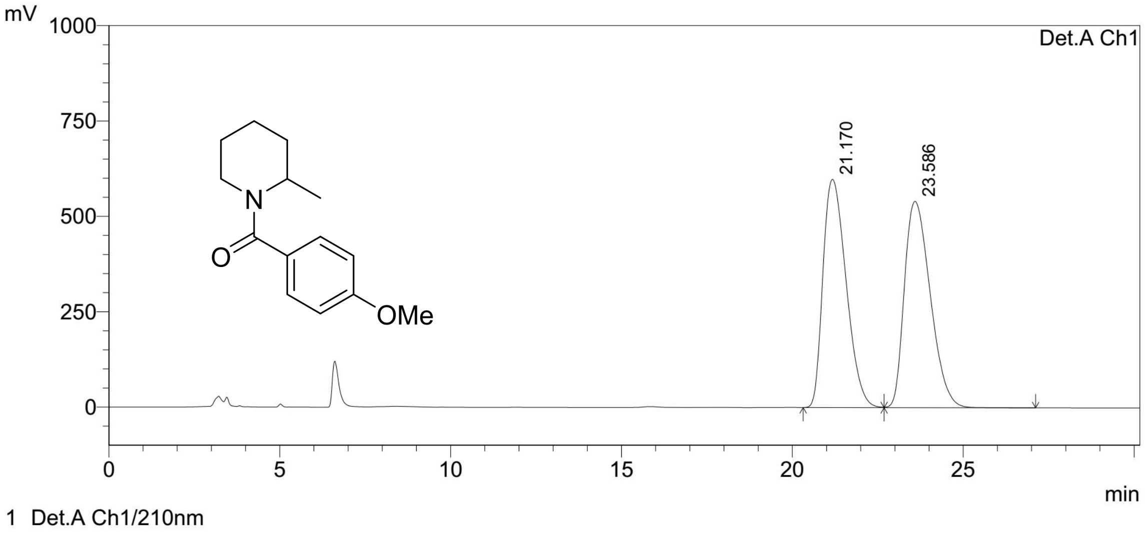

PeakTable

Detector A Ch1 210nm

Peak#	Ret. Time	Area	Height	Area %	Height %
1	21.170	28254240	598413	49.761	52.547
2	23.586	28525519	540402	50.239	47.453
Total		56779759	1138814	100.000	100.000

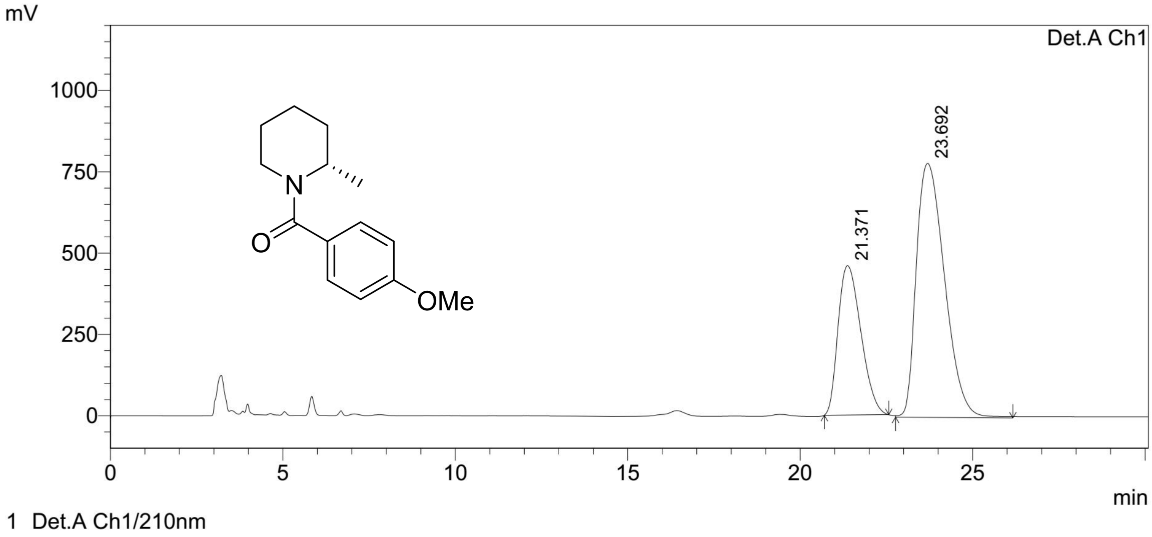

PeakTable

Detector A Ch1 210nm

Peak#	Ret. Time	Area	Height	Area %	Height %
1	21.371	21165894	459040	32.204	37.044
2	23.692	44559262	780131	67.796	62.956
Total		65725156	1239171	100.000	100.000

N-benzyl-*N*-(4-oxo-2-phenylquinazolin-3(4*H*)-yl)benzamide (8a)

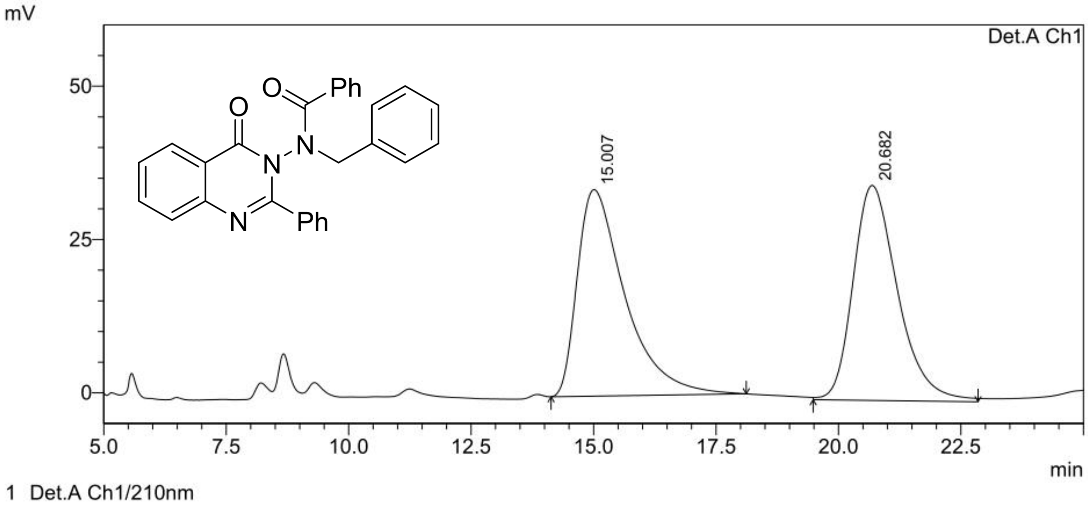

PeakTable

Detector A Ch1 210nm

Peak#	Ret. Time	Area	Height	Area %	Height %
1	15.007	2282290	33672	50.708	49.018
2	20.682	2218527	35021	49.292	50.982
Total		4500817	68693	100.000	100.000

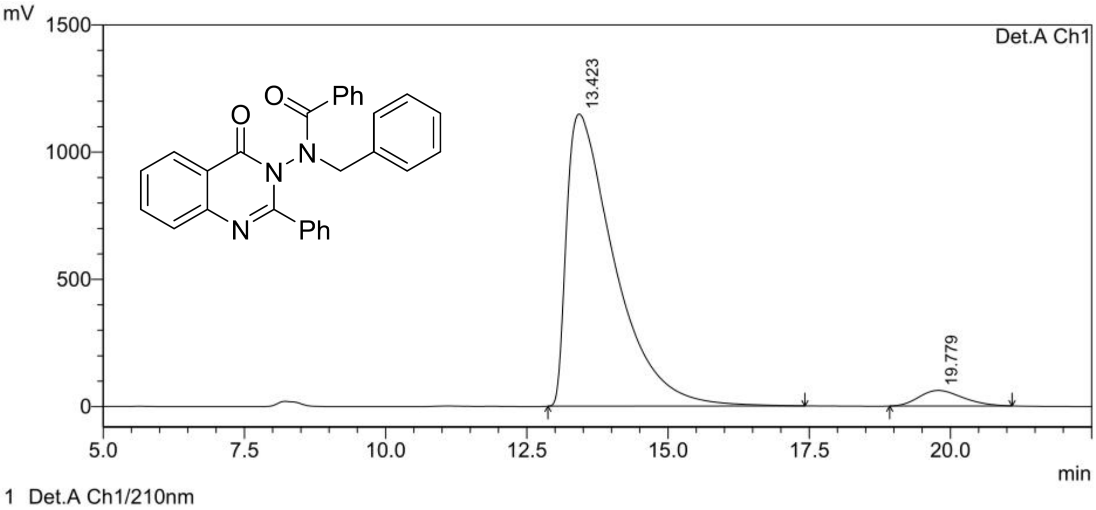

PeakTable

Detector A Ch1 210nm

Peak#	Ret. Time	Area	Height	Area %	Height %
1	13.423	67959937	1146818	95.353	94.927
2	19.779	3311683	61285	4.647	5.073
Total		71271621	1208104	100.000	100.000

Methyl 2-((*N*-(4-oxo-2-phenylquinazolin-3(4*H*)-yl)benzamido)methyl)acrylate (8b)

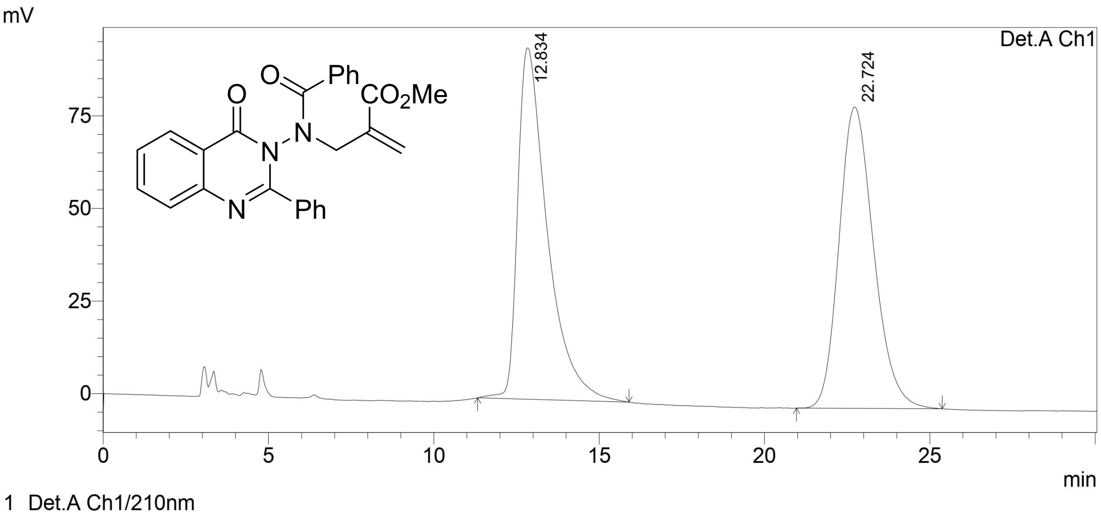

1 Det.A Ch1/210nm

PeakTable

Detector A Ch1 210nm

Peak#	Ret. Time	Area	Height	Area %	Height %
1	12.834	5882728	94842	49.800	53.829
2	22.724	5929894	81349	50.200	46.171
Total		11812622	176190	100.000	100.000

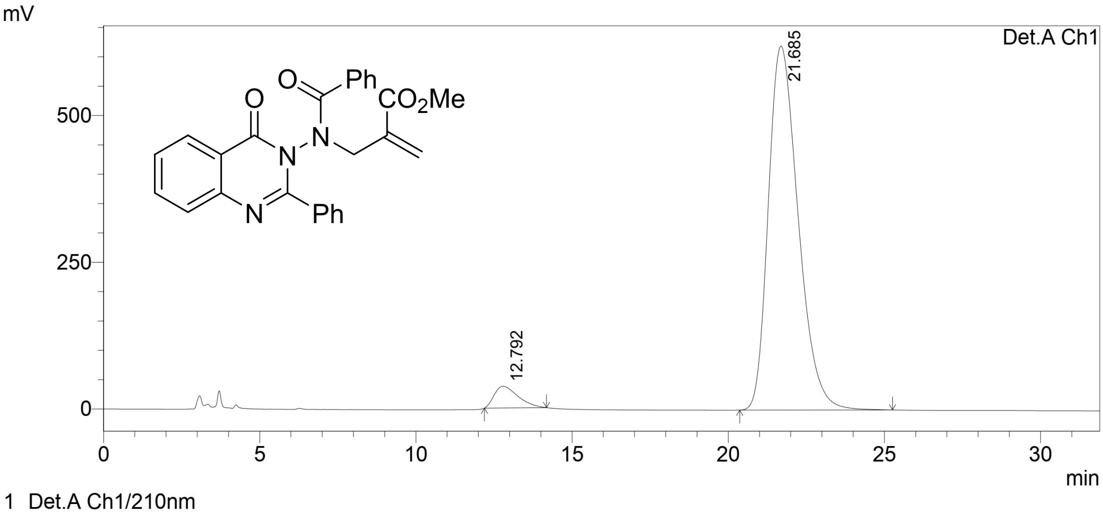

1 Det.A Ch1/210nm

PeakTable

Detector A Ch1 210nm

Peak#	Ret. Time	Area	Height	Area %	Height %
1	12.792	1958959	36955	4.531	5.625
2	21.685	41279190	620016	95.469	94.375
Total		43238148	656971	100.000	100.000

10. NMR Analysis

(*R*$_{a,cis,cis}$)-*N*-cinnamoyl-*N*-(4-oxo-2-phenylquinazolin-3(4*H*)-yl)benzamide (3aa)

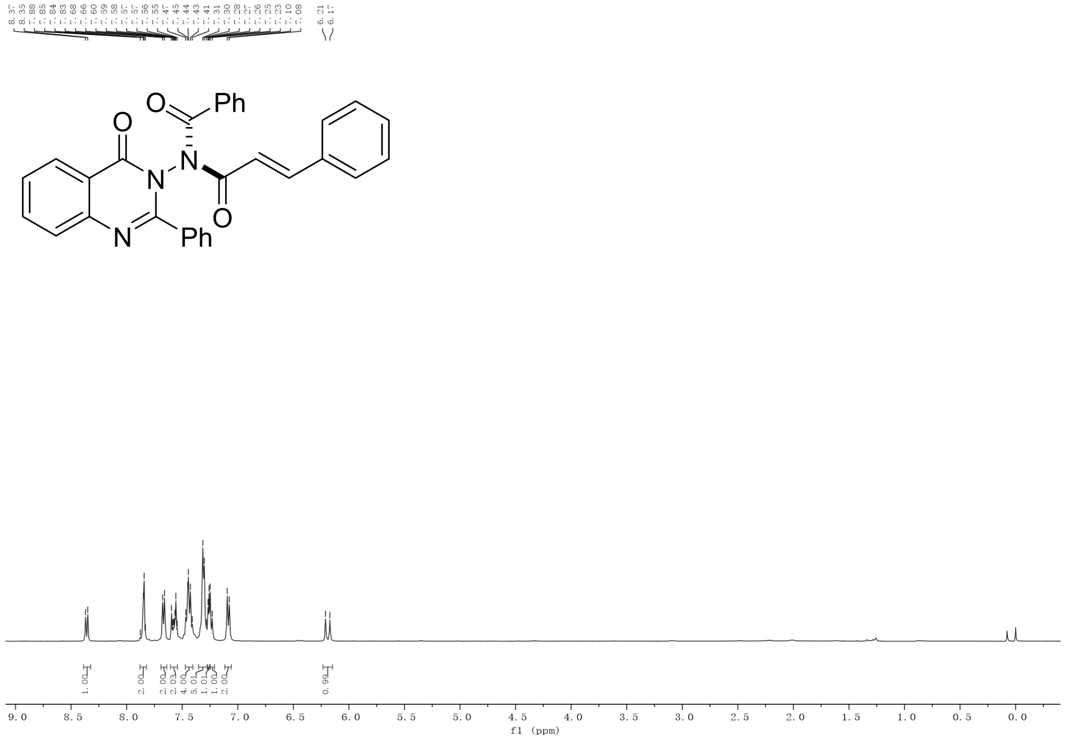

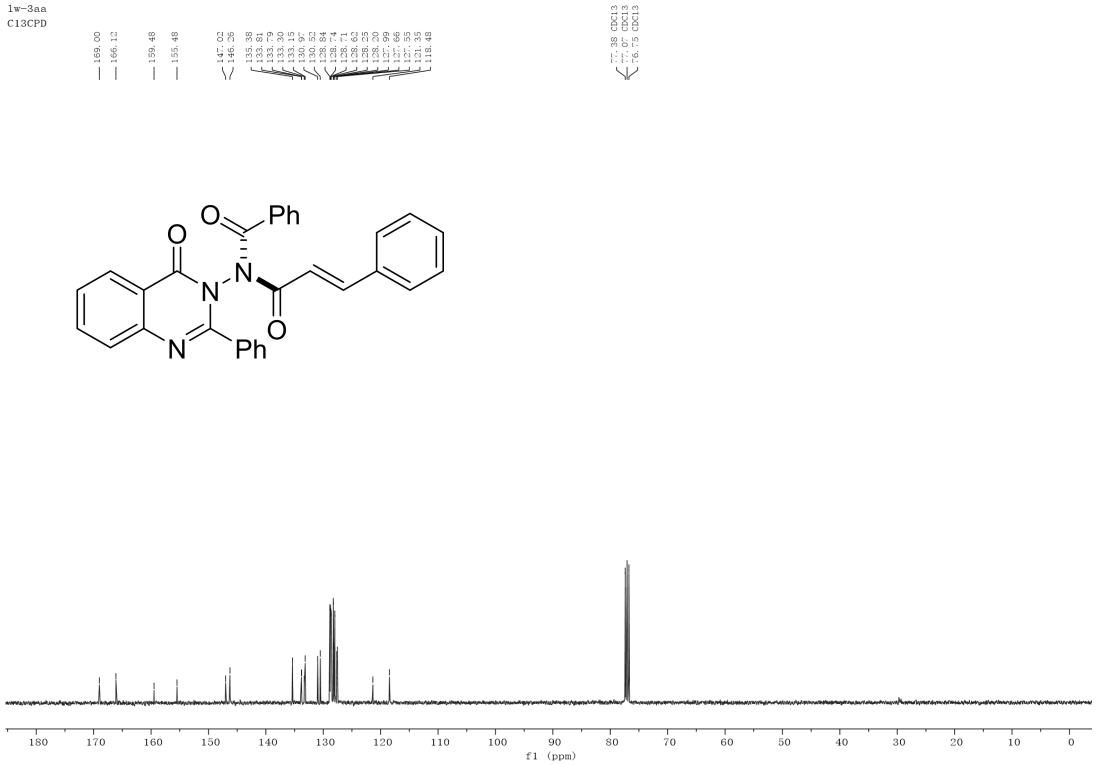

(*R*$_{a,cis,cis}$)-*N*-(5-chloro-4-oxo-2-phenylquinazolin-3(4*H*)-yl)-*N*-cinnamoylbenzamide (3ba)

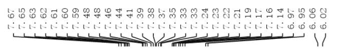

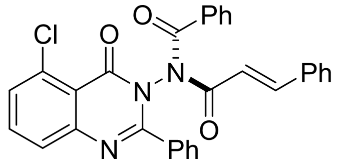

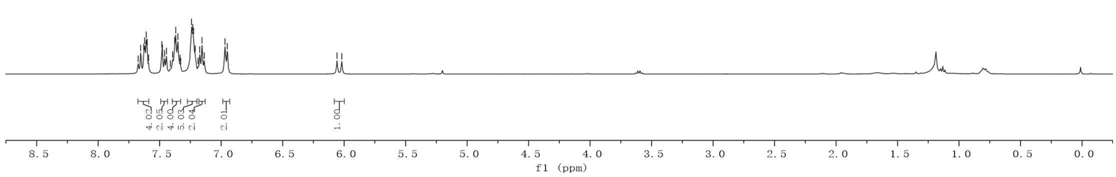

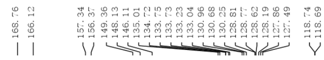

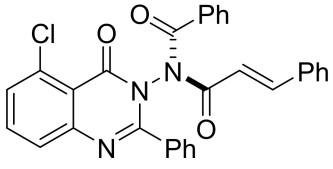

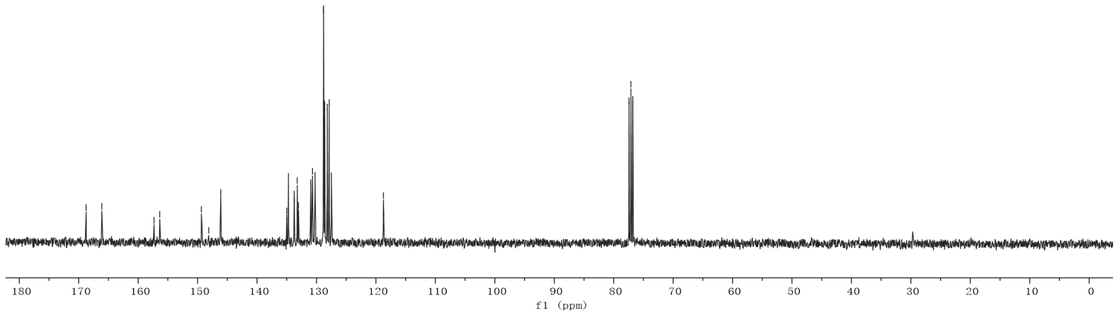

($R_{a,cis,cis}$)-*N*-cinnamoyl-*N*-(5-methyl-4-oxo-2-phenylquinazolin-3(4*H*)-yl)benzamide (3ca)

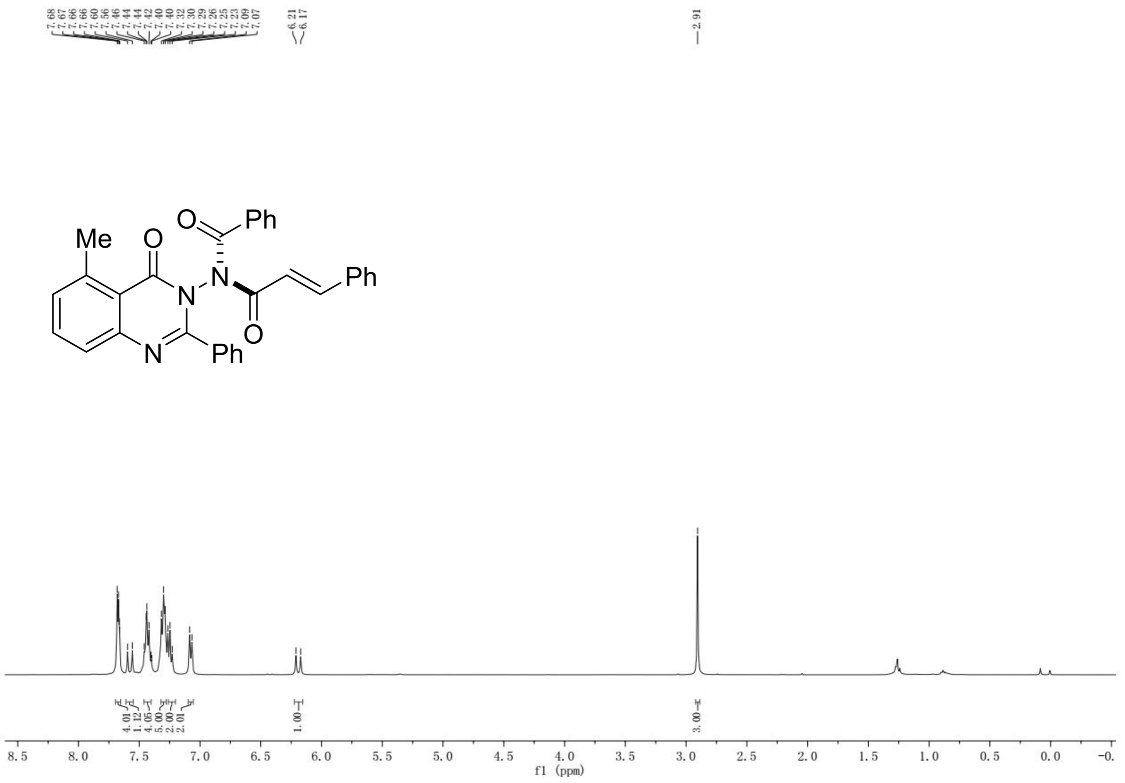

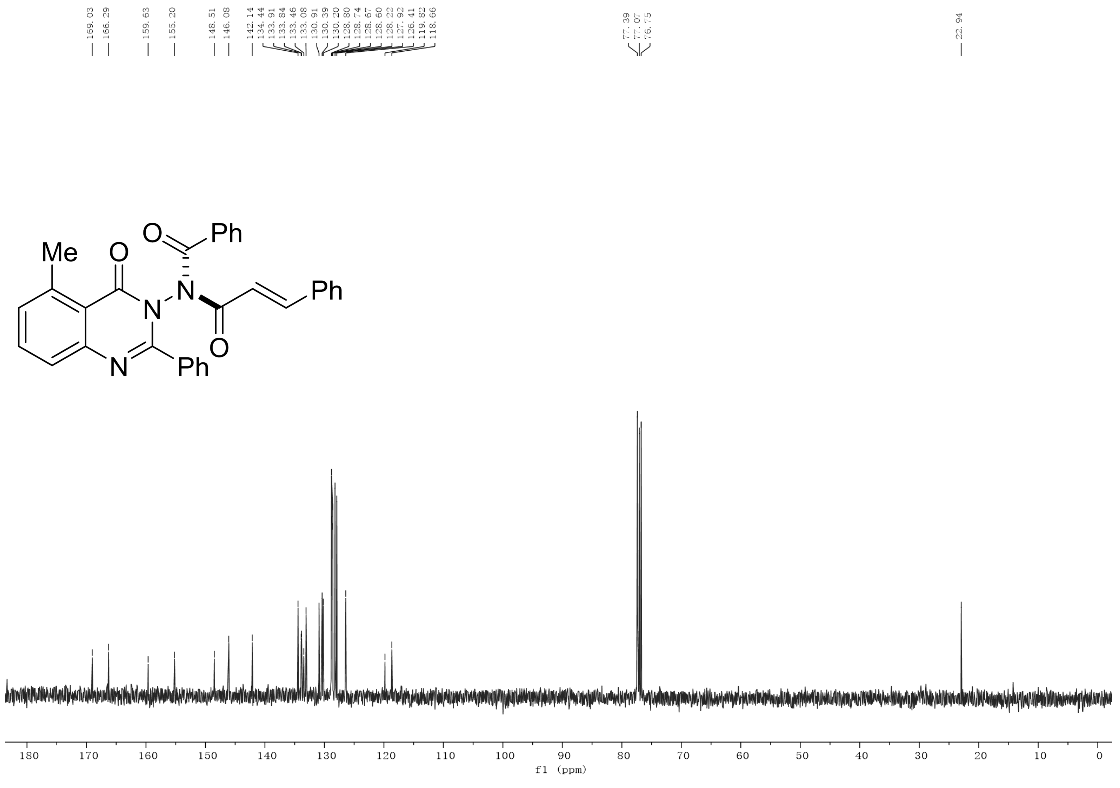

($R_{a,cis,cis}$)-*N*-cinnamoyl-*N*-(6-methoxy-4-oxo-2-phenylquinazolin-3(4*H*)-yl)benzamide (3da)

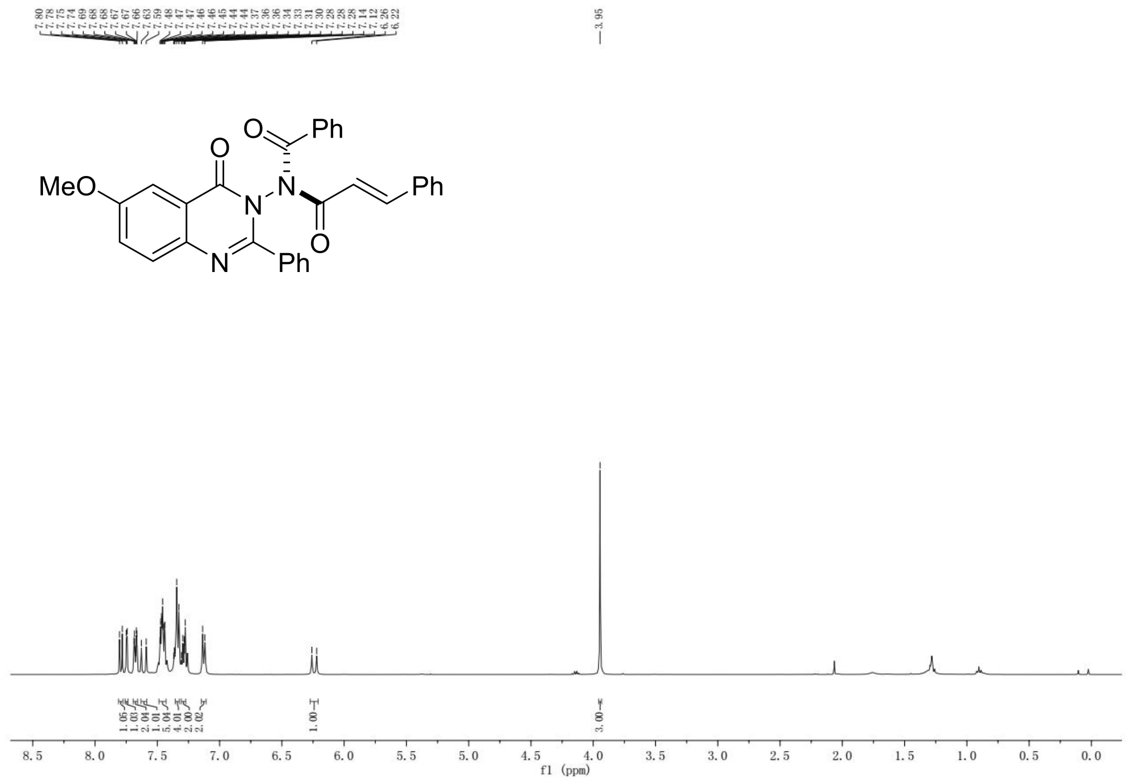

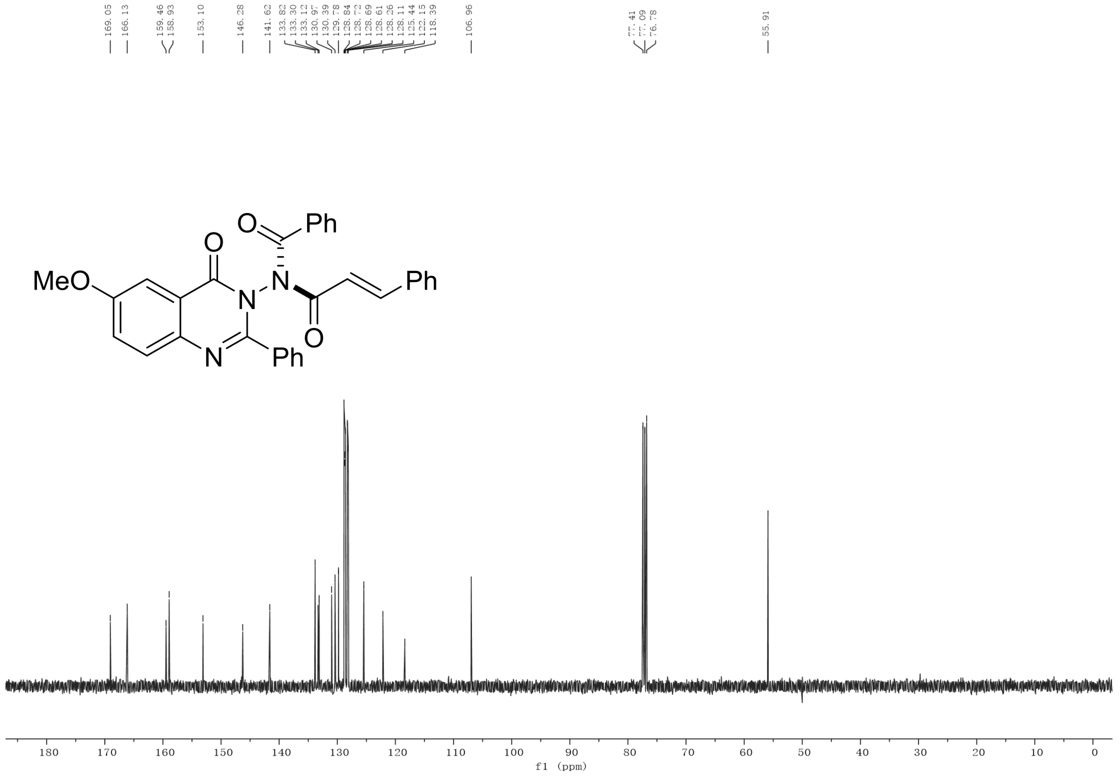

($R_{a,cis,cis}$)-*N*-cinnamoyl-*N*-(6-iodo-4-oxo-2-phenylquinazolin-3(4*H*)-yl)benzamide (3ea)

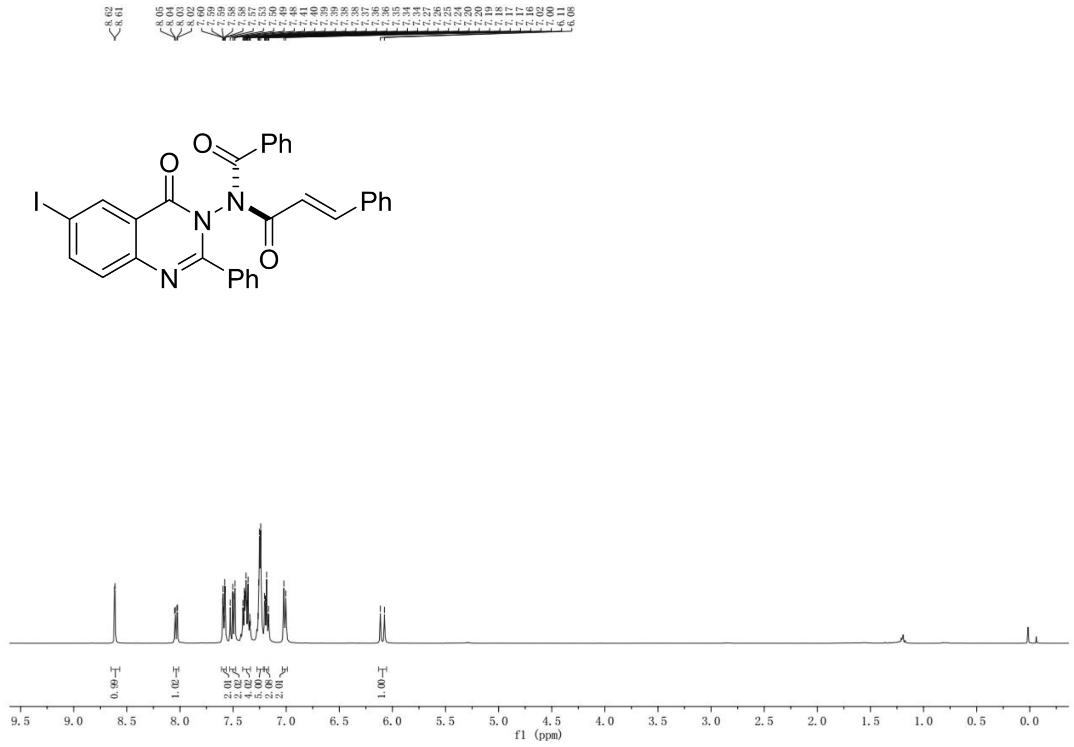

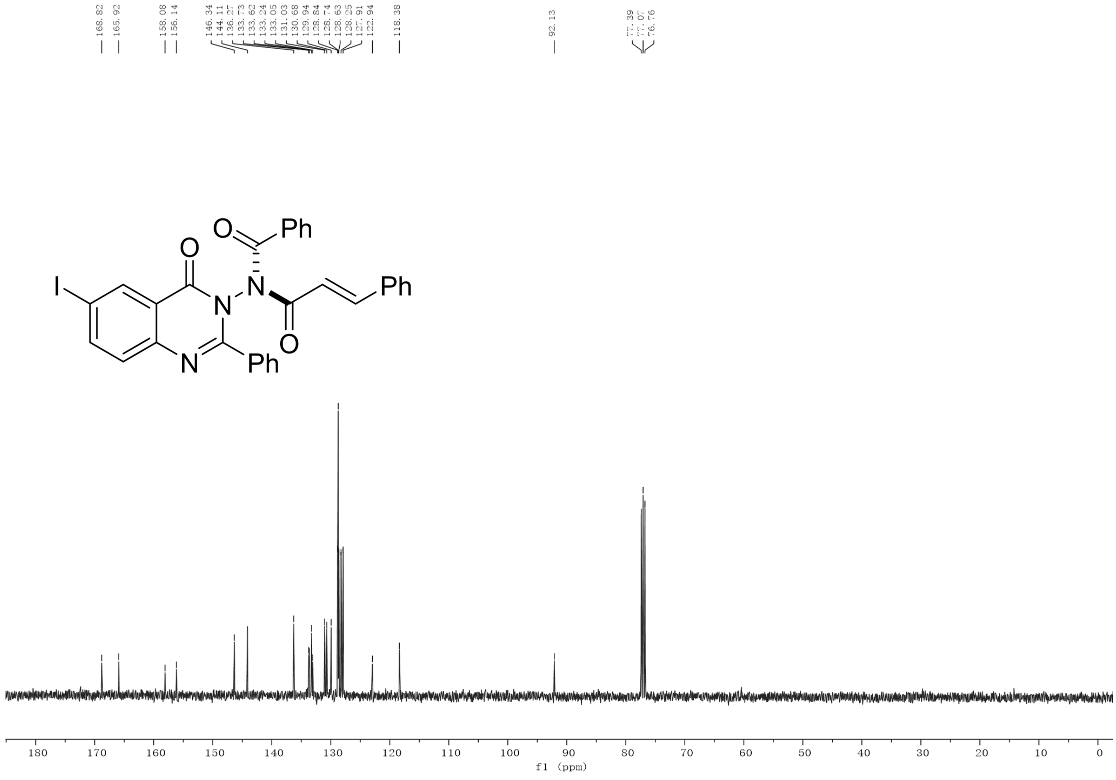

(*R*$_{a,cis,cis}$)-*N*-(7-bromo-4-oxo-2-phenylquinazolin-3(4*H*)-yl)-*N*-cinnamoylbenzamide (3fa)

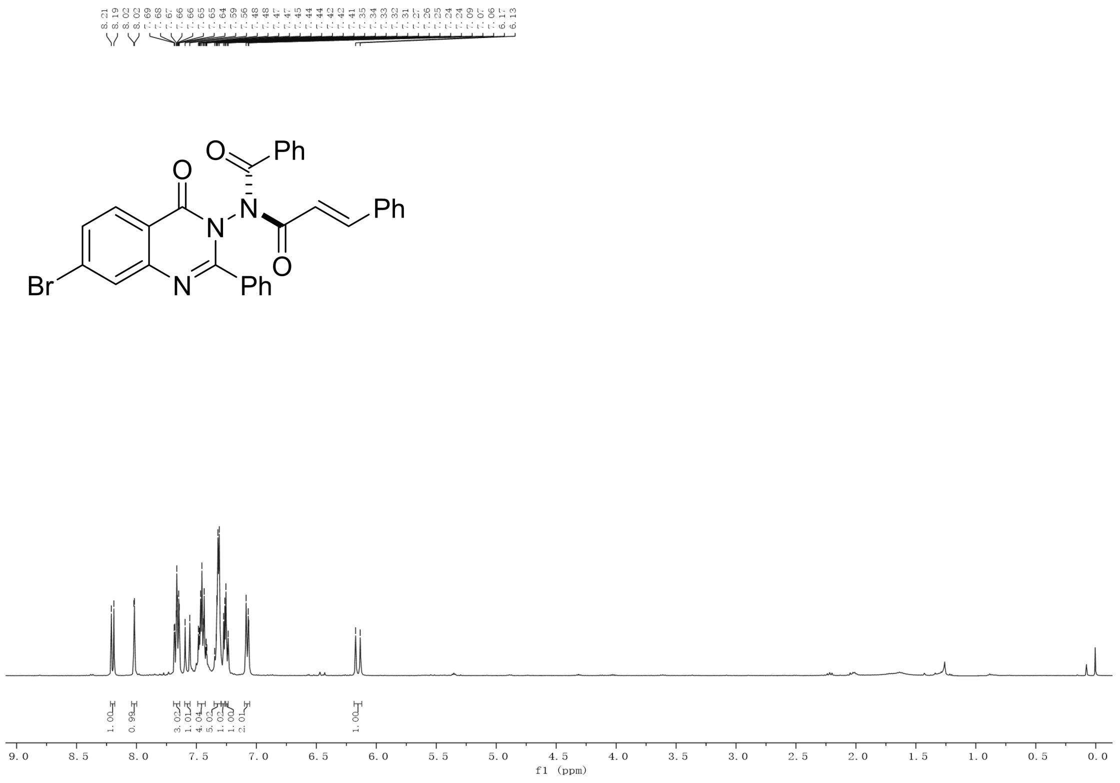

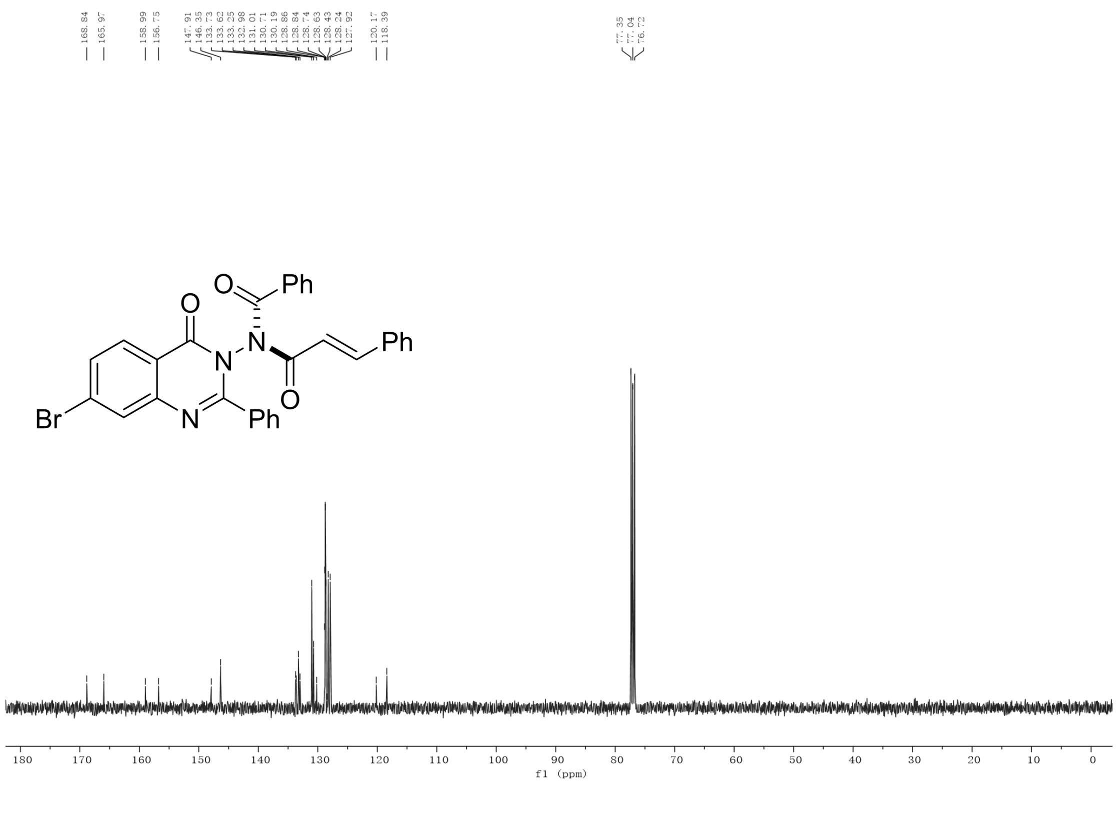

($R_{a,cis,cis}$)-*N*-(8-chloro-4-oxo-2-phenylquinazolin-3(4*H*)-yl)-*N*-cinnamoylbenzamide (3ga)

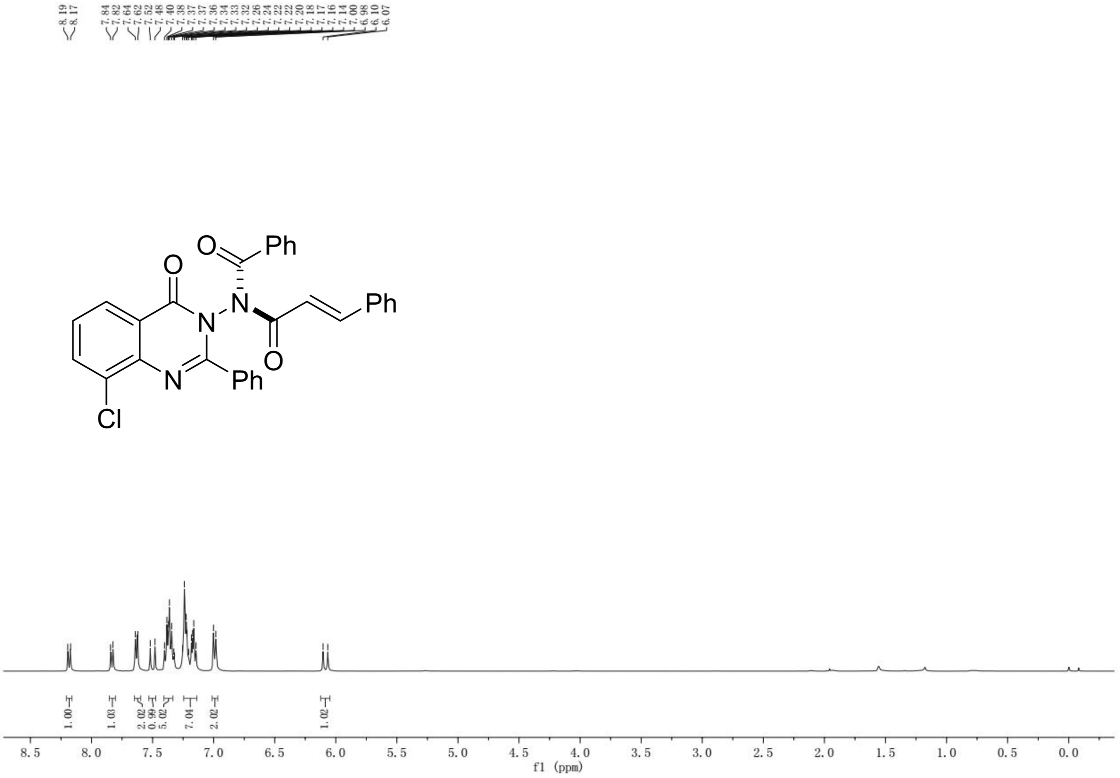

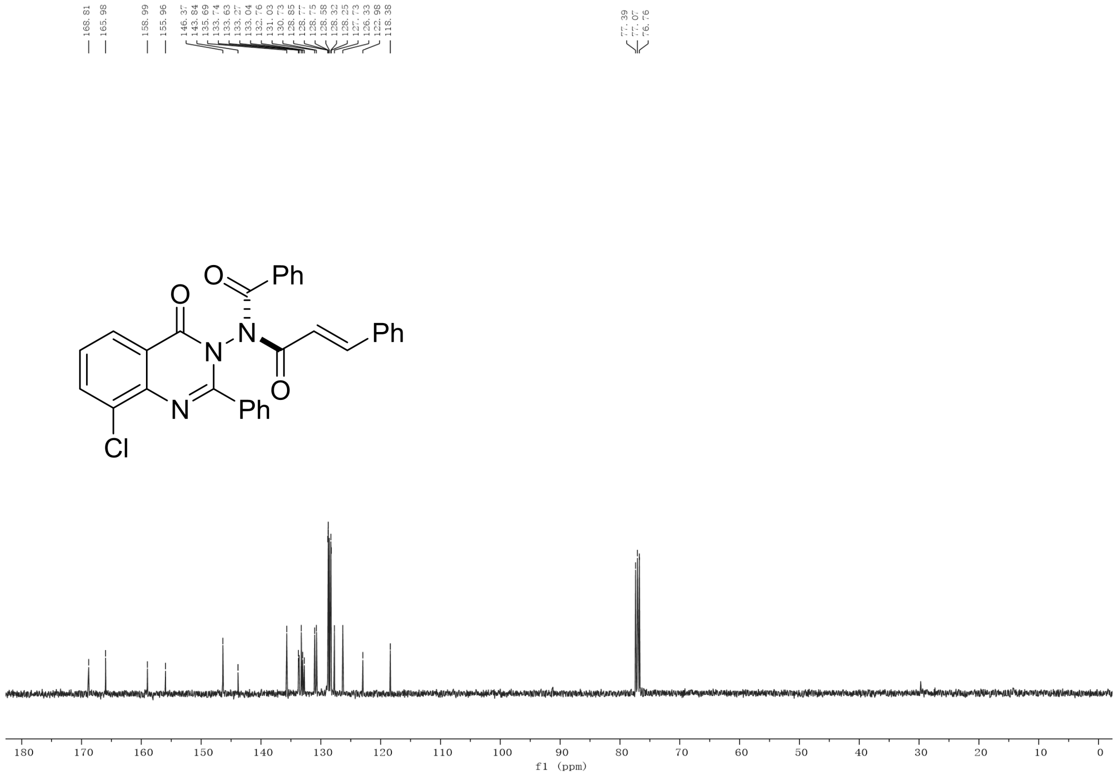

($R_{a,cis,cis}$)-4-chloro-*N*-cinnamoyl-*N*-(4-oxo-2-phenylquinazolin-3(4*H*)-yl)benzamide (3ha)

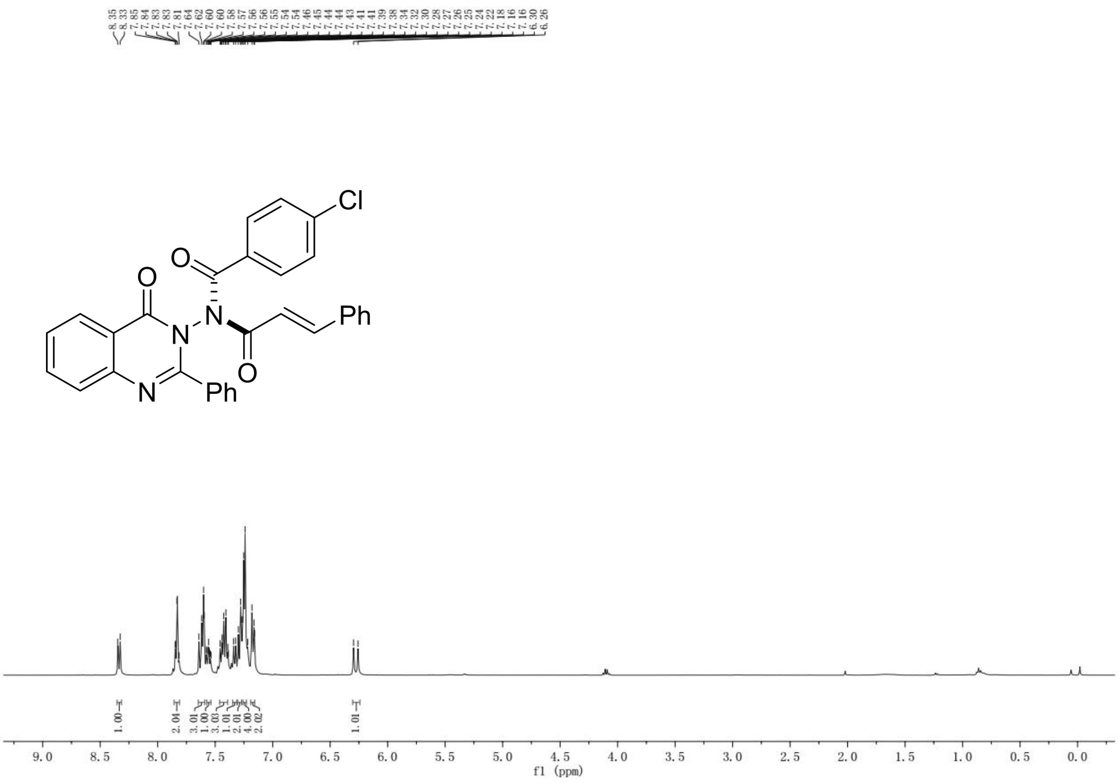

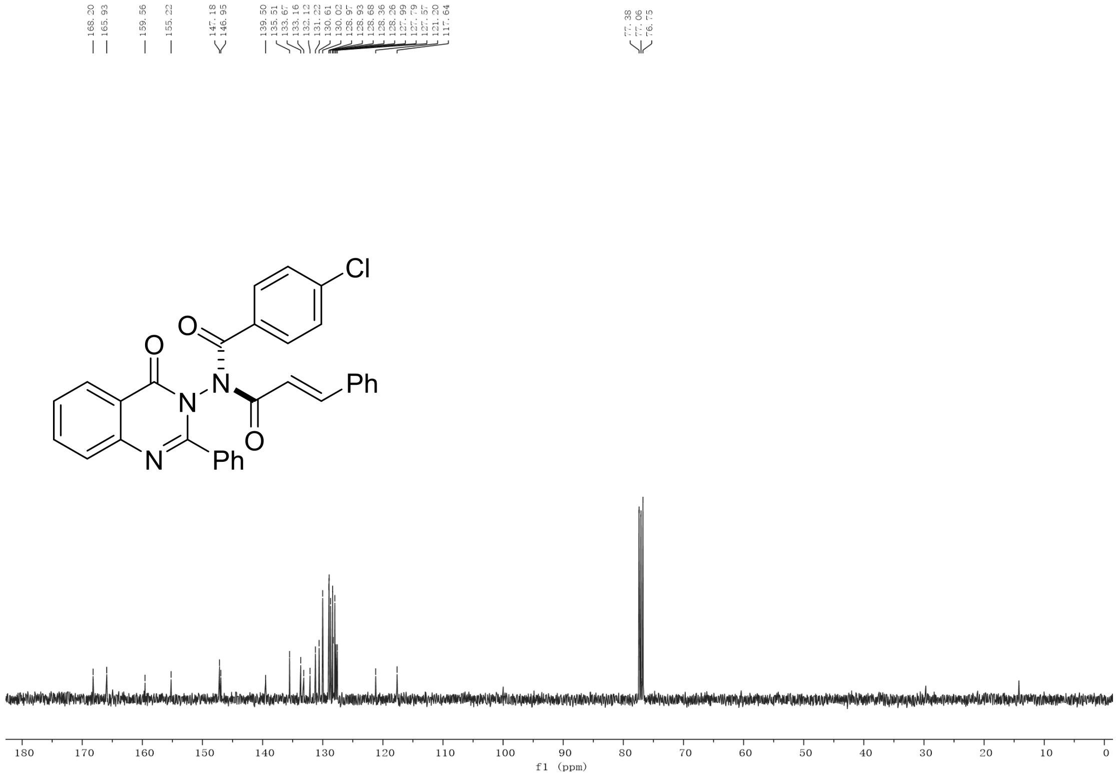

(*R*$_{a,cis,cis}$)-*N*-cinnamoyl-4-methyl-*N*-(4-oxo-2-phenylquinazolin-3(4*H*)-yl)benzamide (3ia)

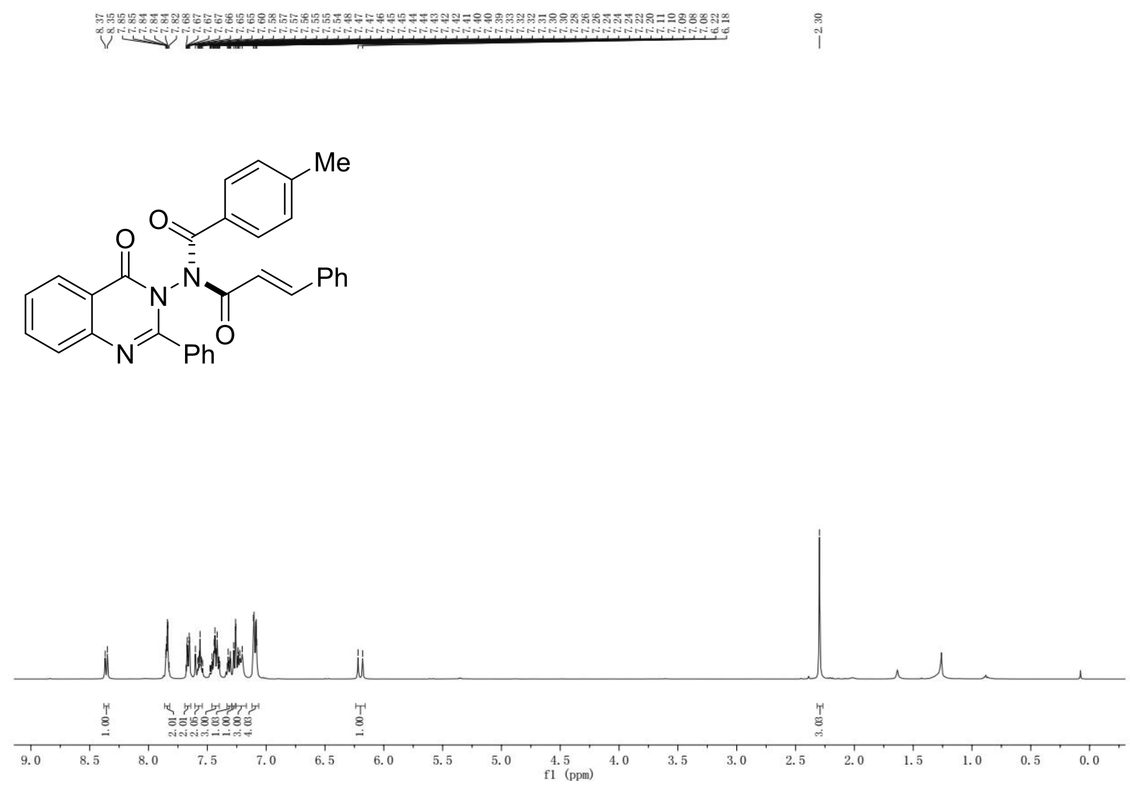

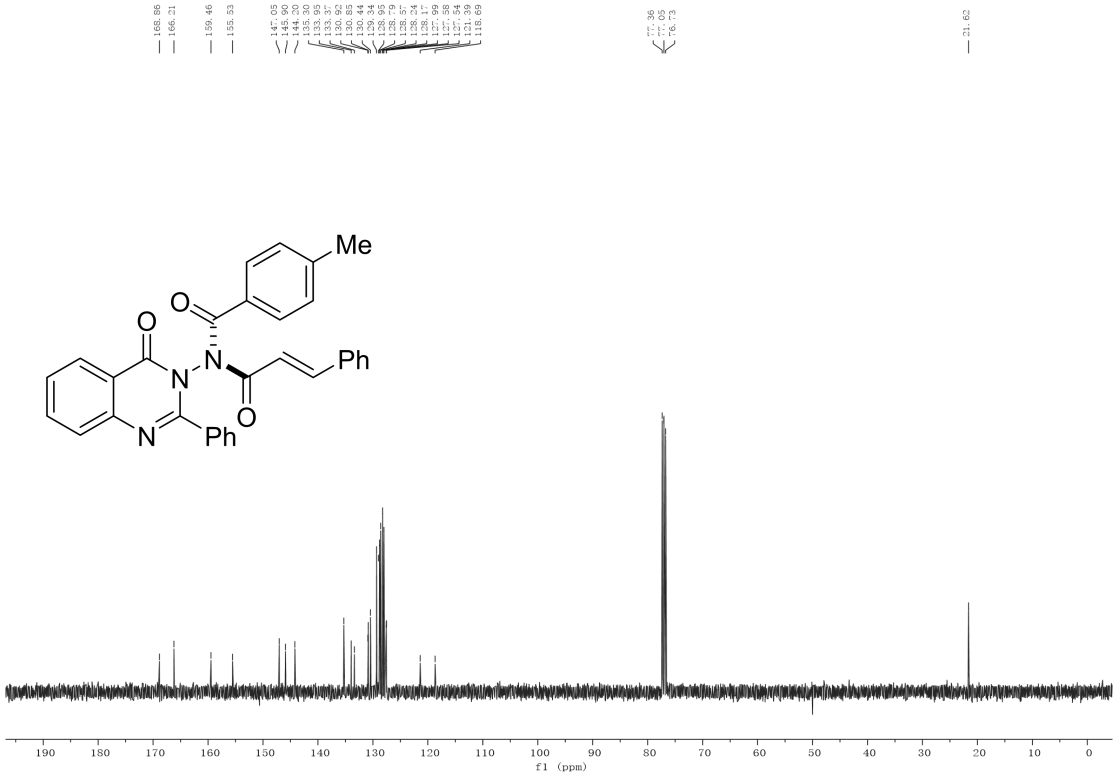

($R_{a,cis,cis}$)-*N*-cinnamoyl-3-methoxy-*N*-(4-oxo-2-phenylquinazolin-3(4*H*)-yl)benzamide (3ja)

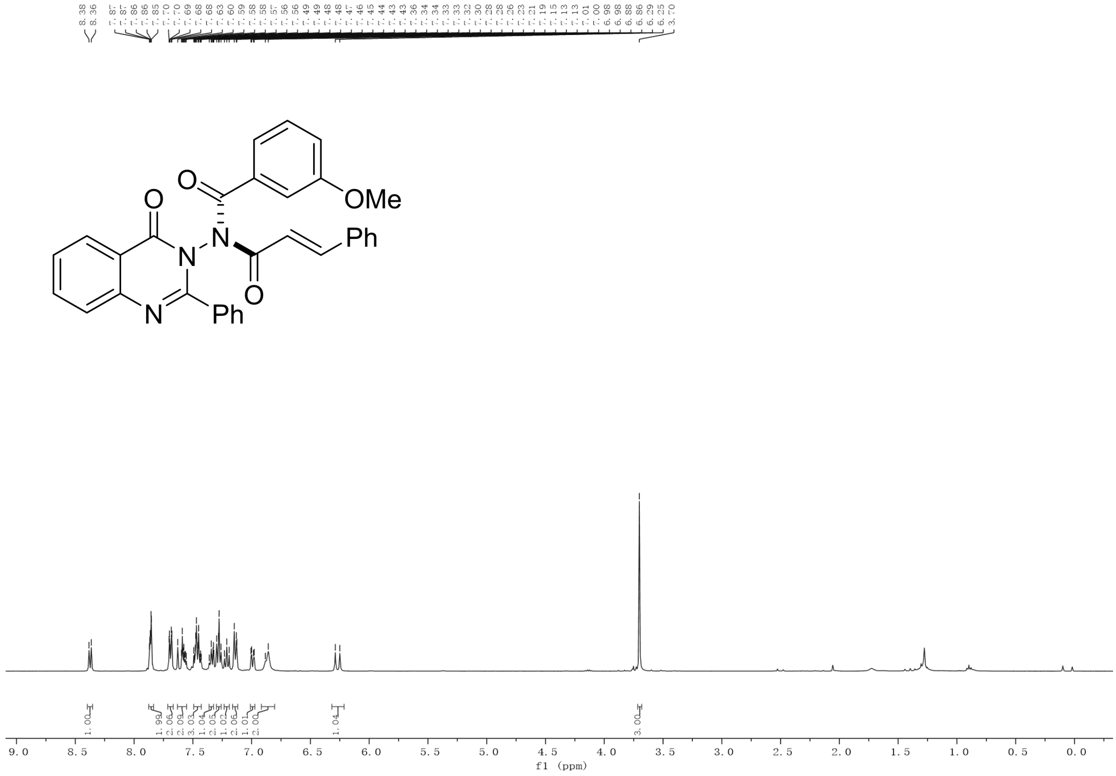

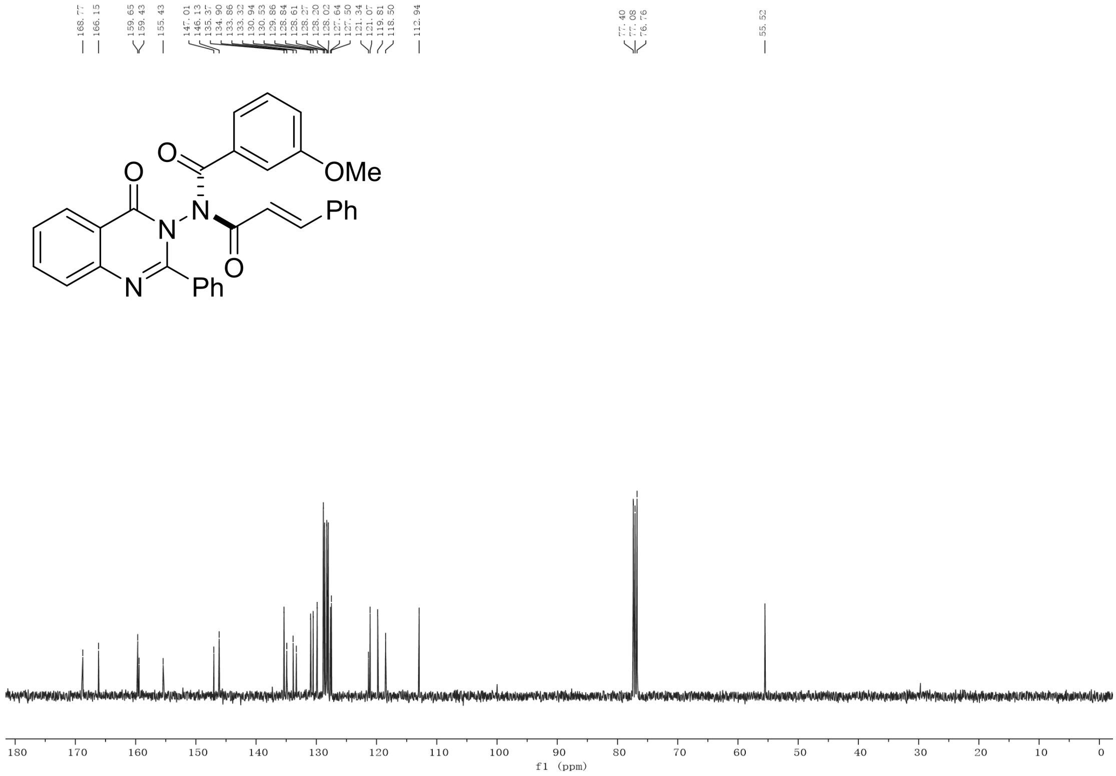

(*R*$_{a,cis,cis}$)-*N*-cinnamoyl-2-iodo-*N*-(4-oxo-2-phenylquinazolin-3(4*H*)-yl)benzamide (3ka)

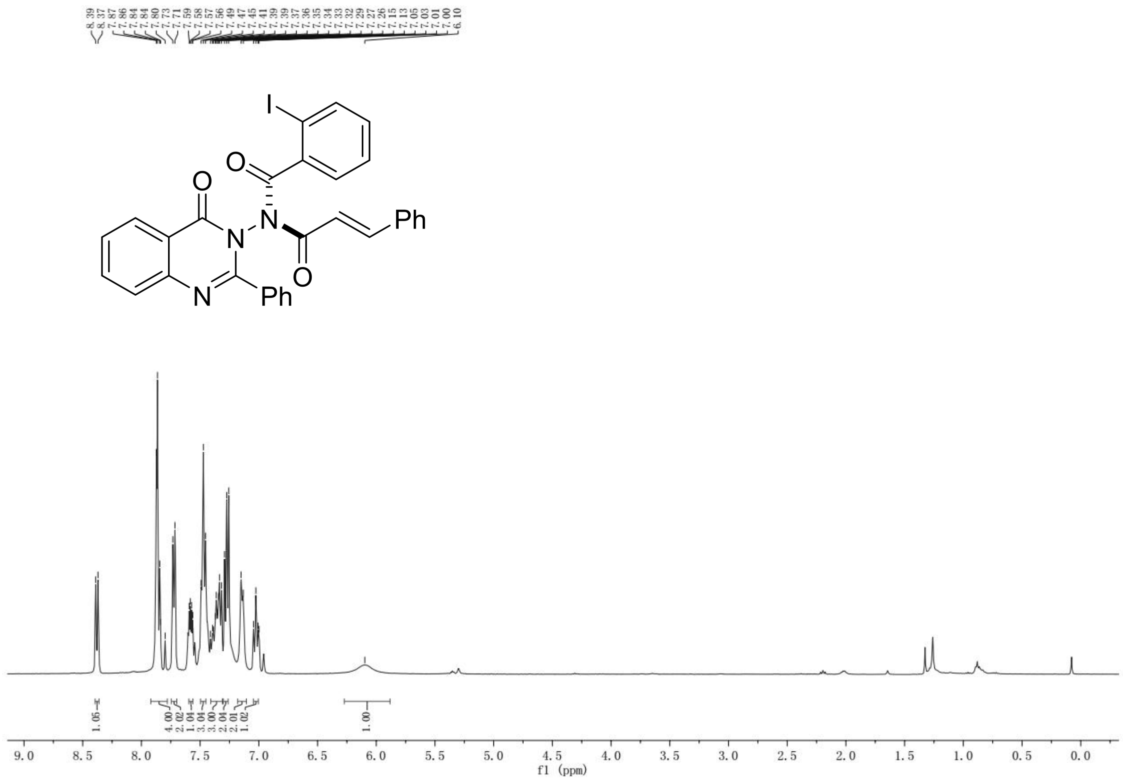

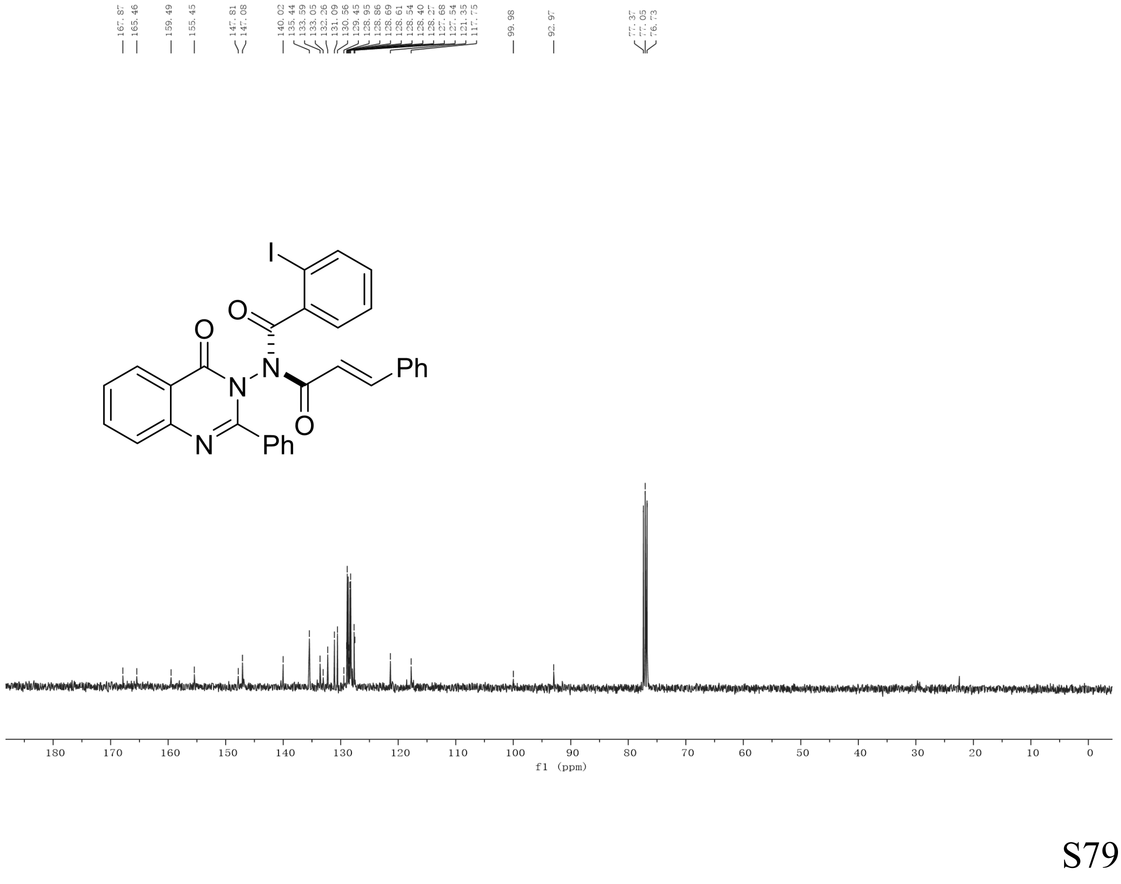

(*R*$_{a,cis,cis}$)-*N*-acetyl-*N*-(4-oxo-2-phenylquinazolin-3(4*H*)-yl)cinnamamide (3la)

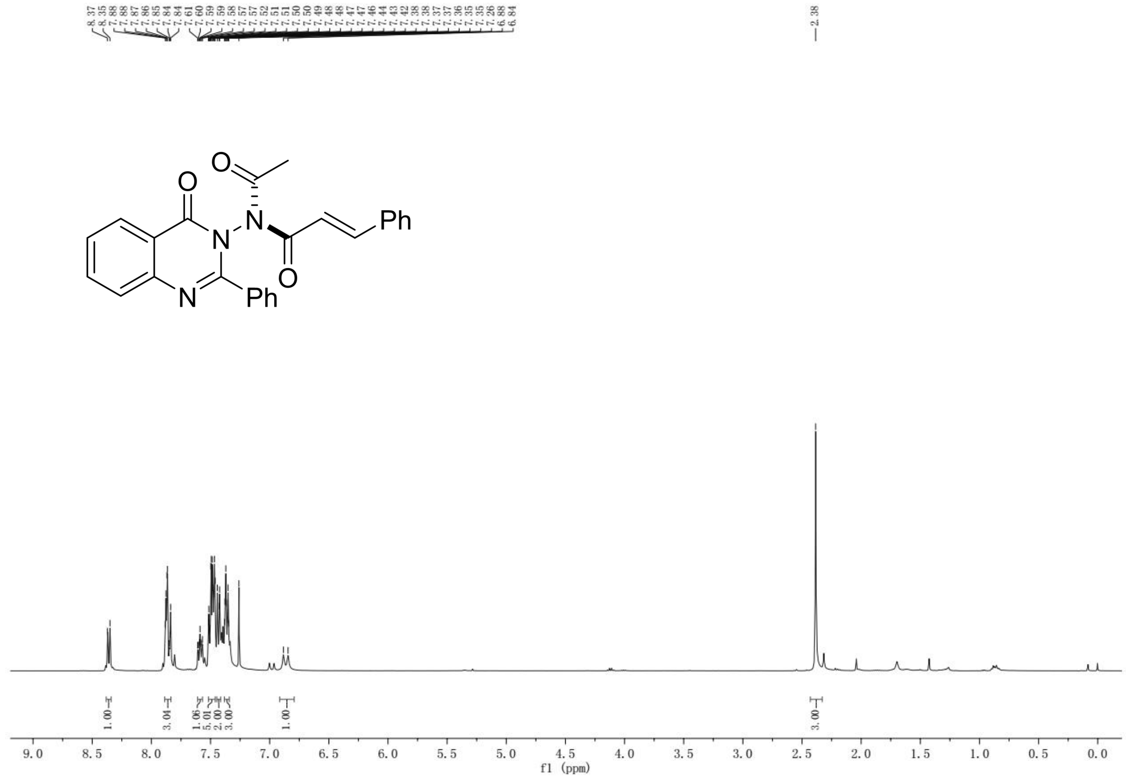

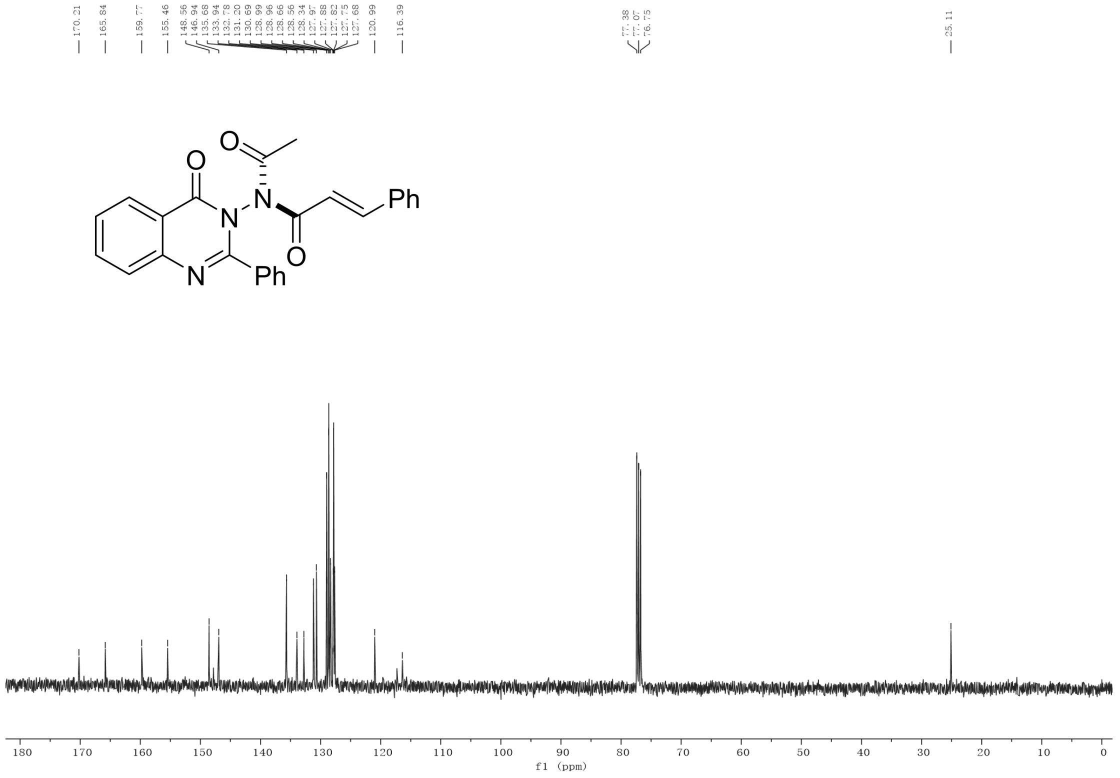

(*R*a,cis,cis)-*N*-(4-oxo-2-phenylquinazolin-3(4*H*)-yl)-*N*-(2-phenylacetyl)cinnamamide (3ma)

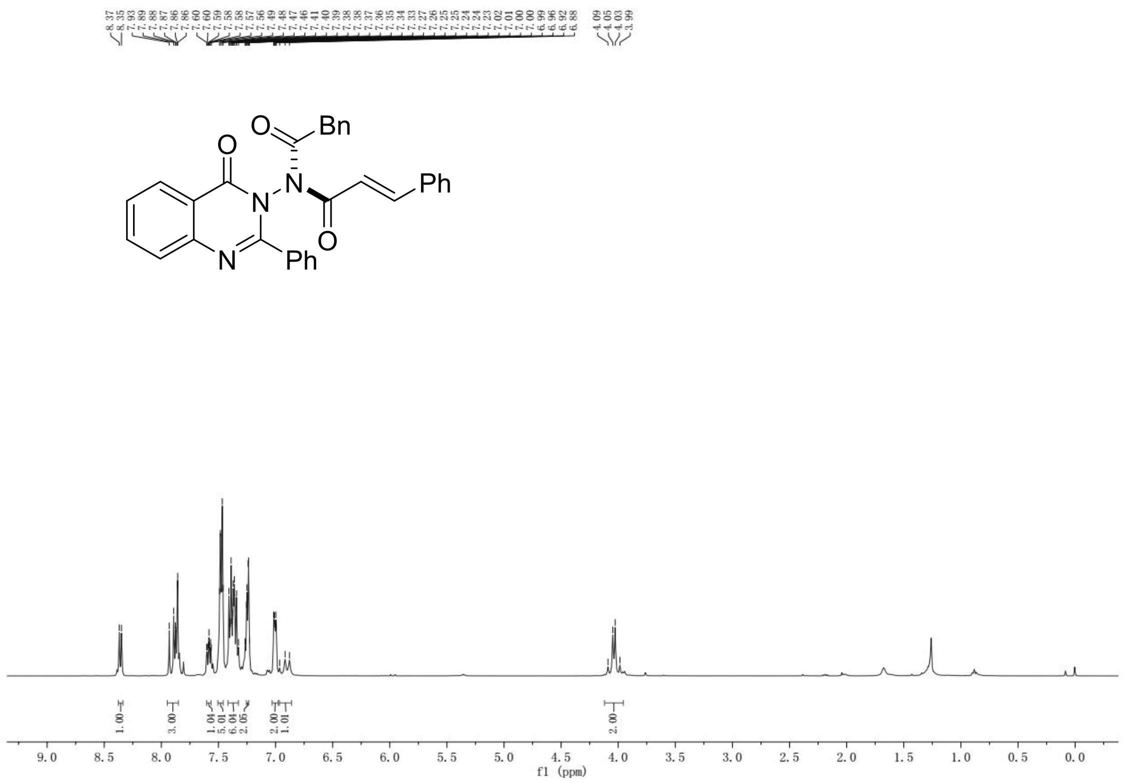

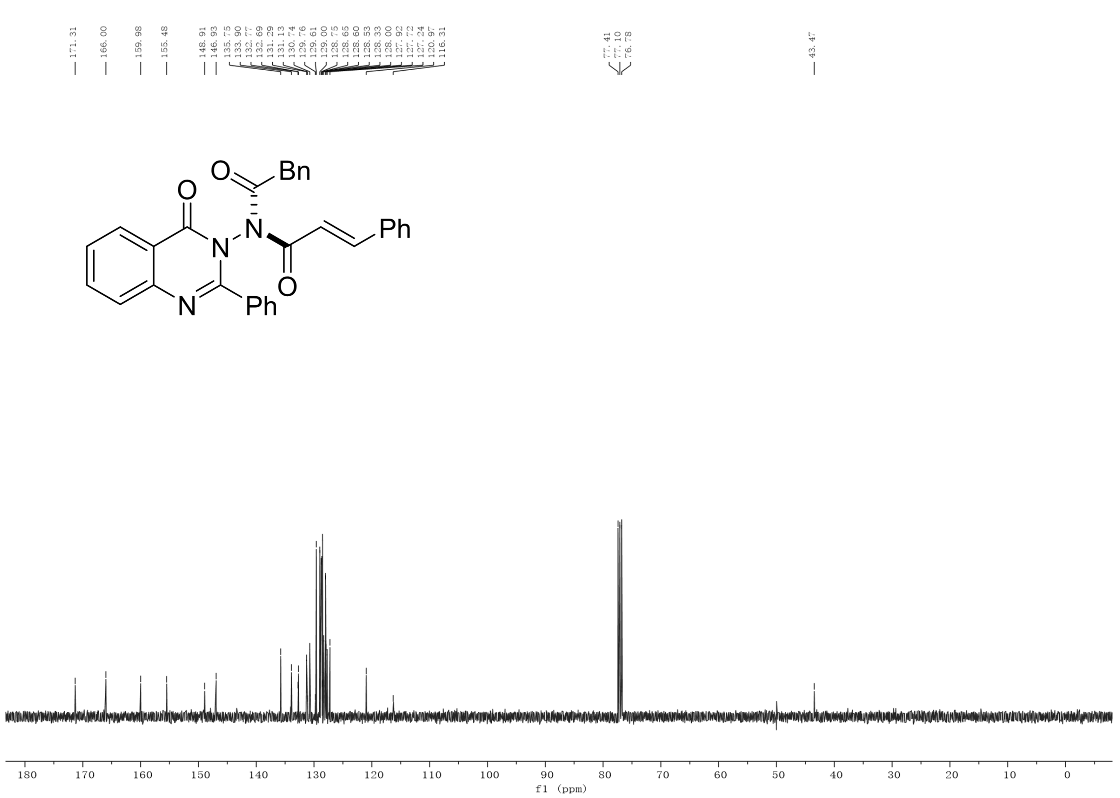

(*R*$_{a,cis,cis}$)-*N*-isobutyryl-*N*-(4-oxo-2-phenylquinazolin-3(4*H*)-yl)cinnamamide (3na)

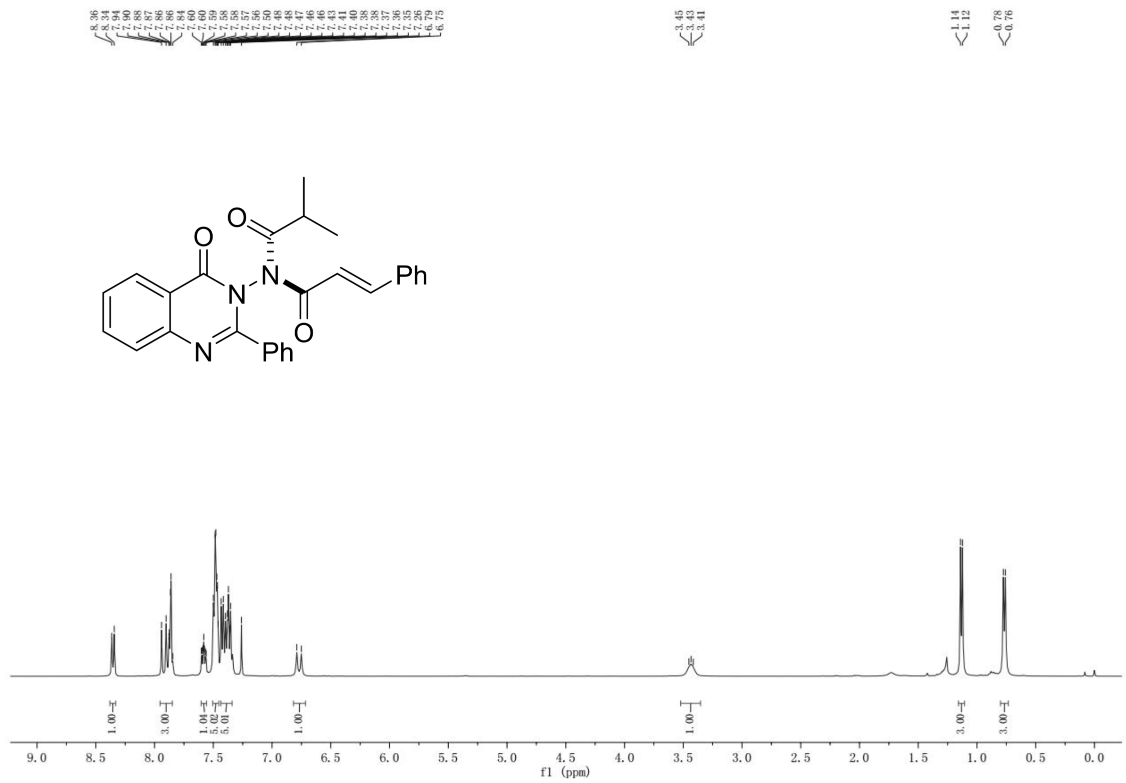

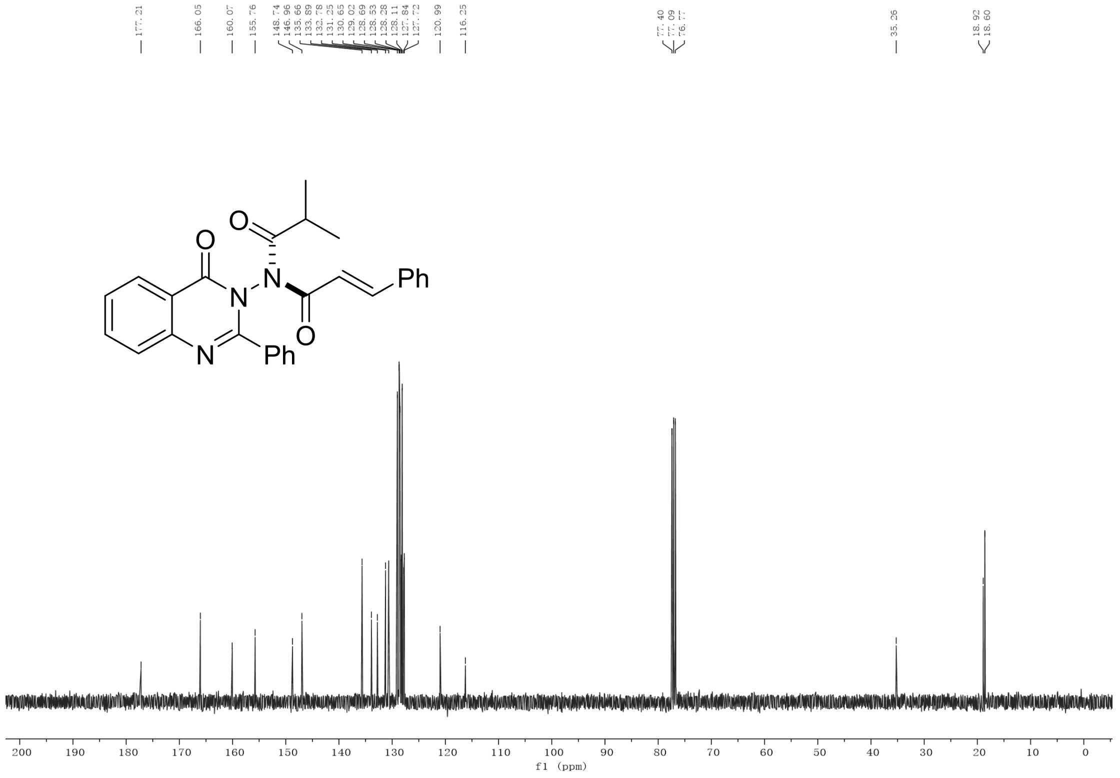

($R_{a,cis,cis}$)-*N*-cinnamoyl-*N*-(4-oxo-2-phenylquinazolin-3(4*H*)-yl)cyclohexane carboxamide (3oa)

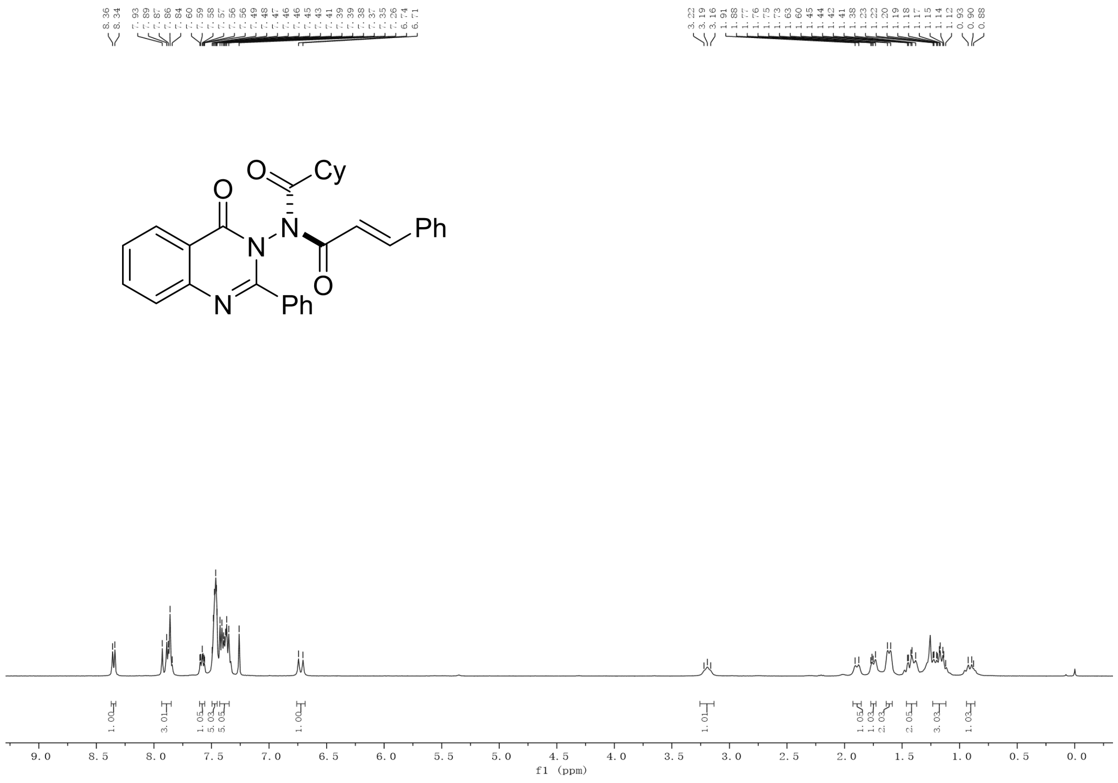

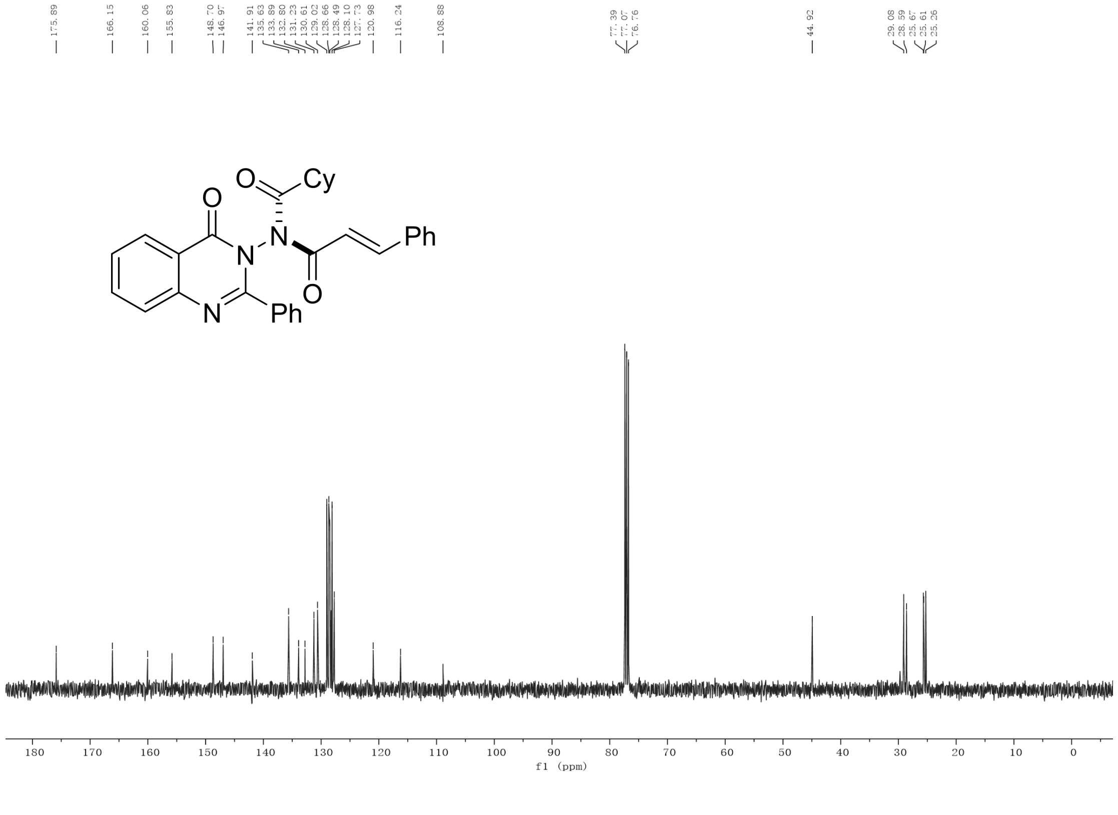

(*R*$_{a,cis,cis}$)-*N*-(2-(4-bromophenyl)-4-oxoquinazolin-3(4*H*)-yl)-*N*-cinnamoylbenzamide (3pa)

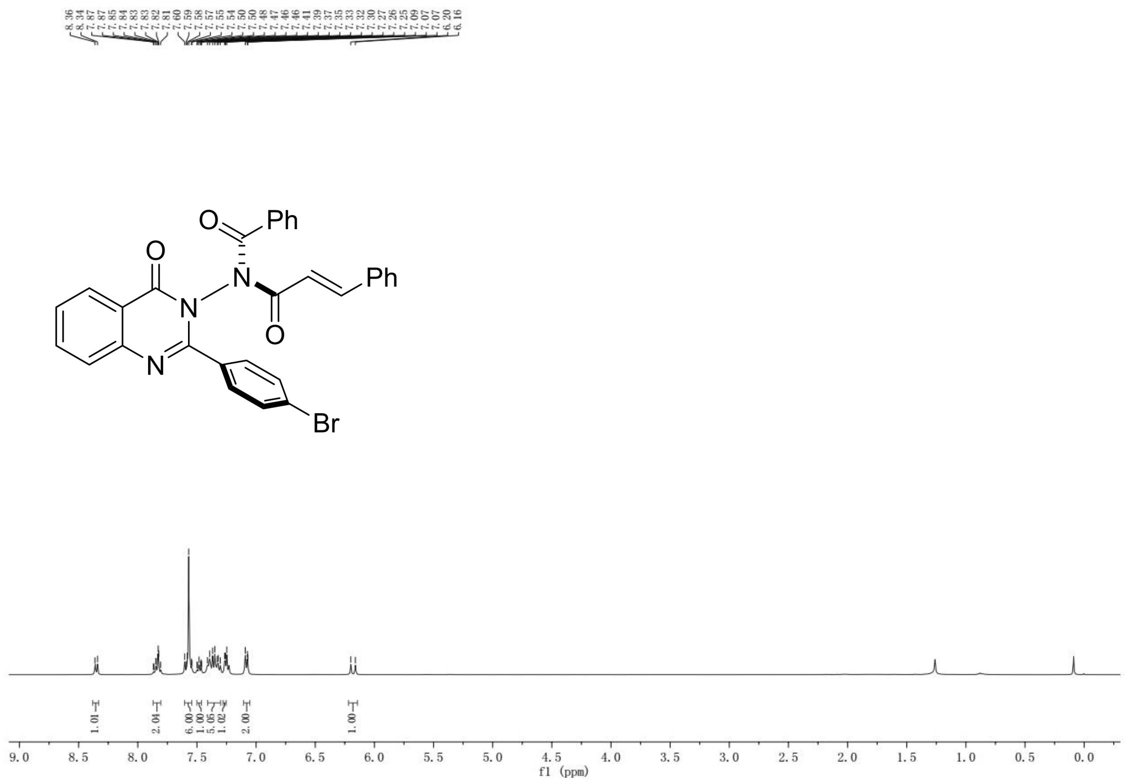

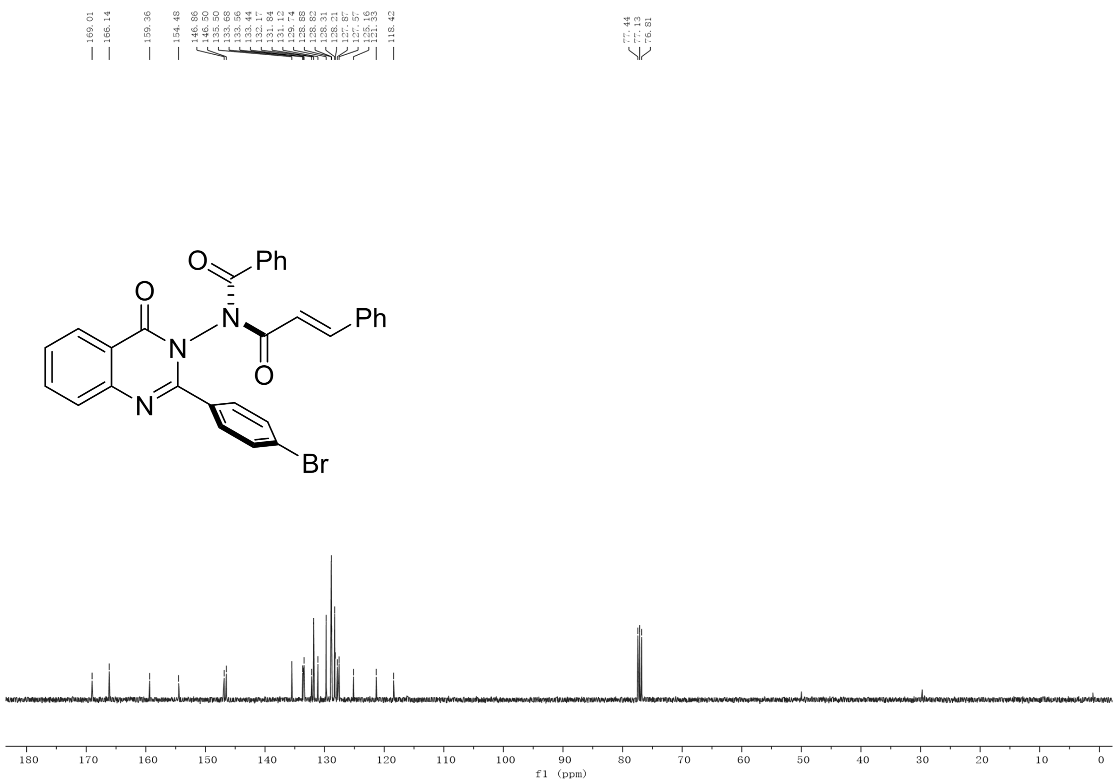

(*R*$_{a,cis,cis}$)-*N*-cinnamoyl-*N*-(2-(4-methoxyphenyl)-4-oxoquinazolin-3(4*H*)-yl) benzamide (3qa)

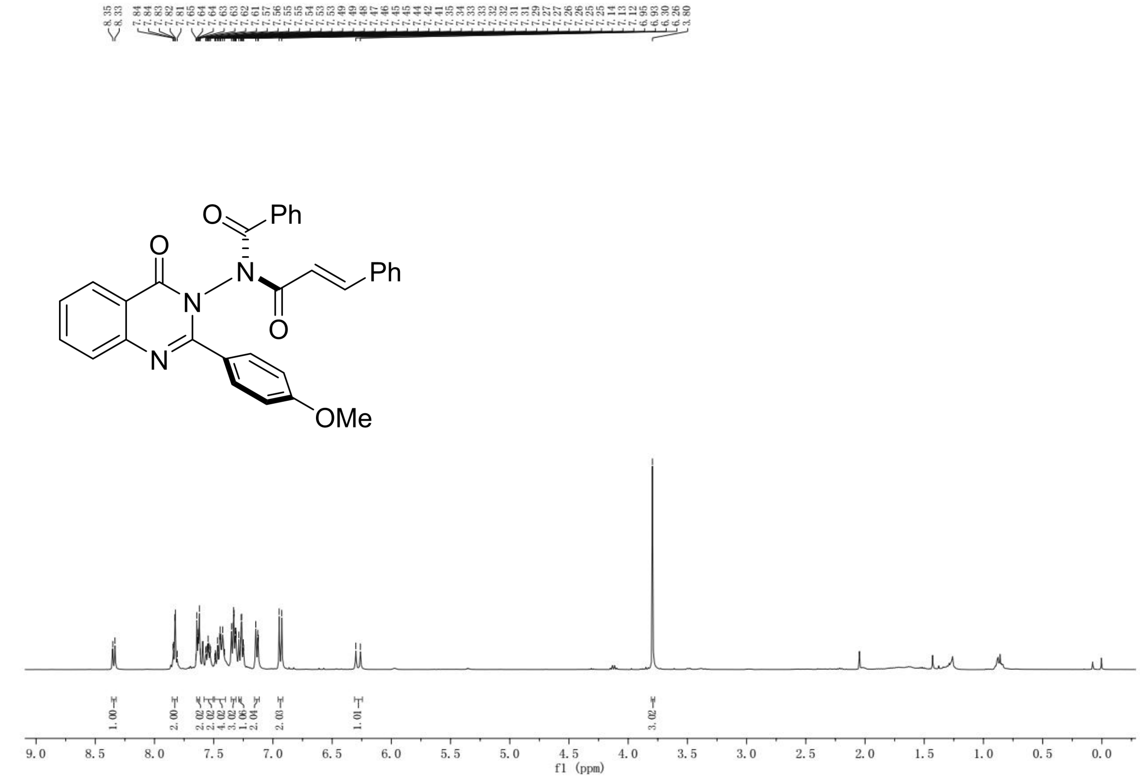

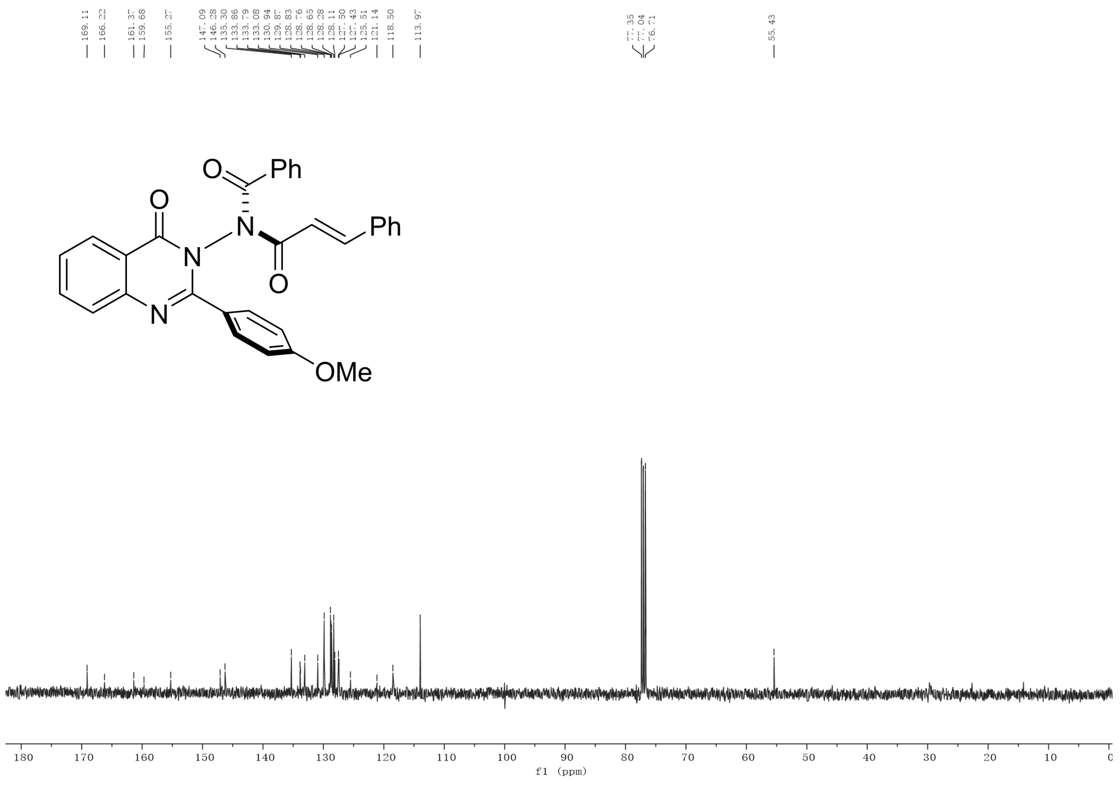

(*R*$_{a,cis,cis}$)-*N*-cinnamoyl-*N*-(4-oxo-2-(m-tolyl)quinazolin-3(4*H*)-yl)benzamide (3ra)

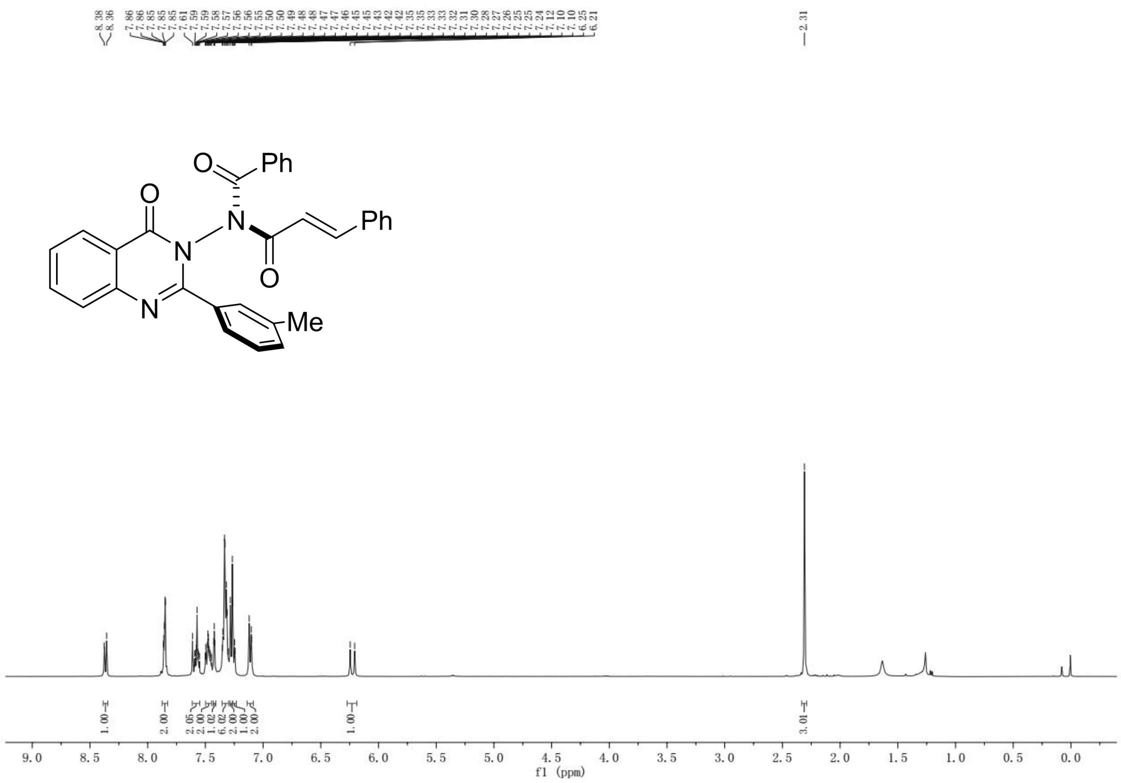

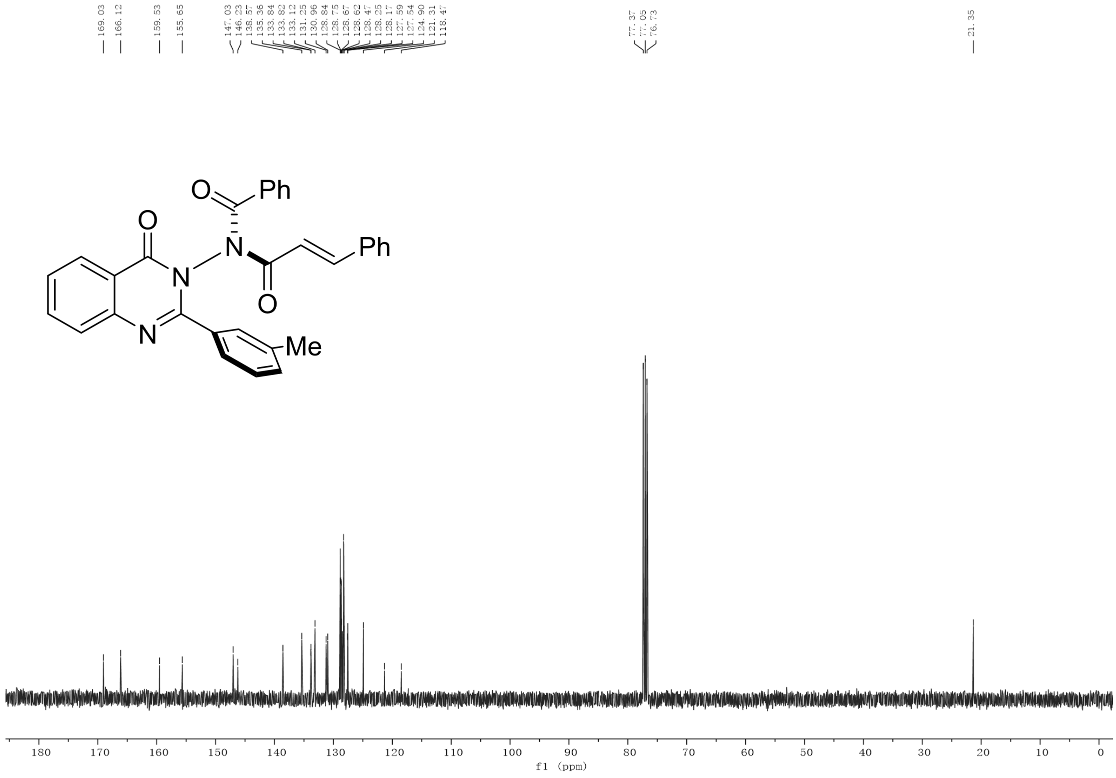

(*R*$_{a,cis,cis}$)-*N*-cinnamoyl-*N*-(2-isopropyl-4-oxoquinazolin-3(4*H*)-yl)benzamide (3sa)

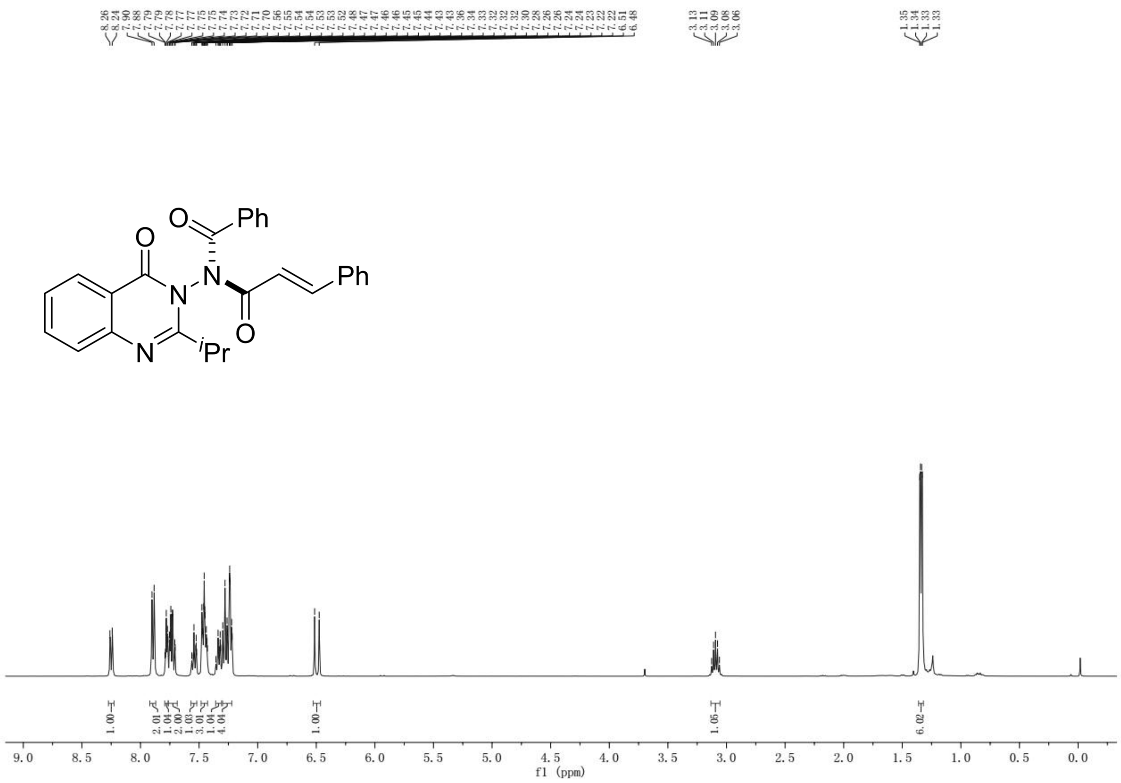

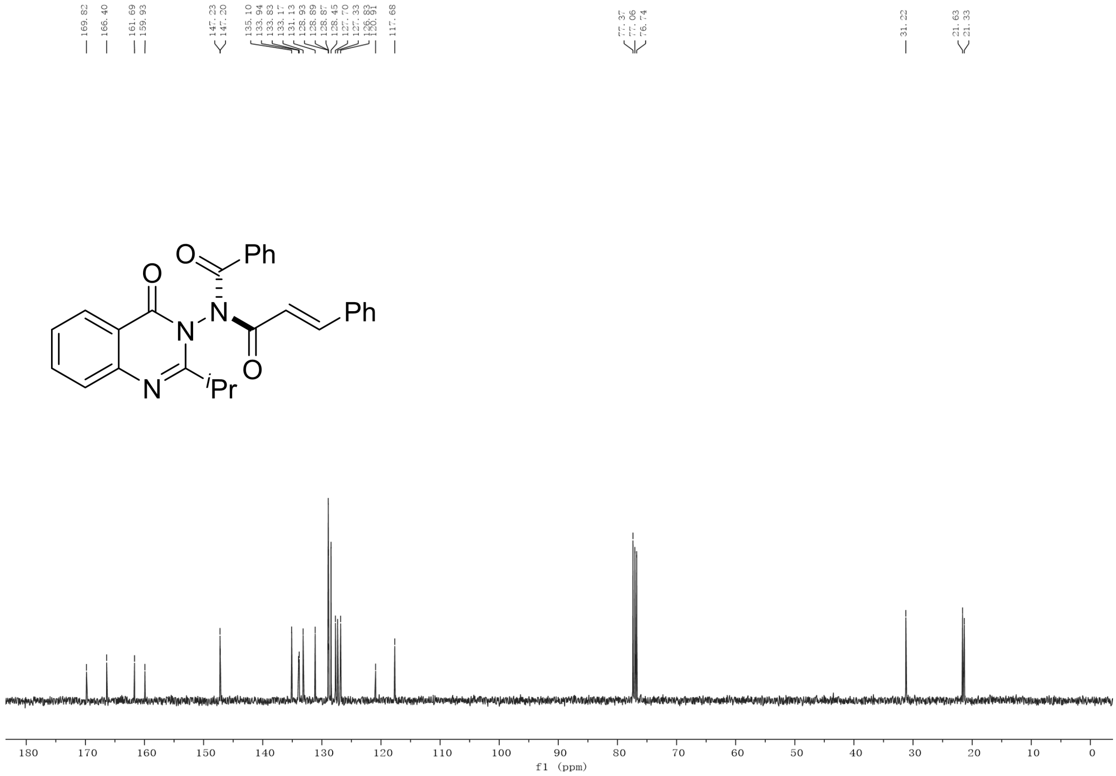

($R_{a,cis,cis}$)-(*E*)-*N*-(2-(1-(4-chlorobenzoyl)-5-methoxy-2-methyl-1H-indol-3-yl)acetyl)-3-(4-methoxyphenyl)-*N*-(4-oxo-2-phenylquinazolin-3(4*H*)-yl)acrylamide (3ta)

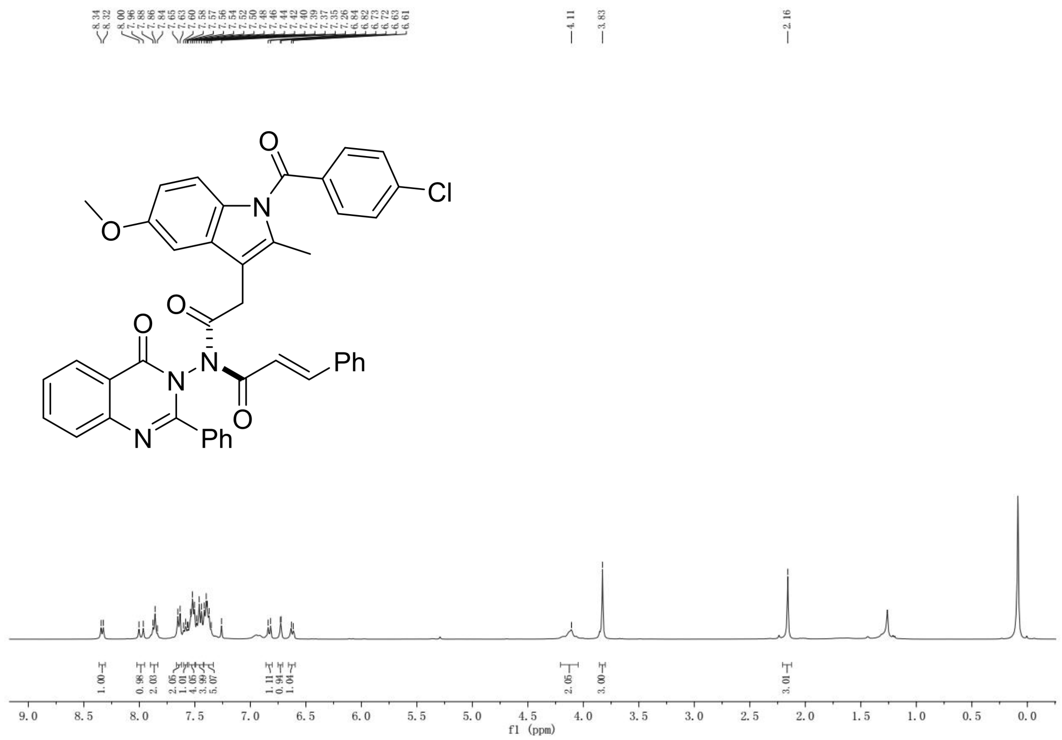

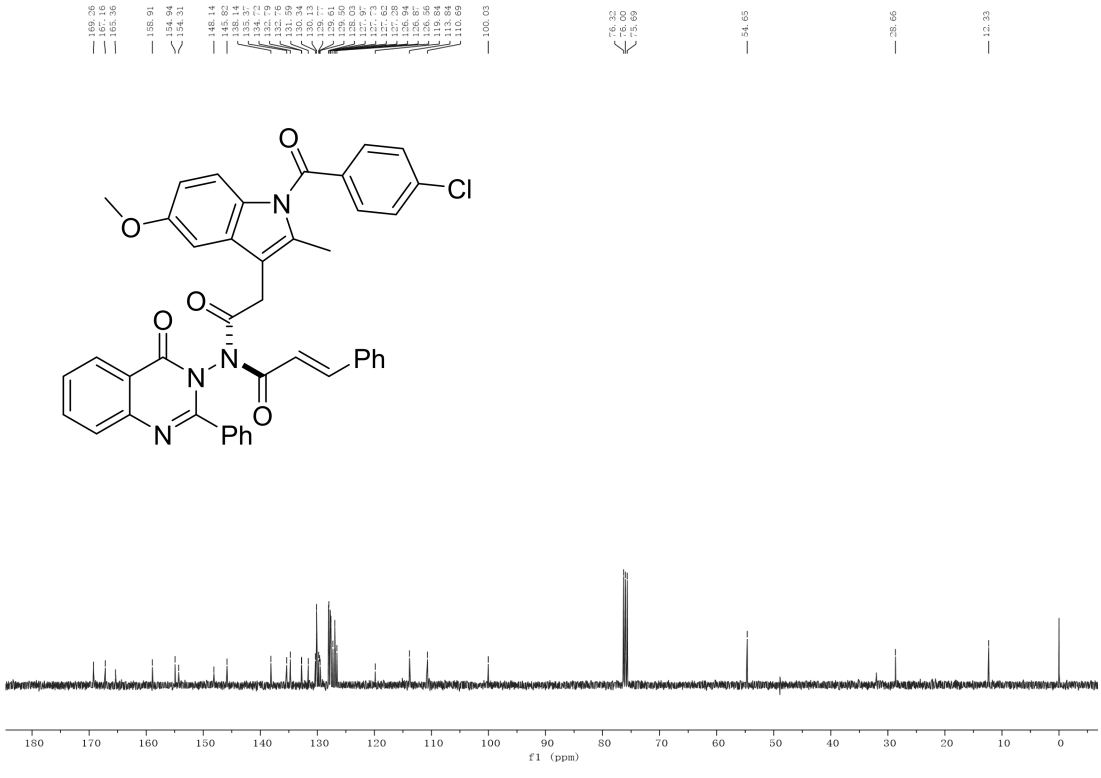

(*R*$_{a,cis,cis}$)-(*S*)-*N*-(2-(6-methoxynaphthalen-2-yl)propanoyl)-*N*-(4-oxo-2-phenylquinazolin-3(4*H*)-yl)cinnamamide(3ua)

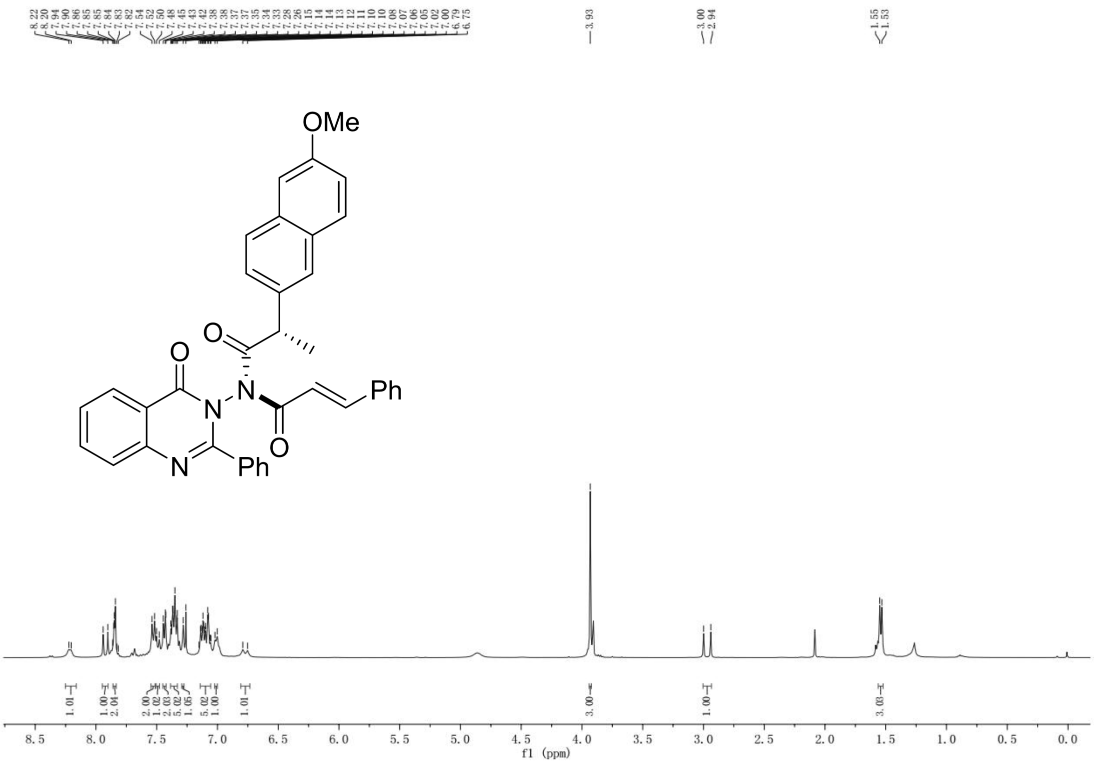

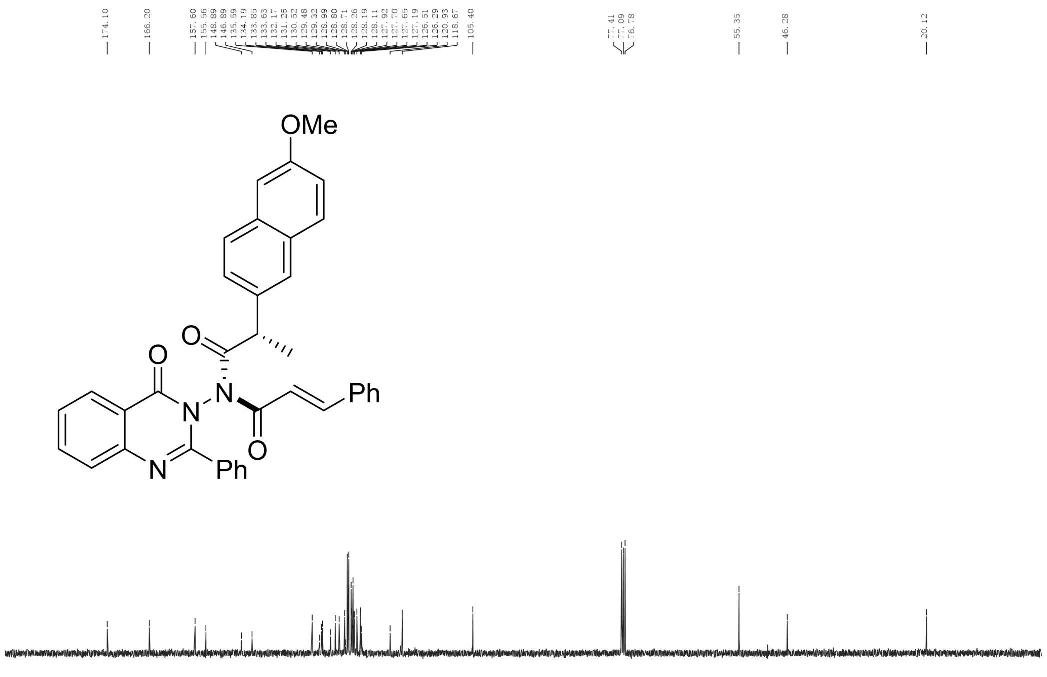

($R_{a,cis,cis}$)-(*E*)-*N*-(4-oxo-2-phenylquinazolin-3(4*H*)-yl)-*N*-(3-(4-(trifluoromethyl)phenyl)acryloyl)benzamide (3ab)

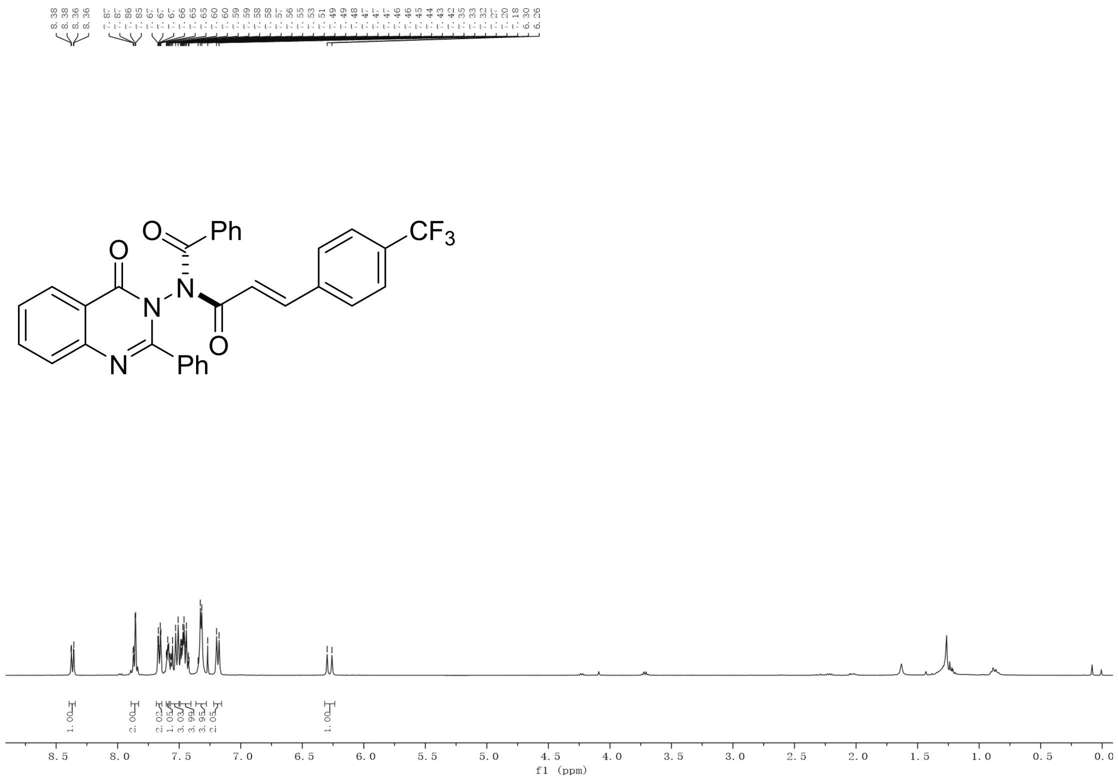

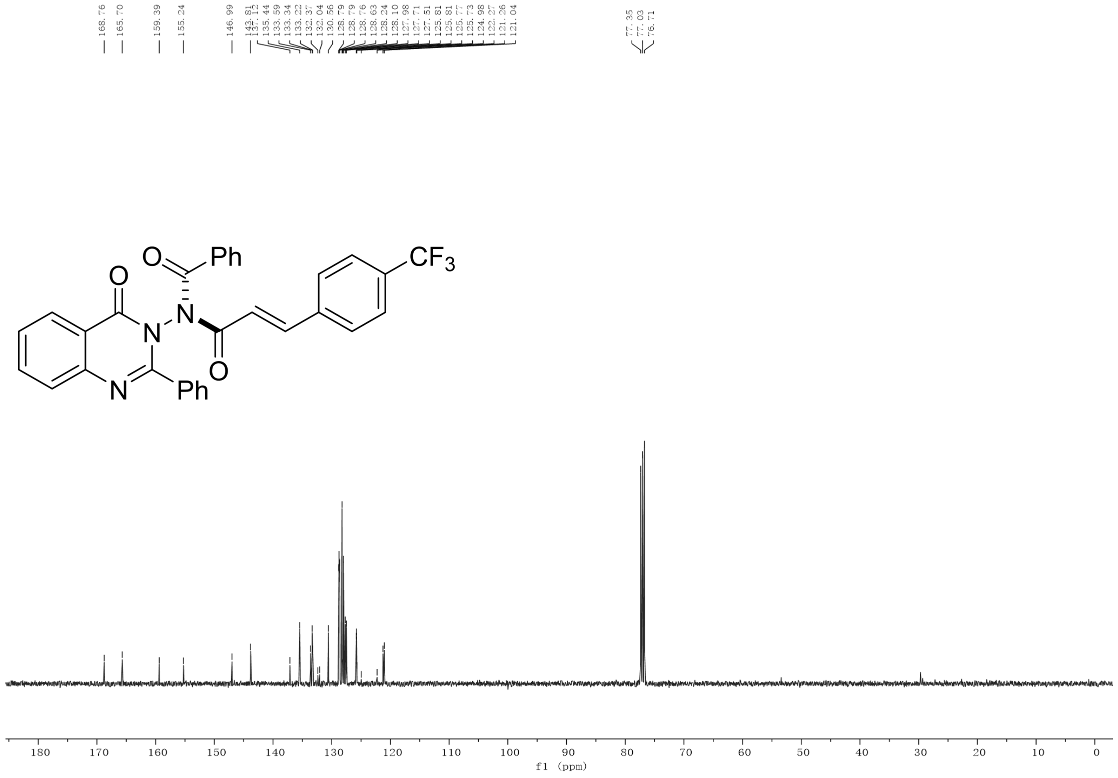

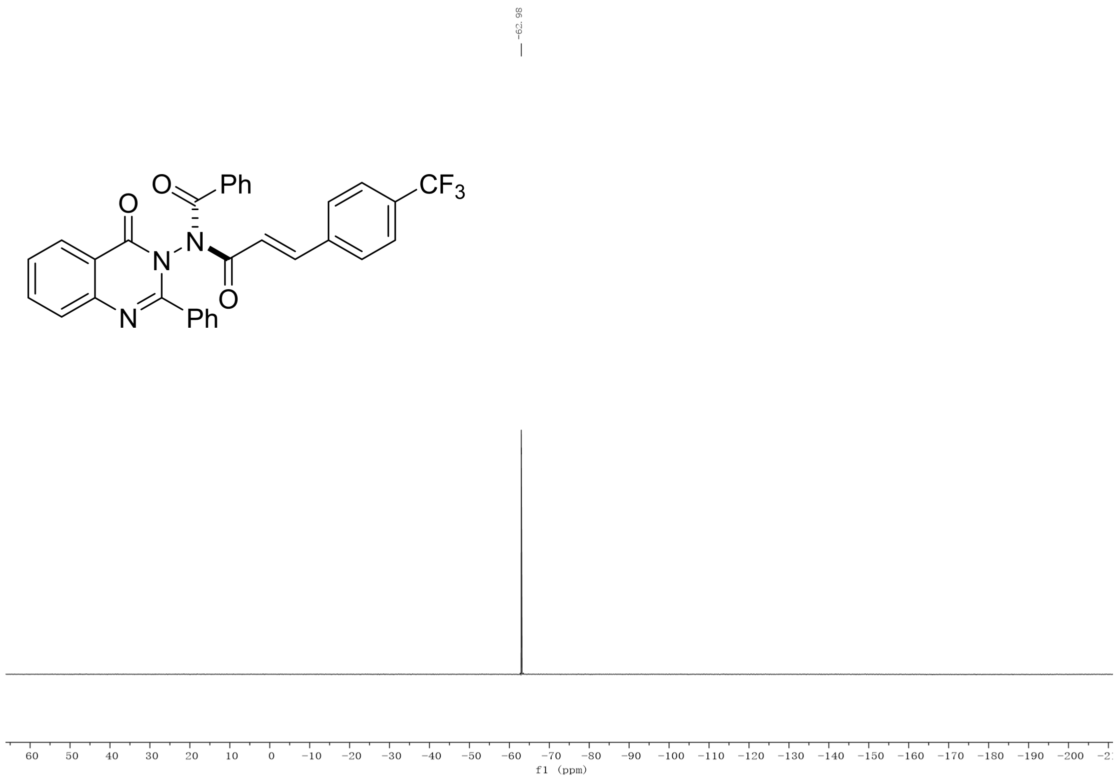

-62.98
O
Ph
CF3
O
N
N
O
N
Ph
60 50 40 30 20 10 0 -10 -20 -30 -40 -50 -60 -70 -80 -90 -100 -110 -120 -130 -140 -150 -160 -170 -180 -190 -200
f1 (ppm)

(*R*$_{a,cis,cis}$)-(*E*)-*N*-(3-(3-bromophenyl)acryloyl)-*N*-(4-oxo-2-phenylquinazolin-3(4*H*)-yl)benzamide (3ac)

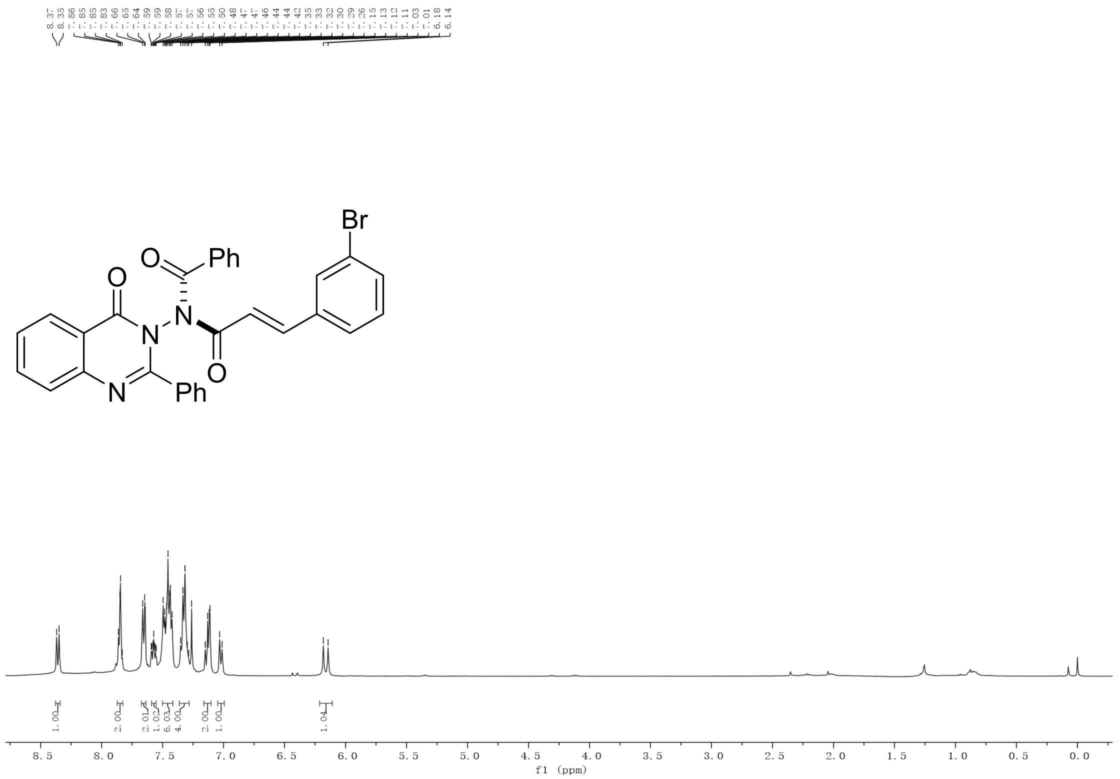

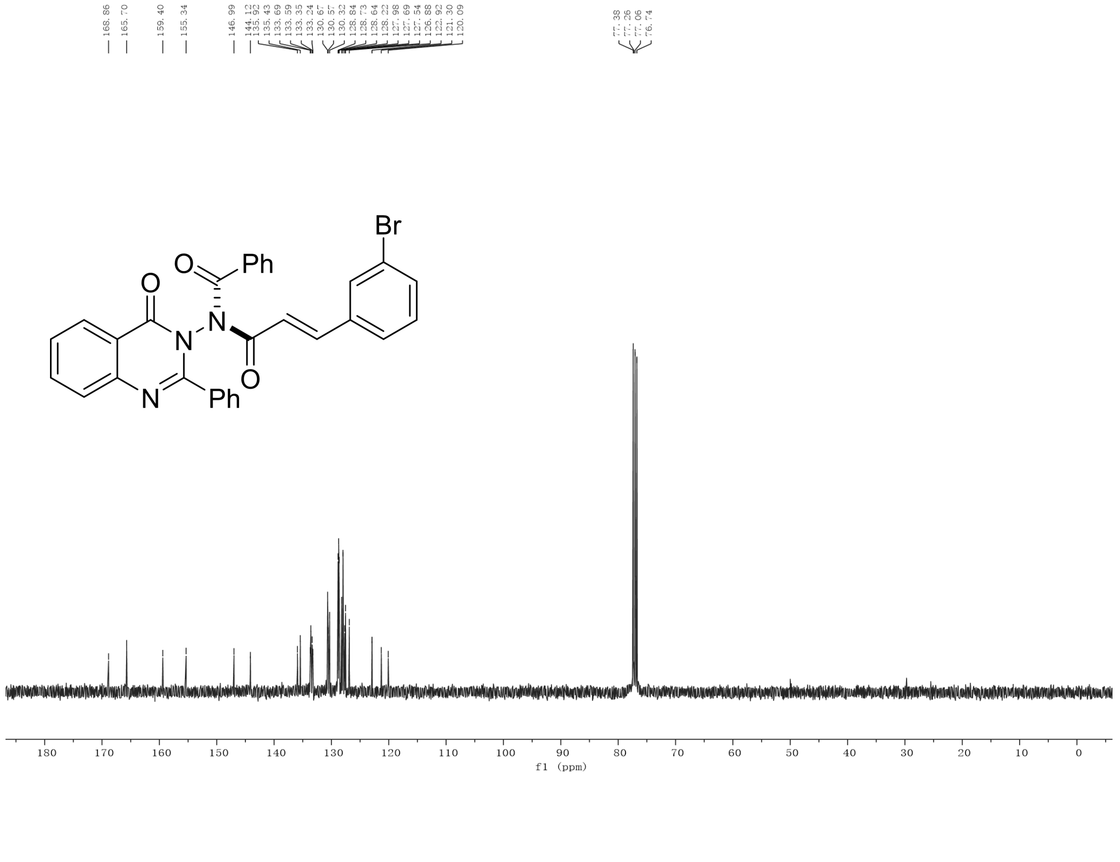

($R_{a,cis,cis}$)-(E)-N-(3-(3-fluorophenyl)acryloyl)-N-(4-oxo-2-phenylquinazolin-3 (4H)-yl)benzamide (3ad)

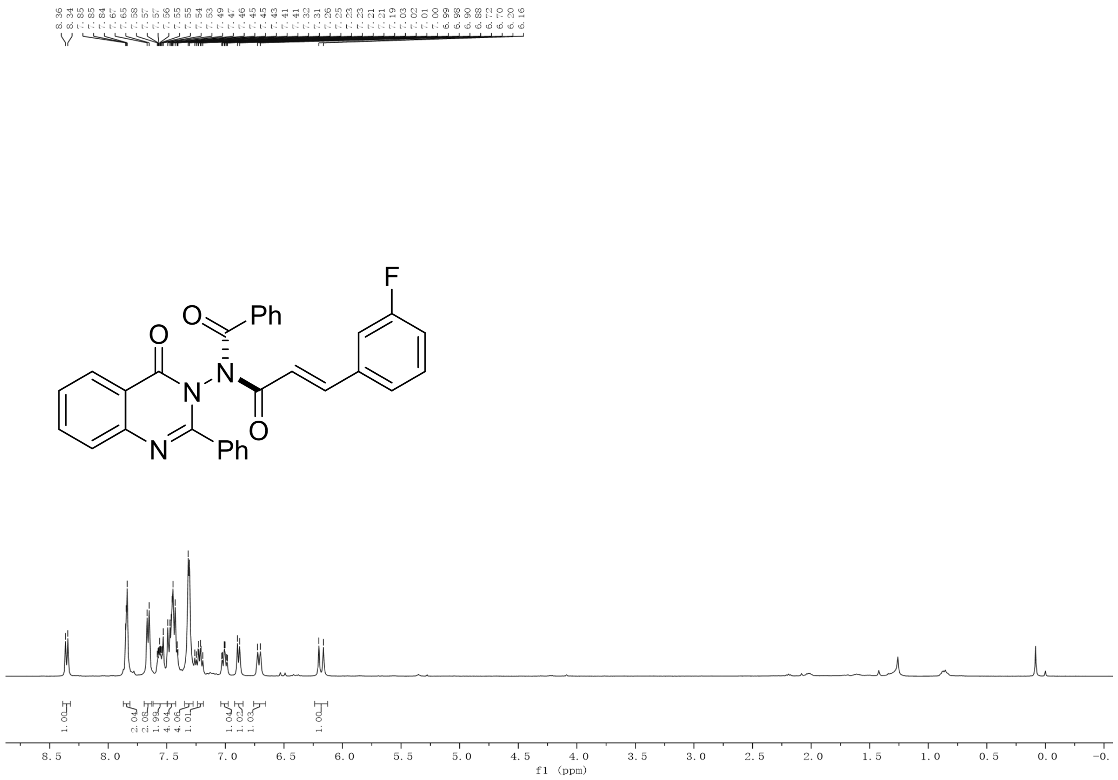

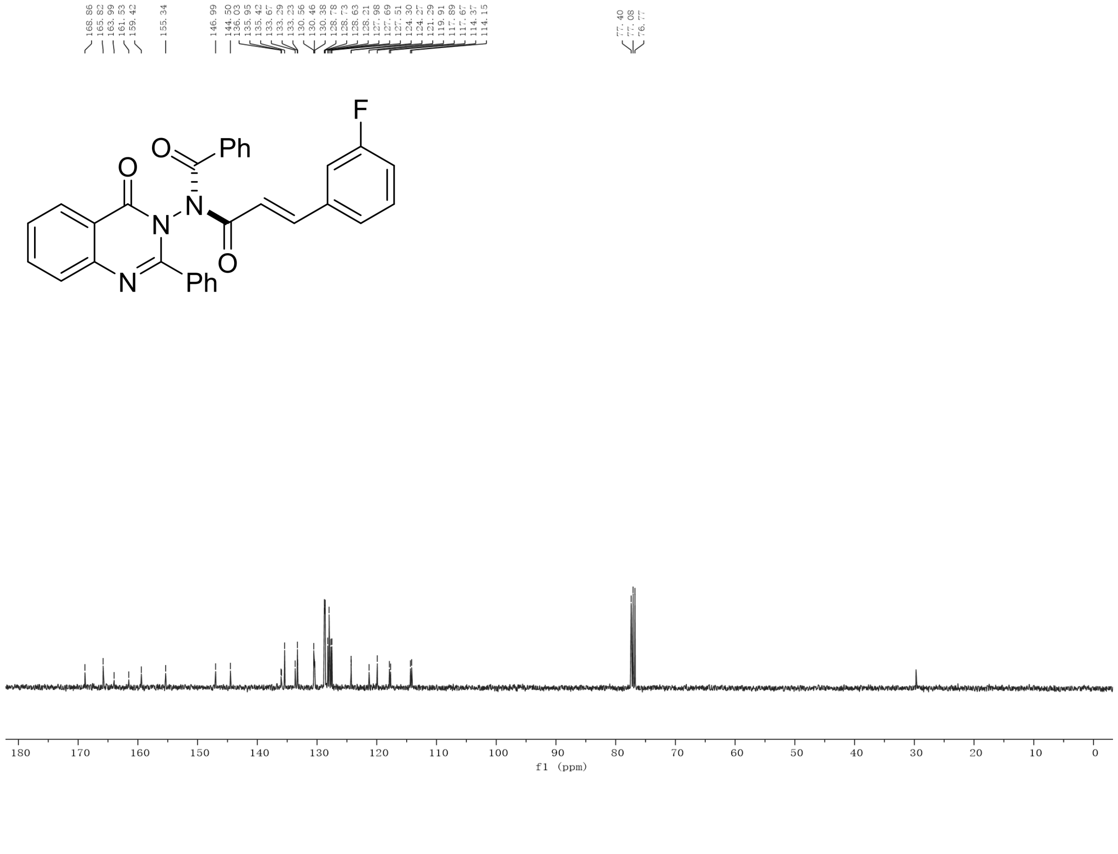

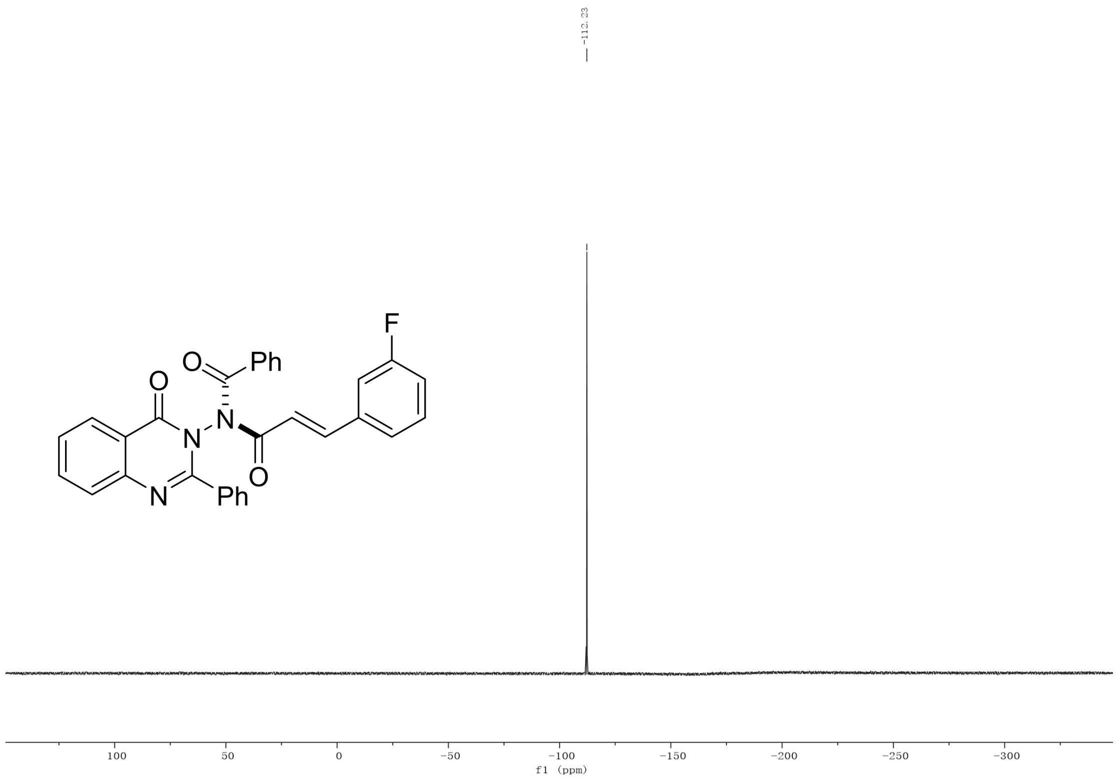

-112.23
O
Ph
O
N
N
O
N
Ph
F
100
50
0
-50
-100
-150
-200
-250
-300
f1 (ppm)

($R_{a,cis,cis}$)-(*E*)-*N*-(4-oxo-2-phenylquinazolin-3(4*H*)-yl)-*N*-(3-(o-tolyl)acryloyl) benzamide (3ae)

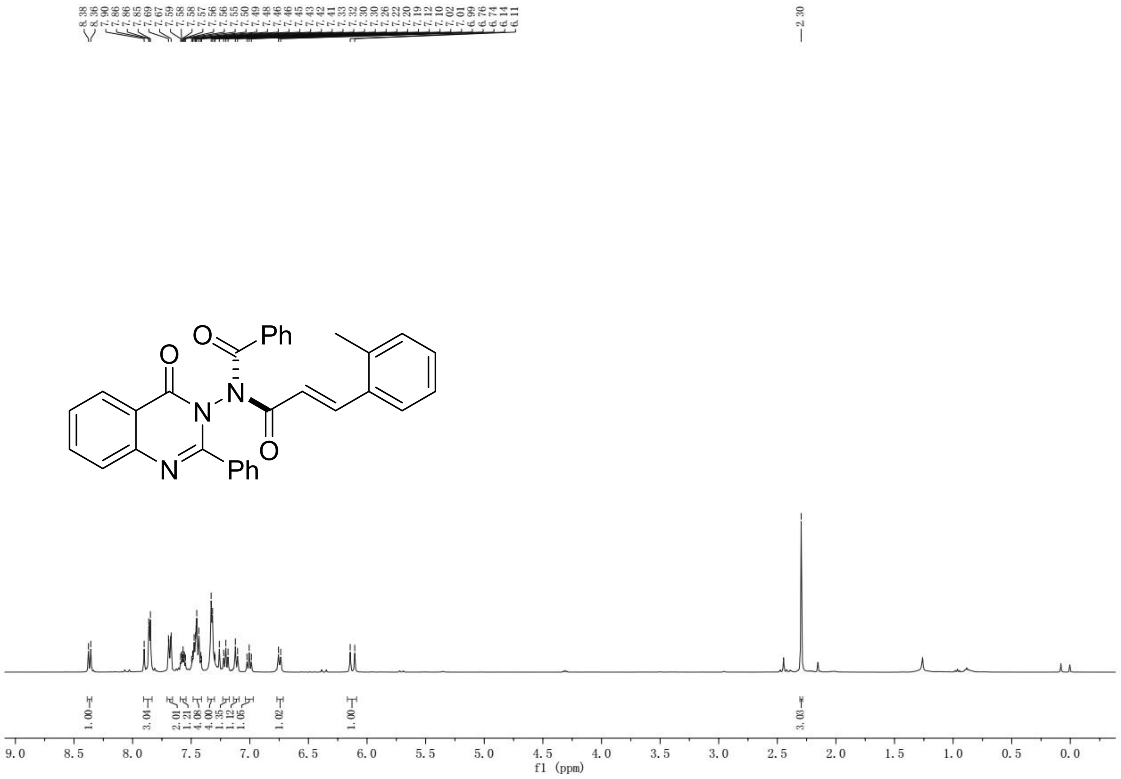

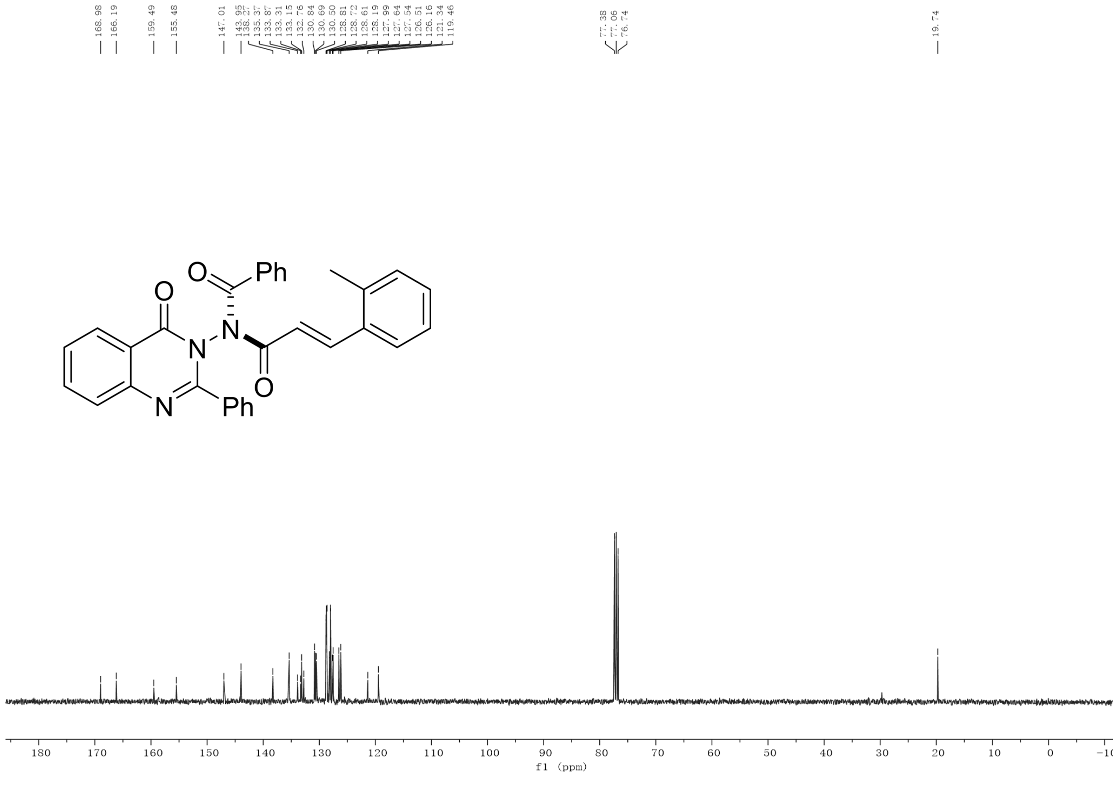

($R_{a,cis,cis}$)-(*E*)-*N*-(3-(naphthalen-1-yl)acryloyl)-*N*-(4-oxo-2-phenylquinazolin-3(4*H*)-yl)benzamide (3af)

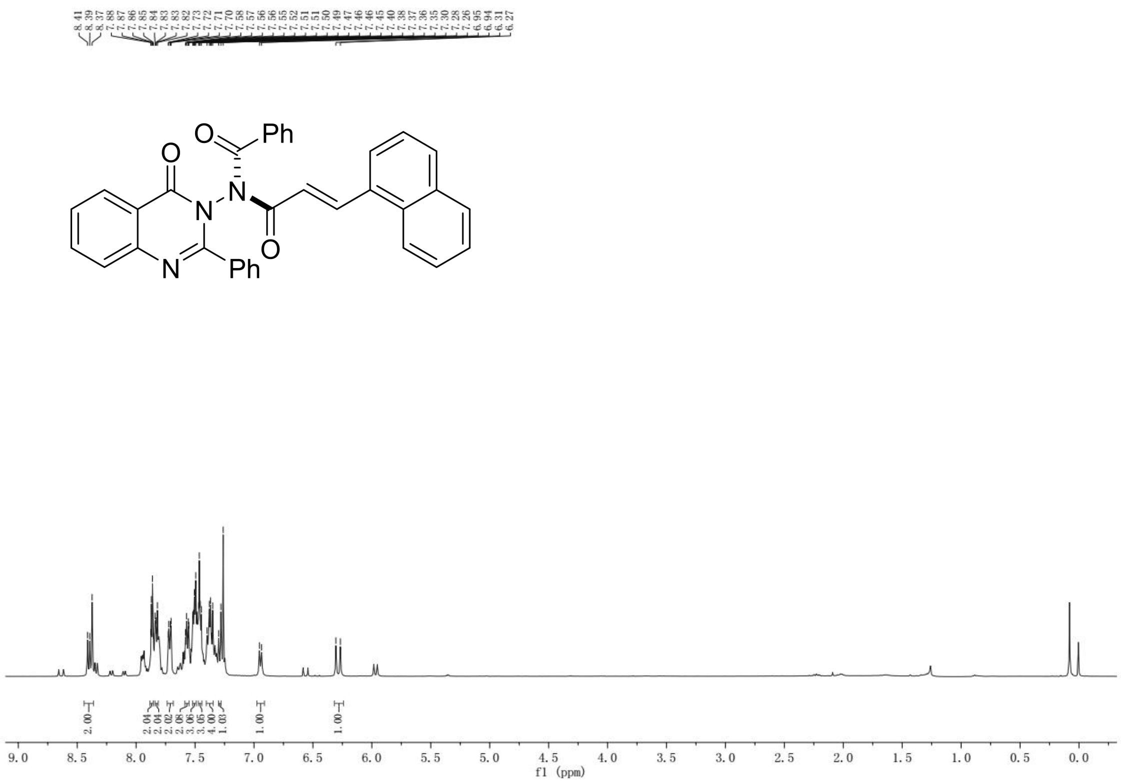

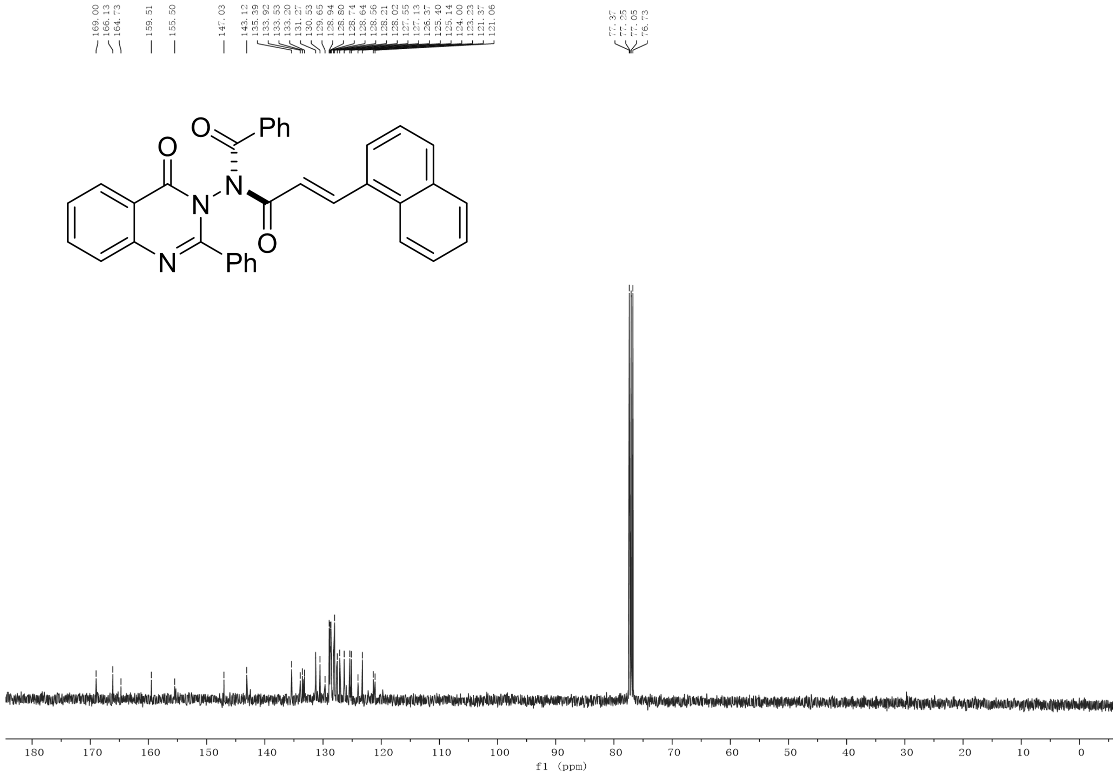

(*R*$_{a,cis,cis}$)-(*E*)-*N*-(4-oxo-2-phenylquinazolin-3(4*H*)-yl)-*N*-(3-(thiophen-2-yl)acryloyl)benzamide (3ag)

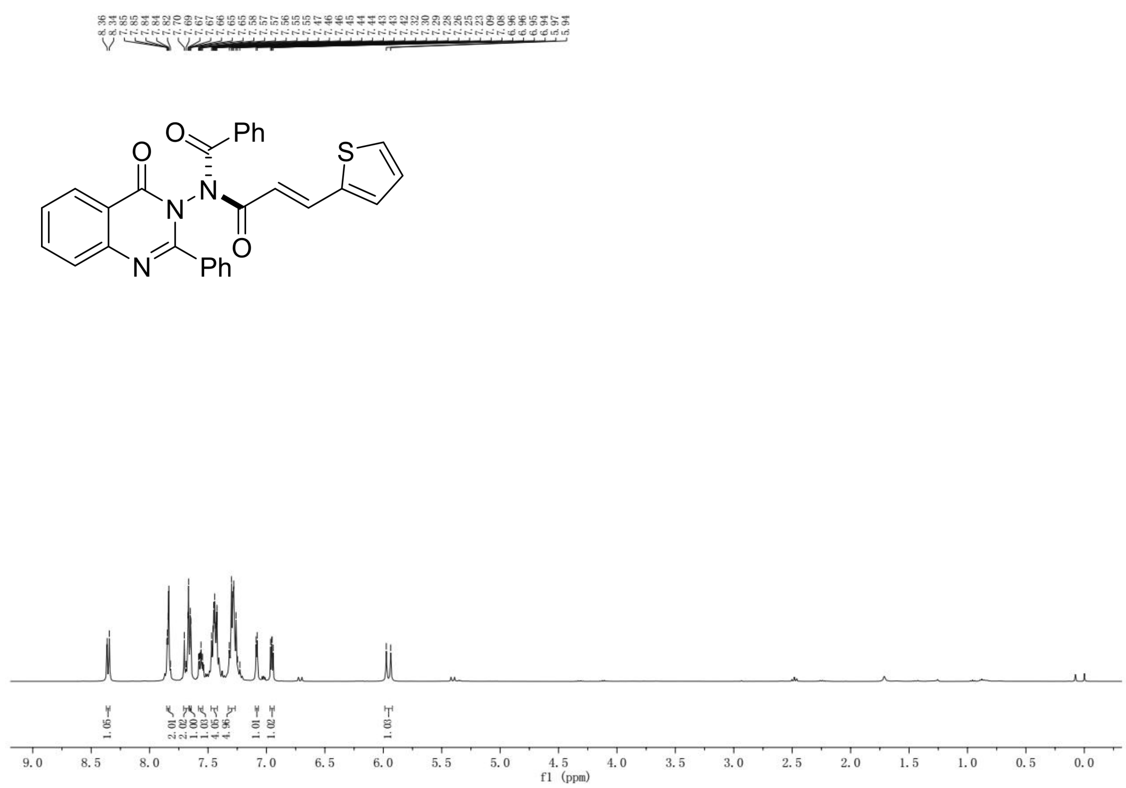

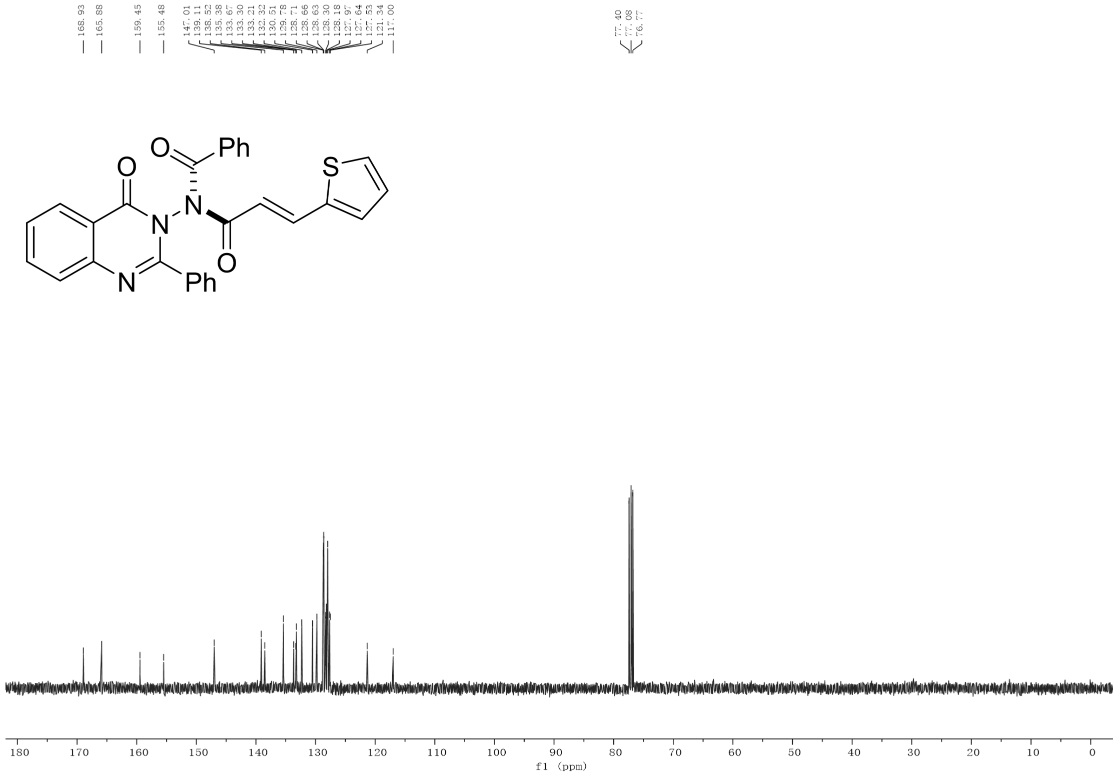

(*R*a,cis,cis)-*N*-(4-oxo-2-phenylquinazolin-3(4*H*)-yl)-*N*-(3-phenylpropanoyl)benzamide (3ah)

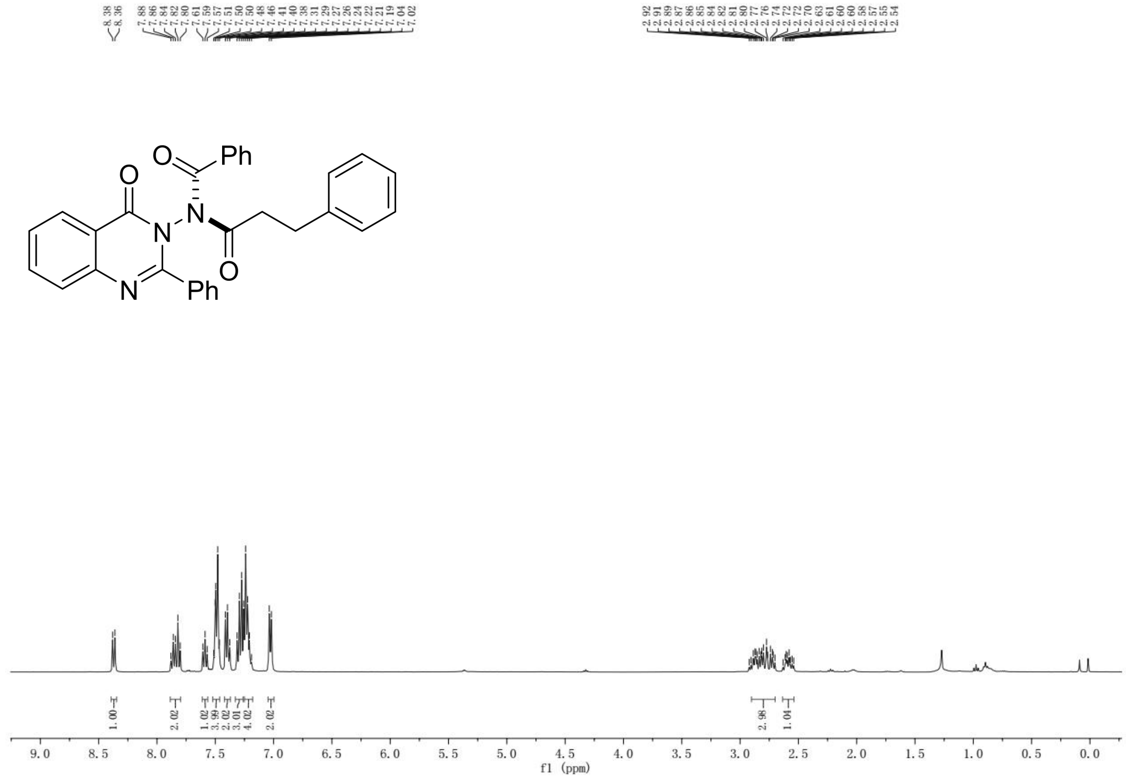

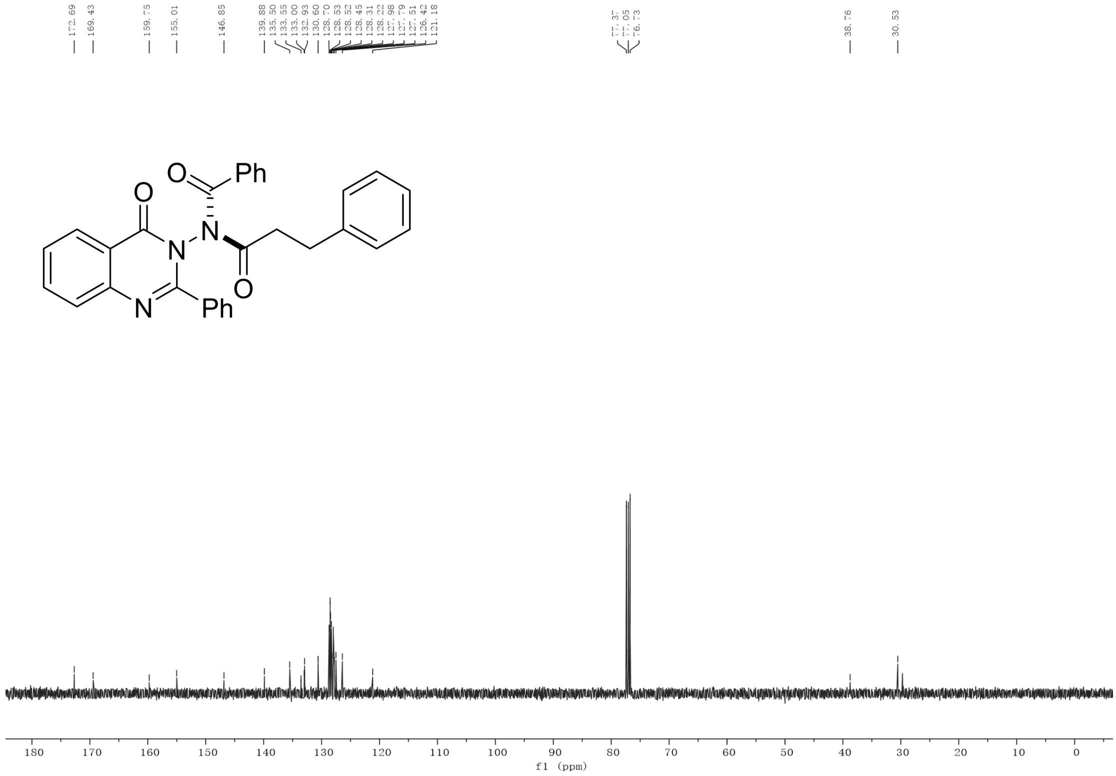

($R_{a,cis,cis}$)-*N*-acetyl-*N*-(4-oxo-2-phenylquinazolin-3(4*H*)-yl)benzamide (3ai)

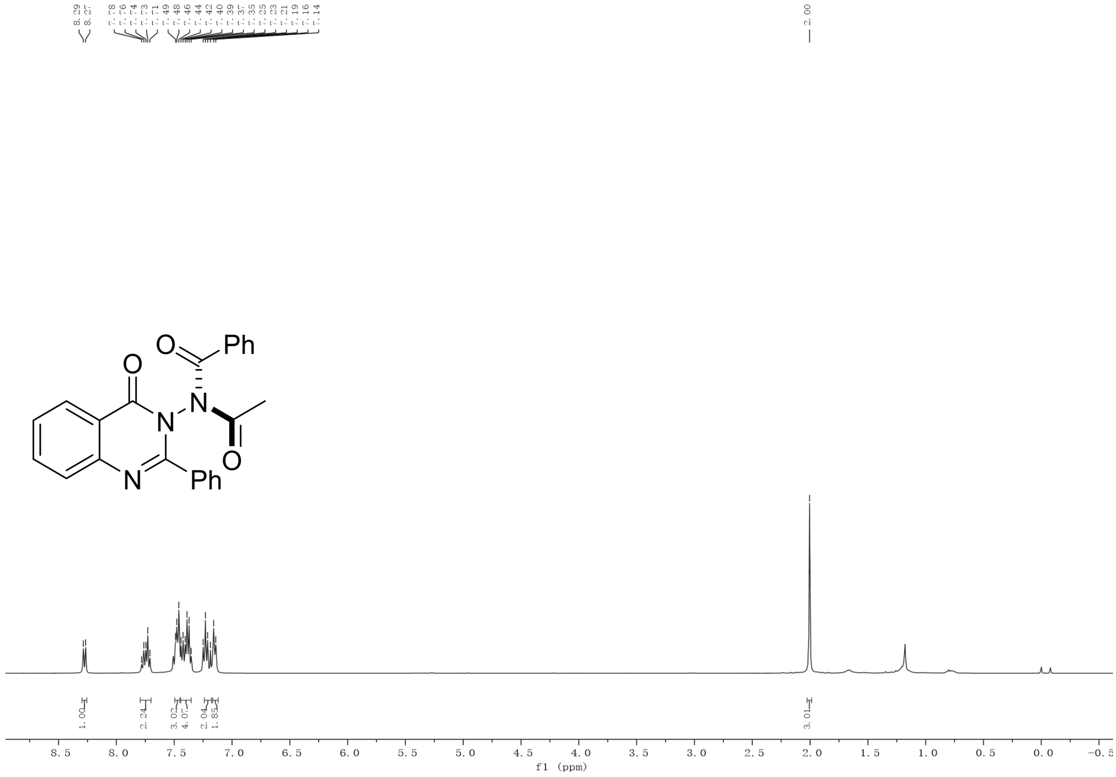

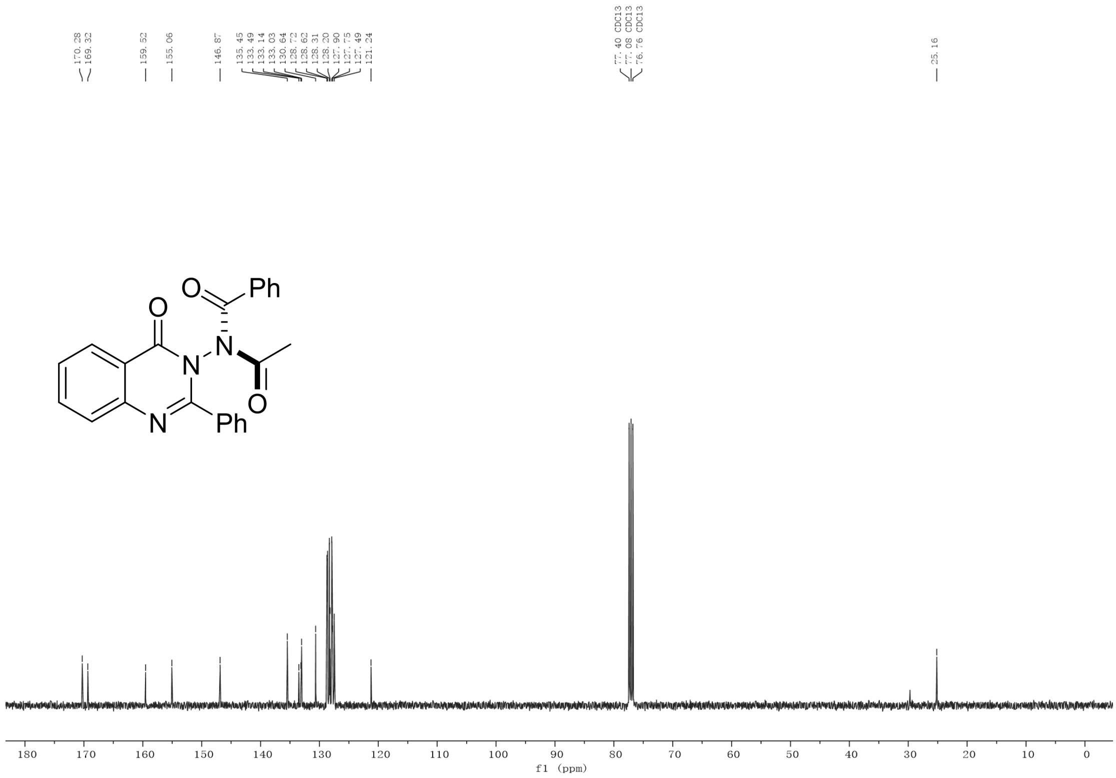

(*R*$_{a,cis,cis}$)- *N*-(4-oxo-2-phenylquinazolin-3(4*H*)-yl)-*N*-propionylbenzamide (3aj)

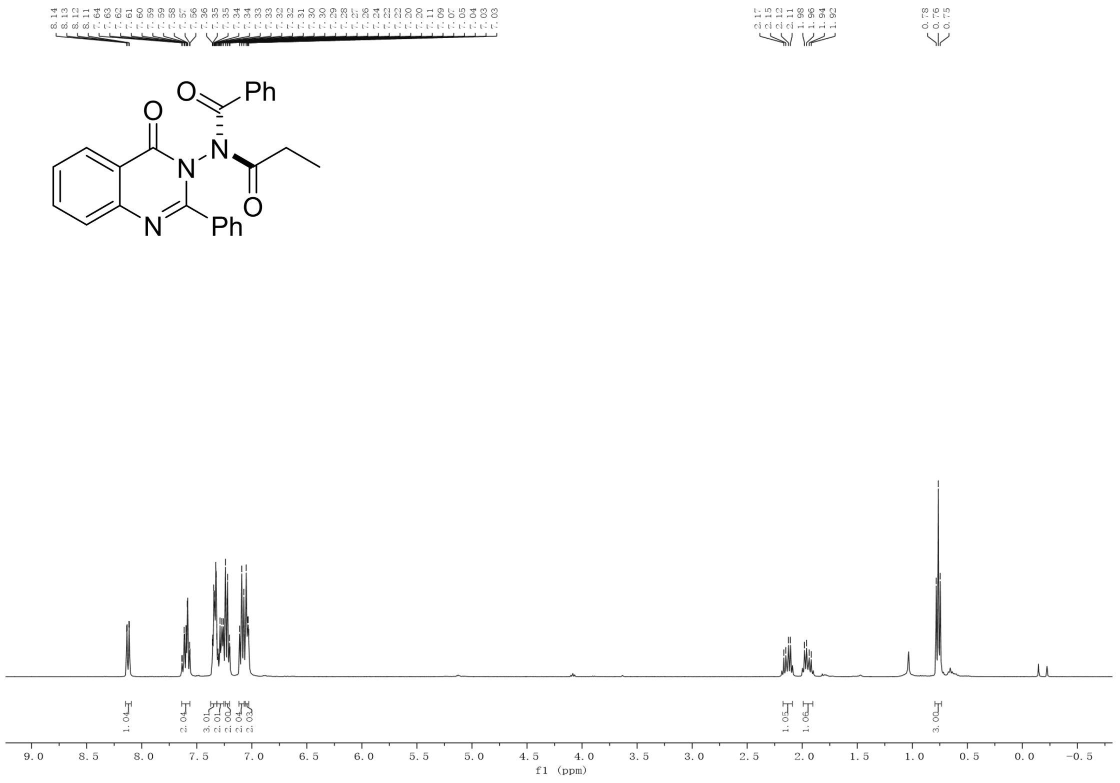

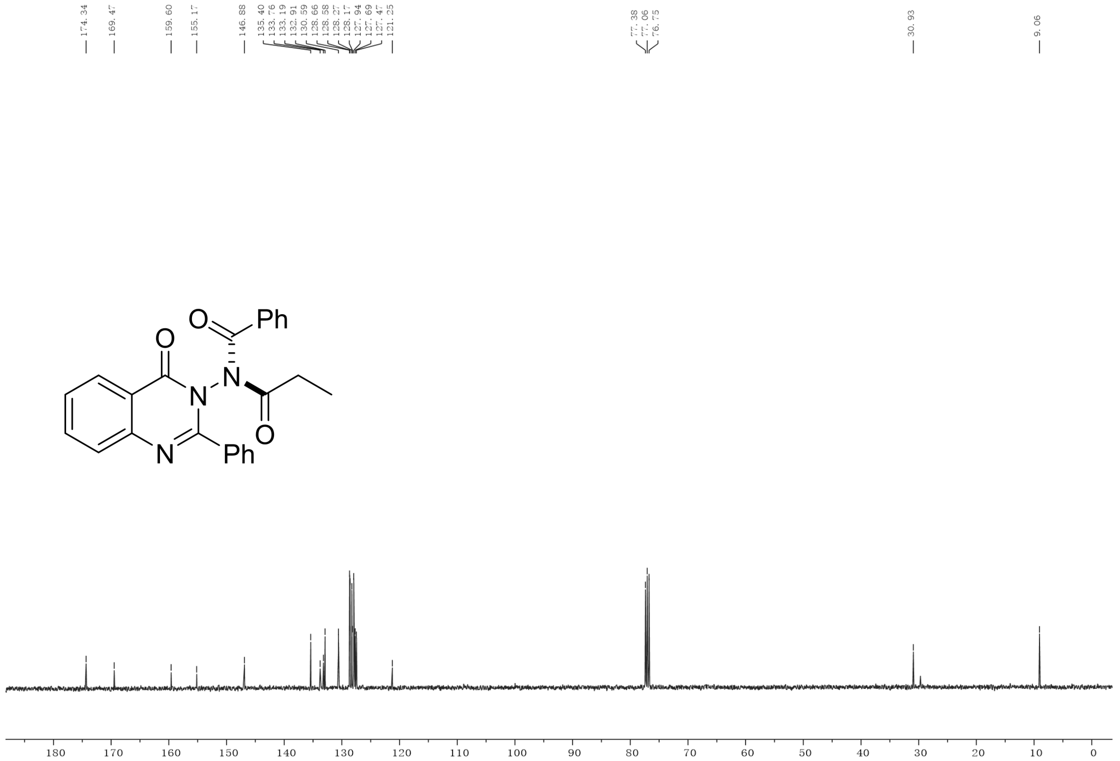

($R_{a,cis,cis}$)*N*-benzoyl-*N*-(4-oxo-2-phenylquinazolin-3(4*H*)-yl)-4-phenyl-4,5-dihydro-3*H*-3λ^3,4λ^3-pyrazole-3-carboxamide (4)

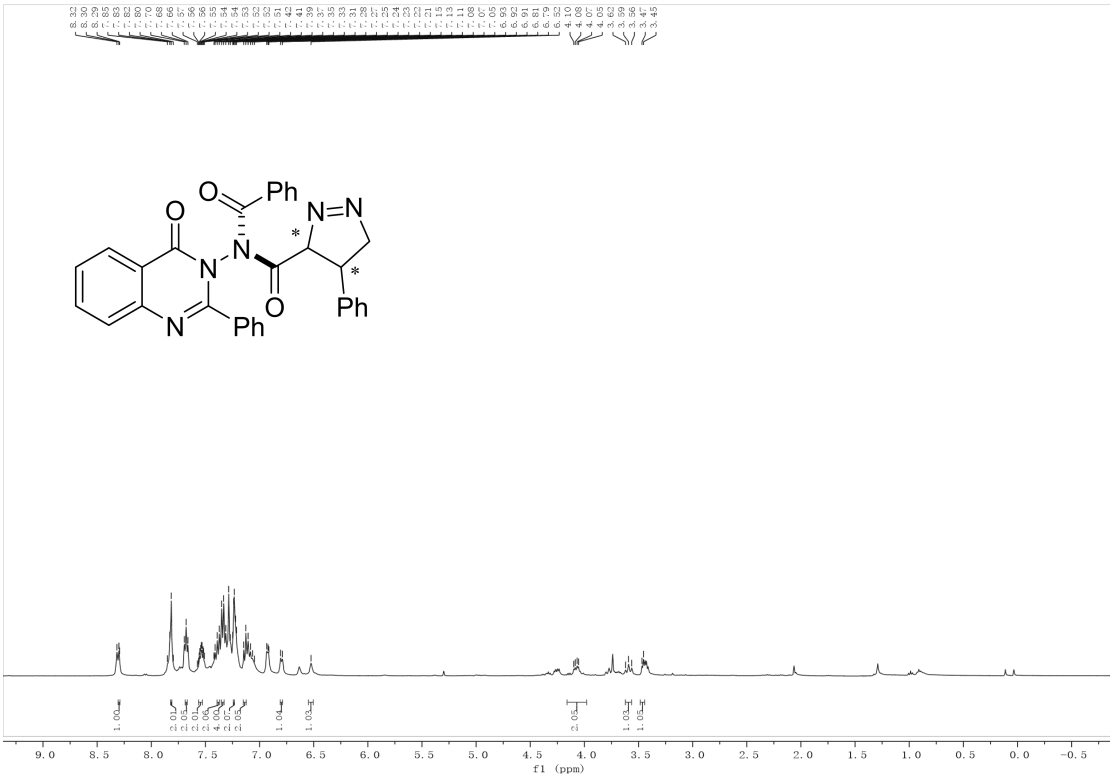

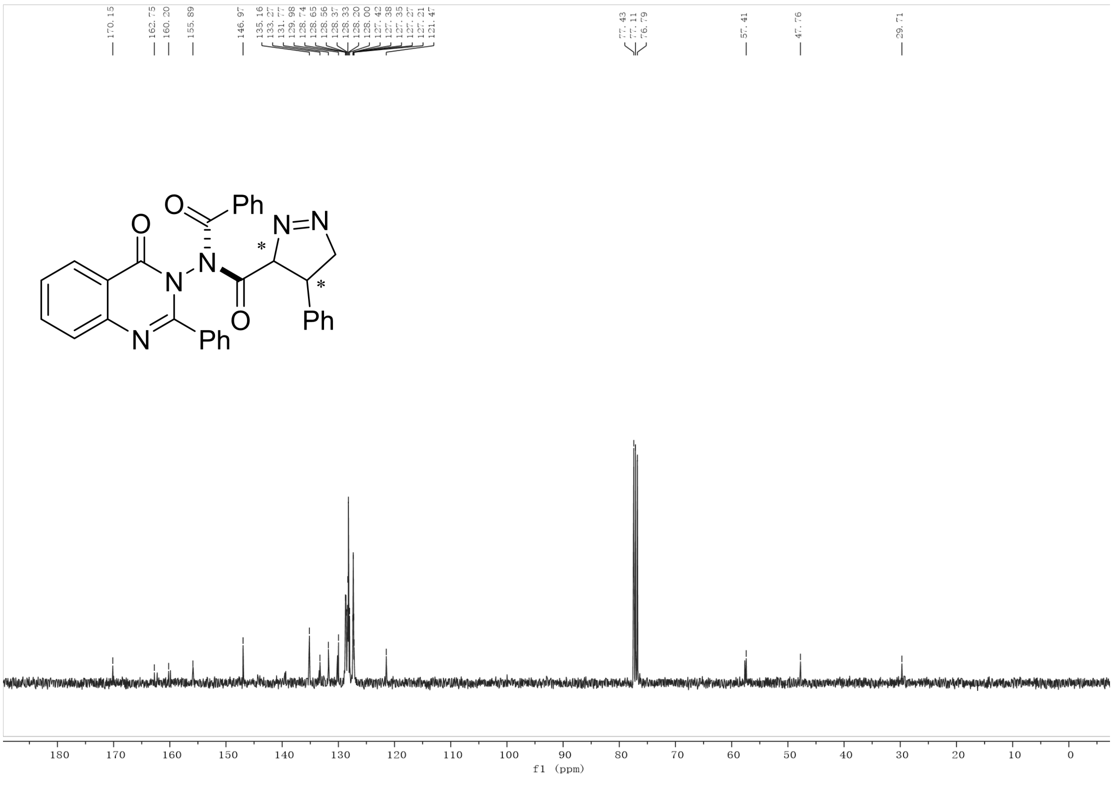

N-benzyl-*N*-(4-oxo-2-phenylquinazolin-3(4*H*)-yl)benzamide (8a)

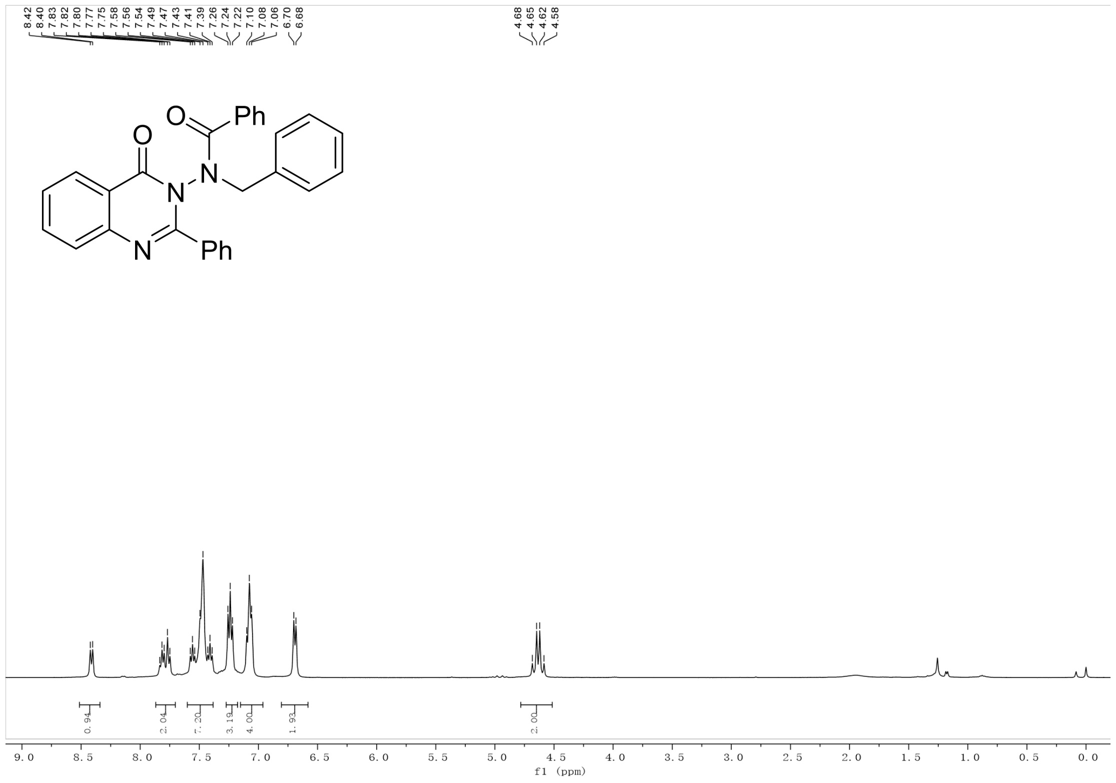

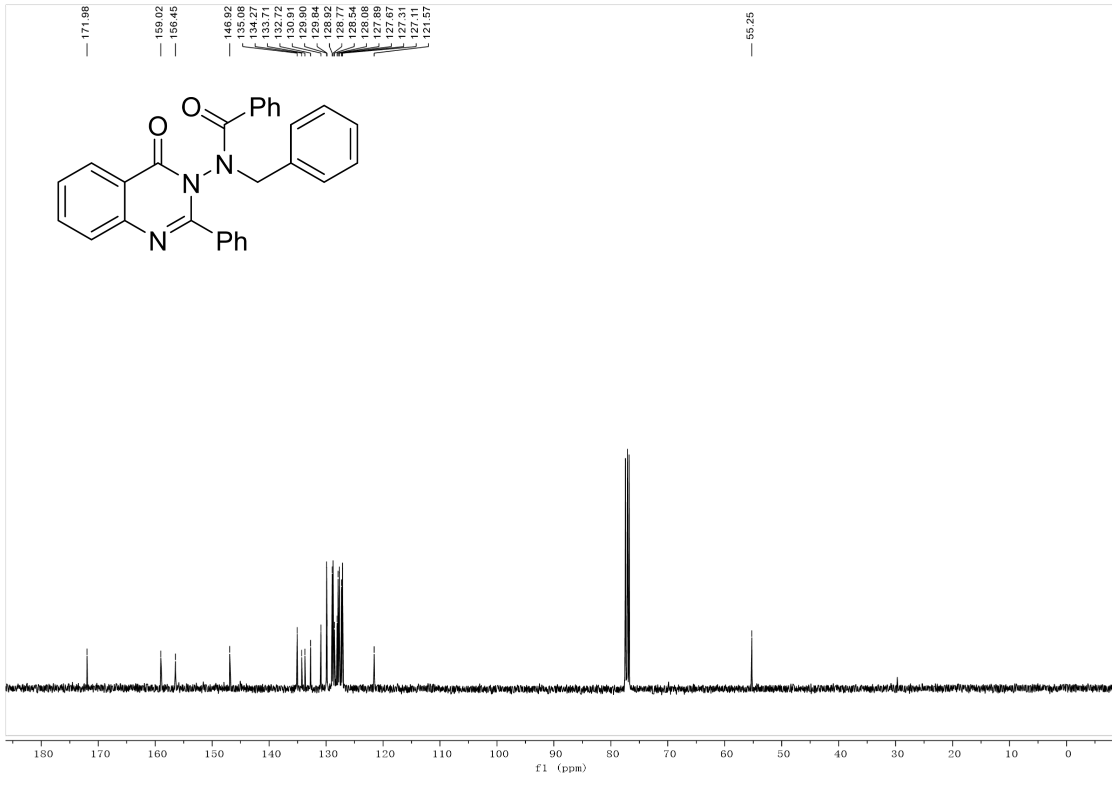

Methyl 2-((*N*-(4-oxo-2-phenylquinazolin-3(4*H*)-yl)benzamido)methyl)acrylate (8b)

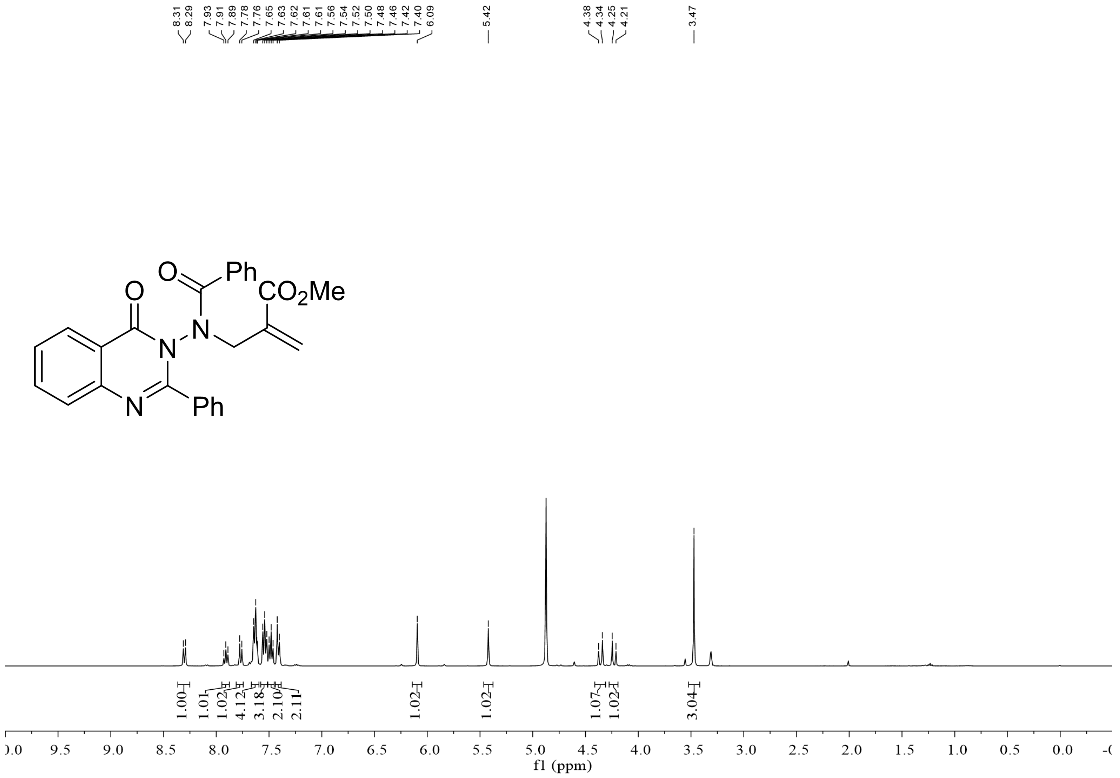

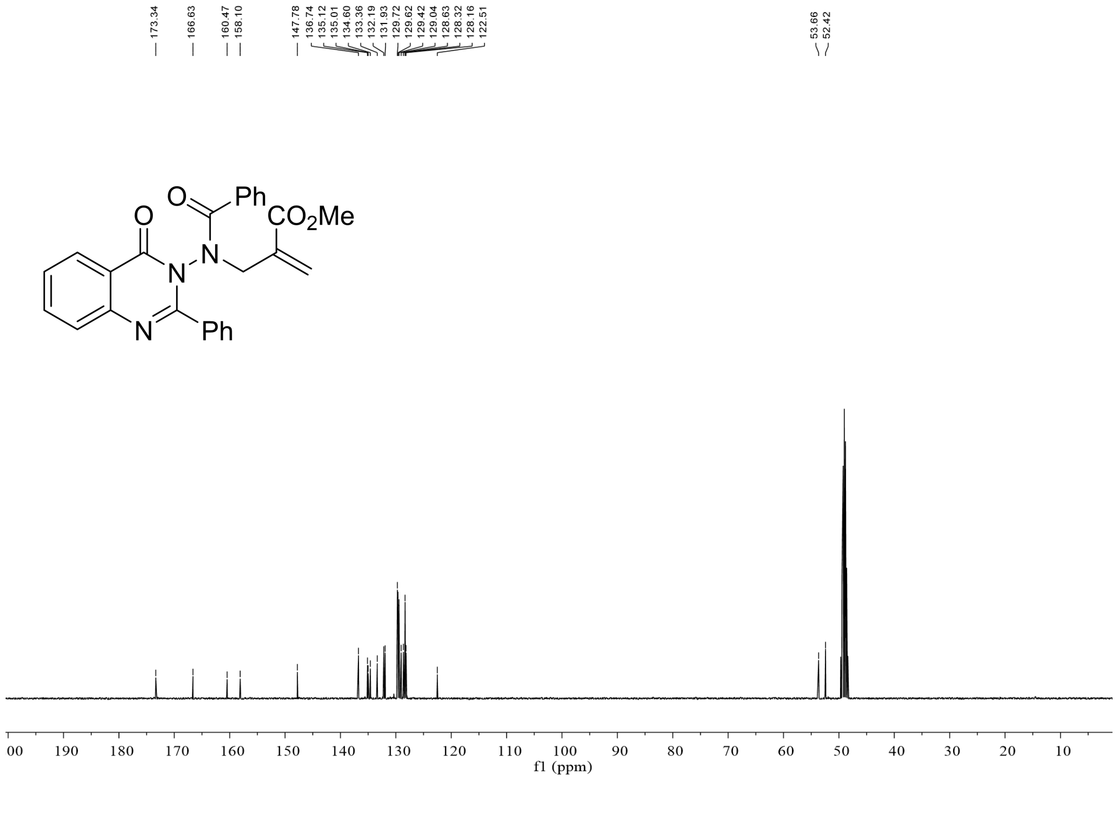

11. X-ray Analysis

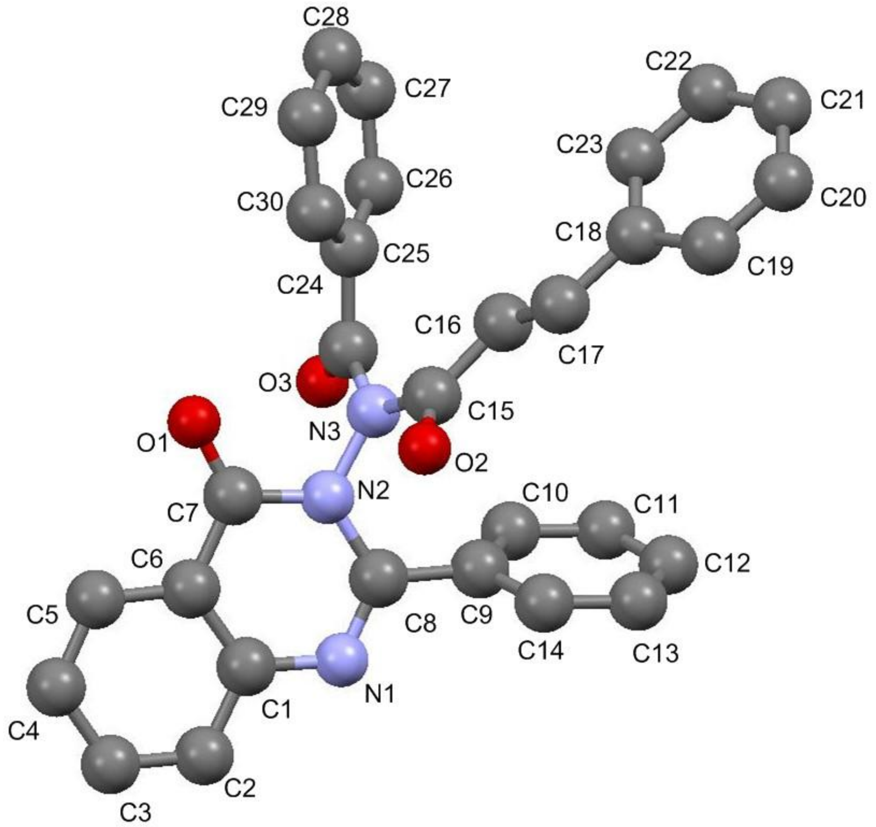

CCDC: 2045433 (**3aa**)	
Identification code	p20201106a
Empirical formula	$C_{30}H_{21}N_3O_3$
Formula weight	471.50
Temperature/K	294.15
Crystal system	triclinic
Space group	P-1
a/Å	9.65870(10)
b/Å	11.53350(10)
c/Å	12.42160(10)
α/°	114.3570(10)
β/°	102.2220(10)
γ/°	94.5930(10)
Volume/Å^3	1210.05(2)
Z	2
ρ_{calc}g/cm^3	1.294
μ/mm^{-1}	0.685
F(000)	492.0
Crystal size/mm^3	0.2 × 0.18 × 0.16
Radiation	CuKα (λ = 1.54184)

2Θ range for data collection/°	8.114 to 158.31
Index ranges	-11 ≤ h ≤ 11, -14 ≤ k ≤ 14, -15 ≤ l ≤ 15
Reflections collected	40355
Independent reflections	5058 [R_{int} = 0.0336, R_{sigma} = 0.0171]
Data/restraints/parameters	5058/0/325
Goodness-of-fit on F^2	1.058
Final R indexes [I>=2σ (I)]	R_1 = 0.0452, wR_2 = 0.1222
Final R indexes [all data]	R_1 = 0.0472, wR_2 = 0.1240
Largest diff. peak/hole / e $Å^{-3}$	0.14/-0.23

13. Computational Methods

All calculations were performed with Gaussian16 packages at *T* = 298.15K and *p*=1atm.[3] Unless noted, all energetics are reported in kcal/mol, and the bond lengths are reported in angstroms (Å). Structures were generated using CYLview[13] and VMD.[14] Geometry optimizations were conducted using M06-2X functional with 6-31g(d) basis set at gas phase. The frequency calculations were conducted at the same level of theory to confirm the nature of stationary points and obtain the thermal corrections. The thermal corrections derived from the vibrational frequency calculations was then added to the electronic energies calculated at B97D3/def2TZVPP-SMD(Toluene) level to obtain Gibbs free energies.[6,16,16] Intermolecular non-covalent interactions (NCI) in transition states were analyzed by Multiwfn[17] using Independent Gradient Model (IGM).[18] The C-H···S interaction could be also verified by atoms in molecules (AIM) analysis[19,20] using Multiwfn.[17]

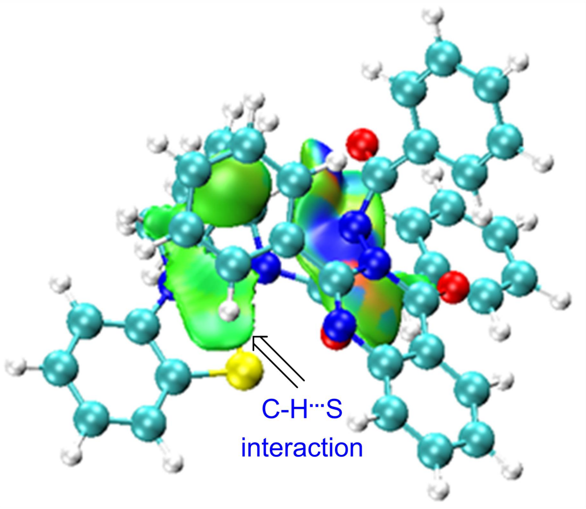

Figure S3. Independent Gradient Model (IGM) analysis of intermolecular interaction in **TS-major** (back view).

Cartesian coordinates
TS-major

Standard orientation:

Center Number	Atomic Number	Atomic Type	Coordinates (Angstroms) X	Y	Z
1	6	0	-6.259502	1.234151	-1.990969
2	6	0	-6.326607	2.004872	-0.826803
3	6	0	-5.186168	2.280306	-0.079959
4	6	0	-3.974957	1.760081	-0.529467
5	6	0	-3.899332	0.999815	-1.695608
6	6	0	-5.044340	0.723861	-2.435536
7	7	0	-2.724954	1.908112	0.076522
8	6	0	-1.713214	1.303800	-0.583438
9	16	0	-2.256582	0.455271	-2.017393
10	6	0	-2.552378	2.541465	1.387118
11	6	0	-1.293857	1.990830	2.028742
12	6	0	-0.107899	2.078789	1.071642
13	7	0	-0.456576	1.391020	-0.184636
14	6	0	0.367149	3.496194	0.831653
15	6	0	-0.165965	4.315997	-0.163755
16	6	0	0.283162	5.626548	-0.310646
17	6	0	1.270653	6.125998	0.533637
18	6	0	1.814426	5.308571	1.522734
19	6	0	1.366532	3.999386	1.669089
20	1	0	-7.163276	1.031257	-2.555136
21	1	0	-7.282161	2.399020	-0.497801
22	1	0	-5.241000	2.887546	0.816912
23	1	0	-4.986920	0.122990	-3.337345
24	1	0	-3.430617	2.285896	1.985553
25	1	0	-2.504657	3.628182	1.253392
26	1	0	-1.065496	2.554086	2.936668
27	1	0	-1.445262	0.941205	2.297898
28	1	0	0.704876	1.487513	1.511644
29	1	0	-0.919152	3.926960	-0.845890
30	1	0	-0.132883	6.253945	-1.092798
31	1	0	1.624369	7.145116	0.413770
32	1	0	2.596565	5.687081	2.173497
33	1	0	1.803286	3.345428	2.421451
34	6	0	0.583259	0.613738	-0.890606
35	6	0	1.959152	1.141605	-0.760283
36	8	0	0.189383	-0.051642	-1.840416
37	6	0	2.837085	0.873185	-1.731603

38	6	0	4.233790	1.322152	-1.759847
39	6	0	5.134281	0.684506	-2.621207
40	6	0	6.469903	1.069240	-2.669047
41	6	0	6.923632	2.109120	-1.862572
42	6	0	6.032925	2.762205	-1.010828
43	6	0	4.700253	2.375720	-0.961005
44	1	0	2.240851	1.671584	0.140051
45	1	0	2.504231	0.243733	-2.554591
46	1	0	4.778057	-0.131501	-3.244218
47	1	0	7.156337	0.557219	-3.336425
48	1	0	7.964817	2.414532	-1.899992
49	1	0	6.379719	3.580648	-0.387248
50	1	0	4.008732	2.904465	-0.310597
51	6	0	-3.942318	-0.601138	2.138359
52	6	0	-3.452285	-0.619133	3.442835
53	6	0	-3.176081	-1.124632	1.098911
54	6	0	2.394777	-3.324382	1.815382
55	6	0	2.739089	-4.420561	2.605018
56	6	0	-2.197871	-1.172986	3.701433
57	6	0	-1.899708	-1.635630	1.347008
58	6	0	1.631326	-0.847999	1.694484
59	6	0	2.026515	-2.119591	2.416148
60	6	0	-1.421407	-1.671719	2.660554
61	6	0	2.709534	-4.332118	3.992904
62	6	0	-1.138438	-2.225268	0.207535
63	6	0	-1.132172	-3.537423	-1.675365
64	6	0	-1.844452	-4.341779	-2.583339
65	6	0	2.035512	-2.026590	3.814105
66	6	0	2.359185	-3.126718	4.598596
67	6	0	0.986438	-2.474472	-0.960627
68	6	0	0.243474	-3.350017	-1.868950
69	6	0	-1.187998	-4.931360	-3.646374
70	6	0	0.901010	-3.945281	-2.952608
71	6	0	0.190785	-4.736256	-3.835802
72	1	0	-4.929416	-0.197763	1.926572
73	1	0	-4.051917	-0.224044	4.257704
74	1	0	-3.566977	-1.163553	0.086157
75	1	0	2.468712	-3.393714	0.737900
76	1	0	3.037517	-5.348540	2.126508
77	1	0	-1.819258	-1.216974	4.718048
78	1	0	2.970059	-5.193455	4.600794
79	1	0	-2.907218	-4.482173	-2.415515
80	1	0	-0.451082	-2.108799	2.869433
81	1	0	-1.742336	-5.554021	-4.342422

82	1	0	1.789348	-1.071181	4.267297
83	1	0	2.350526	-3.041613	5.681252
84	1	0	1.964683	-3.764299	-3.068354
85	1	0	0.693850	-5.206867	-4.674353
86	7	0	-1.809263	-2.963935	-0.615748
87	7	0	0.703197	-0.806363	0.713185
88	7	0	0.223450	-1.965928	0.117198
89	8	0	2.082024	0.217598	2.146413
90	8	0	2.165597	-2.198092	-1.075627

TS-minor

Standard orientation:

Center Number	Atomic Number	Atomic Type	Coordinates (Angstroms) X	Y	Z
1	6	0	-5.878776	1.476966	-1.937894
2	6	0	-5.955405	2.262397	-0.784195
3	6	0	-4.818762	2.558940	-0.039708
4	6	0	-3.601859	2.048058	-0.482883
5	6	0	-3.515570	1.284449	-1.644422
6	6	0	-4.656746	0.979025	-2.377602
7	7	0	-2.357565	2.195332	0.138621
8	6	0	-1.347048	1.568316	-0.492178
9	16	0	-1.865661	0.766082	-1.963717
10	6	0	-2.236096	2.770713	1.483134
11	6	0	-0.955039	2.280235	2.128564
12	6	0	0.206026	2.407577	1.140590
13	7	0	-0.101750	1.590557	-0.041956
14	6	0	0.508844	3.849733	0.765655
15	6	0	0.345285	4.350406	-0.525315
16	6	0	0.614316	5.690541	-0.801750
17	6	0	1.052024	6.542128	0.205941
18	6	0	1.228863	6.045981	1.496757
19	6	0	0.959102	4.711090	1.772219
20	1	0	-6.780875	1.251928	-2.496674
21	1	0	-6.916216	2.646165	-0.457965
22	1	0	-4.882772	3.168217	0.855022
23	1	0	-4.591431	0.359303	-3.266210
24	1	0	-3.107107	2.437195	2.052331
25	1	0	-2.255190	3.862735	1.394137
26	1	0	-0.750338	2.869508	3.025208
27	1	0	-1.055638	1.228063	2.405537
28	1	0	1.067455	1.944170	1.618989
29	1	0	0.029336	3.691207	-1.328250
30	1	0	0.485613	6.063859	-1.812979
31	1	0	1.263978	7.584065	-0.012102
32	1	0	1.584644	6.698490	2.288130
33	1	0	1.114167	4.325900	2.777603
34	6	0	0.861469	0.576580	-0.592048
35	6	0	2.303169	0.813772	-0.277937
36	8	0	0.509688	0.113971	-1.685067

37	6	0	3.211314	0.447752	-1.185506
38	6	0	4.664035	0.431502	-0.964599
39	6	0	5.528593	0.417806	-2.065240
40	6	0	6.907963	0.383686	-1.889063
41	6	0	7.445220	0.348867	-0.604653
42	6	0	6.593642	0.340695	0.499632
43	6	0	5.215741	0.376479	0.322832
44	1	0	2.584458	1.143496	0.713889
45	1	0	2.853530	0.095180	-2.152143
46	1	0	5.106270	0.433421	-3.066924
47	1	0	7.564029	0.379523	-2.754376
48	1	0	8.521239	0.315701	-0.463765
49	1	0	7.006175	0.292973	1.502967
50	1	0	4.555611	0.325915	1.184899
51	6	0	3.784688	-2.884837	-0.586659
52	6	0	4.131422	-2.903065	-1.934360
53	6	0	2.453673	-2.747838	-0.204806
54	6	0	0.046794	-3.231774	2.562187
55	6	0	-0.419693	-4.157175	3.493133
56	6	0	3.134791	-2.811991	-2.904031
57	6	0	1.451091	-2.644064	-1.173834
58	6	0	0.747050	-0.795156	2.025640
59	6	0	0.198568	-1.888669	2.919371
60	6	0	1.802444	-2.693924	-2.525732
61	6	0	-0.752450	-3.752103	4.781741
62	6	0	-0.002694	-2.512001	-0.866339
63	6	0	-2.184750	-3.054298	-1.343314
64	6	0	-3.096214	-3.769405	-2.144508
65	6	0	-0.112755	-1.496990	4.224741
66	6	0	-0.596742	-2.416398	5.146532
67	6	0	-1.757105	-1.415577	0.449784
68	6	0	-2.689592	-2.207246	-0.343847
69	6	0	-4.454987	-3.615688	-1.953850
70	6	0	-4.071356	-2.044945	-0.165011
71	6	0	-4.951302	-2.747010	-0.964603
72	1	0	4.555398	-2.952092	0.174671
73	1	0	5.174905	-2.977023	-2.225512
74	1	0	2.209442	-2.715861	0.850426
75	1	0	0.303152	-3.578457	1.567042
76	1	0	-0.522499	-5.198676	3.204810
77	1	0	3.395478	-2.828086	-3.958021
78	1	0	-1.124668	-4.475291	5.501060
79	1	0	-2.693916	-4.426298	-2.908386
80	1	0	1.015918	-2.613591	-3.267820

81	1	0	-5.150226	-4.168812	-2.578535
82	1	0	0.044888	-0.459369	4.499262
83	1	0	-0.845094	-2.093833	6.153136
84	1	0	-4.413961	-1.357233	0.601972
85	1	0	-6.022142	-2.630262	-0.832843
86	7	0	-0.834108	-3.197873	-1.583143
87	7	0	0.524890	-0.791621	0.692448
88	7	0	-0.404364	-1.661309	0.160479
89	8	0	1.412694	0.102965	2.556743
90	8	0	-2.086116	-0.577726	1.279614

----

12. References

(1) N. Armesto, M. Ferrero, S. Fernandez and V. Gotor, *J. Org. Chem.*, 2003**,** **68**, 5784.

(2) Z.-J. Huang, X. Huang, B.-S. Li, C.-L. Mou, S. Yang, B.-A. Song and Y.-G. Chi, *J. Am. Chem. Soc.* 2016, **138**, 7524.

(3) Gaussian 16, Revision C.01, M. J. Frisch, G. W. Trucks, H. B. Schlegel, G. E. Scuseria, M. A. Robb, J. R. Cheeseman, G. Scalmani, V. Barone, G. A. Petersson, H. Li, X. Nakatsuji, M. Caricato, A. V. Marenich, J. Bloino, B. G. Janesko, R. Gomperts, B. Mennucci, H. P. Hratchian, J. V. Ortiz, A. F. Izmaylov, J. L. Sonnenberg, D. Williams-Young, F. Ding, F. Lipparini, F. Egidi, J. Goings, B. Peng, A. Petrone, T. Henderson, D. Ranasinghe, V. G. Zakrzewski, J. Gao, N. Rega, G. Zheng, W. Liang, M. Hada, M. Ehara, K. Toyota, Fukuda, R. J. Hasegawa, M. Ishida, T. Nakajima, Y. Honda, O. Kitao, H. Nakai, T. Vreven, K. Throssell, J. A. Montgomery, J. Jr., E. Peralta, F. Ogliaro, M. J. Bearpark, J. J. Heyd, E. N. Brothers, K. N. Kudin, V. N. Staroverov, T. A. Keith, R. Kobayashi, J. Normand, K. Raghavachari, A. P. Rendell, J. C. Burant, S. S. Iyengar, J. Tomasi, M. Cossi, J. M. Millam, M. Klene, C. Adamo, R. Cammi, J. W. Ochterski, R. L. Martin, K. Morokuma, O. Farkas, J. B. Foresman, and D. J. Fox, Gaussian, Inc., Wallingford CT, 2019.

(4) Y. Zhao and D. G. Truhlar, *Acc. Chem. Res.* 2008*,* **41**, 157.

(5) Y. Zhao and D. G. Truhlar, *Theor. Chem. Acc.* 2008, **120**, 215.

(6) A. V. Marenich, C. J. Cramer, and D. G. Truhlar, *J. Phys. Chem. B* 2009, **113**, 6378-6396.

(7) S. Grimme, *Chem. Eur. J.*, 2012, **18**, 9955.

(8) I. M. Alecu, J. Zheng, Y. Zhao and D. G. Truhlar, *J. Chem. Theory Comput.*, 2010, **6**, 2872.

(9) R. F. Ribeiro, A. V. Marenich, C. J. Cramer and D. G. Truhlar, *J. Phys. Chem. B* 2011, **115**, 14556.

(10) G. Luchini, J. V. Alegre-Requena, I. Funes-Ardoiz and R. S. Paton, GoodVibes: Automated Thermochemistry for Heterogeneous Computational Chemistry Data. *F1000Research*, 2020, **9**, 291.

(11) F. Weigend, R. Ahlrichs, *Phys. Chem. Chem. Phys.*, 2005, **7**, 3297.

(12) F. Weigend, *Phys. Chem. Chem. Phys.* 2006, **8**, 1057.

(13) C. Y. Legault, *CYLview, 1.0b*; Université de Sherbrooke: Sherbrooke (Québec) Canada, 2009.

(14) W. Humphrey, A. Dalke and K. Schulten, *J. Molec. Graphics*, 1996, **14**, 33.

(15) A. D. Becke, *J. Chem. Phys.*, 1997, **107**, 8554.

(16) S. Grimme, *WIREs: Comput. Mol. Sci.*, 2011, **1**, 211.

(17) T. Lu and F. Chen, *J. Comp. Chem.*, 2012, **33**, 580.

(18) C. Lefebvre, G. Rubez, H. Khartabil and J.-C. Boisson, *Phys. Chem. Chem. Phys.*, 2017, **19**, 17928.

(19) R. F. W. Bader, *Acc. Chem. Res.*, 1985, **18**, 9.
(20) R. F. W. Bader, *Chem. Rev.*, 1991, **91**, 893.
